# Synthesis of Nitrostyrylthiazolidine-2,4-dione Derivatives Displaying Antileishmanial Potential

**DOI:** 10.3390/ph17070878

**Published:** 2024-07-03

**Authors:** Omar Khoumeri, Sébastien Hutter, Nicolas Primas, Caroline Castera-Ducros, Sandra Carvalho, Susan Wyllie, Mohamed Lotfi Efrit, Dimitri Fayolle, Marc Since, Patrice Vanelle, Pierre Verhaeghe, Nadine Azas, Hussein El-Kashef

**Affiliations:** 1 Team Pharmaco-Chimie Radicalaire, Faculté de Pharmacie, Aix Marseille University, CNRS, ICR UMR 7273, 27 Boulevard Jean Moulin, CS30064, CEDEX 05, 13385 Marseille, France; omar.khoumeri@univ-amu.fr (O.K.); caroline.ducros@univ-amu.fr (C.C.-D.); patrice.vanelle@univ-amu.fr (P.V.); 2 IHU Méditerranée Infection, UMR RITMES, TEAM-VEPTE, Aix Marseille University, 19-21 Boulevard Jean Moulin, 13005 Marseille, France; sebastien.hutter@univ-amu.fr (S.H.); nadine.azas@univ-amu.fr (N.A.); 3 Service Central de la Qualité et de l’Information Pharmaceutiques, Hôpital de la Conception, AP-HM, 147 Boulevard Baille, 13005 Marseille, France; 4 Wellcome Centre for Anti-Infectives Research, School of Life Sciences, University of Dundee, Dow Street, Dundee DD1 5EH, UK; s.z.carvalho@dundee.ac.uk (S.C.); s.wyllie@dundee.ac.uk (S.W.); 5 Laboratoire de Synthèse Organique et Hétérocyclique Sélective-Evaluation D’activité Biologique, LR17ES01, Faculté des Sciences de Tunis, Université de Tunis El Manar, Campus Universitaire, Tunis 2092, Tunisia; medlotfi.efrit@fst.utm.tn; 6 Normandie Université, UNICAEN, CERMN, DruiD Platform, Boulevard Becquerel, 14000 Caen, France; dimitri.fayolle@unicaen.fr (D.F.); marc.since@unicaen.fr (M.S.); 7 CNRS, Département de Pharmacochimie Moléculaire UMR 5063, University Grenoble Alpes, 38041 Grenoble, France; pierre.verhaeghe@univ-grenoble-alpes.fr; 8 LCC-CNRS, UPR8241, Laboratoire de Chimie de Coordination, Université de Toulouse, CNRS, UPS, 31400 Toulouse, France; 9 Chemistry Department, Faculty of Science, Assiut University, Assiut 71516, Egypt; 10 Faculty of Pharmacy, Sphinx University, Regional Road, New Assiut 71515, Egypt

**Keywords:** visceral leishmaniasis, *L. infantum*, thiazolidinedione ring, nitrostyryl moiety

## Abstract

A series of 61 thiazolidine-2,4-diones bearing a styryl group at position 5 was synthesized in 2–5 steps and their structure was proved by elemental and spectral analyses. The compounds obtained were evaluated in vitro against the promastigote stage of the kinetoplastid parasite *Leishmania infantum* and the human HepG2 cell line, to determine selectivity indices and to compare their activities with those of antileishmanial reference drugs. The study of structure–activity relationships indicated the potential of some derivatives bearing a nitro group on the phenyl ring, especially when located at the *meta* position. Thus, among the tested series, compound **14c** appeared as a hit compound with good antileishmanial activity (EC_50_ = 7 µM) and low cytotoxicity against both the hepatic HepG2 and macrophage THP-1 human cell lines (CC_50_ = 101 and 121 µM, respectively), leading to good selectivity indices (respectively, 14 and 17), in comparison with the reference antileishmanial drug compound miltefosine (EC_50_ = 3.3 µM, CC_50_ = 85 and 30 µM, SI = 26 and 9). Regarding its mechanism of action, among several possibilities, it was demonstrated that compound **14c** is a prodrug bioactivated, predominantly by *L. donovani* nitroreductase 1, likely leading to the formation of cytotoxic metabolites that form covalent adducts in the parasite. Finally, compound **14c** is lipophilic (measured CHI LogD_7.7_ = 2.85) but remains soluble in water (measured PBS solubility at pH_7.4_ = 16 µM), highlighting the antileishmanial potential of the nitrostyrylthiazolidine-2,4-dione scaffold.

## 1. Introduction

Leishmaniasis is a neglected tropical disease, caused by a parasite belonging to the genus *Leishmania*. The infection is transmitted through a bite of a female Phlebotomine sandfly infected with the protozoan. According to the World Health Organization (WHO), leishmaniasis is the second major tropical parasitic killer disease after malaria [1]. Currently, it affects 12 million people in 98 countries mainly in tropical and sub-tropical regions, and in southern Europe, especially around the Mediterranean area. There are three clinical forms of leishmaniasis: cutaneous (CL), mucocutaneous (MCL), and visceral (VL). The first type is the most common while the third one is the most severe, mainly caused by *Leishmania donovani* and *Leishmania infantum*, targeting visceral organs such as the spleen and liver [1]. Currently, there is no vaccine available for any form of human leishmaniasis [1]. Moreover, there is a limited number of available drugs representing major therapeutic limitations including severe side effects and increasing parasite drug-resistance issues.

The available drugs for the treatment of VL include sodium stibogluconate (pentavalent antimonials), miltefosine, pentamidine, paromomycin, and amphotericin B. However, these drugs are outdated, showing serious side effects such as renal or cardiac toxicity, teratogenicity, and parasite resistance. Moreover, today, miltefosine remains the only orally available antileishmanial drug against VL. This matter calls for the discovery of new antileishmanial chemical entities, with both original mechanisms of action and oral route of administration, to improve and facilitate the therapeutic care of the infected populations, especially in endemic developing countries [2,3,4].

Many studies have been performed to evaluate the antileishmanial activity of several nitrogen-containing heterocycles such as pyridines [5], pyrimidines [5], isoxazole [6,7,8], pyrazoles [9,10,11], thiazoles [12], 1,2,4-triazoles [13], 1,3,4-oxadiazoles [14], thiohydantoins [15], quinolines [2,16,17,18], quinazolines [19,20], pyrazolo[3,4-*d*]pyridine [21,22], pyrazolo[3,4-*d*]pyridazines [23], and imidazo[1,2-*a*]pyridines [24,25,26].

Thiazolidinediones (TZDs), also known as Glitazones, are an important class of heterocycles abundantly reported in the literature. Initially, certain derivatives were used as insulin sensitizers for the treatment of Type 2 diabetes mellitus (T2DM) due to their PPAR-γ activation properties [27]. However, the TZDs, clinically used as anti-diabetes mellitus drugs, were later reported to suffer from several serious side effects and thus many of them were withdrawn from the market [28]. However, pioglitazone is still marketed in the USA and Lobeglitazone was recently approved in India for the treatment of Type 2 diabetes.

A literature survey revealed that TZDs possess a very wide range of biological activities, which are included in the reference [29], such as antidiabetic, anti-infectious, anti-inflammatory, analgesic, antioxidant, immunomodulatory, overactive bladder inhibitory and insecticide activities. TZDs also possess inhibitory activity against tyrosinase, hyperlipidemia, and acute liver injury. Furthermore, it was reported that TZDs affected cancer development, progression and metastasis, among which the Raf/MEK/extracellular signal-regulated kinase (ERK), phosphatidylinositol 3-kinase (PI3K)/AKT, Wnt signal transduction pathways, PIM-1 and PIM-2 protein kinases overexpression, receptor-stimulated genes and peroxisome proliferator-activated receptors (PPARs) signaling cascades which are the most commonly up-regulated in human cancers [29]. It is important to note that the TZD derivative S49076 has reached Phase I clinical trials against solid tumors [30]. On the other hand, the potential of TZD against leishmaniasis has not yet been explored, with the exception of a single study [31].

In the present work, we synthesized a library of sixty-one 5-stryryl-3-substituted thiazolidine-2,4-dione derivatives, in an endeavor to evaluate their in vitro antileishmanial activity.

## 2. Results and Discussion

### 2.1. Chemistry

**Scheme 1:**
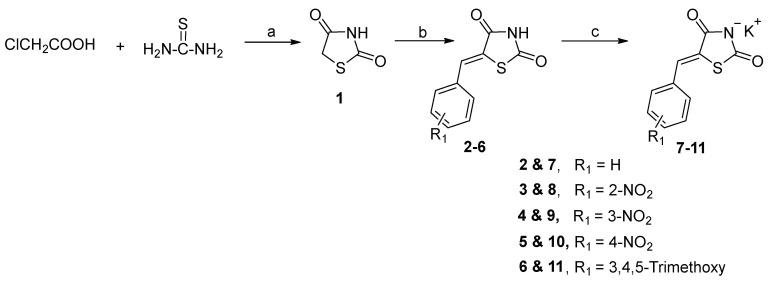
Synthesis of the starting material **1**. Reagents and conditions: (a) i. water, 0–5 °C, 20 min, ii. HCl, reflux, 12 h. (b) aromatic aldehyde, anhydrous toluene, cat. piperidinium acetate, reflux, 15 h. (c) KOH/ethanol-dioxane, boiling, 20 min. Yields = 75–90% for **2–6**, and 69–90% for **7–11**.

The 1,3-thiazolidine-2,4-dione core heterocycle (1), used as a starting material, was prepared via the reaction of chloroacetic acid with thiourea, as reported in our previous work [29] (Scheme 1).

**Scheme 2:**
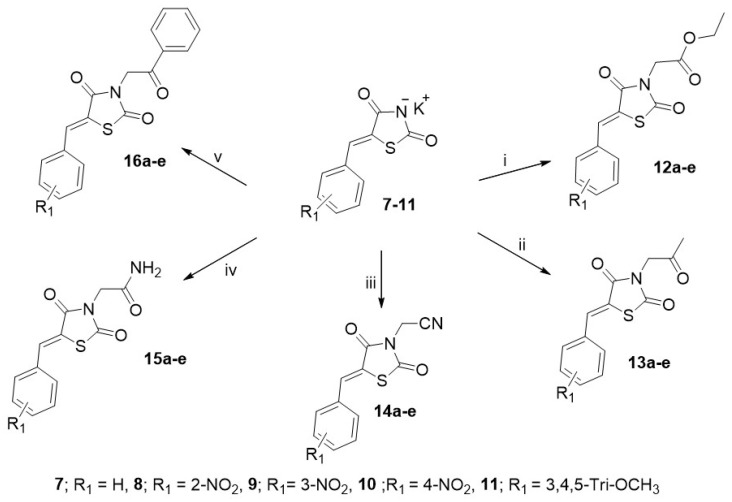
General synthesis of compounds **12–16**. Reagents and conditions: heating in DMF at reflux for 6 h with (i) ethyl chloroacetate, (ii) chloroacetone, (iii) chloroacetonitrile, (iv) 2-chloroacetamide, (v) phenacyl bromide. Yields = 62–95%.

Compound **1** is a versatile heterocycle that allows the synthesis of various thiazolidinediones by virtue of its two reactive centers: (A) the acidic NH hydrogen at position 3 and (B) the active-methylene group at position 5. Thus, when this 5-methylene group was allowed to react, under Knoevenagel reaction conditions, with the aromatic aldehydes; benzaldehyde, *o*-, *m*-, *p*-nitrobenzaldehyde, and 3,4,5-trimethoxy benzaldehyde, the corresponding 5-stryryl-1,3-thiazolidine-2,4-diones **2–6** were obtained (Scheme 1) in 75–90% yields. These latter compounds **2–6**, could be easily converted into the corresponding potassium salts **7–11** in 69–90% yields upon treatment with KOH in boiling ethanol or ethanol-dioxane mixture (Scheme 1). The reaction of the nucleophilic *N*-anion of the latter potassium salts **7–11** with a series of halo-compounds namely ethyl chloroacetate, chloroacetone, chloroacetonitrile, 2-chloroacetamide, and phenacyl bromide in refluxing DMF, resulted in the formation of a series of the corresponding target (*Z*)-*N*-substituted 5-styrylthiazolidinediones **12a-e** to **16a-e** (Scheme 2, Table 1) in 62–95% yields.

**Scheme 3:**
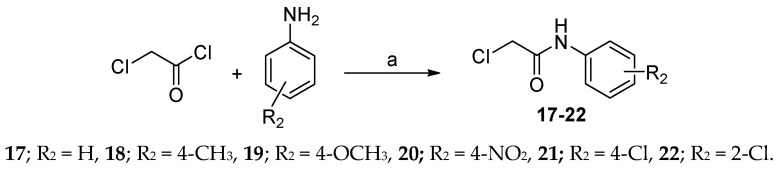
Synthesis of *N*-aryl-2-chloroacetamides **23–28**. Reagents and conditions: (a) dry DMF, 0–5 °C, 3 h, then rt, 3 h. Yields = 85–96%.

Some 2-chloro-*N*-arylacetamides (**17–22**) were prepared by chloroacetylation of the aromatic amines; aniline, 4-methylaniline, 4-methoxyaniline, 4-nitroaniline, 4-chloroaniline and 2-chloroaniline, using chloroacetyl chloride in dry DMF (Scheme 3).

**Scheme 4:**
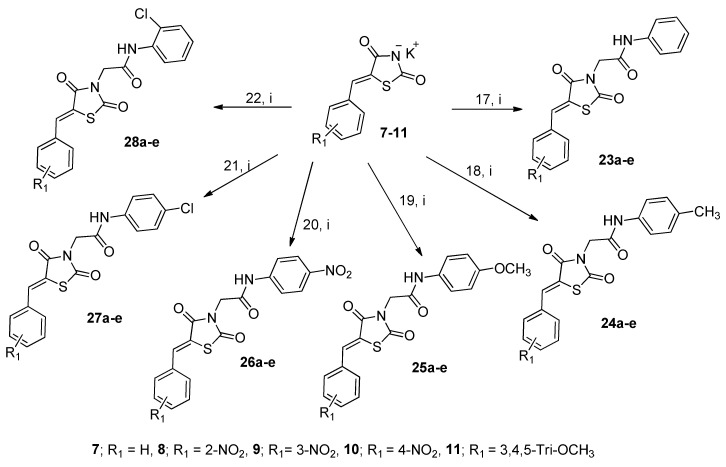
General synthesis of thiazolidinediones **23a-e** to **28a-e**. Reagents and conditions: (i) DMF, reflux, 6 h. Yields = 69–94%.

The 2-chloro-*N*-arylacetamides **17–22** were then reacted via their labile chlorine atoms with the potassium salts **7–11** in boiling DMF, affording another series of 30 new thiazolidine-2,4-dione derivatives **23a-e** to **28a-e**, as shown in Scheme 4 and Table 2.

All the obtained thiazolidinediones were found to possess exclusively a *Z*-configuration as evidenced by their ^1^H-NMR spectra. The methylidene =CH- proton of these compounds appeared as a deshielded singlet at 8.25–7.79 ppm. This deshielding is caused by the anisotropic effect of the neighboring C=O group at the 4-position. On the other hand, no signal indicating the presence of the *E*-isomer was observed. The =CH- proton of the *E*-isomer should appear around 6.2–6.3 ppm, due to the less deshielding effect of the S atom of the TZD ring [29]. Moreover, the *Z*-configuration is more thermodynamically stable due to the hydrogen bonding between methylidene hydrogen and the 4-C=O group. The X-ray single crystal analysis of compound **6** is a further unambiguous confirmation of the Z-configuration of **2–6** [32].

**Scheme 5:**
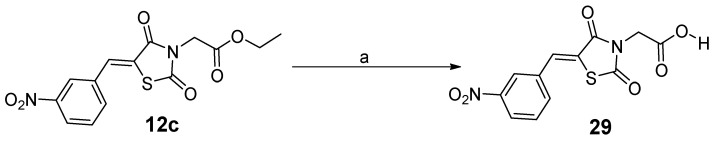
Acidic hydrolysis of ethyl ester **12c** into **29**. Reagents and conditions: (a) heating under reflux in glacial acetic acid and conc. HCl for 4 h. Yield = 90%.

Finally, for discussing structure–activity relationships, the ethyl ester **12c** was hydrolyzed into the corresponding carboxylic acid **29** (Scheme 5), following a procedure reported by Maccari et al. [33].

It is of interest to note that the ^1^H-NMR spectrum of acid **29** did not show the signal corresponding to the carboxylic acid OH group. This could be explained by a rapid exchange of the acidic proton with the deuterium of the deuterated solvent DMSO_d6_. However, its ^13^C-NMR spectrum showed the expected 12 carbons which includes the carbonyl carbon of the -COOH group (cf. Supplementary Material).

### 2.2. In Vitro Assays

All the compounds belonging to the 5-stryryl-1,3-thiazolidine-2,4-dione series (apart from corrosive potassium salts **7–11**) were first evaluated in vitro on the promastigote stage of *L. infantum*, to determine their half-maximal effective concentrations (EC_50_), and to compare the obtained results with those of some antileishmanial reference drug compounds: amphotericin B (the most potent of all antileishmanial drugs), miltefosine (the unique orally available antileishmanial drug) and fexinidazole sulfone (the active metabolite of fexinidazole). Out of these 61 tested compounds, 22 were found to be insufficiently soluble in the aqueous culture medium to be properly evaluated. Among the 38 remaining molecules, 21 showed moderate to good antileishmanial activity with EC_50_ values ≤ 30 µM, including 19 nitroaromatic derivatives, as presented in Table 3.

Then, the cytotoxicity of these 21 bioactive molecules was evaluated against the human hepatic HepG2 cell line and compared to the one of doxorubicin (chosen as a reference cytotoxic drug compound), by determining their 50% cytotoxic concentrations (CC_50_), to calculate the corresponding selectivity indices (SI = CC_50_/EC_50_). Eight molecules showed low cytotoxicity (CC_50_ ≥ 50 µM) and 4 hit compounds bearing a nitro group on the benzene ring of the styryl moiety (**5**, **12c**, **12d**, **14c**) were thus highlighted, presenting good activities (5.5 ≤ EC_50_ ≤ 14 µM), low cytotoxicities (101 ≤ CC_50_ ≤ 125 µM) and good selectivity index values (10 ≤ SI ≤ 23), as shown in Table 3. This in vitro biological activity profile appeared rather similar to that of the miltefosine reference drug (EC_50_ = 3.3 µM, CC_50_ = 85 µM, SI = 26) but remained far from reaching the high potency of amphotericin B (EC_50_ = 0.06 µM).

In terms of structure–activity relationships (SARs), it was initially observed that 19 out of the 21 stryrylthiazolidinone derivatives that showed antileishmanial activity (EC_50_ ≤ 30 µM), had a nitro group on the benzene ring of the styryl moiety (Table 3). The pharmacophore appeared to be a 5-styrylthiazolidin-2,4-dione scaffold, substituted at positions *meta* or *para* of the phenyl ring by a nitro group (compounds **4** and **5**). Compounds **12a** and **27e** were the only ones without a nitro group. Compound **27e** showed a moderate selectivity index (SI = 3.9), whereas compound **12a** was more promising (SI = 9.4), due to its low cytotoxicity, indicating a positive contribution of the ethyloxycarbonylmethyl group at position 3 of the thiazolidine-2,4-dione ring.

All the derivatives bearing a nitro group at the *ortho* position of the phenyl ring were moderately active and/or selective. Logically, combining an ethyloxycarbonylmethyl group at position 3 of the thiazolidine-2,4-dione ring and a nitro group at position *meta* of the phenyl ring led to a first hit compound (**12c**) with good efficacy (EC_50_ = 5.51 µM) and selectivity (SI > 22.7). Maintaining the *meta*-nitrostyryl moiety and introducing a cyanomethyl group at position 3 of the thiazolidine-2,4-dione ring led to a second hit compound (**14c**), showing both activity (EC_50_ = 7 µM) and selectivity (SI = 14.4). The *para*-nitro isomers of **12c** and **14c**; **12d** and **14d**, were less active and less selective. Among all other substituents introduced at position 3 of the thiazolidine-2,4-dione ring, none allowed any improvement, neither regarding antileishmanial activity, nor cytotoxicity. Introducing a second nitroaromatic moiety on the pharmacophore (compounds **26b-d**) was not favorable.

Examining the structure of the two hit compounds **12c** and **14c** revealed that **12c** bears an ester function which is usually quite labile in vivo. Consequently, it was crucial to determine whether its carboxy metabolite was active or not. Thus, the ester compound **12c** was subjected to acid hydrolysis to give the corresponding acid **29** (Scheme 5), and this latter was evaluated in vitro (Table 3). Unfortunately, despite being non-cytotoxic (CC_50_ > 250 µM), carboxylic acid **29** was not active on *L. infantum* (EC_50_ > 50 µM), making the ester **12c** a poor candidate for further development. Thus, compound **14c** was regarded as the best molecule in the series. Accordingly, it was assessed for its cytotoxicity on a second human cell line (THP-1) along with its lipophilicity and aqueous solubility, to evaluate its potential for further hit to lead chemistry (Table 4). Compound **14c** was not cytotoxic on the THP-1 cell line (CC_50_ = 121 µM), and presented a measured logD_7.4_ value of 2.85, making it a lipophilic molecule. However, **14c** showed a PBS (pH 7.4) solubility of 16 µM (logS = 4.8). These complementary parameters were in favor of pursuing the development of this chemical series to search for a new derivative with improved potency.

For decades, antiparasitic (more precisely antiprotozoal) properties were reported for many nitroaromatic compounds, including commercial drug compounds such as metronidazole, tinidazole, secnidazole, ornidazole (anti-*T. vaginalis*, anti-*G. intestinalis*, anti-*E. histolytica*), nifurtimox and benznidazole (anti-*T. cruzi*) or nitazoxanide (anti-*C. parvum*). These nitroaromatic compounds were shown to behave as prodrugs which are bioactivated in the parasite cell by nitroreductase enzymes (type 1 and/or 2), leading to lethal cytotoxic metabolites [4,34]. Indeed, nitroaromatics remain attractive chemotypes for the conception of selective and potent novel antiparasitic candidates, such as recently approved fexinidazole (anti-*T. brucei gambiense*), but also to develop novel antibacterial compounds, as recently illustrated with the approval of antitubercular drug compounds delamanid and pretomanid.

To date, two leishmanial nitroreductases have been characterized: an essential type 1 mitochondrial NTR1 (PMID: 23208716) and a cytosolic type 2 NTR2 (PMID: 27812217), that catalyze the 1-electron reduction in nitroaromatics. To determine if NTR-mediated bio-activation played a role in the mechanism of action of compounds in this series, the potency of **14c** was assessed against both wild-type *L. donovani* (LdBOB)and transgenic *L. donovani* overexpressing either NTR1 or NTR2. Indeed, parasites overexpressing these nitroreductases were hyper-sensitive to **14c** compared to wild-type, most notably promastigotes expressing elevated levels of NTR1 (Table 5). This strongly suggests that **14c** is a substrate of *Leishmania* NTRs, and that bioactivation by these enzymes is a key factor in its antileishmanial activity. However, beyond the bioactivation of these nitroaromatic derivatives by parasitic NTRs, it is possible that stryrylthiazolidin-2,4-diones interact with other antileishmanial targets, such as reported in the literature dealing with inhibition of pteridine reductase 1 [31], perhaps explaining the antileishmanial activity noted with non-nitrated compounds **12a** and **27e**.

## 3. Material and Methods

### 3.1. Chemistry

#### 3.1.1. General Methods

The starting materials were purchased from Sigma-Aldrich (Saint Louis, MO, USA). All melting points were determined on a Stuart melting point apparatus SMP3 and are uncorrected. IR spectra were recorded on a Nicolet iS10 FT-IR spectrometer using KBr wafer technique. The ^1^H-NMR spectra were recorded on a Bruker Avance 400 spectrometer operating at 400 MHz (^1^H) or 100 MHz (^13^C). ^1^H- and ^13^C-NMR chemical shifts (δ) were reported in parts per million (ppm) and were referenced to the residual proton peaks in the deuterated solvent DMSO-*d*_6_ (2.50 ppm for ^1^H and 39.70 ppm for ^13^C). Multiplicities are represented by s (singlet), d (doublet), t (triplet), q (quartet), and m (multiplet). Coupling constants (*J*) are reported in Hertz (Hz). Elemental analyses (C, H, N) were carried out using a Perkin Elmer 240 C Micro analyzer at the Microanalytical Laboratory at Assiut University, and the results obtained were found in good agreement with the theoretical values within ±0.4%. All the compounds involved in biological evaluation showed a level of purity above 95%. Thiazolidine-2,4-dione (1) was prepared according to a literature procedure [31].

#### 3.1.2. Physicochemical Determination of Aqueous Solubility and Partition Coefficient

The solubility of **14c** was evaluated by the shake-flask method (*N* = 2). Briefly, an excess of compound (approx. 2 mg) was stirred in PBS (0.5 mL, 0.15 M, pH 7.4) at 25 °C for 72 h. A 100 µL sample was drawn, any excess material removed by centrifugation, and diluted tenfold with methanol/water 1:1 before HPLC-UV quantification using a pre-established calibration curve (PLRP-S column (Agilent), 3 µm particle size, 100 A porosity, 50 × 2.1 mm internal dimensions). Eluent: isocratic acetonitrile/water/formic acid 80:20:0.1, 0.4 mL/min, detection at 320 nm, 2-µL injections).

Lipophilicity was evaluated by the Chromatographic Hydrophobicity Index (CHI) [35] on a Restek Ultra C_18_ column (5 µm particle size, 100 A porosity, 50 × 2.1 mm internal dimensions) using a gradient of A: 10 mM ammonium acetate, adjusted to pH 7.4; B: acetonitrile, at a flow rate of 0.300 mL/min and 0.5-µL injections.

The validity of the CHI versus retention time was established on a reference mixture containing theophylline, phenyltetrazole, benzimidazole, acetophenone, propiophenone, butyrophenone and valerophenone. The correlation between CHI and logD was taken from literature data: logD_7.4_^CHI^ = 0.054 × CHI-1.467 [35].

#### 3.1.3. Organic Synthesis and Product Characterization

##### General Procedure for the Synthesis of the Potassium 5-Arylidenethiazolidin-2,4-dion-3-ides **7–11**

A solution of potassium hydroxide (0.123 g, 2.2 mmol) in ethanol (7 mL) was added to a hot solution of compounds **2–6** (2 mmol) in an ethanol-dioxane mixture (30 mL). The reaction mixture was then heated under stirring for 20 min. The solid products obtained were filtered, washed with ethanol, dried, and crystallized.

*(1) Potassium (Z)-5-benzylidenethiazolidine-2,4-dion-3-ide (**7**).* White solid from ethanol-dioxane, yield (69%) mp 320 °C (charring). IR (ν cm^−1^): 3080 (CH arom.), 3052 (CH arom.), (3016 CH arom.), 1674 (C=O), 1617, 1617, 1548, 1491. ^1^H-NMR (400 MHz, DMSO) δ (ppm): 7.53 (d, *J* = 7.5 Hz, 2H, Ph-H), 7.43 (t, *J* = 7.7 Hz, 2H, Ph-H), 7.34 (s, 1H, -CH=), 7.30 (t, *J* = 7.3 Hz, 1H, Ph-H). ^13^C-NMR (100 MHz, DMSO) δ (ppm): 183.27 (C=O), 176.30 (C=O), 136.45 (C), 136.24 (=CH-), 129.47 (2CH), 129.22 (2CH), 128.32 (C), 122.95 (CH). Anal. Calcd. for C_10_H_6_KNO_2_S (243.32): C, 49.36; H, 2.49; N, 5.76; S, 13.18. Found: C, 49.49; H, 2.40; N, 5.69; S, 13.08%.

*(2) Potassium (Z)-5-(2-nitrobenzylidene)thiazolidine-2,4-dion-3-ide (**8**).* Yellow solid from ethanol-dioxane, yield (87%), mp 220 °C. IR (ν cm^−1^): 1691 (C=O), 1616, 1570, 1556, 1542 1473. ^1^H-NMR (400 MHz, DMSO) δ (ppm): 7.99 (d, *J* = 8.1 Hz, 1H, Ar-H), 7.79 (s, 1H, -CH=), 7.78–7.76 (m, 1H, Ar-H), 7.56–75.2 (m, 1H, Ar-H), 7.44 (s, 1H, Ar-H). ^13^C-NMR (100 MHz, DMSO) δ (ppm): 182.39 (C=O), 175.31 (C=O), 149.20 (C), 142.33 (C), 133.75 (CH), 131.06 (C), 129.36 (CH), 129.11 (CH), 124.93 (CH), 116.27 (CH). Anal. Calcd. for C_10_H_5_KN_2_O_4_S (288.32): C, 41.66; H, 1.75; N, 9.72; S, 11.12. Found: C, 41.53; H, 1.66; N, 9.62; S, 11.33%.

*(3) Potassium (Z)-5-(3-nitrobenzylidene)thiazolidine-2,4-dion-3-ide (**9**).* Yellow solid from ethanol-dioxane, yield (85%), mp 300 °C (charring). IR (ν cm^−1^): 3091 (C-H arom), 3065 (C-H arom.), 2871 (C-H aliph.), 1678 (C=O), 1607, 1545, 1352. ^1^H-NMR (400 MHz, DMSO) δ (ppm): 8.38 (t, *J* = 2.0 Hz, 1H, Ar-H), 8.14 (ddd, *J* = 8.2, 2.3, 0.9 Hz, 1H, Ar-H), 7.97 (d, *J* = 7.8 Hz, 1H, Ar-H), 7.72 (t, *J* = 8.0 Hz, 1H, Ar-H), 7.45 (s, 1H, -CH=). ^13^C-NMR (100 MHz, DMSO) δ (ppm): 182.03 (C=O), 174.57 (C=O), 148.65 (C), 139.40 (=CH-), 138.14 (C), 135.73 (CH), 130.73 (CH), 123.16 (CH), 122.61 (CH), 120.62 (C). Anal. Calcd. for C_10_H_5_KN_2_O_4_S (288.32): C, 41.66; H, 1.75; N, 9.72; S, 11.12. Found: C, 41.55; H, 1.63; N, 9.68; S, 11.16%.

*(4) Potassium (Z)-5-(4-nitrobenzylidene)thiazolidine-2,4-dion-3-ide (**10**).* Yellow solid from ethanol-dioxane, yield (90%), mp 310 °C (charring). IR (ν cm^−1^): 3107 (C-H arom.), 2897 (C-H aliph.), 2864 (C-H aliph.), 1693 (C=O), 1619 1596, 1577, 1528. ^1^H-NMR (400 MHz, DMSO) δ (ppm): 8.27 (d, *J* = 8.8 Hz, 2H, Ar-H), 7.77 (d, *J* = 8.8 Hz, 2H, Ar-H), 7.40 (s, 1H, -CH=). ^13^C-NMR (100 MHz, DMSO) δ (ppm): 182.82 (C=O), 174.94 (C=O), 146.25 (C), 143.34 (=CH-), 142.03 (C), 130.15 (2CH), 124.40 (2CH), 120.14 (C). Anal. Calcd. for C_10_H_5_KN_2_O_4_S (288.32): C, 41.66; H, 1.75; N, 9.72; S, 11.12. Found: C, 41.60; H, 1.71; N, 9.65; S, 11.32%.

##### General Procedure for the Synthesis of 5-Arylidenethiazolidinediones **12b-e–16b-e**

An equimolar mixture (1 mmol) of ethyl chloroacetate (0.123 g, 0.107 mL), and the potassium salts; **7** (0.234 g), **8** (0.288 g), **9** (0.288 g), **10** (0.288 g) or **11** (0.333 g) in DMF (5 mL) was heated under reflux for 6 h. After cooling, the solid products obtained were filtered, washed with water, dried, and recrystallized.

(1) Synthesis of compounds **12b-e**

*(1) Ethyl ((Z)-5-(2-nitrobenzylidene)thiazolidine-2,4-dion-3-yl)acetate (**12b**).* Brownish yellow crystals from ethanol, yield (79%), mp 81–83 °C. IR (ν cm^−1^): 3068 (C-H arom.), 3009 (C-H arom.), 2983 (C-H aliph.), 2937 (C-H aliph) 1741 (C=O), 1694 (C=O), 1607, 1568. 1568. 1524. ^1^H-NMR (400 MHz, DMSO) δ (ppm): 8.25–8.23 (m, 2H, (-CH= + Ar-H), 7.92 (t, *J* = 7.5 Hz, 1H, Ar-H), 7.74–7.78 (m, 2H, Ar-H), 4.52 (s, 2H, CH_2_), 4.20 (q, *J* = 7.2 Hz, 2H, CH_3_CH_2_), 1.23 (t, *J* = 7.2 Hz, 3H, CH_3_CH_2_). ^13^C-NMR (100 MHz, DMSO) δ (ppm): 167.12 (C=O), 167.07 (C=O), 164.66 (C=O), 148.25 (C), 135.17 (-CH=), 131.94 (CH), 131.85 (CH), 129.81 (CH), 129.20 (C), 126.02 (CH), 125.42 (CH), 62.18 (OCH_2_), 42.76 (COCH_2_), 14.40 (CH_3_CH_2_). Anal. Calcd. for C_14_H_12_N_2_O_6_S (336.32): C, 50.00; H, 3.60; N, 8.33; S, 9.53. Found: C, 49.71; H, 3.61; N, 8.28; S, 9.55%.

*(2) Ethyl ((Z)-5-(3-nitrobenzylidene)thiazolidine-2,4-dion-3-yl)acetate (**12c**).* Yellow crystals from ethanol, yield (86%), mp 130–132 °C. IR (ν cm^−1^): 3067 (C-H arom.), 3000 (C-H arom.), 2963 (C-H aliph.), 2939 (C-H aliph.), 1734 (C=O), 1704 (C=O), 1689 (C=O), 1606, 1571, 1533. ^1^H-NMR (400 MHz, DMSO) δ (ppm): 8.53 (t, *J* = 1.9 Hz, 1H, Ar-H), 8.34 (ddd, *J* = 8.3, 2.2, 0.8 Hz, 1H, Ar-H), 8.19 (s, 1H, -CH=), 8.08 (d, *J* = 7.9 Hz, 1H, Ar-H), 7.86 (t, *J* = 8.0 Hz, 1H, Ar-H), 4.53 (s, 2H, -CH_2_-), 4.19 (q, *J* = 7.2 Hz, 2H, CH_2_CH_3_), 1.23 (t, *J* = 7.2 Hz, 3H, CH_2_CH_3_). ^13^C-NMR (100 MHz, DMSO) δ (ppm): 167.06 (C=O), 166.79 (C=O), 165.10 (C=O), 148.76 (C), 135.93 (-CH=), 134.88 (C), 132.37 (CH), 131.47 (CH), 125.41 (CH), 125.34 (CH), 123.97 (C), 62.19 (OCH_2_), 42.84 (COCH_2_), 14.42 (CH_3_CH_2_). Anal. Calcd. for C_14_H_12_N_2_O_6_S (336.32): C, 50.00; H, 3.60; N, 8.33; S, 9.53. Found: C, 49.63; H, 3.51; N, 8.17; S, 9.57%.

*(3) Ethyl ((Z)-5-(4-nitrobenzylidene)thiazolidine-2,4-dion-3-yl)acetate (**12d**).* Yellow platelets from ethanol, yield (88%), mp 136–138 °C. IR (ν cm^−1^): 3111 (C-H arom.), 2995 (C-H aliph.), 1738 (C=O), 1698 (C=O), 1607, 1595, 1512. ^1^H-NMR (400 MHz, DMSO) δ (ppm): 8.35 (d, *J* = 8.8 Hz, 2H, Ar-H), 8.11 (s, 1H, -CH=), 7.91 (d, *J* = 8.8 Hz, 2H, Ar-H), 4.53 (s, 2H, -CH_2_-), 4.19 (q, *J* = 7.2 Hz, 2H, CH_2_CH_3_), 1.22 (t, *J* = 7.2 Hz, 3H, CH_2_CH_3_). ^13^C-NMR (100 MHz, DMSO) δ (ppm): 167.03 (C=O), 166.93 (C=O), 165.07 (C=O), 148.36 (C), 139.38 (-CH=), 132.03 (C), 131.65 (2CH), 125.25 (C), 124.77 (2CH), 62.20 (O-CH_2_), 42.84 (CO-CH_2_), 14.40 (CH_3_). Anal. Calcd. for C_14_H_12_N_2_O_6_S (336.32): C, 50.00; H, 3.60; N, 8.33; S, 9.53. Found: C, 49.88; H, 3.51; N, 8.21; S, 9.40%.

(2) Synthesis of compounds **13a-e**

These compounds were prepared using 1 mmol of chloroacetone (0.093 g, 0.08 mL) following the above procedure described for **12a-e**.

*(1) (Z)-5-Benzylidene-3-(2-oxopropyl)thiazolidine-2,4-dione (**13a**).* Pale-yellow crystals from ethanol, yield (80%), mp 139–140 °C. IR (ν cm^−1^): 3050 (CH arom.), 3017 (CH arom.), 2975 (CH aliph.), 2933 (CH aliph.), 1743 (C=O), 1727 (C=O), 1682 (C=O), 1608, 1597, 1570, 1573. ^1^H-NMR (400 MHz, DMSO) δ (ppm): 7.96 (s, 1H, -CH=), 7.65–7.63 (m, 2H, Ph-H), 7.57–7.49 (m, 3H, Ph-H), 4.68 (s, 2H, CH_2_), 2.26 (s, 3H, CH_3_). ^13^C-NMR (100 MHz, DMSO) δ (ppm): 200.77 (C=O), 167.30 (C=O), 165.67 (C=O), 133.95 (C=O), 133.27 (-CH=), 131.27 (C), 130.67 (2CH), 129.85 (2CH), 121.30 (C), 50.88 (CH_2_), 27.34 (CH_3_). Anal. Calcd. for C_13_H_11_NO_3_S (261.30): C, 59.76; H, 4.24; N, 5.36; S, 12.27. Found: C, 59.56; H, 4.20; N, 5.29; S, 12.21%.

*(2) (Z)-5-(2-Nitrobenzylidene)-3-(2-oxopropyl)thiazolidine-2,4-dione (**13b**).* Shiny-buff crystals from ethanol, yield (79%), mp 146–148 °C. IR (ν cm^−1^): 3102 (CH arom.), 3073 (CH arom.), 2984 (CH aliph.), 2945 (CH aliph.), 2861 (C-H aliph.), 1749 (C=O), 1732 (C=O), 1694 (C=O), 1615, 1603, 1570 and 1528. ^1^H NMR (400 MHz, DMSO) δ (ppm): 8.26–8.23 (m, 1H, Ar-H), 8.21 (s, 1H, -CH=), 7.94–7.90 (m, 1H, Ar-H), 7.76 (m, 2H, Ar-H), 4.70 (s, 2H, CH_2_), 2.27 (s, 3H, CH_3_). ^13^C-NMR (100 MHz, DMSO) δ (ppm): 200.76 (C=O), 167.10 (C=O), 164.80 (C=O), 148.26 (C), 135.16 (-CH=), 131.79 (CH), 131.47 (CH), 129.82 (CH), 129.28 (C), 126.02 (CH), 125.73 (C), 50.85 (CH_2_), 27.38 (CH_3_). Anal. Calcd. for C_13_H_10_N_2_O_5_S (306.29): C, 50.98; H, 3.29; N, 9.15; S, 10.47. Found: C, 50.81; H, 3.23; N, 9.10; S, 10.40%.

*(3) (Z)-5-(3-Nitrobenzylidene)-3-(2-oxopropyl)thiazolidine-2,4-dione (**13c**).* Small lustrous brown crystals from ethanol, yield (88%), mp 188–190 °C. IR (ν cm^−1^): 3073 (CH arom.), 2992 (CH aliph.), 1756 (C=O), 1729 (C=O), 1686 (C=O), 1604, 1570, 1528. ^1^H-NMR (400 MHz, DMSO) δ (ppm): 8.52 (t, *J* = 1.9 Hz, 1H, Ar-H), 8.32 (ddd, *J* = 8.2, 2.2, 0.8 Hz,1H, Ar-H), 8.15 (s, 1H, -CH=), 8.07 (d, *J* = 8.0 Hz, 1H, Ar-H), 7.85 (t, *J* = 8.0 Hz, 1H, Ar-H), 4.71 (s, 2H, -CH_2_-), 2.27 (s, 3H, CH_3_). ^13^C-NMR (100 MHz, DMSO) δ (ppm): 200.73 (C=O), 166.76 (C=O), 165.23 (C=O), 148.76 (C), 135.90 (-CH=), 134.98 (C), 131.92 (CH), 131.45 (CH), 125.28 (CH), 124.29 (C), 50.93 (CH_2_), 27.52 (CH_3_). Anal. Calcd. for C_13_H_10_N_2_O_5_S (306.29): C, 50.98; H, 3.29; N, 9.15; S, 10.47. Found: C, 50.79; H, 3.23; N, 9.11; S, 10.39%.

*(4) (Z)-5-(4-Nitrobenzylidene)-3-(2-oxopropyl)thiazolidine-2,4-dione (**13d**).* Yellow crystals from ethanol-dioxane, yield (95%), mp 207–209 °C. IR (ν cm^−1^): 3014 (CH arom.), 2975 (CH aliph.), 2933 (CH aliph.), 1731 (C=O), 1682 br (C=O), 1608, 1514 and 1407. ^1^H-NMR (400 MHz, DMSO) δ (ppm): 8.36 (d, *J* = 8.8 Hz, 2H, Ar-H), 8.08 (s, 1H, -CH=), 7.91 (d, *J* = 8.8 Hz, 2H, Ar-H), 4.71 (s, 2H, CH_2_), 2.27 (s, 3H, CH_3_). ^13^C-NMR (100 MHz, DMSO) δ (ppm): 200.70 (C=O), 166.82 (C=O), 165.22 (C=O), 148.19 (C), 139.52 (-CH=), 131.60 (C + 2CH), 125.60 (C), 124.78 (2CH), 50.94 (CH_2_), 27.51 (CH_3_). Anal. Calcd. for C_13_H_10_N_2_O_5_S (306.29): C, 50.98; H, 3.29; N, 9.15; S, 10.47. Found: C, 50.91; H, 3.21; N, 9.09; S, 10.43%.

(3) Synthesis of compounds **14a-e**

These compounds were prepared using 1 mmol of 2-chloroacetonitrile (0.076 g, 0.064 mL), following the above procedure described for **12a-e**.

*(1) (Z)-2-(5-Benzylidenethiazolidine-2,4-dion-3-yl)acetonitrile (**14a**).* Brownish-yellow crystals from ethanol, yield (80%), mp 230–232 °C. IR (ν cm^−1^): 3000 (CH arom.), 2958 (CH arom.), 2252 (CN), 1777 (C=O), 1747 (C=O), 1697 (C=O), 1608, 1571, 1493. ^1^H-NMR (400 MHz, DMSO) δ (ppm): 8.00 (s, 1H, -CH=), 7.65–7.63 (m, 2H, Ph-H), 7.58–7.50 (m, 3H, Ph-H), 4.81 (s, 2H, CH_2_). ^13^C-NMR (100 MHz, DMSO) δ (ppm): 166.87 (C=O), 164.70 (C=O), 134.66 (-CH=), 133.17 (C), 131.44 (CH), 130.74 (2CH), 129.91 (2CH), 120.95 (CH), 115.02 (C), 29.26 (CH_2_). Anal. Calcd. for C_12_H_8_N_2_O_2_S (244.27): C, 59.01; H, 3.30; N, 11.47; S, 13.12. Found: C, 59.16; H, 3.27; N, 11.41; S, 13.09%.

*(2) (Z)-2-(5-(2-Nitrobenzylidene)thiazolidine-2,4-dion-3-yl)acetonitrile (**14b**)*. Yellow crystals from ethanol, yield (85%), mp 119–120 °C. IR (ν cm^−1^): 3003 (CH arom.), 2963 (CH arom.), 1746 (C=O), 1694 br (C=O), 1619 (C=O), 1619, 1604, 1571. ^1^H-NMR (400 MHz, DMSO) δ (ppm): 8.26–824 (m, 2H, Ar-H + -CH=), 7.93 (td, *J* = 7.6, 0.9 Hz, 1H, Ar-H), 7.79–7.72 (m, 2H, Ar-H), 4.82 (s, 2H, CH_2_). ^13^C-NMR (100 MHz, DMSO) δ (ppm): 166.73 (C=O), 164.03 (C=O), 148.24 (C), 135.24 (-CH=), 131.94 (CH), 131.90 (CH), 129.75 (CH), 129.22 (C), 126.06 (CH), 125.51 (C), 114.94 (CN), 29.32 (CH_2_). Anal. Calcd. for C_12_H_7_N_3_O_4_S (289.27): C, 49.83; H, 2.44; N, 14.53; S, 11.08. Found: C, 49.70; H, 2.36; N, 14.42; S, 11.12%

*(3) (Z)-2-(5-(3-Nitrobenzylidene)thiazolidine-2,4-dion-3-yl)acetonitrile (**14c**)*. Beige crystals from ethanol-dioxane, yield (95%), mp 173–175 °C. IR (ν cm^−1^): 3084 (CH arom.), 2998 (CH aliph.), 2957 (CH aliph.), 1751 (C=O), 1688_b_ (C=O), 1608, 1570, 1541. ^1^H-NMR (400 MHz, DMSO) δ (ppm): 8.52 (t, *J* = 2.0 Hz, 1H, Ar-H), 8.34 (dt, *J* = 10.9, 4.3 Hz, 1H, Ar-H), 8.20 (s, 1H, -CH=), 8.07 (d, *J* = 7.7 Hz, 1H, Ar-H), 7.86 (t, *J* = 8.0 Hz, 1H, Ar-H), 4.83 (s, 2H, CH_2_). ^13^C-NMR (100 MHz, DMSO) δ (ppm): 166.38 (C=O), 164.45 (C=O), 148.80 (C), 135.88 (-CH=), 134.87 (C), 132.23 (CH), 131.52 (CH), 125.43 (CH), 125.29 (CH), 124.10 (C), 114.93 (CN), 29.40 (CH_2_). Anal. Calcd. for C_12_H_7_N_3_O_4_S (289.27): C, 49.83; H, 2.44; N, 14.53; S, 11.08. Found: C, 49.80; H, 2.35; N, 14.27; S, 11.12%.

*(4) (Z)-2-(5-(4-Nitrobenzylidene)thiazolidine-2,4-dion-3-yl)acetonitrile (**14d**)*. Fine brownish-yellow crystals from ethanol-dioxane, yield (87%), mp 193–195 °C. IR (ν cm^−1^): 3079 (C-H arom.), 3029 (C-H arom.), 2997 (C-H aliph.), 2956 (C-H aliph.), 1753 (C=O), 1698 br (C=O), 1608, 1596 and 1530. ^1^H-NMR (400 MHz, DMSO) δ (ppm): 8.35 (d, *J* = 8.8 Hz, 2H, Ar-H), 8.11 (s, 1H, -CH=), 7.89 (d, *J* = 8.8 Hz, 2H, Ar-H), 4.83 (s, 2H, CH_2_). ^13^C-NMR (100 MHz, DMSO) δ (ppm): 166.43 (C=O), 164.43 (C=O), 148.22 (C), 139.37 (C), 131.88 (-CH=), 131.61 (2CH), 125.38 (C), 124.80 (2CH), 114.87 (CH), 29.40 (CH_2_). Anal. Calcd. for C_12_H_7_N_3_O_4_S (289.27): C, 49.83; H, 2.44; N, 14.53; S, 11.08. Found: C, 49.70; H, 2.39; N, 14.50; S, 11.20%.

(4) Synthesis of compounds **15a-e**

These compounds were prepared using 2-chloroacetamide (1 mmol, 0.093 g), following the procedure described for **12a-e**.

*(1) (Z)-2-(5-Benzylidenethiazolidine-2,4-dion-3-yl)acetamide (**15a**)*. Yellow crystals from ethanol, yield (76%), mp 229–231 °C. IR (ν cm^−1^): 3365, 3180 (NH_2_), 3056 (C-H arom.), 2942 (C-H aliph.), 1739 (C=O), 1693 (C=O), 1667 (C=O), 1607, 1573, 1492, 1448.^1^H-NMR (400 MHz, DMSO) δ (ppm): 7.97 (s, 1H, -CH=), 7.75 (s, 1H, NH), 7.66–7.65 (m, 2H, Ph-H), 7.58–7.51 (m, 3H, Ph-H), 7.34 (s, 1H, NH), 4.25 (s, 2H, CH_2_) ^13^C-NMR (100 MHz, DMSO) δ (ppm): 167.58 (C=O), 167.30 (C=O), 165.79 (C=O), 133.72 (-CH=), 133.38 (C), 131.20 (C), 130.62 (2CH), 129.88 (2CH), 121.70 (C), 43.80 (CH_2_). Anal. calcd. For C_12_H_10_N_2_O_3_S (262.28): C, 54.95; H, 3.84; N, 10.68; S, 12.22. Found: C, 54.87; H, 3.78; N, 10.60; S, 12.19%

*(2) (Z)-2-(5-(2-Nitrobenzylidene)thiazolidine-2,4-dion-3-yl)acetamide (**15b**)*. Yellow crystals from ethanol, yield (89%), mp 233–235 °C. IR (ν cm^−1^): 3441 (N-H), 3315 (N-H), 3181 (N-H), 1747 (C=O), 1678_b_ (C=O), 1607, 1525 and 1474. ^1^H-NMR (400 MHz, DMSO) δ (ppm): 8.25–8.23 (m, 1H, Ar-H), 8.19 (s, 1H, -CH=), 7.92 (t, *J* = 7.5 Hz, 1H, Ar-H), 7.78–7.75 (m, 3H, Ar-H + NH), 7.36 (s, 1H, NH), 4.26 (s, 2H, CH_2_).^13^C-NMR (100 MHz, DMSO) δ (ppm): 167.35 (C=O), 167.22 (C=O), 165.05 (C=O), 148.30 (C), 135.17 (-CH=), 131.74 (CH), 130.93 (CH), 129.77 (CH), 129.32 (C), 126.08 (C), 126.05 (CH), 43.87 (CH_2_). Anal. calcd. for C_12_H_9_N_3_O_5_S (307.28): C, 46.91; H, 2.95; N, 13.68; S, 10.43. Found: C, 46.83; H, 2.90; N, 13.61; S, 10.32%.

*(3) (Z)-2-(5-(3-Nitrobenzylidene)thiazolidine-2,4-dion-3-yl)acetamide (**15c**)*. Yellow crystals from ethanol-dioxane, yield (87%), mp 256–258 °C. IR (ν cm^−1^): 3441 (N-H), 3316 (N-H), 3190 (N-H), 3079 (C-H arom), 1747 (C=O), 1678_b_ (C=O),1607, 1525 and 1444. ^1^H NMR (400 MHz, DMSO) δ (ppm): 8.52 (s, 1H, Ar-H), 8.32 (dd, *J* = 7.1, 1.1 Hz, 1H, Ar-H), 8.14 (s, 1H, -CH=), 8.07 (d, *J* = 7.9 Hz, 1H, Ar-H), 7.85 (t, *J* = 8.0 Hz, 1H, Ar-H), 7.76 (s, 1H, NH), 7.36 (s, 1H, NH), 4.26 (s, 2H, CH_2_).^13^C-NMR (100 MHz, DMSO) δ (ppm): 167.18 (C=O), 167.10 (C=O), 165.48 (C=O), 148.77 (C), 135.86 (-CH=), 135.06 (C), 131.46 (2CH), 125.23 (CH), 125.18 (CH), 124.67 (C), 43.95 (CH_2_). Anal. calcd. for C_12_H_9_N_3_O_5_S (307.28): C, 46.91; H, 2.95; N, 13.68; S, 10.43. Found: C, 46.85; H, 2.87; N, 13.59; S, 10.39%.

*(4) (Z)-2-(5-(4-Nitrobenzylidene)thiazolidine-2,4-dion-3-yl)acetamide* (***15d***). Small shiny yellow crystals from ethanol-dioxane, yield (94%), mp 281–283 °C. IR (ν cm^−1^): 3432, 3325, 3199 (NH_2_), 3026 (CH arom.), 2981 (CH aliph.), 2942 (CH aliph.), 1747 (C=O), 1683 br. (C=O), 1694 (C=O), 1607, 1509, 1442. ^1^H-NMR (400 MHz, DMSO) δ (ppm): 8.36 (d, *J* = 8.8 Hz, 2H, Ar-H), 8.07 (s, 1H, -CH=), 7.91 (d, *J* = 8.8 Hz, 2H, Ar-H), 7.76 (s, 1H, NH), 7.36 (s, 1H, NH), 4.26 (s, 2H, CH_2_). ^13^C-NMR (100 MHz, DMSO) δ (ppm): 167.17 (C=O), 167.09 (C=O), 165.47 (C=O), 148.14 (C), 139.62 (=CH-), 131.54 (C), 131.16 (C), 126.00 (C), 124.80 (2CH), 43.97 (CH_2_). Anal. calcd. for C_12_H_9_N_3_O_5_S (307.28): C, 46.91; H, 2.95; N, 13.68; S, 10.43. Found: C, 46.97; H, 2.88; N, 13.60; S, 10.36%.

(5) Synthesis of compounds **16b-e**

These compounds were prepared using phenacyl bromide (1 mmol, 0.199 g), following the procedure described for **12b-e**.

*(1) (Z)-5-(2-Nitrobenzylidene)-3-(2-oxo-2-phenylethyl)thiazolidine-2,4-dione (**16b**).* Yellow crystals from ethanol-dioxane, yield (82%), mp 156–158 °C. IR (ν cm^−1^): 3058 (C-H arom), 2937 (C-H arom.), 2977 (C-H aliph.), 2973 (C-H aliph.), 1746 (C=O), 1693 (C=O), 1682 (C=O), 16,204, 1595, 1570. ^1^H-NMR (400 MHz, DMSO) δ (ppm): 8.27–8.25 (m, 2H, =CH- + Ar-H), 8.12–8.10 (m, 2H, Ph-H), 7.93 (t, *J* = 7.5 Hz, 1H, Ar-H), 7.81–7.74 (m, 3H, Ph-H), 7.62 (t, *J* = 7.7 Hz, 2H, Ar-H), 5.37 (s, 2H, CH_2_). ^13^C-NMR (100 MHz, DMSO) δ (ppm): 191.71 (C=O), 167.27 (C=O), 164.97 (C=O), 148.29 (C), 135.19 (-CH=), 134.99 (C), 134.21 (C), 131.82 (CH), 131.73 (C), 129.85 (CH), 129.52 (2CH), 129.29 (CH), 128.82 (2CH), 126.05 (CH), 125.69 (C), 48.46 (CH_2_). Anal. calcd. for C_18_H_12_N_2_O_5_S (368.36): C, 58.69; H, 3.28; N, 7.60; S, 8.70. Found: C, 58.60; H, 3.17; N, 7.54; S, 8.66%.

*(2) (Z)-5-(3-Nitrobenzylidene)-3-(2-oxo-2-phenylethyl)thiazolidine-2,4-dione (**16c**).* Yellow crystals from ethanol-dioxane, yield (85%), mp 160–162 °C. IR (ν cm^−1^): 3081 (C-H arom), 3067 (C-H arom.), 2934 (C-H aliph.), 1749 (C=O), 1708 (C=O), 1683 (C=O), 1599, 1535, 1449. ^1^H-NMR (400 MHz, DMSO) δ (ppm): 8.53 (t, *J* = 1.9 Hz, 1H, Ar-H), 8.33 (dd, *J* = 8.2, 2.1 Hz, 1H, Ar-H), 8.18 (s, 1H, -CH=), 8.11–8.08 (m, 3H, Ph-H), 7.86 (t, *J* = 8.0 Hz, 1H, Ar-H), 7.76 (t, *J* = 7.0 Hz, 1H Ar-H), 7.61 (t, *J* = 7.7 Hz, 2H, Ph-H), 5.37 (s, 2H, CH_2_). ^13^C-NMR (400 MHz, DMSO) δ (ppm): 191.66 (C=O), 166.91 (C=O), 165.38 (C=O), 148.74 (C), 135.93 (-CH=), 134.99 (C), 134.95 (C), 134.19 (CH), 132.14 (CH), 131.45 (CH), 129.52 (2CH), 128.82 (2CH), 125.34 (CH), 125.29 (CH), 124.23 (C), 48.54 (CH_2_). Anal. calcd. for C_18_H_12_N_2_O_5_S (368.36): C, 58.69; H, 3.28; N, 7.60; S, 8.70. Found: C, 58.75; H, 3.22; N, 7.51; S, 8.65%.

*(3) (Z)-5-(4-Nitrobenzylidene)-3-(2-oxo-2-phenylethyl)thiazolidine-2,4-dione (**16d**).* Brown crystals from ethanol-dioxane, yield (83%), mp 232–233 °C. IR (ν cm^−1^): 3073 (C-H arom), 3013 (C-H arom.), 2969 (C-H aliph.), 2933 (C-H aliph.), 1755 (C=O), 1697 (C=O), 1682 (C=O), 1607, 1595, 1517. ^1^H-NMR (400 MHz, DMSO) δ (ppm): 8.38 (d, *J* = 8.8 Hz, 2H, Ar-H), 8.14 (s, 1H, -CH=), 8.11 (m, 2H, Ph-H), 7.95 (d, *J* = 8.8 Hz, 2H, Ar-H), 7.76 (t, *J* = 7.4 Hz, 1H, Ph-H), 7.62 (t, *J* = 7.8 Hz, 2H, Ph-H), 5.37 (s, 2H, CH_2_). ^13^C-NMR (100 MHz, DMSO) δ (ppm): 191.68 (C=O), 167.02 (C=O), 165.40 (C), 148.25 (CH), 139.54 (C), 135.02 (C), 134.19 (-CH=), 131.87 (CH), 131.66 (2CH), 129.54 (2CH), 128.84 (2CH), 125.59 (C), 124.82 (2CH), 48.58 (CH_2_). Anal. calcd. for C_18_H_12_N_2_O_5_S (368.36): C, 58.69; H, 3.28; N, 7.60; S, 8.70: Found: C, 58.59; H, 3.28; N, 7.60; S, 8.70%.

*(4) (Z)-3-(2-Oxo-2-phenylethyl)-5-(3,4,5-trimethoxybenzylidene)thiazolidine-2,4-dione (**16e**).* Brownish-yellow crystals from DMF-H_2_O, yield (62%), mp 187–189 °C. IR (ν cm^−1^): 3014 (C-H arom), 2969 (C-H aliph.), 2837 (C-H aliph.), 1740 (C=O), 1686_b_ (C=O), 1605, 1577, 1504. ^1^H-NMR (400 MHz, DMSO) δ (ppm): 8.14 (m, 2H, Ph-H), 8.02 (s, 1H, =CH-), 7.81 (t, *J* = 8.0 Hz, 1H, Ph-H), 7.66 (t, *J* = 7.7 Hz, 2H, Ph-H), 7.05 (s, 2H, Ar-H), 5.39 (s, 2H, CH_2_), 3.91 (s, 6H, 2OCH_3_), 3.81 (s, 3H, OCH_3_). ^13^C-NMR (100 MHz, DMSO) δ 191.80 (C=O), 167.49 (C=O), 165.65 (C=O), 153.74 (2C), 140.24 (CH), 134.97 (C), 134.63 (=CH-), 134.24 (C), 129.53 (2CH), 128.78 (2CH), 120.24 (C), 108.25 (2CH), 60.71 (OCH_3_), 56.53 (2OCH_3_), 55.37 (CH_2_). Anal. calcd. for C_21_H_19_NO_6_S (413.44): C, 61.01; H, 4.63; N, 3.39; S, 7.75. Found C, 61.30; H, 4.54; N, 3.35; S, 7.82%.

##### General Procedure for the Synthesis of Thiazolidine-2,4-dione Derivatives **23b-e–28b-e**

(1) Synthesis of compounds **23b-e**

A mixture of equimolar amounts of 2-chloro-*N*-phenylacetamide (**17**) (1 mmol, 0.169 g) and the potassium salt of **7** (0.234 g), **8** (0.288 g), **9** (0.288 g), **10** (0.288 g) or **11** (0.333 g) in DMF (5 mL) was heated under reflux for 6 hr. After cooling, the solid products obtained were filtered, washed with water, dried, and recrystallized.

*(1) (Z)-2-(5-(2-Nitrobenzylidene)thiazolidine-2,4-dion-3-yl)-N-phenylacetamide (**23b**).* Yellow crystals from ethanol, yield (85%), mp 186–188 °C. IR (ν cm^−1^): 3359 (N-H), 3071 (C-H arom.) 2977 (C-H aliph.), 2938 (C-H aliph.),1748 (C=O), 1697 br (C=O), 1617, 1602, 1540, 1498. ^1^H-NMR (400 MHz, DMSO) δ (ppm): 10.42 (s, 1H, NH), 8.26 (s, 1H, Ar-H), 8.24 (s, 1H, -CH=), 7.95–7.91 (m, 1H, Ar-H), 7.79–7.75 (m, 2H, Ar-H), 7.57 (d, *J* = 7.7 Hz, 2H, Ph-H), 7.34 (t, *J* = 7.9 Hz, 2H, Ph-H), 7.09 (t, *J* = 7.4 Hz, 1H, Ph-H), 4.54 (s, 2H, CH_2_). ^13^C-NMR (100 MHz, DMSO) δ (ppm): 167.37 (C=O), 165.03 (C=O), 164.16 (C=O), 148.30 (C), 138.85 (C), 135.20 (-CH=), 131.80 (C), 131.43 (C), 129.82 (CH), 129.37 (2CH), 129.31 (CH), 126.04 (CH), 125.82 (CH), 124.21 (CH), 119.63 (2CH), 44.60 (CH_2_). Anal. calcd. for: C_18_H_13_N_3_O_5_S (383.38): C, 56.39; H, 3.42; N, 10.96; S, 8.36. Found: C, 56.45; H, 3.35; N, 10.89; S, 8.48%.

*(2) (Z)-2-(5-(3-Nitrobenzylidene)thiazolidine-2,4-dion-3-yl)-N-phenylacetamide (**23c**)*. Yellow crystals from ethanol-dioxane, yield 90%, mp 255–257 °C. IR (ν cm^−1^): 3414 (N-H), 3067 (CH arom.), 2934 (CH aliph.), 1749 (C=O), 1683_br_ (C=O), 1599, 1570, 1484. ^1^H-NMR (400 MHz, DMSO) δ (ppm): 10.41 (s, 1H, NH), 8.53 (t, *J* = 1.9 Hz, 1H, Ar-H), 8.33 (dd, *J* = 8.3, 2.2 Hz, 1H, Ar-H), 8.18 (s, 1H, -CH=), 8.07 (d, *J* = 7.9 Hz, 1H, Ar-H), 7.86 (t, *J* = 8.0 Hz, 1H, Ar-H), 7.57 (d, *J* = 8.3 Hz, 2H, Ph-H), 7.33 (m, 2H, Ph-H), 7.09 (t, *J* = 7.4 Hz, 1H, Ph-H), 4.55 (s, 2H, CH_2_). ^13^C-NMR (100 MHz, DMSO) δ (ppm): 167.02 (C=O), 165.46 (C=O), 164.12 (C=O), 148.77 (CH), 138.84 (C), 135.90 (-CH=), 135.00 (C), 131.84 (CH), 131.46 (CH), 129.36 (2CH), 125.30 (C),125.25 (CH), 124.40 (CH) 124.21 (C), 119.63 (2CH), 44.66 (CH_2_). Anal. calcd. for C_18_H_13_N_3_O_5_S (383.38): C, 56.39; H, 3.42; N, 10.96; S, 8.36. Found: C, 56.42; H, 3.37; N, 11.00; S, 8.41%.

*(3) (Z)-2-(5-(4-Nitrobenzylidene)thiazolidine-2,4-dion-3-yl)-N-phenylacetamide (**23d**).* Yellow crystals from DMF-H_2_O, yield (75%), mp 259–261 °C. IR (ν cm^−1^): 3393 (N-H), 3075 (C-H arom.), 2984 (C-H aliph.), 1753 (C=O), 1697 (C=O), 1682 (C=O), 1609, 1541, 1514. ^1^H NMR (400 MHz, DMSO) δ (ppm): 10.41 (s, 1H, NH), 8.37 (d, *J* = 8.8 Hz, 2H, Ar-H), 8.12 (s, 1H, -CH=), 7.93 (d, *J* = 8.8 Hz, 2H, Ar-H), 7.56 (d, *J* = 7.6 Hz, 2H, Ph-H), 7.33 (t, *J* = 7.9 Hz, 2H, Ph-H), 7.09 (t, *J* = 7.4 Hz, 1H, Ph-H), 4.55 (s, 2H, CH_2_). ^13^C-NMR (100 MHz, DMSO) δ (ppm): 167.10 (C=O), 165.46 (C=O), 164.10 (C=O), 148.20 (C), 139.56 (C), 138.82 (-CH=), 131.61 (2CH), 131.56 (C), 129.37 (2CH), 125.74 (C), 124.81 (2CH), 124.22 (CH), 119.64 (2CH), 44.68 (CH_2_). Anal. calcd. for: C_18_H_13_N_3_O_5_S (383.38): C, 56.39; H, 3.42; N, 10.96; S, 8.36. Found: C, 56.46; H, 3.50; N, 11.01; S, 8.45%.

*(4) (Z)-N-Phenyl-2-(5-(3,4,5-trimethoxybenzylidene)thiazolidine-2,4-dion-3-yl) acetamide (**23e**)*. Pale yellow crystals from DMF-H_2_O, yield (76%), mp 226–228 °C. IR (ν cm^−1^): 3264 (N-H), 3147 (C-H arom.), 2992 (C-H aliph.) 2933 (C-H aliph.), 2836 (C-H aliph.),1741 (C=O), 1686 (C=O), 1670 (C=O), 1606, 1579, 1551. ^1^H-NMR (400 MHz, DMSO) δ (ppm): 10.40 (s, 1H, NH), 7.95 (s, 1H, -CH=), 7.57 (d, *J* = 7.7 Hz, 2H, Ph-H), 7.33 (t, *J* = 7.9 Hz, 2H, Ph-H), 7.09 (t, *J* = 7.4 Hz, 1H, Ph-H), 6.99 (s, 2H, Ar-H), 4.53 (s, 2H, CH_2_), 3.86 (s, 6H, 2 OCH_3_), 3.75 (s, 3H, OCH_3_). ^13^C-NMR (100 MHz, DMSO) δ (ppm): 167.57 (C=O), 165.73 (C=O), 164.25 (C=O), 153.73 (2C), 140.19 (C), 138.88 (C), 134.31 (-CH=), 129.36 (2CH), 128.82 (C), 124.17 (CH), 120.39 (C), 119.62 (2 CH), 108.21 (2CH), 60.70 (OCH_3_), 56.52 (2OCH_3_), 44.48 (CH_2_). Anal. calcd. for: C_21_H_20_N_2_O_6_S (428.46): C, 58.87; H, 4.71; N, 6.54; S, 7.48. Found: C, 59.01; H, 4.79; N, 6.63; S, 7.46%.

(2) Synthesis of compounds **24a-e**.

These compounds were synthesized using 2-chloro-*N*-(4-methylphenyl)acetamide (**18**) (0.184 g, 1 mmol)**,** following the procedure described for **23a-e**.

*(1) (Z)-2-(5-Benzylidenethiazolidine-2,4-dion-3-yl)-N-(4-methylphenyl) acetamide (**24a**).* Shiny white crystals from ethanol-dioxane, yield (88%), mp 277–279 °C. IR (ν cm^−1^): 3266 (N-H), 3126 (C-H arom.), 3059 (C-H arom.), 2922 (C-H aliph.), 1745 (C=O), 1695 (C=O), 1659 (C=O), 1608, 1596, 1573. ^1^H-NMR (400 MHz, DMSO) δ (ppm): 10.31 (s, 1H, NH), 8.00 (s, 1H, -CH=), 7.68–7.66 (m, 2H, Ph-H), 7.60–7.51 (m, 3H, Ph-H), 7.45 (d, *J* = 8.4 Hz, 2H, Ph-H), 7.13 (d, *J* = 8.4 Hz, 2H, Ph-H), 4.46 (s, 2H, CH_2_), 2.26 (s, 3H, CH_3_).^13^C-NMR (100 MHz, DMSO) δ (ppm): 167.60 (C=O), 165.79 (C=O), 163.97 (C=O), 136.37 (C), 134.05 (-CH=), 133.34 (C), 133.13 (C), 131.29 (C), 130.68 (2CH), 129.81 (2CH), 129.72 (2CH), (121.49) (C), 119.64 (2CH), 44.49 (CH_2_), 20.90 (CH_3_). Anal. Calcd. For C_19_H_16_N_2_O_3_S (352.41): C, 64.76; H, 4.58; N, 7.95; S, 9.10. Found: C, 64.82; H, 4.49; N, 7.87; S, 9.14%.

*(2) (Z)-N-(4-Methylphenyl)-2-(5-(2-nitrobenzylidene)thiazolidine-2,4-dion-3-yl) acetamide (**24b**)*. Yellow crystals from ethanol, yield (83%), mp 181–183 °C. IR (ν cm^−1^): 3260 (N-H), 3196 (C-H arom.), 3128 (C-H arom.), 3077 (C-H arom.) 2991 (C-H aliph.), 1769 (C=O), 1698 (C=O), 1666 (C=O), 1607, 1551, 1523. ^1^H-NMR (400 MHz, DMSO) δ (ppm): 10.33 (s, 1H, NH), 8.25 (m, 2H, Ar-H + -CH=), 7.93 (t, *J* = 7.5 Hz, 1H, Ar-H), 7.77 (t, *J* = 7.7 Hz, 2H, Ar-H), 7.45 (d, *J* = 8.4 Hz, 2H, Ar-H), 7.14 (d, *J* = 8.4 Hz, 2H, Ar-H), 4.52 (s, 2H, CH_2_), 2.26 (s, 3H, CH_3_). ^13^C-NMR (100 MHz, DMSO) δ (ppm): 167.36 (C=O), 165.04 (C=O), 163.89 (C=O), 148.30 (C), 136.35 (C), 135.18 (CH), 133.17 (C), 131.79 (C), 131.36 (CH), 129.80 (CH), 129.73 (2CH), 129.30 (CH), 126.04 (CH), 125.94 (C), 119.64 (2CH), 44.56 (CH_2_), 20.90 (CH_3_). Anal. calcd. for C_19_H_15_N_3_O_5_S (397.41): C, 57.42; H, 3.80; N, 10.57; S, 8.07. Found: C, 57.52; H, 3.89; N, 10.63; S, 8.17%.

*(3) (Z)-N-(4-Methylphenyl)2-(5-(3-nitrobenzylidene)thiazolidine-2,4-dion-3-yl) acetamide (**24c**).* Yellow crystals from ethanol-dioxane, yield (82%), mp 276–278 °C. IR (ν cm^−1^): 3264 (N-H), 3071 (CH arom.), 2948 (CH aliph.), 2835 (CH aliph.), 1746 (C=O), 1681 (C=O), 1664 (C=O), 1603, 1570. ^1^H-NMR (400 MHz, DMSO) δ (ppm): 10.32 (s, 1H, NH), 8.52 (s, 1H, Ar-H), 8.32 (dd, *J* = 8.2, 1.6 Hz, 1H, Ar-H), 8.16 (s, 1H –CH=), 8.07 (d, *J* = 7.9 Hz, 1H, Ar-H), 7.85 (t, *J* = 8.0 Hz, 1H, Ar-H), 7.44 (d, *J* = 8.4 Hz, 2H, Ar-H), 7.13 (d, *J* = 8.4 Hz, 2H, Ar-H), 4.53 (s, 2H, CH_2_), 2.26 (s, 3H, CH_3_). ^13^C-NMR (100 MHz, DMSO) δ (ppm) 167.02 (C=O), 165.46 (C=O), 163.85 (C=O), 148.75 (C), 136.33 (C), 135.89 (–CH=), 134.99 (C), 133.17 (C), 131.80 (CH), 131.45 (CH), 129.72 (2CH), 125.38 (C) 125.23 (CH), 124.41 (CH), 119.65 (2CH), 44.61 (CH_2_), 20.90 (CH_3_). Anal. calcd. for: C_19_H_15_N_3_O_5_S (397.41): C, 57.42; H, 3.80; N, 10.57; S, 8.07. Found: C, 57.50; H, 3.88; N, 11.06; S, 8.09%.

*(4) (Z)-N-(4-Methylphenyl)2-(5-(4-nitrobenzylidene)thiazolidine-2,4-dion-3-yl) acetamide (**24d**)*. Yellow crystals from DMF-H_2_O, yield (80%), mp 250–252 °C. IR (ν cm^−1^): 3282 (N-H), 3036 (C-H arom.), 2919 (C-H aliph.), 1750 (C=O), 1704 (C=O), 1676 (C=O), 1611, 1596, 1541. ^1^H-NMR (400 MHz, DMSO) δ (ppm) 10.32 (s, 1H, NH), 8.36 (d, *J* = 8.8 Hz, 2H, Ar-H), 8.11 (s, 1H, -CH=), 7.92 (d, *J* = 8.8 Hz, 2H, Ar-H), 7.44 (d, *J* = 8.4 Hz, 2H, Ar-H), 7.13 (d, *J* = 8.4 Hz, 2H, Ar-H), 4.53 (s, 2H, CH_2_), 2.26 (s, 3H, CH_3_). ^13^C-NMR (100 MHz, DMSO) δ (ppm) 167.10 (C=O), 165.47 (C=O), 163.83 (C=O), 148.18 (C), 139.56 (C), 136.32 (-CH=), 133.18 (C), 131.60 (2CH), 131.51 (C), 129.72 (2CH), 125.76 (C), 124.80 (2CH), 119.65 (2CH), 44.45 (CH_2_). 20.89 (CH_3_). Anal. calcd. for: C_19_H_15_N_3_O_5_S (397.41): C, 57.42; H, 3.80; N, 10.57; S, 8.07. Found: C, 57.49; H, 3.90; N, 11.06; S, 8.03%.

*(5) (Z)-N-(4-Methylphenyl)-2-(5-(3,4,5-trimethoxybenzylidene)thiazolidine-2,4-dion-3-yl)acetamide (**24e**)*. Yellow crystals from DMF-H_2_O, yield (81%), mp 196–198 °C. IR (ν cm^−1^): 3251 (N-H), 3028 (C-H arom.), 2989 (C-H aliph.) 2934 (C-H aliph.), 2836 (C-H aliph.), 1741 (C=O), 1686 (C=O), 1666 (C=O), 1606, 1579, 1546. H-NMR (400 MHz, DMSO) δ (ppm): 10.31 (s, 1H, NH), 7.94 (s, 1H, -CH=), 7.45 (d, *J* = 8.4 Hz, 2H, Ar-H), 7.13 (d, *J* = 8.4 Hz, 2H, Ar-H), 6.98 (s, 2H, Ar-H), 4.51 (s, 2H, CH_2_), 3.85 (s, 6H, 2OCH_3_), 3.75 (s, 3H, OCH_3_), 2.26 (s, 3H, CH_3_). ^13^C-NMR (100 MHz, DMSO) δ (ppm): 167.57 (C=O), 165.73 (C=O), 163.98 (C=O), 153.73 (2C), 140.18 (C), 136.38 (C), 134.28 (-CH=), 133.13 (C), 129.71 (2CH), 128.82 (C), 120.39 (C), 119.63 (2CH), 108.20 (2CH), 60.69 (CH_2_), 56.51 (OCH_3_), 44.44 (2 OCH_3_), 20.89 (CH_3_). Anal. calcd. for: C_22_H_22_N_2_O_6_S (442.49): C, 59.72; H, 5.01; N, 6.33; S, 7.25. Found: C, 57.42; H, 3.80; N, 10.57; S, 8.07%.

(3) Synthesis of compounds **25b-e**.

These compounds were synthesized using 2-chloro-*N*-(4-methoxylphenyl)acetamide **19** (1 mmol, 0.2 g), following the procedure described for **23a-e**.

*(1) (Z)-2-(5-(2-Nitrobenzylidene)thiazolidine-2,4-dion-3-yl)-N-(4-methoxyphenyl) acetamide (**25b**).* Brown crystals from ethanol-dioxane, yield (86%), mp 204–205 °C. IR (ν cm^−1^): 3285 (N-H), 3193 (C-H arom.), 3139 (C-H arom.), 3080 (C-H arom.) 2932 (C-H aliph.), 2835 (C-H aliph.), 1750 (C=O), 1699 (C=O), 1662 (C=O), 1610, 1551, 1520.^1^H-NMR (400 MHz, DMSO) δ (ppm): 10.27 (s, 1H, NH), 8.25 (d, *J* = 8.3 Hz, 1H, Ar-H), 8.23 (s, 1H, -CH=), 7.93 (t, *J* = 7.6 Hz, 1H, Ar-H), 7.77 (t, *J* = 7.6 Hz, 2H, Ar-H), 7.48 (d, *J* = 9.0 Hz, 2H, Ar-H), 6.91 (d, *J* = 9.0 Hz, 2H, Ar-H), 4.51 (s, 2H, CH_2_), 3.73 (s, 3H, OCH_3_). ^13^C-NMR (100 MHz, DMSO) δ (ppm): 167.37 (C=O), 165.04 (C=O), 163.63 (C=O), 155.97 (C), 148.30 (C), 135.19 (CH), 131.97 (C), 131.79 (C), 131. 35 (CH), 129.81 (CH), 129.31 (CH), 126.04 (CH), 125.95 (C), 121.19 (2CH), 114.46 (2CH), 55.63 (OCH_3_), 44.49 (CH_2_). Anal. calcd. for: C_19_H_15_N_3_O_6_S (413.40) C, 55.20; H, 3.66; N, 10.16; S, 7.76. Found: C, 55.27; H, 3.69; N, 10.22; S, 7.78%.

*(2) (Z)-2-(5-(3-Nitrobenzylidene)thiazolidine-2,4-dion-3-yl)-N-(4-methoxyphenyl) acetamide (**25c**).* Beige crystals from ethanol-dioxane, yield (84%), mp 252–254 °C. IR (ν cm^−1^): 3258 (N-H), 3069 (CH arom.), 2948 (CH aliph.), 2835 (CH aliph.), 1746 (C=O), 1682 (C=O), 1659 (C=O), 1603, 1533. ^1^H-NMR (400 MHz, DMSO) δ (ppm): 10.26 (s, 1H, NH), 8.54 (t, *J* = 1.7 Hz, 1H, Ar-H), 8.34 (dd, *J* = 8.2, 2.2 Hz, 1H, Ar-H), 8.18 (s, 1H, -CH=), 8.09 (d, *J* = 7.8 Hz, 1H, Ar-H), 7.86 (t, *J* = 8.0 Hz, 1H, Ar-H), 7.47 (d, *J* = 9.1 Hz, 2H, Ar-H), 6.90 (d, *J* = 9.1 Hz, 2H, Ar-H), 4.51 (s, 2H, CH_2_), 3.73 (s, 3H, OCH_3_). ^13^C-NMR (100 MHz, DMSO) δ (ppm): 167.04 (C=O), 165.48 (C=O), 163.60 (C=O), 155.97 (C), 148.78 (C), 135.89 (-CH=), 135.02 (C), 131.96 (C), 131.79 (CH), 131.47 (CH), 125.30 (CH), 125.25 (CH), 124.45 (C), 121.19 (2CH), 114.46 (2CH), 55.63 (OCH_3_), 44.56 (CH_2_). Anal. calcd. for: C_19_H_15_N_3_O_6_S (413.40) C, 55.20; H, 3.66; N, 10.16; S, 7.76. Found: C, 55.32; H, 3.57; N, 10.12; S, 7.71%.

*(3) (Z)-N-(4-Methoxyphenyl)-2-(5-(4-nitrobenzylidene)thiazolidine-2,4-dion-3-yl)acetamide (**25d**).* Yellow crystals from DMF-H_2_O, yield (83%), mp 254–256 °C. IR (ν cm^−1^): 3349 (N-H), 3073 (C-H arom.), 2937 (C-H aliph.), 1751 (C=O), 1690 _br_ (C=O), 1611, 1593, 1512. ^1^H- NMR (400 MHz, DMSO) δ (ppm): 10.26 (s, 1H, NH), 8.36 (d, *J* = 8.8 Hz, 2H, Ar-H), 8.11 (s, 1H, -CH=), 7.92 (d, *J* = 8.8 Hz, 2H, Ar-H), 7.47 (d, *J* = 9.1 Hz, 2H, Ar-H), 6.90 (d, *J* = 9.1 Hz, 2H, Ar-H), 4.52 (s, 2H, CH_2_), 3.72 (s, 3H, OCH_3_). ^13^C-NMR (100 MHz, DMSO) δ (ppm): 167.10 (C=O), 165.47 (C=O), 163.57 (C=O), 155.98 (C), 148.18 (C), 139.56 (C), 131.95 (2CH), 131.59 (CH), 131.49 (C), 125.77 (C), 124.79 (2CH), 121.20 (2CH), 114.45 (2CH), 55.62 (OCH_3_), 44.57 (CH_2_). Anal. calcd. for: C_19_H_15_N_3_O_6_S (413.40) C, 55.20; H, 3.66; N, 10.16; S, 7.76. Found: C, 55.29; H, 3.59; N, 10.00; S, 7.77%.

*(4) (Z)-N-(4-Methoxyphenyl)-2-(5-(3,4,5-trimethoxybenzylidene)thiazolidine-2,4-dion-3-yl)acetamide (**25e**).* Pale yellow crystals from DMF-H_2_O, yield (77%), mp 215–216 °C. IR (ν cm^−1^): 3284 (N-H), 3022 (C-H arom.), 2993 (C-H aliph.) 2934 (C-H aliph.), 2837 (C-H aliph.),1741 (C=O), 1686 (C=O), 1663 (C=O), 1606, 1579, 1550. ^1^H-NMR (400 MHz, DMSO) δ (ppm): 10.25 (s, 1H, NH), 7.95 (s, 1H, -CH=), 7.47 (d, *J* = 9.1 Hz, 2H, Ar-H), 6.99 (s, 2H, Ar-H), 6.90 (d, *J* = 9.1 Hz, 2H, Ar-H), 4.49 (s, 2H, CH_2_), 3.86 (s, 6H, 2OCH_3_), 3.75 (s, 3H, OCH_3_), 3.73 (s, 3H, OCH_3_). ^13^C-NMR (100 MHz, DMSO) δ (ppm): 167.58 (C=O), 165.75 (C=O), 163.72 (C=O), 155.95 (C), 153.74 (2C), 140.17 (C), 134.25 (-CH=), 132.00 (C), 128.84 (C), 121.17 (2CH), 120.45 (C), 114.45 (2CH), 108.20 (2CH), 60.70 (OCH_3_), 56.53 (2OCH_3_), 55.63 (OCH_3_), 44.38 (CH_2_). Anal. calcd. for C_22_H_22_N_2_O_7_S (458.49): C, 57.63; H, 4.84; N, 6.11; S, 6.99. Found: C, 57.57; H, 4.83; N, 6.02; S, 6.90%.

(4) Synthesis of compounds **26a-e**.

These compounds were synthesized using 2-chloro-*N*-(4-nitrophenyl)acetamide **20** (1 mmol, 0.215 g), following the procedure described for **23a-e**.

*(1) (Z)-2-(5-Benzylidenethiazolidine-2,4-dion-3-yl)-N-(4-nitrophenyl)acetamide (**26a**)*. Fluffy off-white needles from DMF-water, yield (89%), mp 270–272 °C. IR (ν cm^−1^): 3273 (N-H), 3168 (C-H arom.), 3104 (C-H arom.), 2929 (C-H arom.) 2991, 1744 (C=O), 1686 br (C=O), 1619, 1595, 1570. ^1^H-NMR (400 MHz, DMSO) δ (ppm): 11.03 (s, 1H, NH), 8.25 (d, *J* = 9.3 Hz, 2H, Ar-H), 8.01 (s, 1H, -CH=), 7.81 (d, *J* = 9.3 Hz, 2H, Ar-H), 7.67 (d, *J* = 7.1 Hz, 2H, Ph-H), 7.59–7.51 (m, 3H, Ph-H), 4.61 (s, 2H, CH_2_). ^13^C-NMR (100 MHz, DMSO) δ (ppm): 167.57 (C=O), 165.70 (C=O), 165.41 (C=O), 144.88 (C), 143.07 (C), 134.29 (CH), 133.19 (C), 131.35 (C), 130.70 (2CH), 129.90 (2CH), 125.57 (2CH), 121.31 (CH), 119.49 (2CH), 44.70 (CH_2_). Anal. Calcd. for: C_18_H_13_N_3_O_5_S (383.38): C, 56.39; H, 3.42; N, 10.96; S, 8.36. Found: C, 56.30; H, 3.39; N, 10.90; S, 8.30%.

*(2) (Z)-2-(5-(2-Nitrobenzylidene)thiazolidine-2,4-dion-3-yl)-N-(4-nitroyphenyl) acetamide (**26b**).* Yellow crystals from ethanol, yield (87%), mp 213–215 °C. IR (ν cm^−1^): 3263 (N-H), 3164 (C-H arom.), 3100 (C-H arom.), 3072 (C-H arom.) 2938 (C-H aliph.), 1750 (C=O), 1679 br (C=O), 1609, 1594, 1568, 1532. ^1^H NMR (400 MHz, DMSO) δ (ppm): 11.05 (s, 1H, NH), 8.26 (d, *J* = 9.2 Hz, 2H, Ar-H) 8.25 (s, 2H, -CH= + Ar-H ), 7.94 (t, *J* = 7.6 Hz, 1H), 7.82 (d, *J* = 9.2 Hz, 2H, Ar-H), 7.78 (t, *J* = 7.6 Hz, 2H, Ar-H), 4.62 (s, 2H, CH_2_). ^13^C-NMR (100 MHz, DMSO) δ (ppm): 167.34 (C=O), 165.35 (C=O), 164.96 (C=O), 148.30 (C), 144.87 (CH), 143.10 (C), 135.21 (-CH=), 131.86 (C), 131.68 (CH), 129.82 (CH), 129.29 (C), 126.06 (CH), 125.68 (C), 125.95 (2CH), 119.51 (2CH), 44.80 (CH_2_). Anal. calcd. for: C_18_H_12_N_4_O_7_S (428.38): C, 50.47; H, 2.82; N, 13.08; S, 7.48. Found: C, 50.44; H, 2.79; N, 13.01; S, 7.42%.

*(3) (Z)-2-(5-(3-Nitrobenzylidene)thiazolidine-2,4-dion-3-yl)-N-(4-nitroyphenyl) acetamide (**26c**)*. Buff crystals from ethanol-dioxane, yield (89%), mp 264–266 °C. IR (ν cm^−1^): 3342 (N-H), 3087 (CH arom.), 2943 (CH aliph.), 1751 (C=O), 1709 (C=O), 1686 (C=O), 1616, 1562, 1348. ^1^H-NMR (400 MHz, DMSO) δ (ppm): 11.04 (s, 1H, NH), 8.52 (t, *J* = 1.6 Hz, 1H, Ar-H), 8.33 (dd, *J* = 8.2, 2.1 Hz, 1H, Ar-H), 8.25 (d, *J* = 9.2 Hz, 2H, Ar-H), 8.18 (s, 1H, -CH=), 8.08 (d, *J* = 7.8 Hz, 1H, Ar-H), 7.85 (t, *J* = 8.1 Hz, 1H, Ar-H), 7.81 (d, *J* = 9.3 Hz, 2H, Ar-H), 4.63 (s, 2H, CH_2_). ^13^C-NMR (100 MHz, DMSO) δ (ppm): 166.98 (C=O), 165.38 (C=O), 165.29 (C=O), 148.76 (C), 144.85 (C), 143.08 (C), 135.91 (-CH=), 134.94 (CH), 132.04 (CH), 131.46 (C), 125.57 (2CH), 125.43 (C), 125.27 (CH), 124.24 (CH), 119.49 (2CH), 44.63 (CH_2_). Anal. calcd. for: C_18_H_12_N_4_O_7_S (428.38): C, 50.47; H, 2.82; N, 13.08; S, 7.48. Found: C, 50.34; H, 2.78; N, 13.01; S, 7.37%.

*(4) (Z)-2-(5-(4-Nitrobenzylidene)thiazolidine-2,4-dion-3-yl)-N-(4-nitroyphenyl) acetamide (**26d**)*. Yellow crystals from DMF-H_2_O, yield (85%), mp 284–285 °C. IR (ν cm^−1^): 3264 (N-H), 3075 (C-H arom.), 2994 (C-H aliph.), 1756 (C=O), 1698 (C=O), 1609, 1593, 1517. ^1^H-NMR (400 MHz, DMSO) δ (ppm): 11.03 (s, 1H, NH), 8.36 (d, *J* = 8.8 Hz, 2H, Ar-H), 8.24 (d, *J* = 9.2 Hz, 2H, Ar-H), 8.11 (s, 1H, -CH=), 7.92 (d, *J* = 8.8 Hz, 2H, Ar-H), 7.80 (d, *J* = 9.3 Hz, 2H, Ar-H), 4.63 (s, 2H, CH_2_). ^13^C-NMR (100 MHz, DMSO) δ (ppm): 167.05 (C=O), 165.38 (C=O), 165.25 (C=O), 148.20 (C), 144.83 (-CH=), 143.08 (C), 139.47 (C), 131.73 (C), 131.62 (2CH), 125.55 (2CH + C), 124.78 (2CH), 119.49 (2CH), 44.84 (CH_2_). Anal. calcd. for: C_18_H_12_N_4_O_7_S (428.38): C, 50.47; H, 2.82; N, 13.08; S, 7.48. Found: C, 50.39; H, 2.77; N, 13.18; S, 7.42%.

*(5) (Z)-N-(4-Nitrophenyl)-2-(5-(3,4,5-trimethoxybenzylidene)thiazolidine-2,4-dion-3-yl)acetamide (**26e**)*. Yellow crystals from DMF-H_2_O, yield (80%), mp 253–254 °C. IR (ν cm^−1^): 3282 (N-H), 3091 (C-H arom.), 2940 (C-H aliph.), 2893 (C-H aliph.), 1738 (C=O), 1682 (C=O), 1668, (C=O), 1607, 1596, 1507. ^1^H-NMR (400 MHz, DMSO) δ (ppm): 11.03 (s, 1H, NH), 8.25 (d, *J* = 9.3 Hz, 2H, Ar-H), 7.96 (s, 1H, -CH=), 7.82 (d, *J* = 9.3 Hz, 2H, Ar-H), 6.99 (s, 2H, Ar-H), 4.61 (s, 2H, CH_2_), 3.86 (s, 6H, 2OCH_3_), 3.75 (s, 3H, OCH_3_). ^13^C-NMR (100 MHz, DMSO) δ (ppm): 167.54 (C=O), 165.65 (C=O), 165.43 (C=O), 153.74 (2C), 144.89 (C), 143.07 (-CH=), 140.24 (C), 134.51 (C), 128.78 (C), 125.58 (2CH), 120.24 (C), 119.48 (2CH), 108.24 (2CH), 60.71 (OCH_3_), 56.53 (2OCH_3_), 44.68 (CH_2_). Anal. calcd. for: C_21_H_19_N_3_O_8_S (473.46): C, 53.27; H, 4.05; N, 8.88; S, 6.77. Found: C, 53.35; H, 4.00; N, 8.79; S, 6.70%.

(5) Synthesis of compounds **27b-e**.

These compounds were synthesized using 2-chloro-*N*-(4-chlorophenyl)acetamide **21** (1 mmol, 0.204 g), following the procedure described for **23a-e**.

*(1) (Z)-N-(4-Chlorophenyl)-2-(5-(2-nitrobenzylidene)thiazolidine-2,4-dion-3-yl) acetamide (**27b**).* Canary yellow crystals from ethanol-dioxane, yield (85%), mp 188–190 °C. IR (ν cm^−1^): 3258 (N-H), 3123 (C-H arom.), 3073 (C-H arom.) 2992 (C-H aliph.), 2835 (C-H aliph.), 1756 (C=O), 1693 (C=O), 1688 (C=O), 1607, 1570, 1546. ^1^H-NMR (400 MHz, DMSO) δ (ppm): 10.57 (s, 1H, NH), 8.25 (d, *J* = 8.5 Hz, 2H, Ar-H + -CH=), 7.93 (t, *J* = 7.4 Hz, 1H, Ar-H), 7.77 (t, *J* = 7.5 Hz, 2H, Ar-H), 7.60 (d, *J* = 8.9 Hz, 2H, Ar-H), 7.39 (d, *J* = 8.9 Hz, 2H, Ar-H), 4.55 (s, 2H, CH_2_). ^13^C-NMR (100 MHz, DMSO) δ (ppm): 167.35 (C=O), 165.00 (C=O), 164.39 (C=O), 148.29 (C), 137.78 (C), 135.19 (CH), 131.81 (C), 131.48 (CH), 129.80 (CH), 129.29 (C+2CH), 127.81 (C), 126.04 (CH), 125.78 (C), 121.23 (2CH), 44.61 (CH_2_). Anal. calcd. for: C_18_H_12_ClN_3_O_5_S (417.02): C, 51.74; H, 2.90; N, 10.06; S, 7.67. Found: C, 51.65; H, 2.86; N, 10.00; S, 7.59%.

*(2) (Z)-N-(4-Chlorophenyl)-2-(5-(3-nitrobenzylidene)thiazolidine-2,4-dion-3-yl) acetamide (**27c**)*. Fluffy white crystals from ethanol-dioxane, yield (90%), mp 260–261 °C. IR (ν cm^−1^): 3349 (N-H), 3075 (CH arom.), 2943 (CH aliph.), 1750 (C=O), 1699_br_ (C=O), 1607 (C=O), 1603, 1570. ^1^H-NMR (400 MHz, DMSO) δ (ppm): 10.56 (s, 1H, NH), 8.52 (t, *J* = 1.9 Hz, 1H, Ar-H), 8.34 (ddd, *J* = 8.3, 2.2, 0.8 Hz, 1H, Ar-H), 8.18 (s, 1H, -CH=), 8.09 (d, *J* = 7.8 Hz, 1H, Ar-H), 7.86 (t, *J* = 8.0 Hz, 1H, Ar-H), 7.59 (d, *J* = 8.9 Hz, 2H, Ar-H), 7.39 (d, *J* = 8.9 Hz, 2H, Ar-H), 4.55 (s, 2H, CH_2_). ^13^C-NMR (100 MHz, DMSO) δ (ppm): 167.00 (C=O), 165.43 (C=O), 164.35 (C=O), 148.76 (C), 137.77 (C), 135.90 (-CH=), 134.97 (CH), 131.90 (CH), 131.46 (CH), 129.29 (2CH), 127.82 (C), 125.31 (C), 125.25 (CH), 124.34 (C), 121.23 (2CH), 44.66 (CH_2_). Anal. calcd. for: C_18_H_12_ClN_3_O_5_S (417.02): C, 51.74; H, 2.90; N, 10.06; S, 7.67. Found: C, 51.62; H, 2.84; N, 9.99; S, 7.55%.

*(3) (Z)-N-(4-Chlorophenyl)-2-(5-(4-nitrobenzylidene)thiazolidine-2,4-dion-3-yl) acetamide (**27d**).* Yellow crystals from DMF-H_2_O, yield (90%), mp 252–254 °C. IR (ν cm^−1^): 3276 (N-H), 3120 (C-H arom.), 2994 (C-H aliph.), 1748 (C=O), 1704 (C=O), 1675 (C=O), 1595, 1541, 1491. ^1^H-NMR (400 MHz, DMSO) δ (ppm): 10.55 (s, 1H, NH), 8.37 (d, *J* = 8.8 Hz, 2H, Ar-H), 8.12 (s, 1H, -CH=), 7.93 (d, *J* = 8.8 Hz, 2H, Ar-H), 7.59 (d, *J* = 8.9 Hz, 2H, Ar-H), 7.39 (d, *J* = 8.9 Hz, 2H, Ar-H), 4.55 (s, 2H, CH_2_). ^13^C-NMR (100 MHz, DMSO) δ (ppm): 167.08 (C=O), 165.43 (C=O), 164.33 (C=O), 148.20 (C), 139.54 (C), 137.76 (-CH=), 131.61 (4CH), 129.29 (2CH), 127.82 (C), 125.68 (C), 124.80 (2CH), 121.23 (C), 44.68 (CH_2_). Anal. calcd. for: C_18_H_12_ClN_3_O_5_S (417.02): C, 51.74; H, 2.90; N, 10.06; S, 7.67. Found: C, 51.68; H, 2.88; N, 10.01; S, 7.66%.

*(4) (Z)-N-(4-Chlorophenyl)-2-(5-(3,4,5-trimethoxybenzylidene)thiazolidine-2,4-dion-3-yl)acetamide (**27e**).* Yellow crystals from DMF-H_2_O, yield (79%), mp 218–219 °C. IR (ν cm^−1^): 3295 (N-H), 3193 (C-H arom.), 3125 (C-H arom.), 3074 (C-H arom.) 2991 (C-H aliph.), 2838 (C-H aliph.), 1750 (C=O), 1686 (C=O), 1677 (C=O), 1603, 1577, 1507. 1H-NMR (400 MHz, DMSO) δ (ppm): 10.55 (s, 1H, NH), 7.95 (s, 1H, -CH=), 7.59 (d, *J* = 8.9 Hz, 2H, Ar-H), 7.39 (d, *J* = 8.9 Hz, 2H, 2Ar-H), 6.99 (s, 2H, Ar-H), 4.53 (s, 2H, CH_2_), 3.85 (s, 6H, 2 OCH_3_), 3.74 (s, 3H, OCH_3_). ^13^C-NMR (100 MHz, DMSO) δ (ppm): 167.55 (C=O), 165.70 (C=O), 164.48 (C=O), 153.73 (2C), 140.20 (C), 137.81 (C), 134.37 (-CH=), 129.28 (2CH), 128.80 (C), 127.77 (C), 121.21 (2CH), 120.33 (C), 108.22 (2CH), 60.70 (OCH_3_), 56.52 (2OCH_3_), 44.49 (CH_2_). Anal. calcd. for: C_21_H_19_ClN_2_O_6_S (462.90): C, 54.49; H, 4.14; N, 6.05; S, 6.93. Found: C, 54.42; H, 4.13; N, 6.12; S, 6.98%.

(6) Synthesis of compounds **28a-e**.

These compounds were synthesized using 2-chloro-*N*-(2-chlorophenyl)acetamide **22** (1 mmol, 0.204 g), following the procedure described for **23a-e**.

*(1) (Z)-2-(5-Benzylidenethiazolidine-2,4-dion-3-yl)-N-(2-chlorophenyl)acetamide (**28a**).* White aggregate crystals from ethanol-dioxane, yield (87%), mp 243–245 °C. IR (ν cm^−1^): 3260 (N-H), 3020 (C-H arom.) 1747 (C=O), 1694 (C=O), 1668 (C=O), 1608, 1597, 1587. ^1^H-NMR (400 MHz, DMSO) δ (ppm): ^1^H NMR (400 MHz, DMSO) δ (ppm): 10.07 (s, 1H, NH), 8.00 (s, 1H, =CH-), 7.71–7.66 (m, 3H, Ar-H), 7.62–7.44 (m, 4H, Ar-H), 7.34 (t, *J* = 7.5 Hz, 1H, Ar-H), 7.22 (t, *J* = 7.3, 1H, Ar-H), 4.62 (s, 2H, CH_2_). ^13^C-NMR (100 MHz, DMSO) δ (ppm): 167.56 (C=O), 165.73 (C=O), 164.97 (C=O), 134.68 (C), 134.07 (-CH=), 133.33 (CH), 131.29 (CH), 130.68 (2CH), 130.10 (CH), 129.90 (2CH), 128.02 (CH), 127.20 (C), 126.88 (C), 126.68 (C), 121.48 (CH), 44.31 (CH_2_). Anal. calcd. for C_18_H_13_ClN_2_O_3_S (372.82): C, 57.99; H, 3.51; N, 7.51; S, 8.60. Found: C, 57.95; H, 3.40; N, 7.48; S, 8.53%.

*(2) (Z)-N-(2-Chlorophenyl)-2-(5-(2-nitrobenzylidene)thiazolidine-2,4-dion-3-yl) acetamide (**28b**)*. Small buff crystals from ethanol-dioxane, yield (80%), mp 174–175 °C. IR (ν cm^−1^): 3346 (N-H), 3065 (CH arom.), 2934 (CH aliph.), 1751 (C=O), 1703 (C=O), 1697 (C=O), 1616, 1569, 1535. ^1^H-NMR (400 MHz, DMSO) δ (ppm): 10.09 (s, 1H, NH), 8.25 (m, 2H, Ar-H + -CH=), 7.93 (t, *J* = 7.6 Hz, 1H, Ar-H), 7.79–7.75 (m, 2H, Ar-H), 7.71 (dd, *J* = 8.0, 1.1 Hz, 1H, Ar-H), 7.53 (dd, *J* = 8.0, 1.1 Hz, 1H, Ar-H), 7.35 (td, *J* = 8.3, 1.2 Hz, 1H, Ar-H), 7.23 (td, *J* = 7.9, 1.2 Hz, 1H, Ar-H), 4.62 (s, 2H, CH_2_). ^13^C-NMR (100 MHz, DMSO) δ (ppm): 167.33 (C=O), 164.94 (C=O), 164.89 (C=O), 148.31 (C), 135.19 (-CH=), 134.65 (C), 131.80 (CH), 131.37 (CH), 130.11 (CH), 129.80 (CH), 129.30 (C), 128.03 (CH), 127.24 (CH), 127.08 (C), 126.91 (C), 126.51 (C), 126.04 (CH), 125.84 (CH), 44.39 (CH_2_). Anal. calcd. for C_18_H_12_ClN_3_O_5_S (417.02): C, 51.74; H, 2.90; N, 10.06; S, 7.67. Found: C, 51.65; H, 2.91; N, 10.00; S, 7.77%.

*(3) (Z)-N-(2-Chlorophenyl)-2-(5-(3-nitrobenzylidene)thiazolidine-2,4-dion-3-yl) acetamide (**28c**).* White crystals from DMF-H_2_O, yield (69%), mp 240–242 °C. IR (ν cm^−1^): 3252 (NH), 3068 (C-H arom.), 3013 (C-H arom.), 2914 (C-H aliph.), 1745 (C=O), 1683 (C=O), 1668 (C=O), 1604, 1570, 1533, 1476. ^1^H-NMR (400 MHz, DMSO) δ (ppm): 10.09 (s, 1H, NH), 8.53 (t, *J* = 1.9 Hz, 1H, Ar-H), 8.33 (ddd, *J* = 8.3, 2.2, 0.8 Hz, 1H, Ar-H), 8.18 (s, 1H, =CH-), 8.09 (d, *J* = 7.9 Hz, 1H, Ar-H), 7.86 (t, *J* = 8.0 Hz, 1H, Ar-H), 7.86 (t, *J* = 8.0 Hz, 1H, Ar-H), 7.70 (dd, *J* = 8.1, 1.5 Hz, 1H, Ar-H), 7.53 (dd, *J* = 8.0, 1.4 Hz, 1H, Ar-H), 7.34 (td, *J* = 7.9, 1.4 Hz, 1H, Ar-H), 7.23 (td, *J* = 7.9, 1.4 Hz, 1H, Ar-H), 4.36 (s, 2H, CH_2_). ^13^C-NMR (100 MHz, DMSO) δ (ppm): 167.00 (C=O), 165.43 (C=O), 164.87 (C=O), 148.78 (C), 135.89 (-CH=), 135.00 (CH), 134.64 (CH), 131.82 (CH), 131.48 (CH), 130.11 (CH), 128.03 (C), 127.26 (C), 126.97 (C), 126.51 (CH), 125.28 (CH), 125.28 (CH), 124.44 (C), 44.45 (CH_2_). Anal. calcd. for: C_18_H_12_ClN_3_O_5_S (417.02): C, 51.74; H, 2.90; N, 10.06; S, 7.67. Found: C, 51.64; H, 2.81; N, 10.01; S, 7.59%.

*(4) (Z)-N-(2-Chlorophenyl)-2-(5-(4-nitrobenzylidene)thiazolidine-2,4-dion-3-yl) acetamide (**28d**)*. Pale yellow crystals from DMF-H2O, yield (89%), mp 242–244 °C. IR (ν cm^−1^): 3280 (N-H), 3119 (C-H arom.), 3050 (C-H arom.) 2947 (C-H aliph.), 1757 (C=O), 1702 (C=O), 1682 (C=O), 1610, 1592, 1540. ^1^H-NMR (400 MHz, DMSO) δ (ppm): 10.09 (s, 1H, NH), 8.37 (d, *J* = 8.8 Hz, 2H, Ar-H), 8.12 (s, 1H, -CH=), 7.93 (d, *J* = 8.8 Hz, 2H, Ar-H), 7.70 (dd, *J* = 8.8, 0.7 Hz, 1H, Ar-H), 7.53 (dd, *J* = 8.0, 1.3 Hz, 1H, Ar-H), 7.34 (td, *J* = 8.0, 1.3 Hz, 1H, Ar-H), 7.23 (td, *J* = 7.9, 1.3 Hz, 1H, Ar-H), 4.63 (s, 2H, CH_2_). ^13^C-NMR (100 MHz, DMSO) δ (ppm): 167.07 (C=O), 165.42 (C=O), 164.84 (C=O), 148.20 (C), 139.57 (-CH=), 134.63 (CH), 131.61 (2CH), 131.53 (CH), 130.11 (CH), 128.03 (CH), 127.26 (C), 126.94 (C), 126.52 (C), 125.78 (C), 124.82 (2CH), 44.47 (CH_2_). Anal. calcd. for: C_18_H_12_ClN_3_O_5_S (417.02): C, 51.74; H, 2.90; N, 10.06; S, 7.67. Found: C, 51.67; H, 2.80; N, 10.11; S, 7.61%.

*(5) (Z)-N-(2-Chlorophenyl)-2-(5-(3,4,5-trimethoxybenzylidene)thiazolidine-2,4-dion-3-yl)acetamide (**28e**).* Pale yellow crystals from DMF-H_2_O, yield (75%), mp 222–223 °C. IR (ν cm^−1^): 3251 (N-H), 3047 (C-H arom.), 2938 (C-H aliph.), 2835 (C-H aliph.), 1740 (C=O), 1686 (C=O), 1671 (C=O), 1605, 1578, 1505. ^1^H-NMR (400 MHz, DMSO) δ (ppm): 10.07 (s, 1H, NH), 7.95 (s, 1H, -CH=), 7.70 (dd, *J* = 8.1, 1.3 Hz, 1H, Ar-H), 7.53 (dd, *J* = 8.0, 1.3 Hz, 1H, Ar-H), 7.34 (td, *J* = 7.9, 1.4 Hz, 1H, Ar-H), 7.23 (td, *J* = 7.9, 1.4 Hz, 1H, Ar-H), 6.99 (s, 2H, Ar-H), 4.61 (s, 2H, CH_2_), 3.85 (s, 6H, 2 OCH_3_), 3.75 (s, 3H, OCH_3_). ^13^C-NMR (100 MHz, DMSO) δ (ppm): 167.54 (C=O), 165.68 (C=O), 164.97 (C=O), 153.74 (2C), 140.18 (CH), 134.68 (C), 134.30 (-CH=), 130.26 (CH), 128.83 (CH), 128.02 (C), 127.19 (C), 126.85 (C), 126.46 (CH), 120.41 (C), 108.20 (2CH), 60.80 (OCH_3_), 56.53 (2OCH_3_), 44.27 (CH_2_). Anal. calcd. for: C_21_H_19_ClN_2_O_6_S (462.90): C, 54.49; H, 4.14; N, 6.05; S, 6.93. Found: C, 54.41; H, 4.09; N, 6.01; S, 6.88%.

##### Synthesis of (*Z*)-2-(5-(3-Nitrobenzylidene)thiazolidin-2,4-dion-3-yl)acetic Acid (**29**)

A mixture of the ester **12c** (1.0 g, 3 mmol), glacial AcOH (16 mL) and concentrated HCl (12 N, 6 mL) was heated under reflux for 2 h. After evaporation under reduced pressure, the residue obtained was heated again under reflux in glacial AcOH (16 mL) and concentrated HCl (6 mL) for 2 h. After evaporation till dryness under reduced pressure, the crude product was washed thoroughly with water and recrystallized from ethanol-water to give off-white crystals, 0.8 g, yield 92%, mp 191–193 °C [lit. [35], mp 235 °C]. IR (ν cm^−1^): 3409 (O-H), 3077 (C-H arom.), 3037 (C-H arom.), 2950 (C-H aliph.), 2784, 2665, 2566 (O-H acid), 1756 (C=O), 1740 (C=O), 1701 (C=O), 1686 (C=O), 1604, 1535. ^1^H-NMR (400 MHz, DMSO) δ (ppm): 8.50 (t, *J* = 1.9 Hz, 1H, Ar-H), 8.32 (dd, *J* = 8.2, 1.5 Hz, 1H, Ar-H), 8.15 (s, 1H, =CH-), 8.06 (d, *J* = 7.9 Hz, 1H, Ar-H), 7.84 (t, *J* = 8.0 Hz, 1H, Ar-H), 4.36 (s, 2H, CH_2_). ^13^C-NMR (100 MHz, DMSO) δ (ppm): 168.41 (C=O), 166.79 (C=O), 165.24 (C=O), 148.64 (C), 135.84 (=CH-), 134.95 (C), 131.95 (CH), 131.43 (CH), 125.30 (CH), 125.24 (CH), 124.24 (C), 43.25 (CH_2_). Anal. calcd. for C_12_H_8_N_2_O_6_S (308.26): C, 46.76; H, 2.62; N, 9.09; S, 10.40. Found: C, 46.85; H, 2.67; N, 10.00; S, 10.46%.

### 3.2. Biology

#### 3.2.1. In Vitro Antileishmanial Activity on the Promastigote Stage of *Leishmania infantum*

Luciferase-expressing *Leishmania infantum* was kindly provided by Professor Thierry Lang of the Institut Pasteur [36]. *L. infantum* promastigotes were routinely grown at 27 °C in Schneider’s Drosophila Medium (Dominique DUTSCHER SAS, Bernolsheim, France) supplemented with 10% fetal calf serum, 2% human urine, sodium pyruvate 0.04 mM, hemin 0.1 µg/mL, HEPES 5 mM, streptomycin 100 µg/mL, penicillin 100 U/mL, L-glutamine 2 mM (culture medium). The in vitro evaluation of the antileishmanial activity of the tested compound on *L. infantum* promastigotes forms was assessed according to the Mosmann protocol [37] with slight modifications. Briefly, *L. infantum* promastigotes in logarithmic phase of growth were incubated at an average density of 1 × 10^6^ parasites/mL in sterile 96-well plates with various concentrations of compound dissolved in DMSO (final concentration less than 0.5% *v*/*v*), in duplicate. Appropriate controls treated with DMSO, Miltefosine, Amphotericin B and Fexinidazole sulfone (reference drugs purchased from Sigma-Aldrich, Saint-Louis, MO, USA) were added to each set of experiments. The plates were incubated at 27 °C for 72 h, Then, each plate-well was microscope-analyzed to detect any precipitate formation. Afterward, 20 µL of MTT (3-(4,5-dimethylthiazol-2-yl)-2,5-diphenyltetrazolium bromide) (Sigma-Aldrich, Saint-Louis, MI, USA) solution (5 mg/mL in PBS) were added to each well and incubated at 27 °C for 4 h. The reaction was then stopped by the addition of 100 µL of 10% sodium dodecyl sulfate—50% isopropanol. Plates were shaken vigorously at 400 rpm for 10 min. The absorbance was measured at 570 nm in a TECAN Infinite F-200 spectrophotometer (Tecan Trading AG, Männedorf, Switzerland). An inhibitory concentration of 50% (EC_50_) was defined as the concentration of drug required to inhibit the metabolic activity of leishmanial promastigote forms by 50% relative to the control. EC_50_ values were calculated by non-linear regression analysis processed dose-response curves, using TableCurve 2D V5.0 software (Systat Software Inc. (Palo Alto, CA, USA)). EC_50_ values represent the mean value calculated from at least three separate experiments.

#### 3.2.2. In Vitro Cytotoxicity on the Human Hepatic HepG2 Cell Line

HepG2 cell line (hepatocarcinoma cell line purchased from ATCC, ref HB-8065) was maintained at 37 °C, 5% CO_2_ with 90% humidity in MEM supplemented with 10% fetal bovine serum, 1% L-glutamine (200 mM) and penicillin (100 U/mL)/streptomycin (100 mg/mL) (complete MEM medium). The tested molecules’ cytotoxicity was assessed according to the method of Mosmann [37] with slight modifications. Briefly, 5 × 10^3^ cells in 100 μL of complete medium were inoculated into each well of 96-well plates and incubated at 37 °C in a humidified 5% CO_2_. After 24 h incubation, 100 μL of medium with various product concentrations dissolved in DMSO (final concentration less than 0.5% *v*/*v*) were added and the plates were incubated for 72 h at 37 °C. Triplicate assays were performed for each sample. Each plate well was then microscope-examined to detect possible precipitate formation before the medium was aspirated from the wells. Then, 100 μL of MTT (3-(4,5-dimethyl-2-thiazolyl)-2,5-diphenyl-2*H*-tetrazolium bromide) solution (0.5 mg/mL in medium without FCS) were then added to each well. Cells were incubated for 2 h at 37 °C, following which the MTT solution was removed and DMSO (100 μL) was added to dissolve the resulting blue formazan crystals. Plates were shaken vigorously (700 rpm) for 10 min. The absorbance was measured at 570 nm with 630 nm as reference wavelength using a TECAN Infinite F-200 spectrophotometer (Tecan Trading AG, Männedorf, Switzerland). DMSO was used as blank and doxorubicin (purchased from Sigma Aldrich, Saint Louis, MI, USA) as positive control. Cell viability was calculated as a percentage of control (cells incubated without compound). The 50% cytotoxic concentration (CC_50_) was determined from the dose–response curve by using the Table Curve 2D v5.0 software (Systat Software Inc.; Palo Alto, CA, USA). CC_50_ values represent the mean value calculated from three separate experiments.

#### 3.2.3. In Vitro Cytotoxicity on the Human Monocyte THP-1 Cell Line

The undifferentiated THP1 human monocyte cells (acute monocytic leukemia cell line purchased from ATCC, ref TIB-202) were routinely grown in RPMI 1640 medium (Life Technologies, Saint-Aubin, France) supplemented with 10% FCS (Life Technologies, Saint-Aubin, France), 100 U/mL penicillin, 100 µg/mL streptomycin and 2 mM L-glutamine at 37 °C, 5% CO_2_. The culture was maintained between 3.10^5^ and 8.10^5^ cells/mL. The in vitro evaluation of the cytotoxicity of the tested compound was assessed according to the method of Mosmann [37] with slight modifications. Briefly, 100 μL of THP-1 cells with Phorbol 12-Myristate 13-Acetate (final concentration 50 ng/mL) were seeded in 96-well plates at an average density of 1 × 10^6^ cells/mL and incubated for 24 h at 37 °C, 5% CO_2_. Wells were washed with RPMI and 100 µL of fresh complete medium was added. Then, 100 µL of various concentrations of compounds dissolved in DMSO (final concentration less than 0.5% *v*/*v*) and reference were added in duplicate. The plates were incubated for 72 h at 37 °C, 5% CO_2_. Each well plate was then microscope-examined to detect possible precipitate formation before the medium was removed from the wells. Then, 100 µL of MTT solution (0.5 mg/mL in medium) was added to each well. Cells were incubated for 2 h at 37 °C. Then, the MTT solution was removed and DMSO (100 µL) was added to dissolve the resulting blue formazan crystals. Plates were shaken vigorously (700 rpm) for 10 min. The absorbance was measured at 570 nm using a TECAN Infinite F-200 spectrophotometer (Tecan Trading AG, Männedorf, Switzerland). DMSO was used as blank and doxorubicin (purchased from Sigma Aldrich) as positive control. Cell viability was calculated as a percentage of control (cells incubated with DMSO). The 50% cytotoxic concentration (CC_50_) was determined from the dose–response curve by using the TableCurve 2D V5 software Systat Software Inc. (Palo Alto, CA, USA). CC_50_ values represent the mean value calculated from three separate experiments.

#### 3.2.4. Assessing Antileishmanial Activity of 14c against *L. donovani* Promastigote Cell Lines Overexpressing NTR1 and NTR2

EC_50_ values were determined as previously described [24]. Nifurtimox was used as a positive control for activation via NTR1 [38] while delamanid was used as a positive control for activation via NTR2 [39].

## 4. Conclusions

To conclude, a series of sixty-one 5-stryryl-thiazolidine-2,4-diones was synthesized in 2–5 reaction steps and evaluated in vitro against the promastigote stage of *Leishmania infantum*. Several nitroaromatic derivatives were active, while the hit derivative proved to be **14c**, bearing a nitro group at the *meta* position of the phenyl ring of the 5-benzylidene moiety and a cyanomethyl function at the 3-position of the thiazolidine-2,4-dione ring. Compound **14c** showed similar activity to miltefosine, the only orally available antileishmanial drug compound, and similar cytotoxicity on the human HepG2 and THP-1 cell lines. Because of its nitroaromatic structure, **14c** behaves as a prodrug, bioactivated predominantly by the parasite Type 1 nitroreductases, probably generating cytotoxic metabolites in the parasite cell. Finally, hit-molecule **14c** is a lipophilic compound. However, it remains soluble in water, allowing further hit-to-lead medicinal chemistry to search for novel antileishmanial candidates that could be orally administered.

## Figures and Tables

**Table 1:** 1,3-Thiazolidine-2,4-diones (**12a-e to 16a-e**).

Cpd	R_1_	Cpd	R_1_	Cpd	R_1_
**12a**	H	**13e**	3,4,5-Tri-OCH_3_	**15d**	4-NO_2_
**12b**	2-NO_2_	**14a**	H	**15e**	3,4,5-Tri-OCH_3_
**12c**	3-NO_2_	**14b**	2-NO_2_	**16a**	H
**12d**	4-NO_2_	**14c**	3-NO_2_	**16b**	2-NO_2_
**12e**	3,4,5-Tri-OCH_3_	**14d**	4-NO_2_	**16c**	3-NO_2_
**13a**	H	**14e**	3,4,5-Tri-OCH_3_	**16d**	4-NO_2_
**13b**	2-NO_2_	**15a**	H	**16e**	3,4,5-Tri-OCH_3_
**13c**	3-NO_2_	**15b**	2-NO_2_		
**13d**	4-NO_2_	**15c**	3-NO_2_		

**Table 2:** 1,3-Thiazolidine-2,4-diones (**23a-e** to **28a-e**).

Cpd	R_1_	Cpd	R_1_	Cpd	R_1_
**23a**	H	**25a**	H	**27a**	H
**23b**	2-NO_2_	**25b**	2-NO_2_	**27b**	2-NO_2_
**23c**	3-NO_2_	**25c**	3-NO_2_	**27c**	3-NO_2_
**23d**	4-NO_2_	**25d**	4-NO_2_	**27d**	4-NO_2_
**23e**	3,4,5-Tri-OCH_3_	**25e**	3,4,5-Tri-OCH_3_	**27e**	3,4,5-Tri-OCH_3_
**24a**	H	**26a**	H	**28a**	H
**24b**	2-NO_2_	**26b**	2-NO_2_	**28b**	2-NO_2_
**24c**	3-NO_2_	**26c**	3-NO_2_	**28c**	3-NO_2_
**24d**	4-NO_2_	**26d**	4-NO_2_	**28d**	4-NO_2_
**24e**	3,4,5-Tri-OCH_3_	**26e**	3,4,5-Tri-OCH_3_	**28e**	3,4,5-Tri-OCH_3_

**Table 3:** Results of the in vitro evaluations (activity and cytotoxicity on HepG2) of the 21 compounds presenting both good solubility in the culture medium (Schneider’s Drosophila Medium) and antileishmanial EC_50_ activity values < 30 µM.

Cpd	Structure	R_1_	R_2_	Anti-*L. infantum* Promastigote Activity EC_50_ (µM)	HepG2 Cytotoxicity CC_50_ (µM)	Selectivity INDEX (SI = CC_50_/EC_50_)
**4**	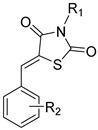	-H	3-NO_2_	28.04 ± 7.15	190.26 ± 18.64	6.8
**5**		-H	4-NO_2_	14.60 ± 2.90	137.11 ± 1.22	9.4
**12a**		-CH_2_COOEt	-H	20.55 ± 5.86	193.84 ± 8.92	9.4
**12b**		-CH_2_COOEt	2-NO_2_	24.07 ± 5.14	39.35 ± 7.09	1.6
**12c**		-CH_2_COOEt	3-NO_2_	5.51 ± 1.29	>125 *	>22.7
**12d**		-CH_2_COOEt	4-NO_2_	11.60 ± 2.37	>125 *	>10.8
**14c**		-CH_2_CN	3-NO_2_	7.00 ± 2.96	100.77 ± 19.33	14.4
**14d**		-CH_2_CN	4-NO_2_	24.80 ± 6.09	108.52 ± 11.04	4.4
**15c**		-CH_2_CONH2	3-NO_2_	13.31 ± 7.75	>62.5 *	>4.7
**16b**	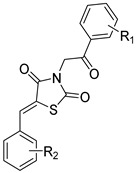	-H	2-NO_2_	5.57 ± 1.28	11.04 ± 2.28	2.0
**16c**		-H	3-NO_2_	3.22 ± 0.94	>12.5 *	>3.9
**23b**	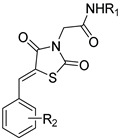	-Ph	2-NO_2_	11.60 ± 3.43	22.28 ± 0.59	1.9
**24b**		4-MePh-	2-NO_2_	24.76 ± 6.68	30.95 ± 3.97	1.2
**25b**		4-OMePh-	2-NO_2_	22.32 ± 4.93	23.29 ± 2.93	1.1
**26b**		4-NO_2_Ph-	2-NO_2_	14.64 ± 2.91	30.12 ± 3.94	2.1
**26c**		4-NO_2_Ph-	3-NO_2_	7.20 ± 1.48	>25 *	>3.5
**26d**		4-NO_2_Ph-	4-NO_2_	14.10 ± 2.80	>25 *	>1.8
**27b**		4-ClPh-	2-NO_2_	16.32 ± 3.85	34.55 ± 3.03	2.1
**27d**		4-ClPh-	4-NO_2_	17.30 ± 4.17	>12.5 *	>0.7
**27e**		4-ClPh-	3,4,5-OMe	5.00 ± 1.98	19.35 ± 2.05	3.9
**28b**		2-ClPh-	2-NO_2_	16.07 ± 4.32	22.98 ± 3.20	1.4
**29**	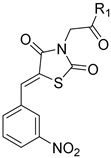	-OH	3-NO_2_	>50	>250	-
Amphotericin B				0.06 ± 0.02	8.80 ± 0.30	146.7
Miltefosine				3.30 ± 0.80	85.00 ± 8.80	25.8
Fexinidazole sulfone				3.10 ± 0.70	>200	>64.5
Doxorubicin				-	0.20 ± 0.02	-

* Compound cytotoxicity could not be evaluated at higher concentrations because of a lack of solubility in the complete MEM culture medium at 37 °C.

**Table 4:** THP-1 cytotoxicity and physicochemical properties of hit compound **14c**.

Cpd	Structure	THP-1 CC_50_ (µM)	CHI logD_7.4_	PBS Solubility ^a^ (log S)
**14c**	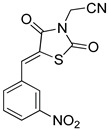	121 ± 9	2.85	4.8
Doxorubicin		2.2 ± 0.1	-	
Amphotericin B		17.6 ± 3	-	
Miltefosine		30 ± 4	-	
Fexinidazole sulfone		>200	-	

^a^ PBS: Phosphate-buffered saline (pH = 7.4).

**Table 5:** Sensitivity of wild-type and NTR-overexpressing *L. donovani* promastigotes to compound **14c**.

Cell Line	EC_50_ Values, nM		
	14c	Nifurtimox	Delamanid
WT	22,085 ± 1914	3108 ± 223	3 ± 0.1
NTR1^OE^	3777 ± 313	144 ± 10	2 ± 0.3
NTR2^OE^	18,467 ± 1007	163 ± 11	0.3 ± 0.001

Data represent the weighted mean ± standard deviation for five independent experiments.

## Data Availability

Data are contained within the article and supplementary materials.

## Supplementary Materials

The following supporting information can be downloaded at: https://www.mdpi.com/article/10.3390/ph17070878/s1, S1. Organic synthesis and product characterization of the known compounds used in our study; ^1^H and ^13^C spectra of the synthesized compounds.
