# Supplementary material for: Synthesis of Nitrostyrylthiazolidine-2,4-dione Derivatives Displaying Antileishmanial Potential
Source: Pharmaceuticals (Basel). 2024 Jul 3;17(7):878. doi: 10.3390/ph17070878 (PMC11280390; doi:10.3390/ph17070878)
Supplement: Supplementary file 1 [file pharmaceuticals-17-00878-s001.zip › pharmaceuticals-2938346-supplementary.pdf]

# Synthesis of Nitrostyrylthiazolidine-2,4-dione Derivatives Displaying Antileishmanial Potential

**Omar Khoumeri [1], Sébastien Hutter [2], Nicolas Primas [1,3,*], Caroline Castera-Ducros [1,3], Sandra Carvalho [4], Susan Wyllie [4], Mohamed Lotfi Efrit [5], Dimitri Fayolle [6], Marc Since [6], Patrice Vanelle [1,3], Pierre Verhaeghe [7,8], Nadine Azas [2] and Hussein El-Kashef [9,10,*]**

1 Team Pharmaco-Chimie Radicalaire, Faculté de Pharmacie, Aix Marseille University, CNRS, ICR UMR 7273, 27 Boulevard Jean Moulin, CS30064, CEDEX 05, 13385 Marseille, France; omar.khoumeri@univ-amu.fr (O.K.); caroline.ducros@univ-amu.fr (C.C.-D.); patrice.vanelle@univ-amu.fr (P.V.)
2 IHU Méditerranée Infection, UMR RITMES, TEAM-VEPTE, Aix Marseille University, 19–21 Boulevard Jean Moulin, 13005 Marseille, France; sebastien.hutter@univ-amu.fr (S.H.); nadine.azas@univ-amu.fr (N.A.)
3 Service Central de la Qualité et de l'Information Pharmaceutiques, Hôpital de la Conception, AP-HM, 147 Boulevard Baille, 13005 Marseille, France
4 Wellcome Centre for Anti-Infectives Research, School of Life Sciences, University of Dundee, Dow Street, Dundee DD1 5EH, UK; s.z.carvalho@dundee.ac.uk (S.C.); s.wyllie@dundee.ac.uk (S.W.)
5 Laboratoire de Synthèse Organique et Hétérocyclique Sélective-Evaluation D'activité Biologique, LR17ES01, Faculté des Sciences de Tunis, Université de Tunis El Manar, Campus Universitaire, Tunis 2092, Tunisia; medlotfi.efrit@fst.utm.tn
6 Normandie Université, UNICAEN, CERMN, DruiD Platform, Boulevard Becquerel, 14000 Caen, France; dimitri.fayolle@unicaen.fr (D.F.); marc.since@unicaen.fr (M.S.)
7 CNRS, Département de Pharmacochimie Moléculaire UMR 5063, University Grenoble Alpes, 38041 Grenoble, France; pierre.verhaeghe@univ-grenoble-alpes.fr
8 LCC-CNRS, UPR8241, Laboratoire de Chimie de Coordination, Université de Toulouse, CNRS, UPS, 31400 Toulouse, France
9 Chemistry Department, Faculty of Science, Assiut University, Assiut 71516, Egypt
10 Faculty of Pharmacy, Sphinx University, Regional Road, New Assiut 71515, Egypt
* Correspondence: nicolas.primas@univ-amu.fr (N.P.); elkashef@aun.edu.eg (H.E.-K.)

## Supplementary materials

## S1. Organic synthesis and product characterization of the known compounds used in our study

### 1. *(Z)-5-Arylidenethiazolidine-2,4-diones* **2-6**. *General procedure.*

A mixture of equimolar amounts of thiazolidine-2,4-dione (**1**) (10 mmol, 1.17 g) and the aromatic aldehyde, benzaldehyde (1.07 g), 2-nitrobenzaldehyde (1.52 g), 3-nitro benzaldehyde (1.52 g), 4-nitrobenzaldehyde (1.52 g), 3,4,5-trimethoxybenzaldehyde (1.98 g) in anhydrous toluene (50 mL) was heated under reflux in the presence of catalytic amount of piperidinium acetate in a flask equipped with a Dean-Stark apparatus. The reaction mixture was heated under reflux for 15 h. After cooling, the solid product obtained was filtered, washed with little toluene, dried and recrystallized.

*1.1. (Z)-5-Benzylidenethiazolidine-2,4-dione* **(2)**. Small white crystals from ethanol-dioxane, yield (82 %), mp 236-38 °C [lit.[1], mp 240-243°C]. **IR** (ν $cm^{-1}$): 3374 (N-H), 3141 (C-H arom.), 3030 (CH arom.), 2785 (C-H aliph.), 1737 (C=O), 1698 (C=O), 1608, 1595, 1495 (C=C arom.). **$^1$H-NMR** (400 MHz, DMSO-$d_6$) δ (ppm): 12.62 (br s, 1H, NH), 7.79 (s, 1H, -CH=), 7.61-7.59 (m, 2H, Ph-H), 7.55-7.46 (m, 3H,

Ph-H).[13]**C-NMR** (100 MHz, DMSO) δ (ppm): 168.34 (C=O), 167.77 (C=O), 133.50 (-CH=), 132.25 (C), 130.88 (CH), 130.47 (2CH), 129.77 (2CH), 124.00 (C). Anal. calcd. for $C_{10}H_7NO_2S$ (205.23): C, 58.52; H, 3.44; N, 6.82; S, 15.62. Found: C, 58.61; H, 3.50; N, 6.89; S, 15.58 %.

*1.2. (Z)-5-(2-Nitrobenzylidene)thiazolidine-2,4-dione (**3**).* Pale brown crystals from acetic acid, yield 75%, mp 195-57 ºC [lit.[2], mp 200-202 ºC]. **IR** (ν $cm^{-1}$): 3148 (N-H), 3054 (CH arom.), 2857 (CH aliph.), 2766 (C-H aliph.) 1737 (C=O), 1716 (C=O), 1610, 1572, 1519. [1]**H-NMR** (400 MHz, DMSO) δ (ppm): 12.77 (br s, 1H, NH), 8.21 (d, *J* = 8.3 Hz, 1H, Ar-H), 8.01 (s, 1H, -CH=), 7.89 (t, *J* = 7.6 Hz, 1H, Ar-H), 7.73 (t, *J* = 8.4 Hz, 2H, Ar-H). [13]**C-NMR** (100 MHz, DMSO) δ (ppm): 168.09 (C=O), 167.08 (C=O), 148.37 (C), 134.98 (-CH=), 131.49 (CH), 129.71 (CH), 129.38 (CH), 128.95 (C), 128.71 (CH), 125.90 (C). Anal. calcd. For $C_{10}H_6N_2O_4S$ (250.23): C, 48.00; H, 2.42; N, 11.20; S, 12.81. Found: C, 48.09; H, 2.31; N, 11.11; S, 12.70 %.

*1.3. (Z)-5-(3-Nitrobenzylidene)thiazolidine-2,4-dione (**4**).* Small canary-yellow crystals from ethanol, yield (79 %), mp 207-209 ºC [lit. [2], mp 208-210 ºC]. **IR** (ν $cm^{-1}$): 3158 (N-H), 3035 (CH arom.), 2767 (CH aliph.), 1745 (C=O), 1694 (C=O), 1604, 1510, 1529. [1]**H-NMR** (400 MHz, DMSO) δ (ppm): 12.75 (br s, 1H, NH), 8.39 (t, *J* = 1.7 Hz, 1H, Ar-H), 8.26 (dd, *J* = 8.2, 1.5 Hz, 1H, Ar-H), 7.97 (d, *J* = 7.9 Hz, 1H, Ar-H), 7.89 (s, 1H, -CH=), 7.79 (t, *J* = 8.0 Hz, 1H, Ar-H).[13]**C-NMR** (100 MHz, DMSO) δ (ppm): 167.71 (C=O), 167.47 (C=O), 148.64 (C), 135.88 (=CH-), 135.17 (C), 131.30 (CH), 129.77 (CH), 127.04 (C), 124.86 (CH), 124.73 (CH). Anal. calcd. For $C_{10}H_6N_2O_4S$ (250.23): C, 48.00; H, 2.42; N, 11.20; S, 12.81. Found: C, 48.13; H, 2.50; N, 11.10; S, 12.85 %.

*1.4. (Z)-5-(4-Nitrobenzylidene)thiazolidine-2,4-dione (**5**).* Small yellow crystals from ethanol-dioxane, yield (75%), mp 267-269 ºC [lit. [2], mp >250 ºC]. **IR** (ν $cm^{-1}$): 3197 (N-H), 3119 (C-H arom), 3048 (CH arom.), 2734 (C-H aliph.), 1753 (C=O), 1676 (C=O), 1609, 1593, 1533 (C=C arom.). [1]**H-NMR** (400 MHz, DMSO) δ (ppm): 12.85 (br s, 1H, NH), 8.37 (d, *J* = 8.8 Hz, 2H, Ar-H), 7.92 (s, 1H, -CH=), 7.88 (d, *J* = 8.8 Hz, 2H, Ar-H). [13]**C-NMR** (100 MHz, DMSO) δ (ppm): 167.82 (C=O), 167.47 (C=O), 147.89 (C), 139.81 (=CH-), 131.36 (2CH), 129.53 (C), 128.46 (C), 124.70 (2CH). Anal. calcd. For $C_{10}H_6N_2O_4S$ (250.23): C, 48.00; H, 2.42; N, 11.20; S, 12.81. Found: C, 48.09; H, 2.40; N, 11.29; S, 12.78 %.

*1.5. (Z)-5-(3,4,5-Trimethoxybenzylidene)thiazolidine-2,4-dione* (**6**). Yellow crystals from cyclohexane, yield (90%), mp 185–187 °C [3]. **IR** (ν $cm^{-1}$): 3176 (NH), 3020 (CH arom.), 2992 (CH aliph.), 2950 (CH aliph.), 2836 (CH aliph.), 1748 (C=O), 1698 (C=O), 1606, 1576, 1506, and 1451 (C=C arom.).[1]**H NMR** (300 MHz, $DMSO_{-d6}$): *δ* 3.72 (s, 3H, $OCH_3$), 3.82 (s, 6H, 2 $OCH_3$), 6.91 (s, 2H, Ar-H), 7.25 (s, 1H, -CH=), 12.59 (s, 1H, NH);[13]**C-NMR** (75 MHz, $DMSO_{-d6}$): *δ* 56.4 (2 OCH3), 60.6 (OCH3), 107.9 (2CH), 122.9 (C), 128.9 (C), 132.5 (C), 139.8 (CH), 153.6 (2C), 167.7 (C=O)**,** 168.3 (C=O). Anal. calcd. for $C_{13}H_{13}NO_5S$ (295.31): C, 52.87; H,4.44; N, 4.74. Found: C, 52.90; H, 4.19; N, 4.66%.

2. *Potassium (Z)-5-(3,4,5-trimethoxybenzylidene)thiazolidine-2,4-dion-3-ide* (**11**). Fluffy white needles, yield (85%), mp 250–255 °C (charring) [3]. **IR** (ν $cm^{-1}$): 3026

(CH arom.), 2998 (CH aliph.), 2940 (CH aliph.), 2835 (CH aliph.), 1732 (C=O), 1682 (C=O), 1607, 1573,1500, 1448 and 1430 (C=C arom.). $^{1}$H-NMR (300 MHz, DMSO-*d*6) $\delta$(ppm): 3.68 (s, 3H, $OCH_3$), 3.80 (s, 6H, 2 $OCH_3$), 6.86 (s, 2H, ArH), 7.25 (s, 1H, -CH=). **$^{13}$C-NMR** (75 MHz, DMSO-*d*6) $\delta$ (ppm): 56.2 (2 $OCH_3$), 60.5 ($OCH_3$), 106.8 (2CH), 122.8 (C), 132.0 (C), 136.0 (C), 137.7 (CH), 153.3 (2C), 176.0 (C=O), 183.3 (C=O). Anal. calcd. for $C_{13}H_{12}KNO_5S$ (333.40): C, 46.83; H,3.63; N, 4.20. Found: C, 46.58; H, 3.50; N, 4.00%.

3. *Ethyl (Z)-2-(5-benzylidenethiazolidine-2,4-dion-3-yl)acetate (**12a**).* Fine white crystals from ethanol-dioxane, yield (79%), mp 87-88 °C [lit. [4], mp 75-77°C]. **IR** (ν cm$^{-1}$): 3083 (C-H arom.), 2993 (C-H aliph.), 2955 (C-H aliph.), 1750 (C=O), 1739 (C=O), 1694 (C=O), 1606. 1568. 1524. **$^{1}$H-NMR** (400 MHz, DMSO) δ (ppm): 8.02 (s, 1H, -CH=), 7.67-7.65 (m, 2H, Ar-H), 7.59-7.51 (m, 3H, Ar-H), 4.51 (s, 2H, $CH_2$), 4.18 (q, *J* = 7.1 Hz, 2H, $CH_3\underline{CH_2}$), 1.22 (t, *J* = 7.1 Hz, 3H $\underline{CH_3}$-$CH_2$). **$^{13}$C-NMR** (100 MHz, DMSO) δ (ppm): 167.33 (C=O), 167.14 (C=O), 165.39 (C=O), 134.61 (-CH=), 133.20 (C), 131.41 (CH), 130.74 (2CH), 129.90 (2CH), 120.99 (C), 62.13 (O-$CH_2$), 42.70 (CO$\underline{CH_2}$), 14.41 ($CH_3$). Anal. Calcd. for $C_{14}H_{13}NO_4S$ (291.32): C, 57.72; H, 4.50; N, 4.81; S, 11.01. Found: C, 57.58; H, 4.41; N, 4.67; S, 11.15 %.

4. *Ethyl ((Z)-5-(3,4,5-trimethoxybenzylidene)thiazolidine-2,4-dion-3-yl)acetate (**12e**).* White crystals from ethanol, yield (69%), mp 145-147 ºC [3]. **IR** (ν cm$^{-1}$): 3018 (CH arom.), 2999 (CH aliph.), 2942 (CH aliph.), 2842 (CH aliph.), 1760 (C=O), 1739 (C=O),1689 (C=O), 1602, 1575, 1504 and 1469 (C=C arom.). **$^{1}$H- NMR** (300 MHz, DMSO-*d*6) $\delta$ (ppm): 1.22 (t, 3H, *J* = 7.1 Hz, $OCH_2\underline{CH_3}$), 3.74 (s, 3H, $OCH_3$), 3.84 (s, 6H, 2 $OCH_3$), 4.18 (q, 2H, *J* = 7.1 Hz, O$\underline{CH_2}CH_3$), 4.50 (s, 2H, $CH_2$), 6.98 (s, 2H, ArH), 7.95 (s, 1H, -CH=). **$^{13}$C-NMR** (75 MHz, DMSO-*d*6) $\delta$ (ppm): 14.4 ($CH_3$), 42.7 (-$NCH_2$), 56.5 (2 OCH3), 60.7 ($OCH_3$), 62.1 ($OCH_2$), 108.2 (2 CH), 119.9 (C), 128.7(C), 134.8 (C), 140.3 (CH), 153.7 (2C), 165.3 (C=O), 167.1 (C=O), 167.3 (C=O). Anal. calcd. for $C_{17}H_{19}NO_7S$ (381.40): C, 53.54; H, 5.02; N, 3.67. Found: 53.38; H, 5.15; N, 3.47%.

5. (Z)-5-(3,4,5-Trimethoxybenzylidene)-3-(2-oxopropyl)thiazolidine-2,4-dione (**13e**). Yellow crystals from ethanol, yield (85%), mp 139-140 ºC [3]. **IR** (ν cm$^{-1}$): 3026 (CH arom.), 2939 (CH aliph.), 2839 (CH aliph.), 1748 (C=O), 1726 (C=O), 1691 (C=O), 1604, 1576, 1506 and 1456 (C]C arom.). **$^{1}$H-NMR** (300 MHz, DMSO-*d*6) $\delta$ (ppm): 2.25 (s, 3H, $CH_3$), 3.74 (s, 3H, $OCH_3$) 3.84 (s, 6H, 2 $OCH_3$), 4.67 (s, 2H, $CH_2$), 6.98 (s, 2H, ArH), 7.92 (s, 1H, -CH=).**$^{13}$C-NMR** (75 MHz, DMSO-*d*6) $\delta$ (ppm): 27.5 ($CH_3$), 50.8 (-$NCH_2$), 56.3 (2 $OCH_3$), 60.7 ($OCH_3$), 108.2 (2 CH), 120.3 (C), 128.8 (C), 134.4 (C), 140.1 (CH), 153.7 (2 C), 165.5 (C=O), 167.3 (C=O), 200.8 (C=O). Anal. calcd. for $C_{16}H_{17}NO_6S$ (351.37): C, 54.69; H, 4.88; N, 3.99. Found: C, 54.48; H, 4.97; N, 3.71%.

6. ((Z)-5-(3,4,5-Trimethoxybenzylidene)thiazolidine-2,4-dion-3-yl) acetonitrile (**14e**). Yellow needles (recrystallization from ethanol), yield (87%), mp 173-175 ºC [3]. **IR** (ν cm$^{-1}$): 3026 (CH arom.), 2998 (CH aliph.), 2836 (CH aliph.), 2222 (CN), 1732 (C=O), 1681 (C=O), 1608, 1573 and 1448 (C=C arom.). **$^{1}$H-NMR** (300 MHz, $CDCl_3$) $\delta$ (ppm): 3.93 (s, 9H, 3 $OCH_3$), 4.63 (s, 2H, $CH_2$), 6.75 (s, 2H, ArH), 7.92 (s, 1H, -

CH=).[13]**C-NMR** (75 MHz, $CDCl_3$) *δ* (ppm): 28.2 ($CH_2$), 56.3 (2 $OCH_3$), 61.1 ($OCH_3$), 109.5 (2 CH), 112.8 (CN), 118.5 (C), 127.9 (C), 136.3 (C), 141.0 (CH), 153.7 (2 C), 164.1 (C=O), 166.1 (C=O). Anal. calcd. for $C_{15}H_{14}N_2O_5S$ (334.35): C, 53.89; H, 4.22; N, 8.38. Found: C, 53.72; H, 4.33; N, 8.18%.

*7. (Z)-2-(5-(3,4,5-Trimethoxybenzylidene)thiazolidine-2,4-dion-3-yl)acetamide (**15e**).* White crystals (recrystallization from ethanol: dioxane mixture (1:3), yield (83%), mp 274-275 ºC (decomposition) [3]. **IR** (ν $cm^{-1}$): 3384, 3304 and 3264 ($NH_2$) 3026 (CH arom), 2974 (CH aliph.), 2839 (CH aliph.), 1740 (C=O), 1682 (C=O), 1604, 1579, 1508 and 1448 (C=C arom.). [1]**H- NMR** (300 MHz, DMSO-*d6*), *δ* (ppm): 3.70 (s, 3H, $OCH_3$), 3.85 (s, 6H, 2 $OCH_3$), 4.24 (s, 2H, $CH_2$), 6.97 (s, 2H, ArH), 7.32 (s, 1H, NH), 7.73 (s, 1H, NH), 7.92 (s, 1H, -H=).[13]**C-NMR** (75 MHz, DMSO-*d*6) *δ* (ppm): 43.8 ($NCH_2$), 56.5 (2 $OCH_3$), 60.7 ($OCH_3$), 108.1 (2 CH), 120.6 (C), 128.9 (C), 133.9 (C), 140.1 (CH), 153.7 (2C), 165.7 (C=O), 167.3 (C=O), 167.5 (C=O). Anal. calcd. for $C_{15}H_{16}N_2O_6S$ (352.36): C, 51.13; H, 4.58; N, 7.95. Found: C, 50.89; H, 4.60; N, 7.78%.

*8. (Z)-5-Benzylidene-3-(2-oxo-2-phenylethyl)thiazolidine-2,4-dione (**16a**).* Off-white crystals from ethanol-dioxane, yield (83%), mp 182-183 ºC [lit. [5], mp 177-178 ºC]. **IR** (ν $cm^{-1}$): 3063 (C-H arom), 3016 (C-H arom.), 2977 (C-H aliph.), 2973 (C-H aliph.), 1739 (C=O), 1693 (C=O), 1678 (C=O), 1604, 1492, 1484. [1]**H-NMR** (400 MHz, DMSO) δ (ppm): 8.11-8.09 (m, 2H, Ph-H), 8.03 (s, 1H, -CH=), 7.76-7.54 (m, 8H, Ph-H), 5.35 (s, 2H, $CH_2$). [13]**C-NMR** (100 MHz, DMSO) δ (ppm): 191.79 (C=O), 167.50 (C=O), 165.70 (C=O), 134.69 (C), 134.40 (CH), 134.24 (-CH=), 133.30 (C), 131.37 (CH), 130.74 (2CH), 129.98 (2CH), 129.72 (2CH), 128.80 (2CH), 121.30 (C), 48.41 ($CH_2$). Anal. calcd. for $C_{18}H_{13}NO_3S$ (323.37): C, 66.86; H, 4.05; N, 4.33; S, 9.91. Found: C, 66.80; H, 4.15; N, 4.40; S, 9.99 %.

9. General procedure for the synthesis of N-aryl-2-chloroacetamide **17-22**

To a stirred cold solution (ice bath temperature) of the aromatic amine (0.1 mol) in dry DMF (40 mL), TEA (0.1 mol) was added, followed by a dropwise addition of a cold solution of 2-chloroacetyl chloride (0.12 mol) in dry DMF (7 mL). Stirring was continued for 3h at this temperature and another 3h at room temperature. The reaction mixture was then poured on ice cold water under stirring. The solid product obtained was filtered, washed with water, dried and crystallized.

*9.1. 2-Chloro-N-phenylacetamide (**17**).* Lustrous white platelets from ethanol-water, yield (89%). mp 136-138 °C [lit.[6], mp 134-135 °C]. **IR** (ν $cm^{-1}$): 3267 (N-H), 3207 (N-H), 3145 (C-H arom.), 3097 (C-H arom.), 2974 (C-H aliph.) 1673(C=O). 1610, 1602, 1558. [1]**H-NMR** (400 MHz, DMSO) δ (ppm): 10.29 (s, 1H, NH), 7.60 (m, 2H, Ph-H), 7.34 (m, 2H, Ph-H), 7.09 (m,1H, Ph-H), 4.26 (s, 2H, $CH_2$). [13]**C-NMR** (100 MHz, DMSO) δ (ppm): 165.08 (C=O), 138.94 (C), 129.32 (2CH), 124.31 (CH), 119.82 (2CH), 44.05 ($CH_2$). Anal. Calcd. for $C_8H_8ClNO$ (169.61): C, 56.65; H, 4.75; N, 8.26. Found: C, 56.73; H, 4.82; N, 8.30 %.

*9.2. 2-Chloro-N-(4-methylphenyl)acetamide (**18**).* White crystals from ethanol, yield (85%). mp 164-166 °C [lit. [6], mp 162-164 °C]. **IR** (ν $cm^{-1}$): 3274 (N-H), 3206 (N-H), 3138 (CH arom.), 2951(CH aliph.), 1671(C=O). 1616, 1554, 1512. [1]**H-NMR** (400

MHz, DMSO) δ (ppm): 10.20 (s, 1H, NH), 7.48 (d, *J* = 8.4 Hz, 2H, Ar-H), 7.13 (d, *J* = 8.4 Hz, 2H, Ar-H), 4.23 (s, 2H, $CH_2$), 2.26 (s, 3H, $CH_3$).**$^{13}$C-NMR** (100 MHz, DMSO) δ (ppm): 164.83 (C=O), 136.44 (C), 133.29 (C), 129.68 (2CH), 119.83 (2CH), 44.04 ($CH_2$), 20.91 ($CH_3$). Anal. Calcd. for $C_9H_{10}ClNO$ (183.64): C, 58.87; H, 5.49; N, 7.63. Found: C, 58.94; H, 5.51; N, 7.69 %.

*9.3. 2-Chloro-N-(4-methoxylphenyl)acetamide (**19**).* White crystals from ethanol, yield (87%). mp 122-123 °C [lit. [6], mp 119-121 °C]. **IR** (ν $cm^{-1}$): 3295 (N-H), 3199 (C-H arom.), 3073 (C-H arom.), 2908 (C-H aliph.), 2835 (C-H aliph.), 1663 (C=O), 1603, 1548. 1517.**$^{1}$H-NMR** (400 MHz, DMSO) δ (ppm): 10.15 (s, 1H, NH), 7.51 (d, *J* = 9.1 Hz, 2H, Ar-H), 6.91 (d, *J* = 9.1 Hz, 2H, Ar-H), 4.22 (s, 2H, $CH_2$), 3.73 (s, 3H, $OCH_3$).**$^{13}$C-NMR** (100 MHz, DMSO) δ (ppm): 164.60 (C=O), 156.08 (C), 132.02 (C), 121.41 (2CH), 114.42 (2CH), 55.63 ($OCH_3$), 43.98 ($CH_2$). Anal. Calcd. For $C_9H_{10}ClNO_2$ (199.63): C, 54.15; H, 5.05; N, 7.02. Found: C, 54.36; H, 5.11; N, 7.13 %.

*9.4. 2-Chloro-N-(4-nitrophenyl)acetamide (**20**).* Yellow crystals from ethanol, yield (96%) mp 184-185 °C [lit. [6], mp 183 °C]. **IR (ν $cm^{-1}$)**: 3277 (N-H), 3228 (N-H), 3163 (C-H arom.), 2942 (C-H aliph.), 2830 (C-H aliph.), 1686 (C=O), 1624, 1598, 1568, 1406. **$^{1}$H-NMR** (400 MHz, DMSO) δ (ppm): 10.87 (s, 1H, NH), 8.22 (d, *J* = 9.1 Hz, 2H, Ar-H), 7.83 (d, *J* = 9.2 Hz, 2H, Ar-H), 4.33 (s, 2H, $CH_2$). **$^{13}$C-NMR** (100 MHz, DMSO) δ (ppm): 166.03 (C=O), 145.04 (C), 143.06 (C), 125.45 ($2CH_2$), 119.52 ($2CH_2$), 44.04 ($CH_2$). Anal. Calcd. For $C_8H_7ClN_2O_3$ (214.61): C, 44.77; H, 3.29; N, 13.05. Found: C, 44.61; H, 3.21; N, 13.16 %

*9.5. 2-Chloro-N-(4-chlorophenyl)acetamide (**21**).* White crystals from ethanol, yield (90%) mp 170-171 °C [lit. [6], mp 168-170 °C]. **IR (ν $cm^{-1}$)**: 3264 (N-H), 3199 (C-H arom.), 3083 (C-H arom.), 2952 (C-H aliph.), 1670 (C=O), 1614. 1597. 1551. **$^{1}$H-NMR** (400 MHz, DMSO) δ (ppm): 10.43 (s, 1H, NH), 7.62 (d, *J* = 8.9 Hz, 2H, Ar-H), 7.39 (d, *J* = 8.9 Hz, 2H, Ar-H), 4.26 (s, 2H, $CH_2$). **$^{13}$C-NMR** (100 MHz, DMSO) δ (ppm): 165.25 (C=O), 137.90 (C), 129.25 ($2CH_2$), 127.90 (C), 121.38 ($2CH_2$), 43.98 ($CH_2$). Anal. Calcd. for: $C_8H_7Cl_2NO$ (204.05): C, 47.09; H, 3.46; N, 6.86. Found: C, 47.20; H, 3.35; N, 6.80 %.

*9.6. 2-Chloro-N-(2-chlorophenyl)acetamide (**22**).* White crystals from ethanol, yield (90%) mp 67-68 °C [lit. [7], mp 71-73 °C]. **IR (ν $cm^{-1}$)**: 3269 (N-H), 3114 (CH arom.), 2944 (CH aliph.), 1671 (C=O), 1591, 1537, 1473.**$^{1}$H-NMR** (400 MHz, DMSO) δ (ppm): 9.87 (s, 1H, NH), 7.73 (dd, *J* = 8.0, 1.6 Hz, 1H, Ar-H), 7.52 (dd, *J* = 8.0, 1.6 Hz, 1H, Ar-H), 7.36 (td, *J* = 8.0, 1.6 Hz, 1H, Ar-H), 7.24 (td, *J* = 8.0, 1.6 Hz, 1H, Ar-H), 4.38 (s, 2H, $CH_2$). **$^{13}$C-NMR** (100 MHz, DMSO) δ (ppm): 165.65 (C=O), 134.67 (C), 130.06 (CH), 128.05 (CH), 127.33 (CH), 127.11 (C), 126.50 (CH), 43.50 ($CH_2$). Anal. Calcd. for: $C_8H_7Cl_2NO$ (204.05): C, 47.09; H, 3.46; N, 6.86. Found: C, 47.18; H, 3.35; N, 6.76 %.

*10. (Z)-2-(5-Benzylidenethiazolidine-2,4-dion-3-yl)-N-phenylacetamide (**23a**).* Off-white crystals from DMF-water, yield (94%), mp 259-260 °C [lit. [8], mp 262-265 °C]. **IR** (ν $cm^{-1}$): 3275 (N-H), 3135 (C-H arom.), 3065 (C-H arom.), 3020 (C-H

arom.), 1748 (C=O), 1697(C=O), 1663 (C=O), 1599, 1574, 1550. **$^{1}$H-NMR** (400 MHz, DMSO) δ (ppm): 10.40 (s, 1H, NH), 8.01 (s, 1H, -CH=), 7.68-7.66 (m, 2H, Ph-H), 7.58-7.50 (m, 5H, Ph-H), 7.33 (t, *J* = 7.9 Hz, 2H, Ph-H), 7.09 (t, *J* = 7.4 Hz, 1H, Ph-H), 4.53 (s, 2H, $CH_2$). **$^{13}$C-NMR** (100 MHz, DMSO) δ (ppm): 167.61 (C=O), 165.78 (C=O), 164.24 (C=O), 138.86 (C), 134.09 (CH), 133.33 (C), 131.30 (CH), 130.68 (2CH), 129.91 (2CH), 129.36 (2CH), 124.19 (C), 121.47 (CH), 119.63 (2CH), 44.52 ($CH_2$). Anal. Calcd. For $C_{18}H_{14}N_2O_3S$ (338.38): C, 63.89; H, 4.17; N, 8.28; S, 9.47. Found: C, 63.77; H, 4.23; N, 8.32; S, 9.40 %.

*11. (Z)-2-(5-Benzylidenethiazolidine-2,4-dion-3-yl)-N-(4-methoxyphenyl) acetamide* ***(25a)***. Fluffy white crystals from ethanol-dioxane, yield (87%), mp 254-256 °C [lit. [8], mp 262-264 °C]. **IR** (ν $cm^{-1}$): 3282 (N-H), 3134 (C-H arom.), 3065 (C-H arom.), 2933 (C-H aliph.), 2837(C-H aliph.),1746 (C=O), 1696 (C=O), 1659 (C=O), 1610, 1574, 1551.**$^{1}$H-NMR** (400 MHz, DMSO) δ (ppm): 10.26 (s, 1H, NH), 7.99 (s, 1H, -CH=), 7.66 (d, 2H, Ar-H), 7.58-7.48 (m, 5H, Ar-H), 6.90 (d, *J* = 8.6 Hz, 2H, Ar-H), 4.51 (s, 2H, $CH_2$), 3.72 (s, 3H, $OCH_3$). **$^{13}$C-NMR** (100 MHz, DMSO) δ (ppm): 167.60 (C=O), 165.79 (C=O), 163.72 (C=O), 155.96 (C), 134.03 (-CH=), 133.34 (C), 132.00 (C), 131.26 (C), 130.66 (2CH), 129.87 (2CH), 121.50 (CH), 121.20 (2CH), 114.44 (2CH), 55.62 ($OCH_3$), 44.42 ($CH_2$). Anal. Calcd. For $C_{19}H_{16}N_2O_4S$ (368.41): C, 61.94; H, 4.38; N, 7.60; S, 8.70. Found: C, 62.10; H, 4.37; N, 7.59; S, 8.72 %.

*12. (Z)-2-(5-Benzylidenethiazolidine-2,4-dion-3-yl)-N-(4-chlorophenyl) acetamide* ***(27a)***. Fluffy white crystals from DMF-water, yield (90%), mp 249-251 °C [lit. [8], mp 252-254 °C]. **IR** (ν $cm^{-1}$): 3269 (N-H), 3118 (C-H arom.), 3035 (C-H arom.), 3022 (C-H arom.) 2943 (C-H aliph.), 1748 (C=O), 1697 (C=O), 1662 (C=O), 1607, 1596, 1573. **$^{1}$H-NMR** (400 MHz, DMSO) δ (ppm): 10.55 (s, 1H, NH), 8.00 (s, 1H, -CH=), 7.68-7.66 (m, 2H, Ar-H), 7.60 (d, *J* = 9.0 Hz, 2H, Ar-H), 7.57-7.50 (m, 3H, Ar-H), 7.39 (d, *J* = 8.9 Hz, 2H, Ar-H), 4.54 (s, 2H, $CH_2$). **$^{13}$C-NMR** (100 MHz, DMSO) δ (ppm): 167.58 (C=O), 165.74 (C=O), 164.46 (C=O), 137.80 (C), 134.15 (-CH=), 133.31 (CH), 131.29 (C), 130.68 (2CH), 129.89 (2CH), 129.28 (2CH), 127.80 (C), 121.32 (C), 121.22 (2CH), 44.53 ($CH_2$). Anal. calcd. for: $C_{18}H_{13}ClN_2O_3S$ (372.82): C, 57.99; H, 3.51; N, 7.51; S, 8.60. Found: C, 58.05; H, 3.53; N, 7.56; S, 8.61 %.

## References


[1] Elkholy, N.; Abdelwaly, A.; Mohamed, K.; Amata, E.; Lombino, J.; Cosentino, G.; Intagliata, S.; Helal, M. A. Discovery of 3-(2-Aminoethyl)-Thiazolidine-2,4-Diones as a Novel Chemotype of Sigma-1 Receptor Ligands. *Chem. Biol. Drug Des.* **2022**, *100*, 25–40. https://doi.org/10.1111/cbdd.14047

[2] El-Kashef, H.; Badr, G.; Abo El-Maali, N.; Sayed, D.; Melnyk, P.; Lebegue, N.; Abd El-Khalek, R. Synthesis of a Novel Series of (*Z*)-3,5-Disubstituted Thiazolidine-2,4-Diones as Promising Anti-Breast Cancer Agents. *Bioorg. Chem.* **2020**, *96*, 103569. https://doi.org/10.1016/j.bioorg.2020.103569.

[3] Cortelazzo-Polisini, E.; Boisbrun, M.; Gansmüller, A. H.; Comoy, C. Photoisomerization of Arylidene Heterocycles: Toward the Formation of Fused Heterocyclic Quinolines. *J. Org. Chem.* **2022**, *87*, 9699–9713. https://doi.org/10.1021/acs.joc.2c00748.

[4] Bhat, B. A.; Ponnala, S.; Sahu, D. P.; Tiwari, P.; Tripathi, B. K.; Srivastava, A. K. Synthesis and Antihyperglycemic Activity Profiles of Novel Thiazolidinedione Derivatives. *Bioorg. Med. Chem.* **2004**, *12*, 5857–5864. https://doi.org/10.1016/j.bmc.2004.08.031.

[5] Omar, M. T.; Youssef, A. M. Conversion of 3-Aroylmethyl-5-Arylmethylene-2,4-Dioxo-1,3-Thiazolidines into 6-Aryl-4,5-Dihydro-1,2,4-Triazin-3(2h)-Ones. *Org. Prep. Proced. Int.* **1991**, *23*, 379–382. https://doi.org/10.1080/00304949109458215.

[6] Kim, S.-C.; Kwon, B.-M. A New Convenient Synthesis of Hydantoin Derivatives by the Phase-Transfer Method. *Synthesis* **1982**, *1982*, 795–796. https://doi.org/10.1055/s-1982-29951.

[7] Monforte, A.-M.; Ferro, S.; De Luca, L.; Lo Surdo, G.; Morreale, F.; Pannecouque, C.; Balzarini, J.; Chimirri, A. Design and Synthesis of $N_1$-Aryl-Benzimidazoles 2-Substituted as Novel HIV-1 Non-Nucleoside Reverse Transcriptase Inhibitors. *Bioorg. Med. Chem.* **2014**, *22*, 1459–1467. https://doi.org/10.1016/j.bmc.2013.12.045.

[8] Hassan, G. S.; Georgey, H. H.; Mohammed, E. Z.; Omar, F. A. Anti-Hepatitis-C Virus Activity and QSAR Study of Certain Thiazolidinone and Thiazolotriazine Derivatives as Potential NS5B Polymerase Inhibitors. *Eur. J. Med. Chem.* **2019**, *184*, 111747. https://doi.org/10.1016/j.ejmech.2019.111747

## S2. Proton and $^{13}$C-NMR spectra of all the synthesized compounds.

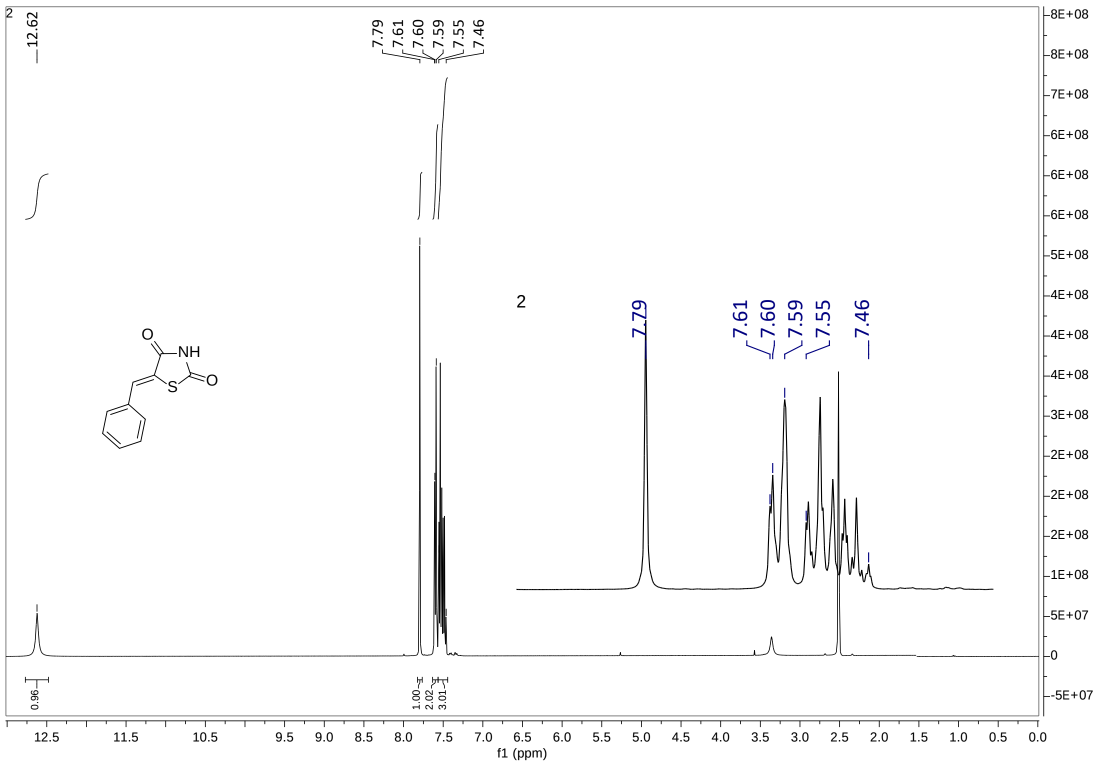


$^{1}$H-NMR spectrum of (*Z*)-5-benzylidenethiazolidine-2,4-dione (**2**)

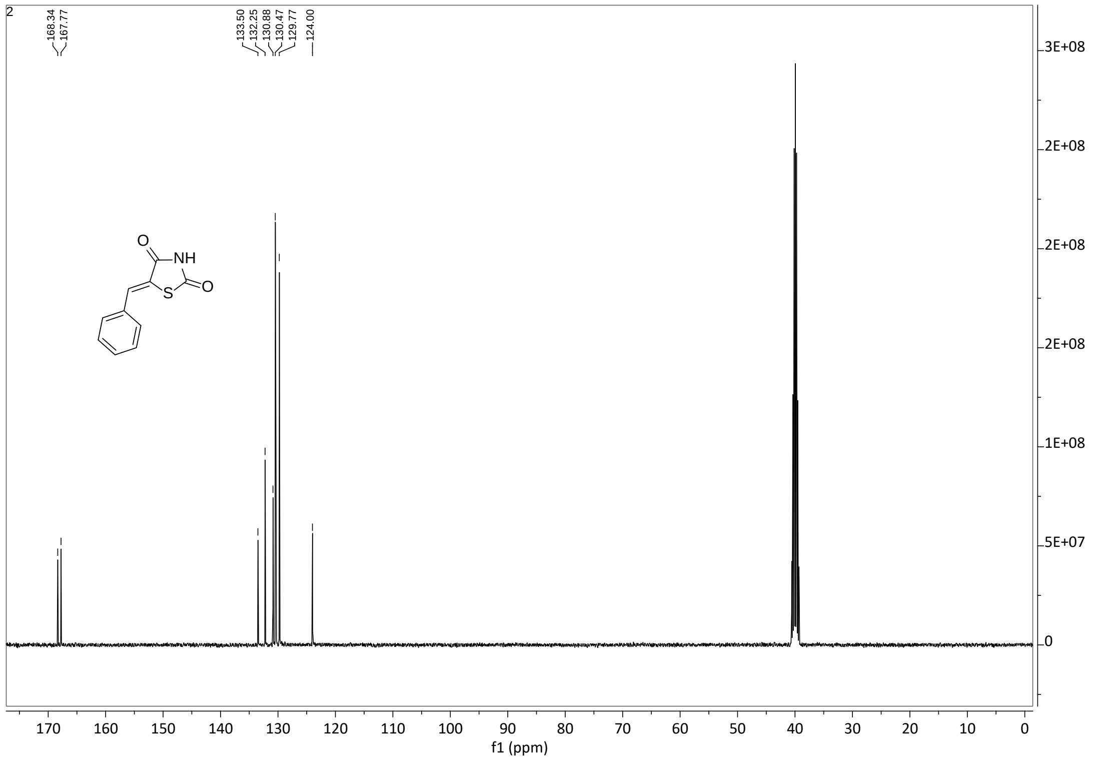


$^{13}$C-NMR spectrum of (*Z*)-5-benzylidenethiazolidine-2,4-dione (**2**)

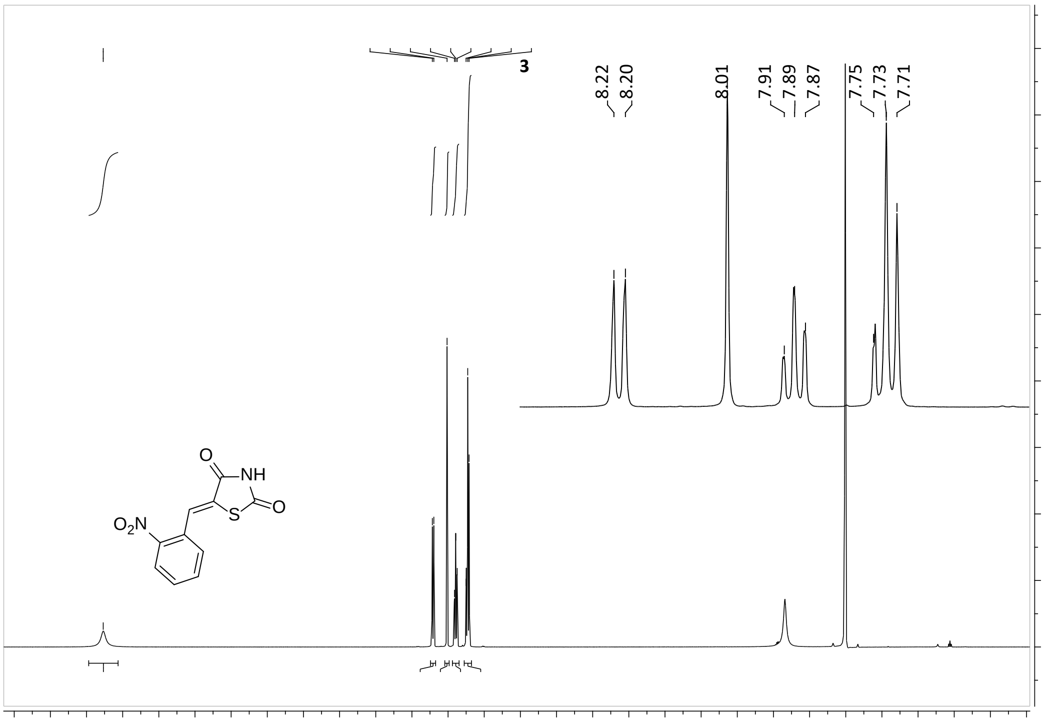


$^{1}$H-NMR spectrum of (*Z*)-(5-(2-nitrobenzylidene)thiazolidine-2,4-dione (**3**)

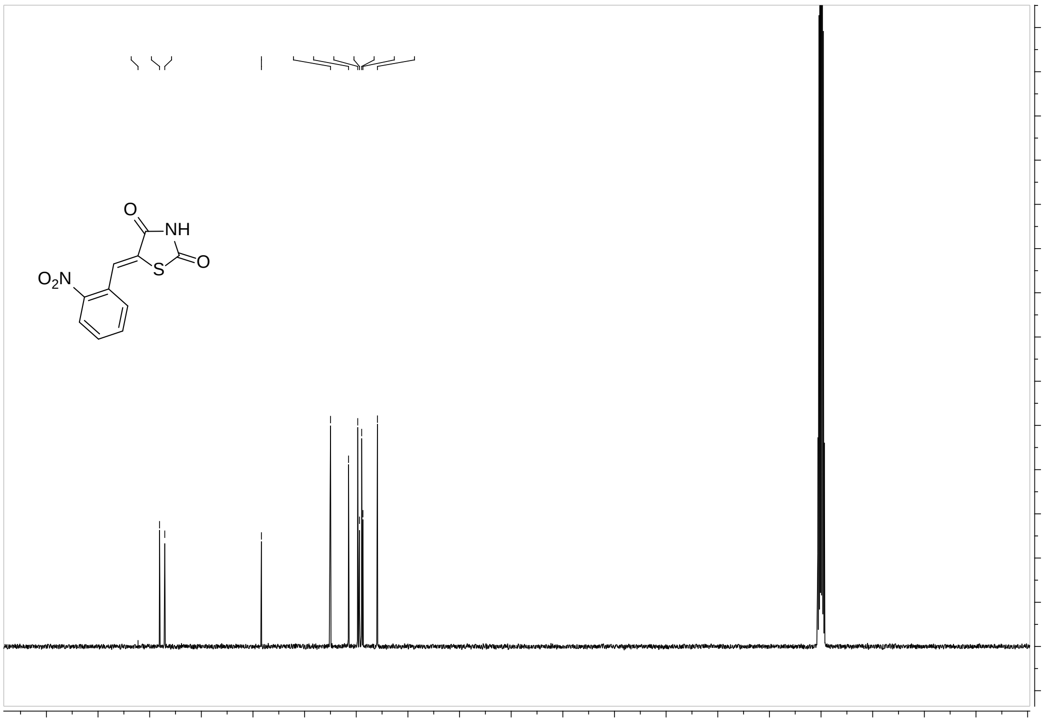


$^{13}$C-NMR spectrum of (*Z*)-(5-(2-nitrobenzylidene)thiazolidine-2,4-dione (**3**)

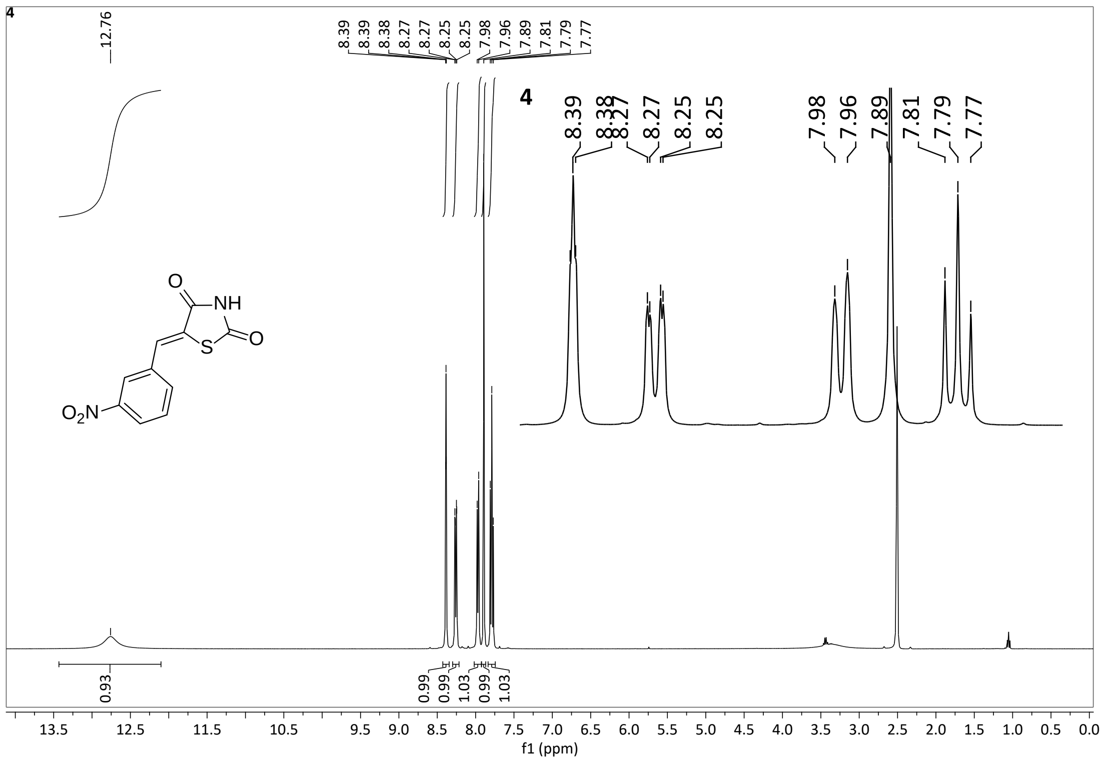

$^{1}$H-NMR spectrum of (*Z*)-(5-(3-nitrobenzylidene)thiazolidine-2,4-dione (4)

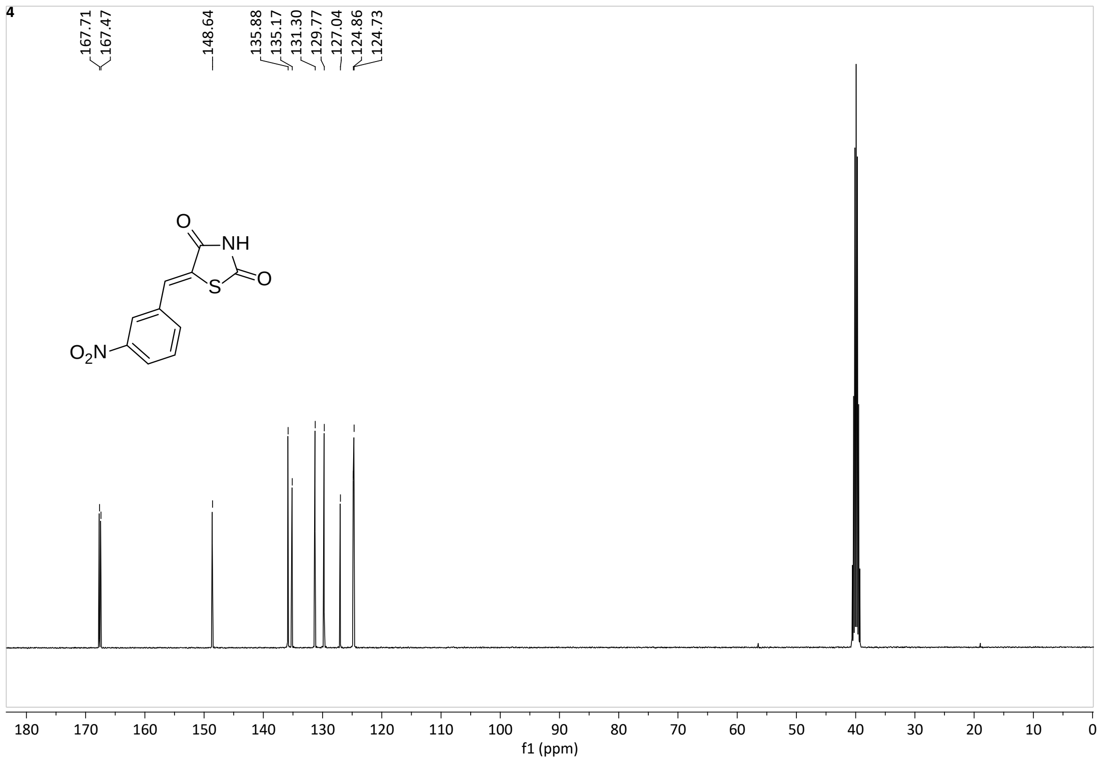

$^{13}$C-NMR spectrum of (*Z*)-(5-(3-nitrobenzylidene)thiazolidine-2,4-dione (4)

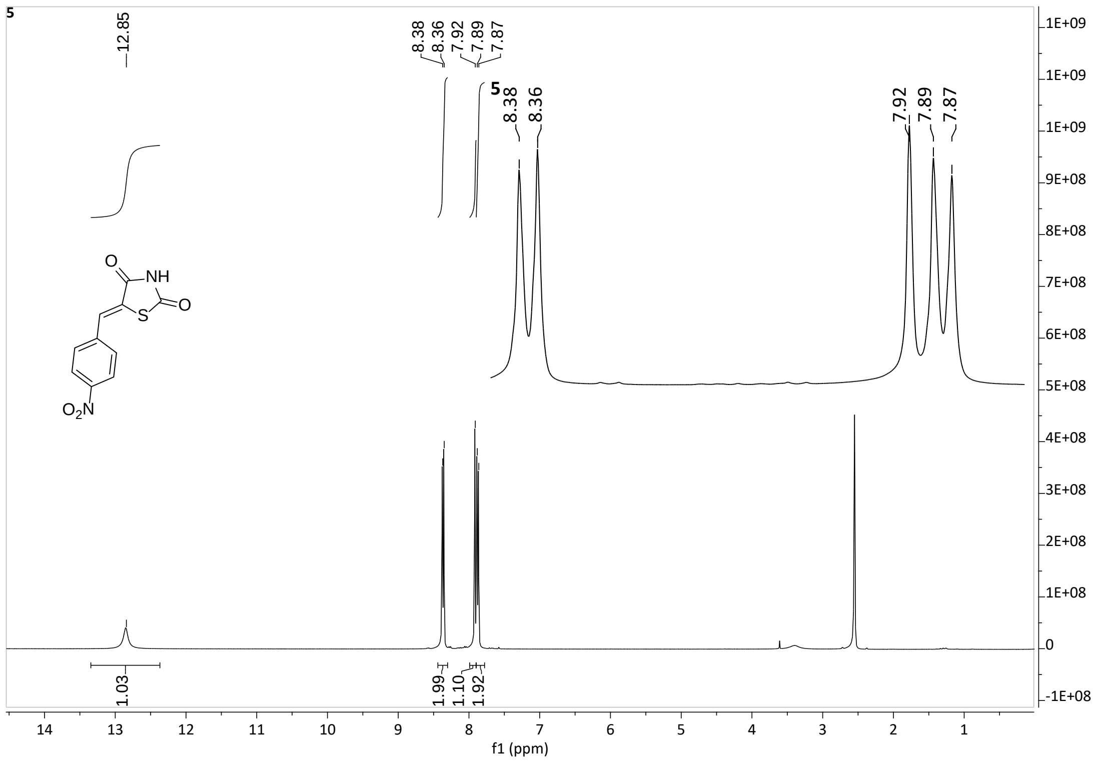


$^{1}$H-NMR spectrum of (*Z*)-(5-(4-nitrobenzylidene)thiazolidine-2,4-dione (**5**)

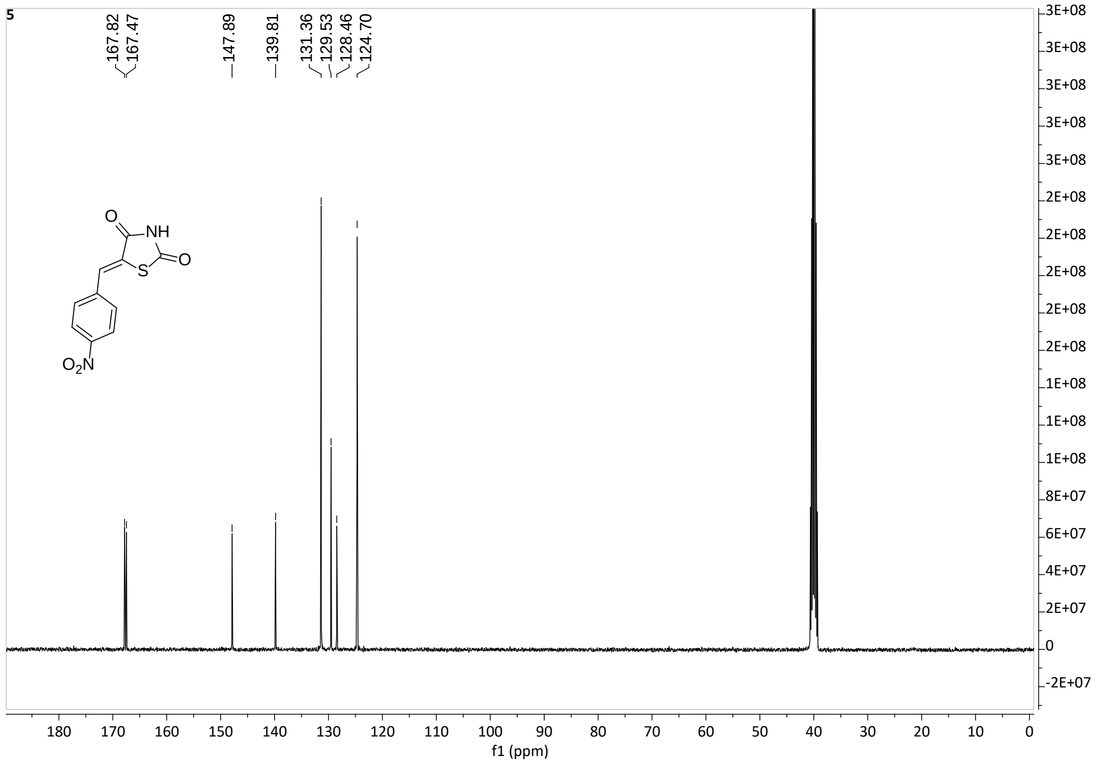


$^{13}$C-NMR spectrum of (*Z*)-(5-(4-nitrobenzylidene)thiazolidine-2,4-dione (**5**)

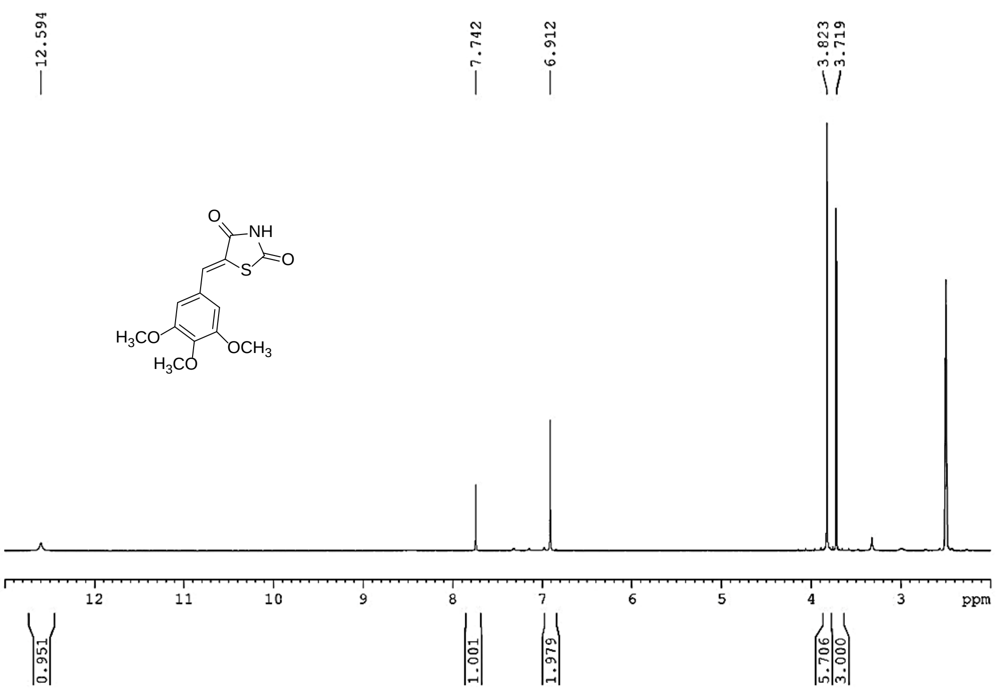


$^{1}$H-NMR spectrum of (*Z*)-(5-(3,4,5-trimethoxybenzylidene)thiazolidine-2,4-dione (**6**)

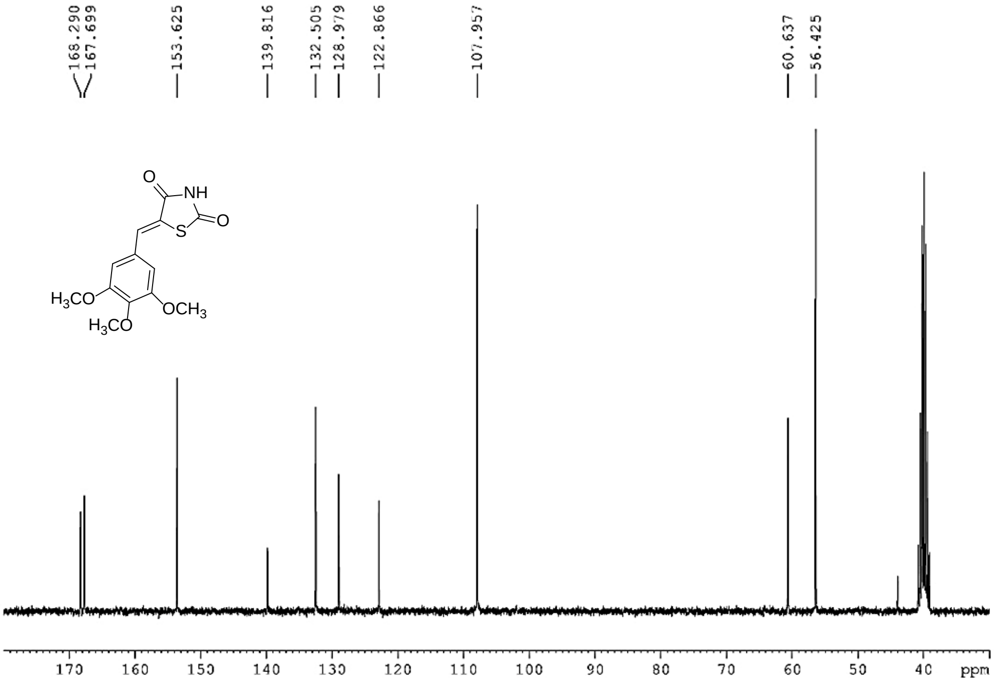


$^{13}$C-NMR spectrum of (*Z*)-(5-(3,4,5-trimethoxybenzylidene)thiazolidine-2,4-dione (**6**)

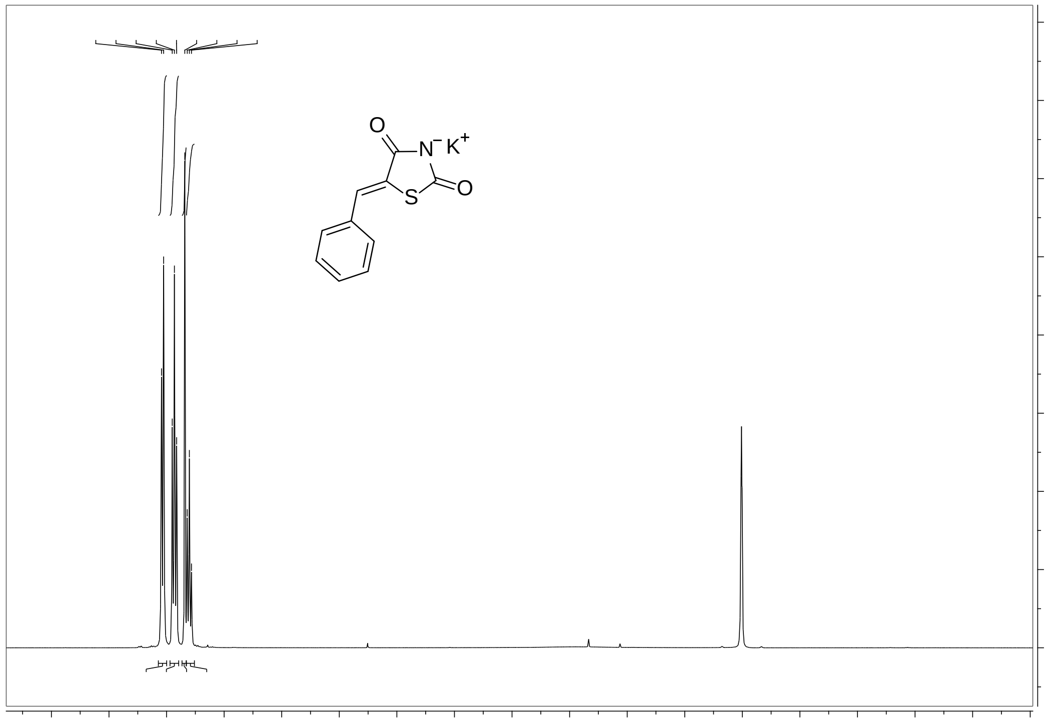


$^{1}$H-NMR spectrum of potassium (*Z*)-5-benzylidenethiazolidine-2,4-dion-3-ide (**7**)

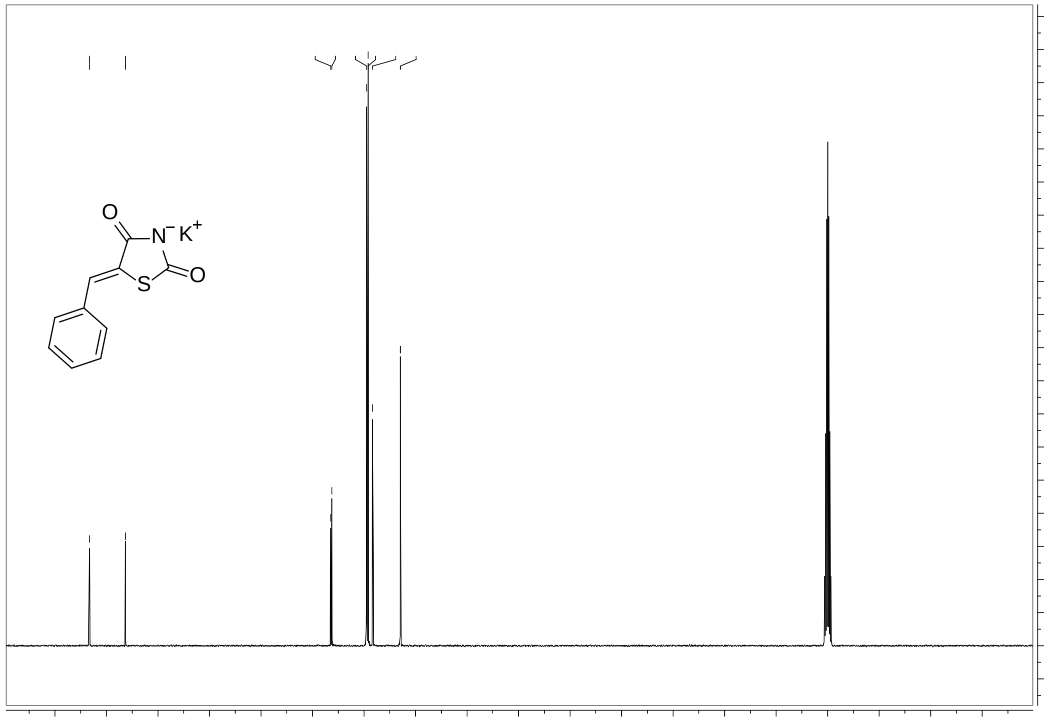


$^{13}$C-NMR spectrum of potassium (*Z*)-5-benzylidenethiazolidine-2,4-dion-3-ide (**7**)

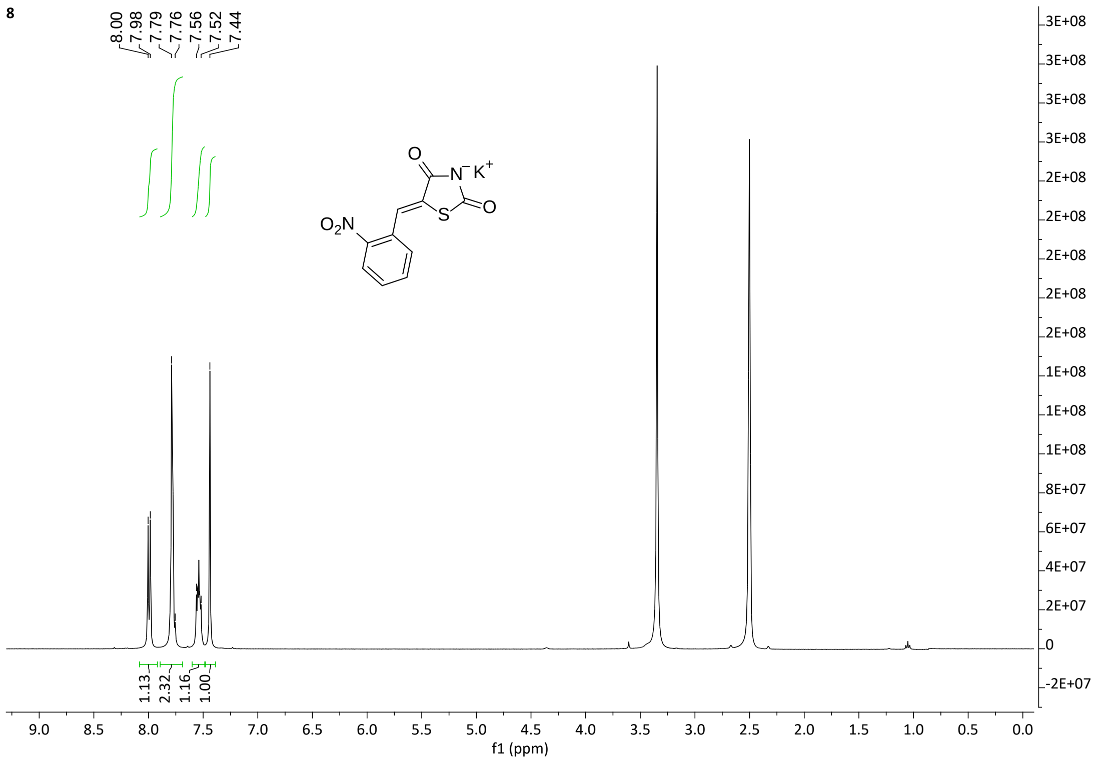


$^{1}$H-NMR spectrum of potassium (*Z*)-5-(2-nitrobenzylidene)thiazolidine-2,4-dion-3-ide (**8**)

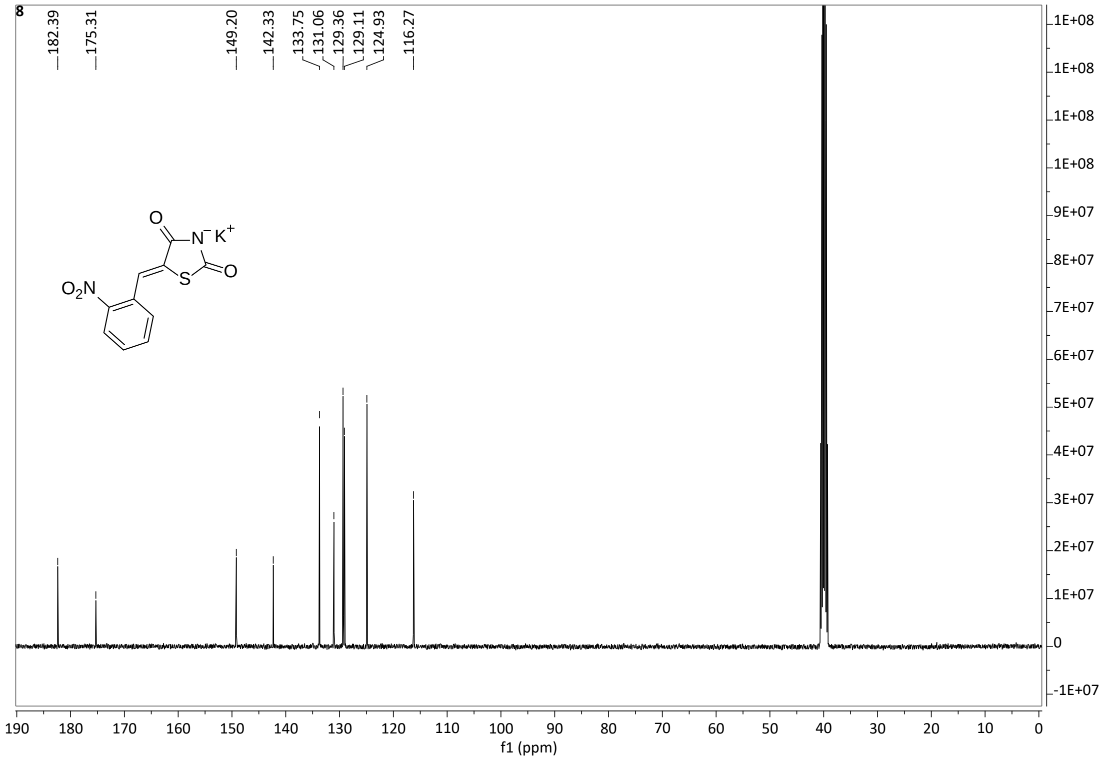


$^{13}$C-NMR spectrum of potassium (*Z*)-5-(2-nitrobenzylidene)thiazolidine-2,4-dion-3-ide (**8**)

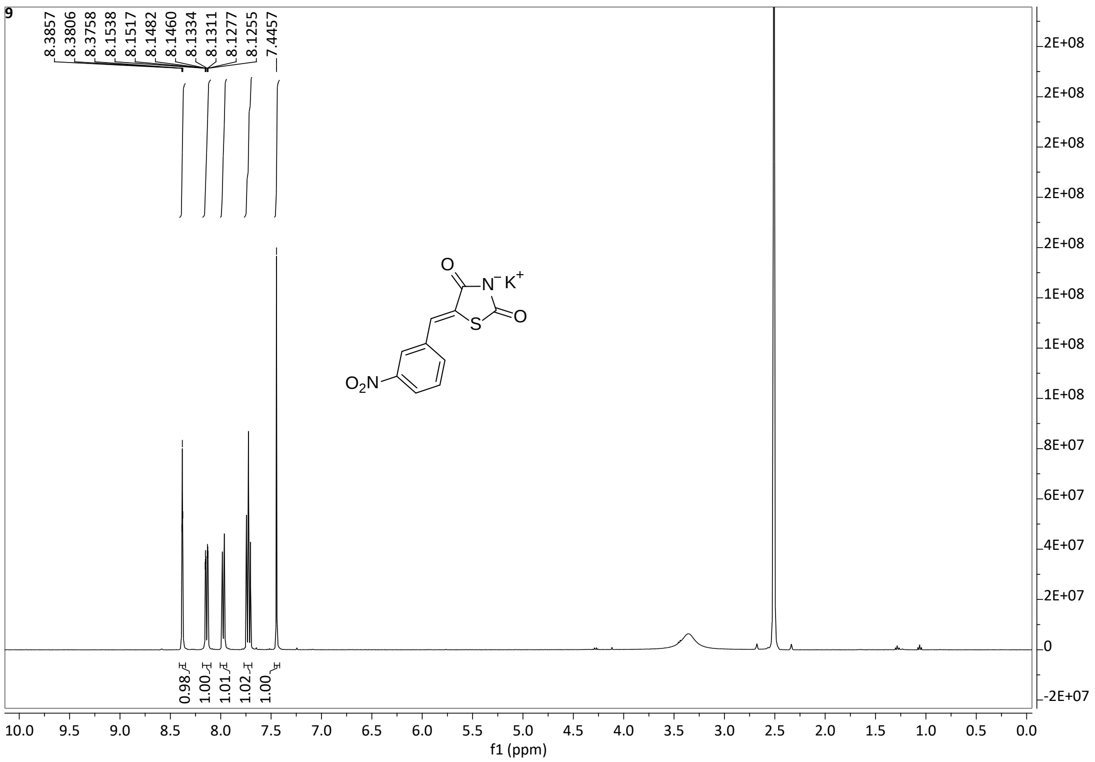


$^{1}$H-NMR spectrum of potassium (*Z*)-5-(3-nitrobenzylidene)thiazolidine-2,4-dion-3-ide (**9**)

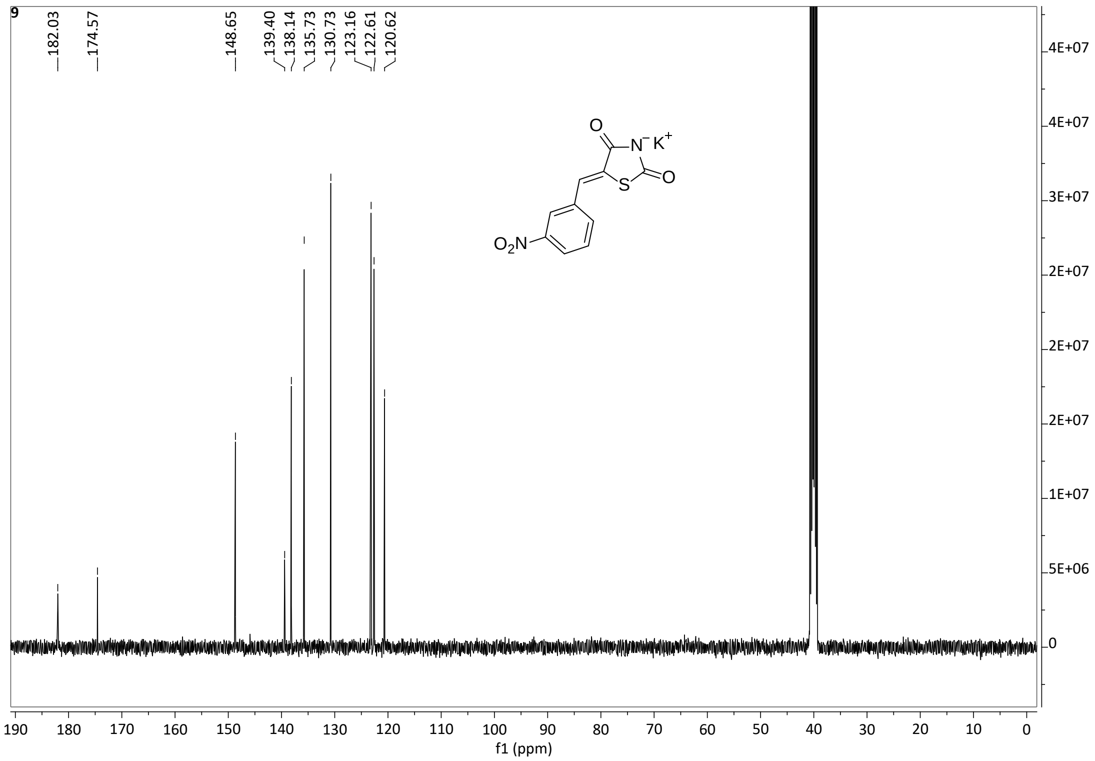


$^{13}$C-NMR spectrum of potassium (*Z*)-5-(3-nitrobenzylidene)thiazolidine-2,4-dion-3-ide (**9**)

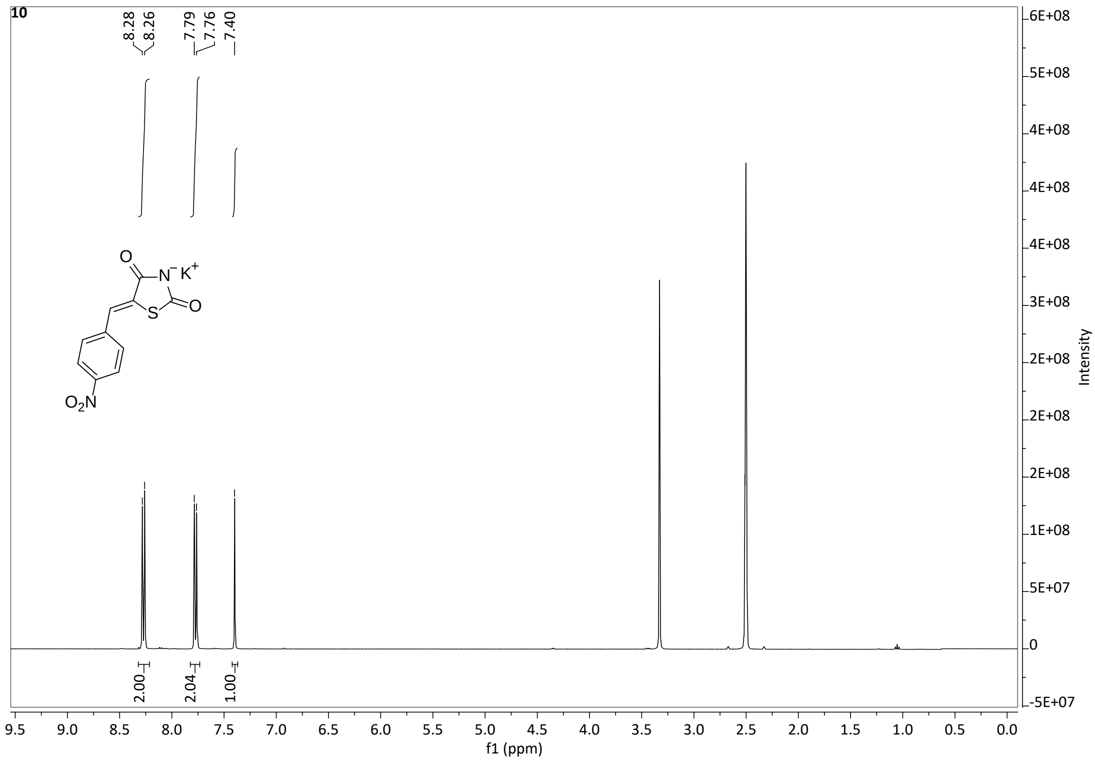


$^{1}$H-NMR spectrum of *potassium* (Z)-5-(4-nitrobenzylidene)thiazolidine-2,4-dion-3-ide (10)

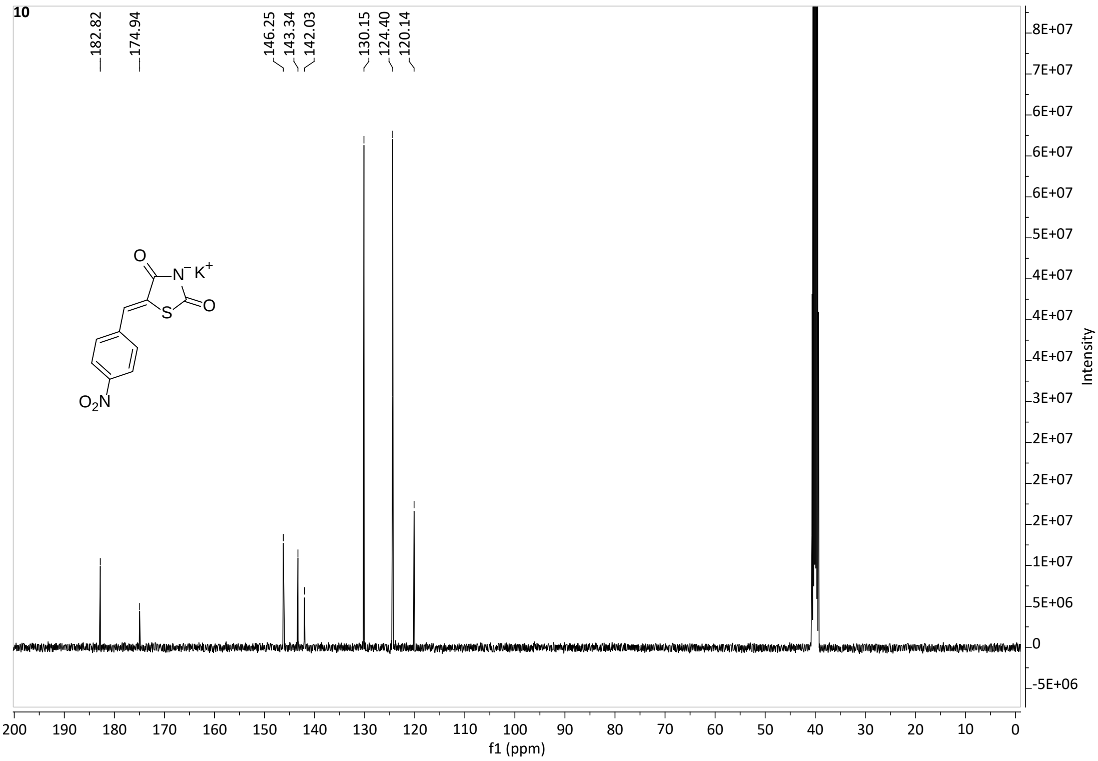


$^{13}$C-NMR spectrum of *potassium* (Z)-5-(4-nitrobenzylidene)thiazolidine-2,4-dion-3-ide (10)

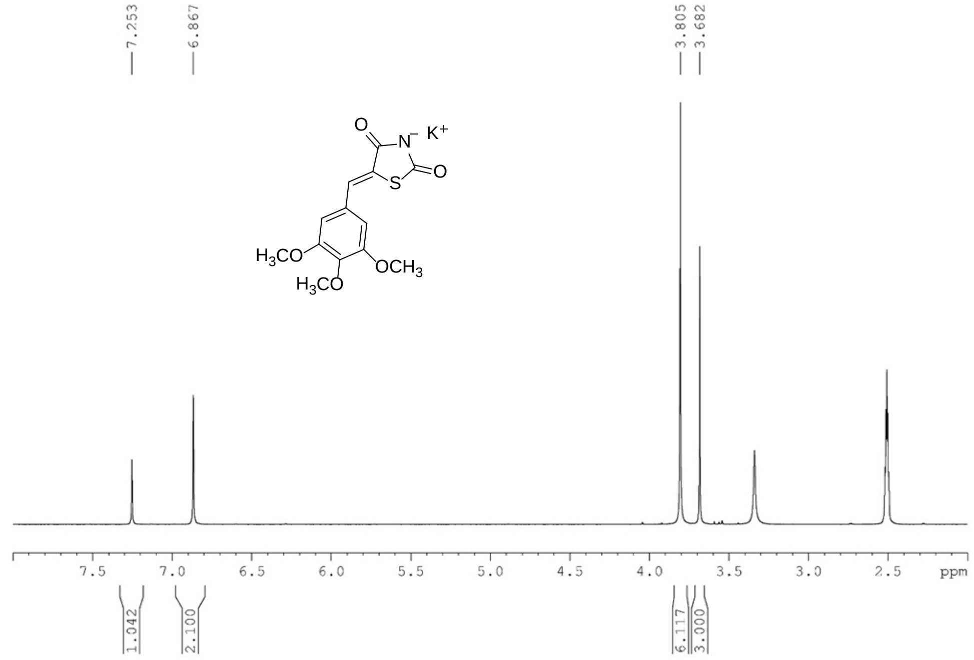


$^{1}$H-NMR spectrum of (*Z*)-5-(3,4,5-trimethoxybenzylidene)thiazolidine-2,4-dion-3-ide

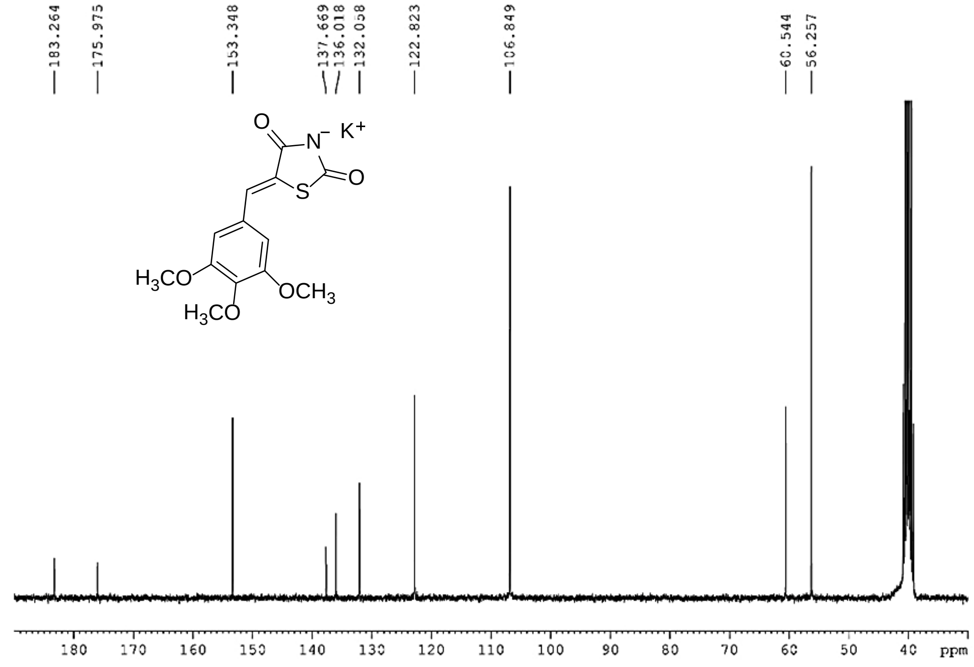


**(11)**

$^{13}$C-NMR spectrum of (*Z*)-5-(3,4,5-trimethoxybenzylidene)thiazolidine-2,4-dion-3-ide (**11**)

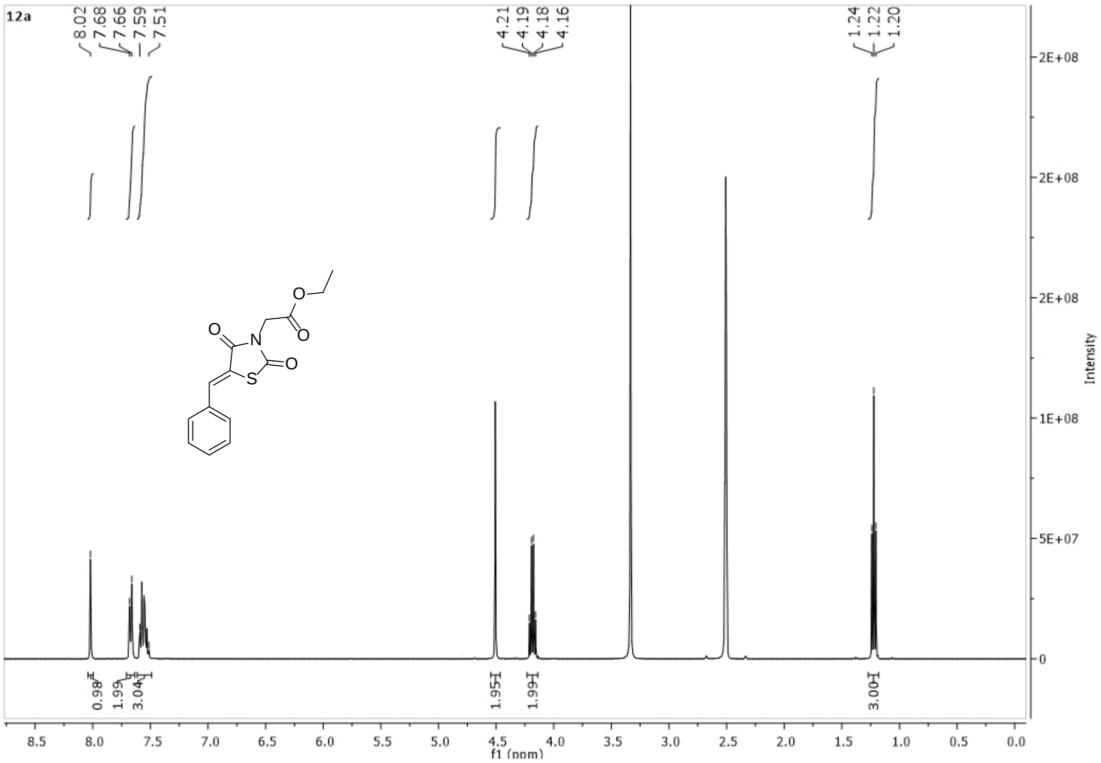


$^{1}$H-NMR spectrum of ethyl (*Z*)-2-(5-benzylidenethiazolidine-2,4-dion-3-yl)acetate

(**12a**)

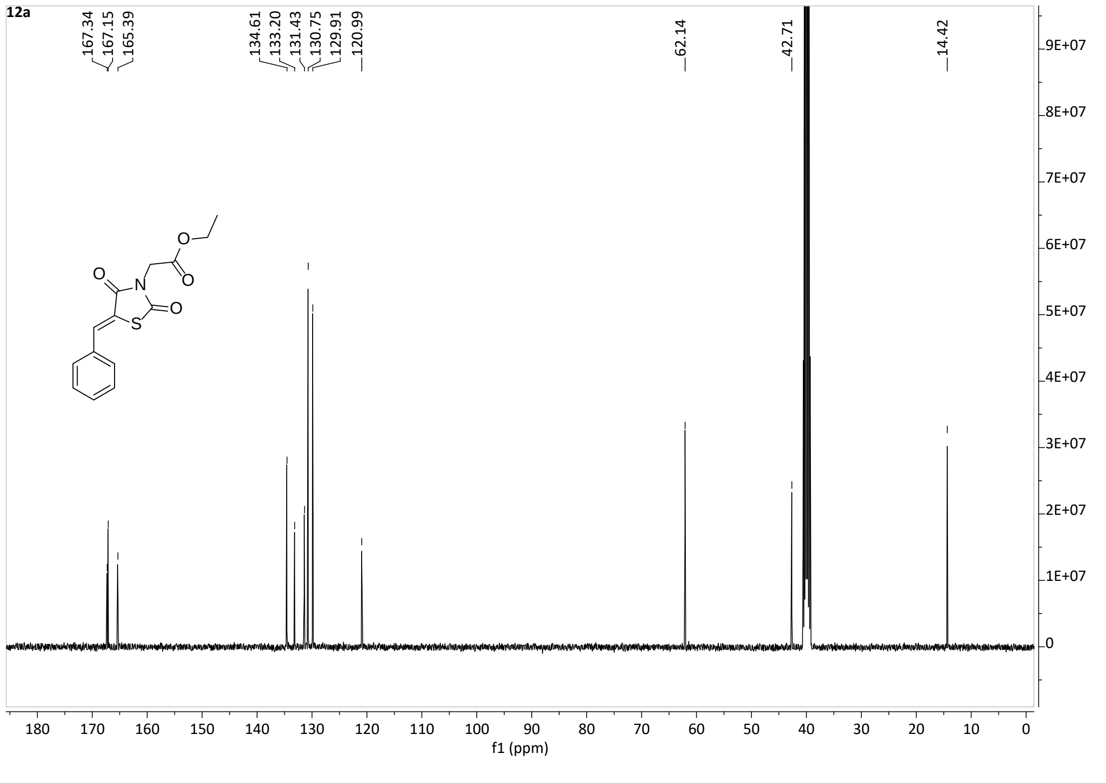

$^{13}$C-NMR spectrum of ethyl (*Z*)-2-(5-benzylidenethiazolidine-2,4-dion-3-yl)acetate (**12a**)

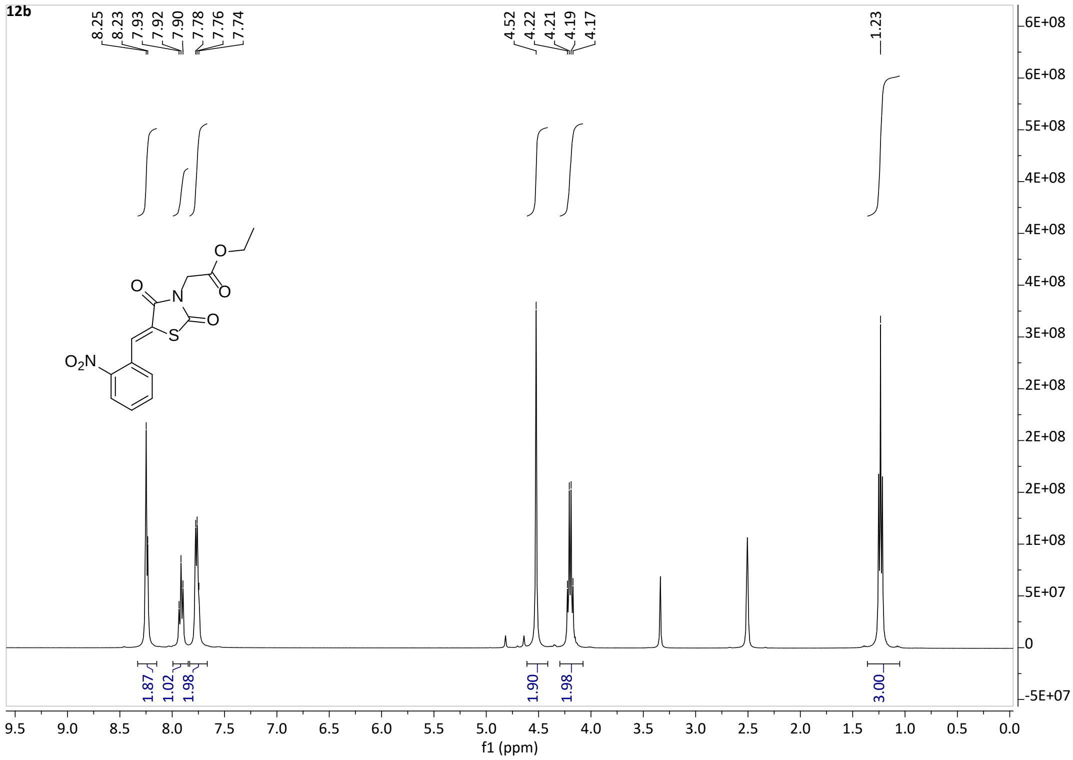


$^{1}$H-NMR spectrum of ethyl ((*Z*)-5-(2-nitrobenzylidene)thiazolidine-2,4-dion-3-yl) acetate (**12b**)

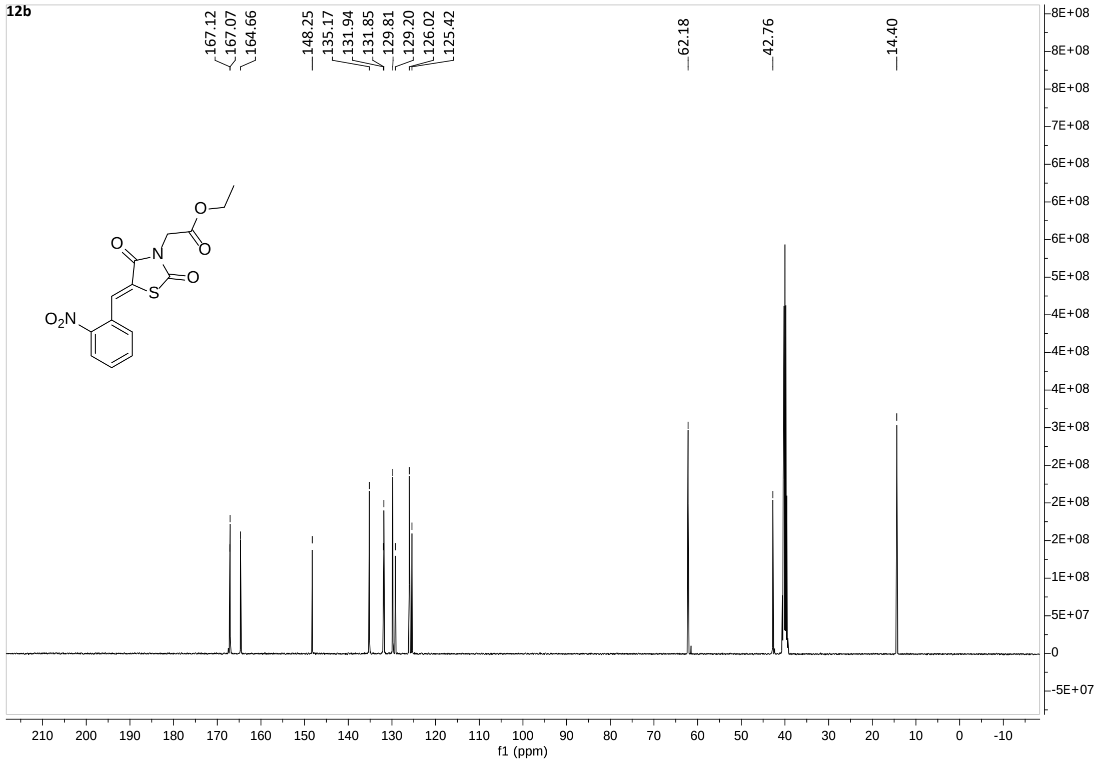

$^{13}$C-NMR spectrum of ethyl ((*Z*)-5-(2-nitrobenzylidene)thiazolidine-2,4-dion-3-yl) acetate (**12b**)

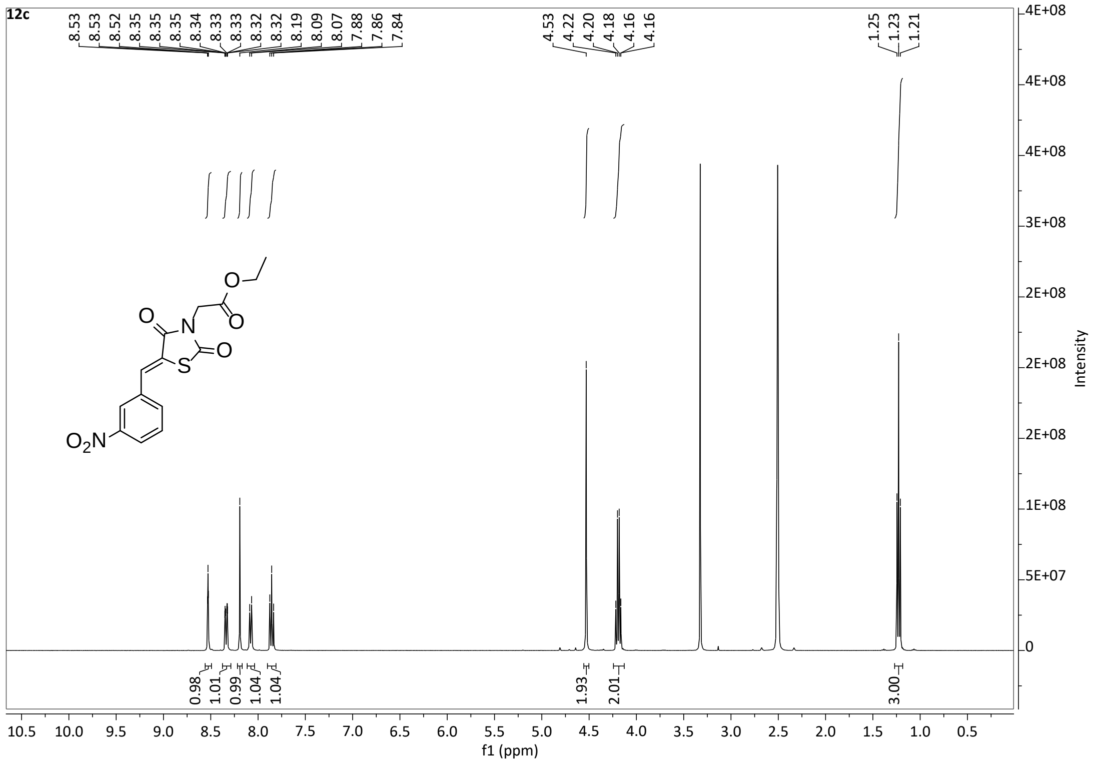


$^{1}$H-NMR spectrum of ethyl ((*Z*)-5-(3-nitrobenzylidene)thiazolidine-2,4-dion-3-yl) acetate (**12c**)

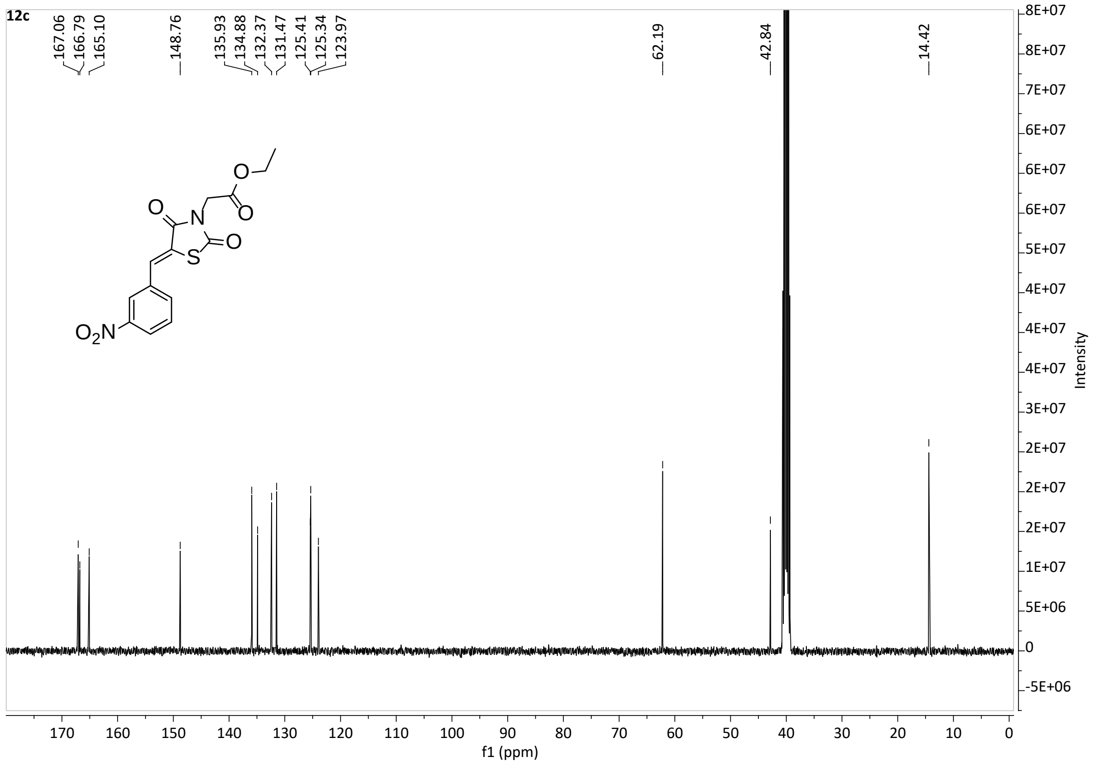

$^{13}$C-NMR spectrum of ethyl ((*Z*)-5-(3-nitrobenzylidene)thiazolidine-2,4-dion-3-yl) acetate (**12c**)

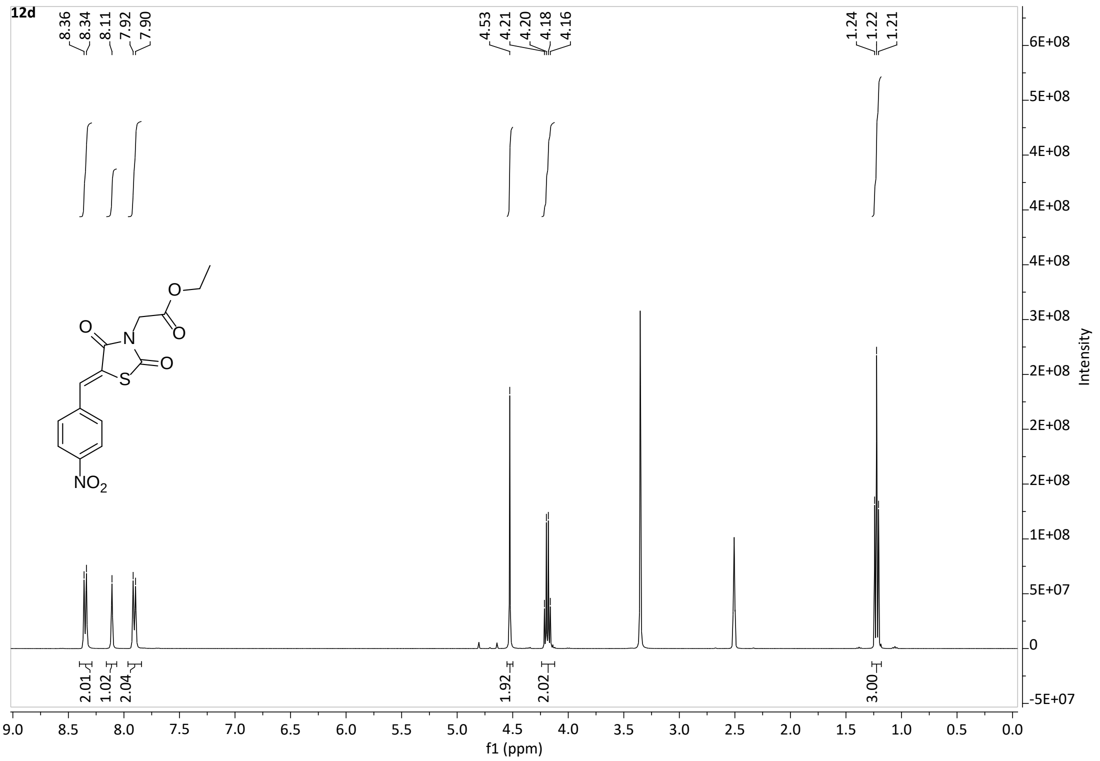


$^1$H-NMR spectrum of ethyl ((*Z*)-5-(4-nitrobenzylidene)thiazolidine-2,4-dion-3-yl) acetate (**12d**)

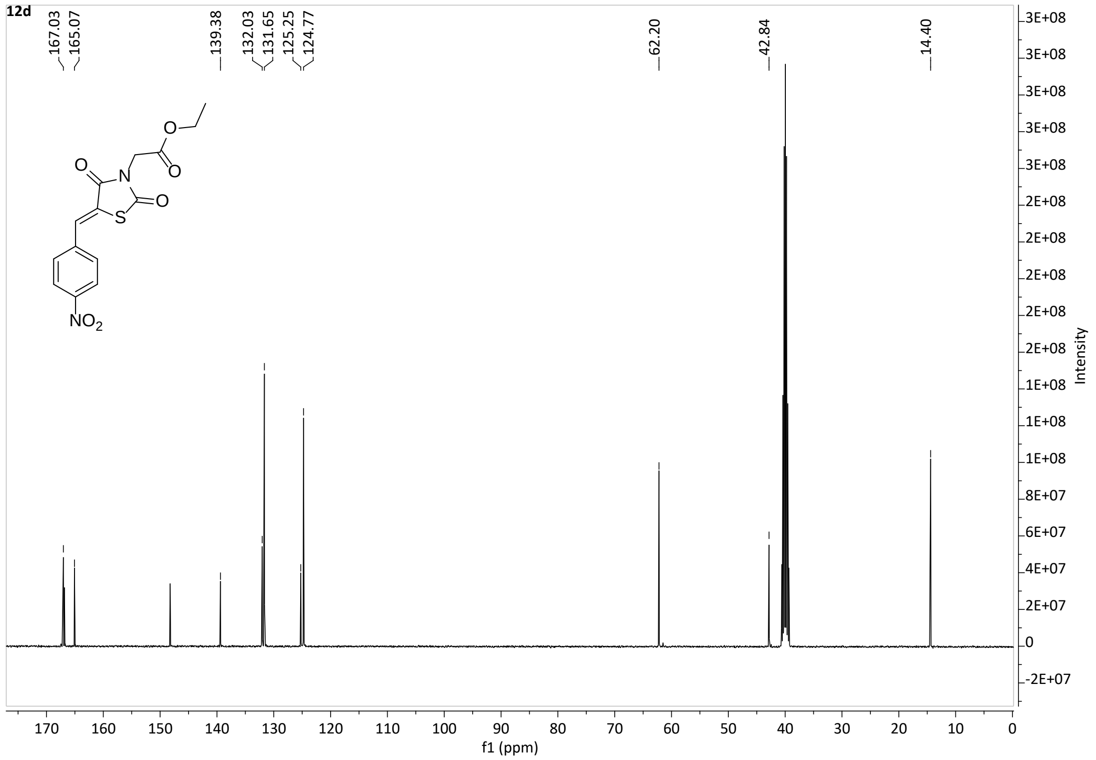

$^{13}$C-NMR spectrum of ethyl ((*Z*)-5-(4-nitrobenzylidene)thiazolidine-2,4-dion-3-yl) acetate (**12d**)

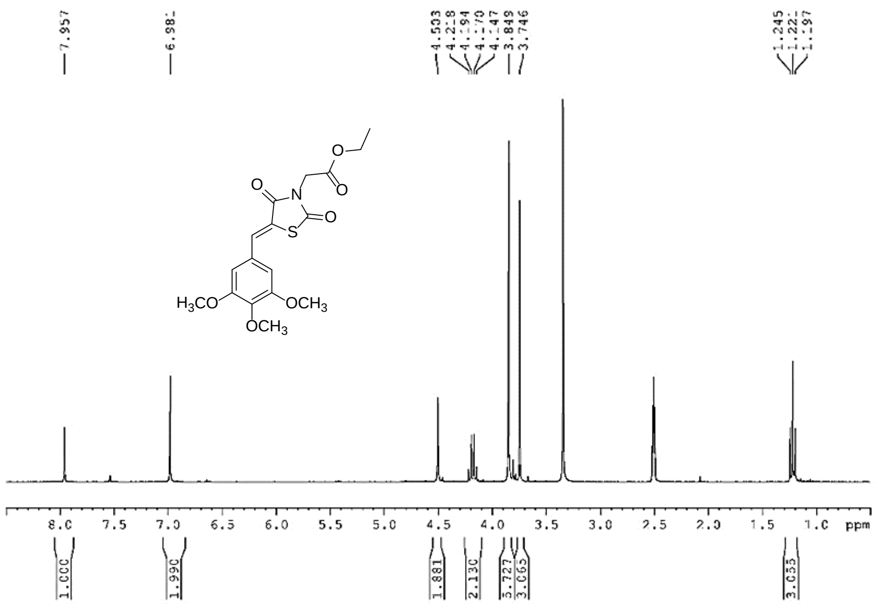

$^{1}$H-NMR spectrum of ethyl ((*Z*)-5-(3,4,5-trimethoxybenzylidene)thiazolidine-2,4-dion-3-yl)acetate (**12e**)

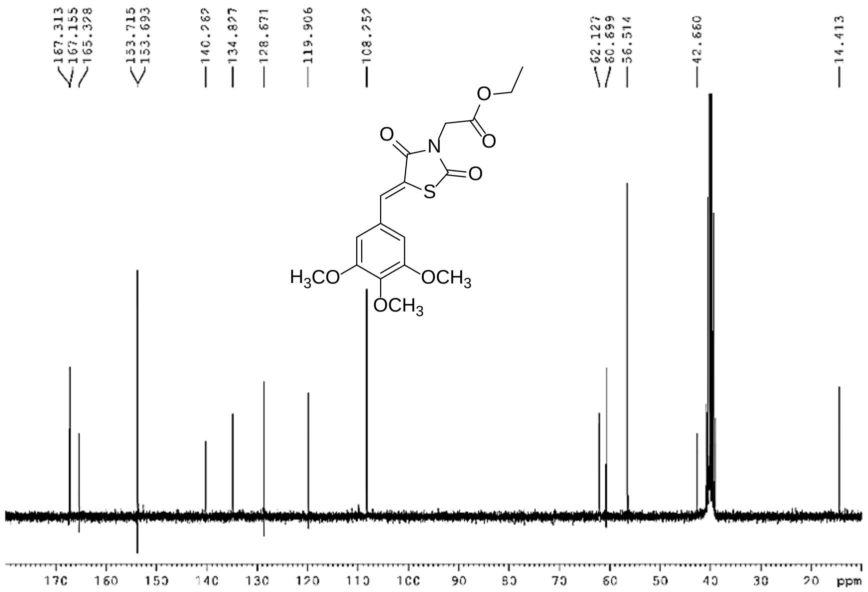

$^{13}$C-NMR spectrum of ethyl ((*Z*)-5-(3,4,5-trimethoxybenzylidene)thiazolidine-2,4-dion-3-yl)acetate (**12e**)

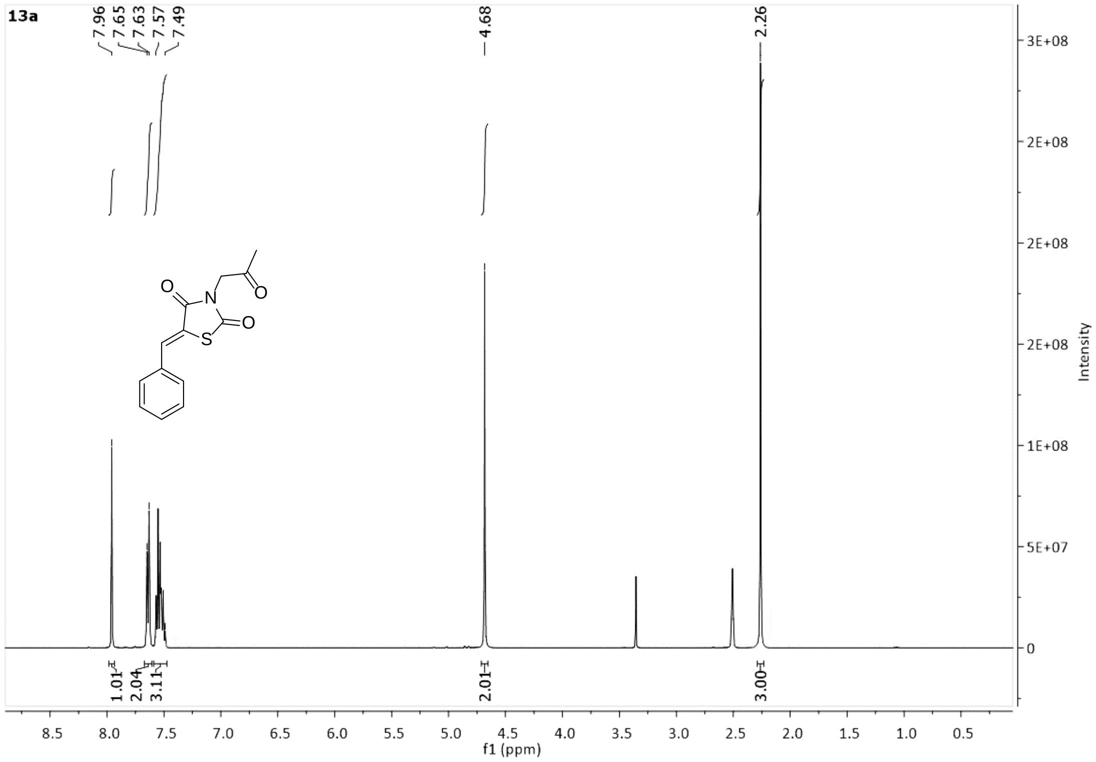


$^{1}$H-NMR spectrum of (*Z*)-5-benzylidene-3-(2-oxopropyl)thiazolidine-2,4-dione (**13a**)

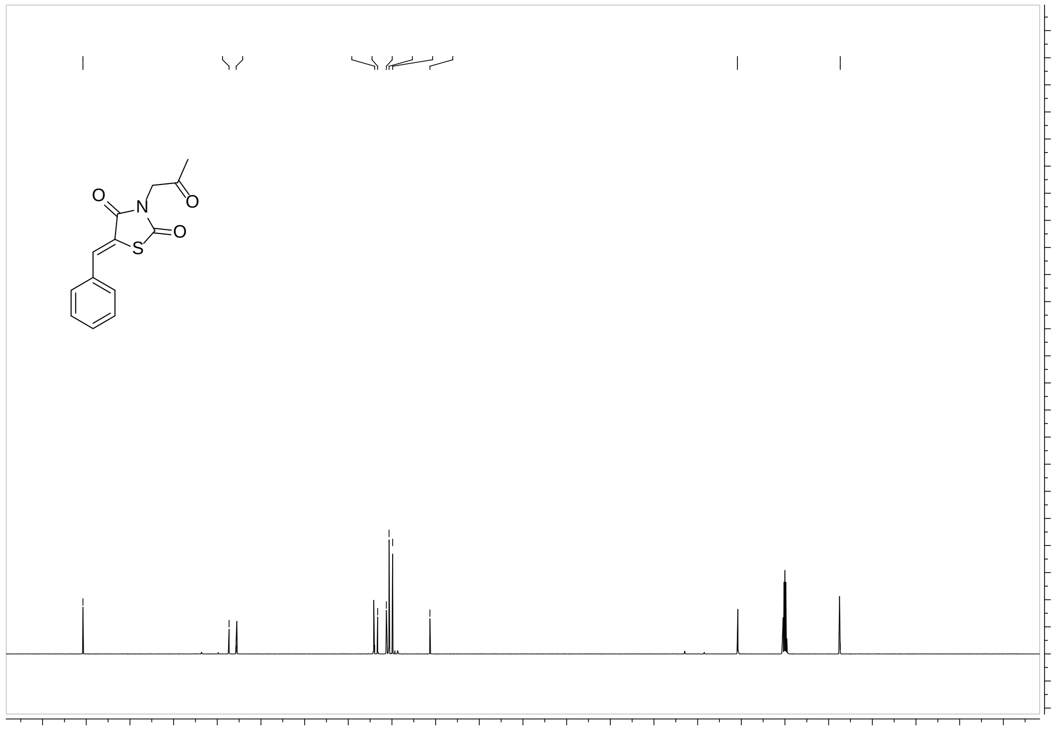

$^{13}$C-NMR spectrum of (*Z*)-5-benzylidene-3-(2-oxopropyl)thiazolidine-2,4-dione (**13a**)

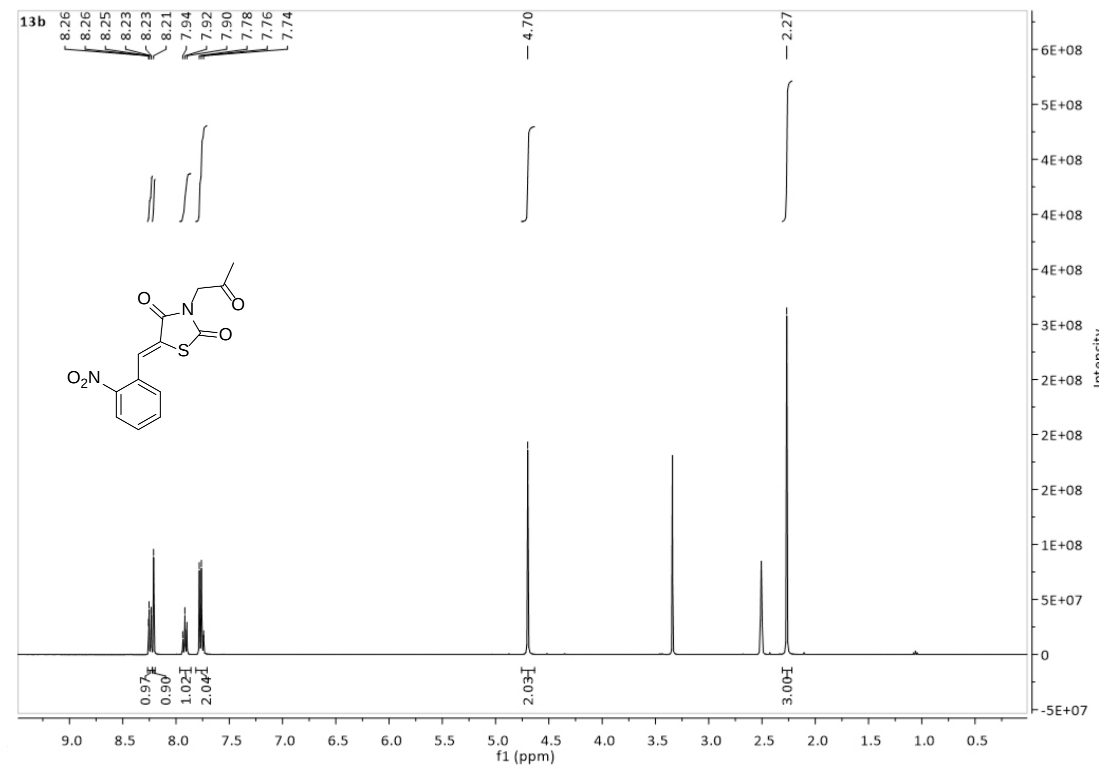


$^{1}$H-NMR spectrum of (*Z*)-5-(2-nitrobenzylidene)-3-(2-oxopropyl)thiazolidine-2,4-dione (**13b**)

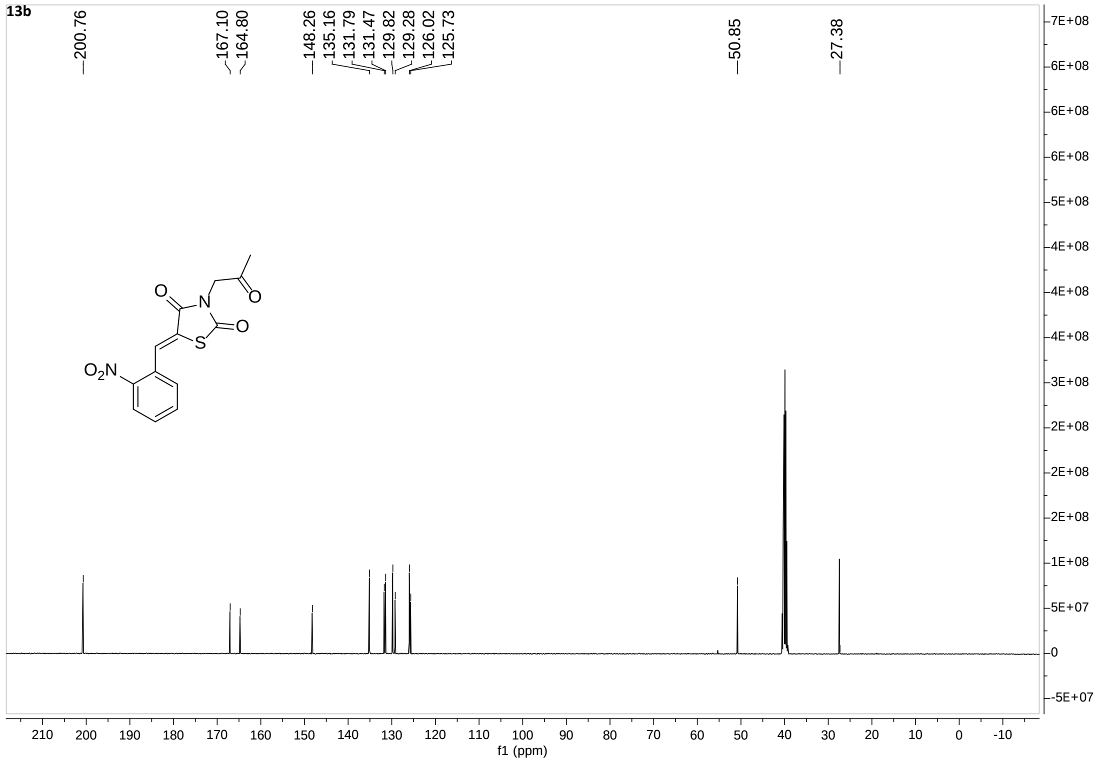


$^{13}$C-NMR spectrum of (*Z*)-5-(2-nitrobenzylidene)-3-(2-oxopropyl)thiazolidine-2,4-dione (**13b**)

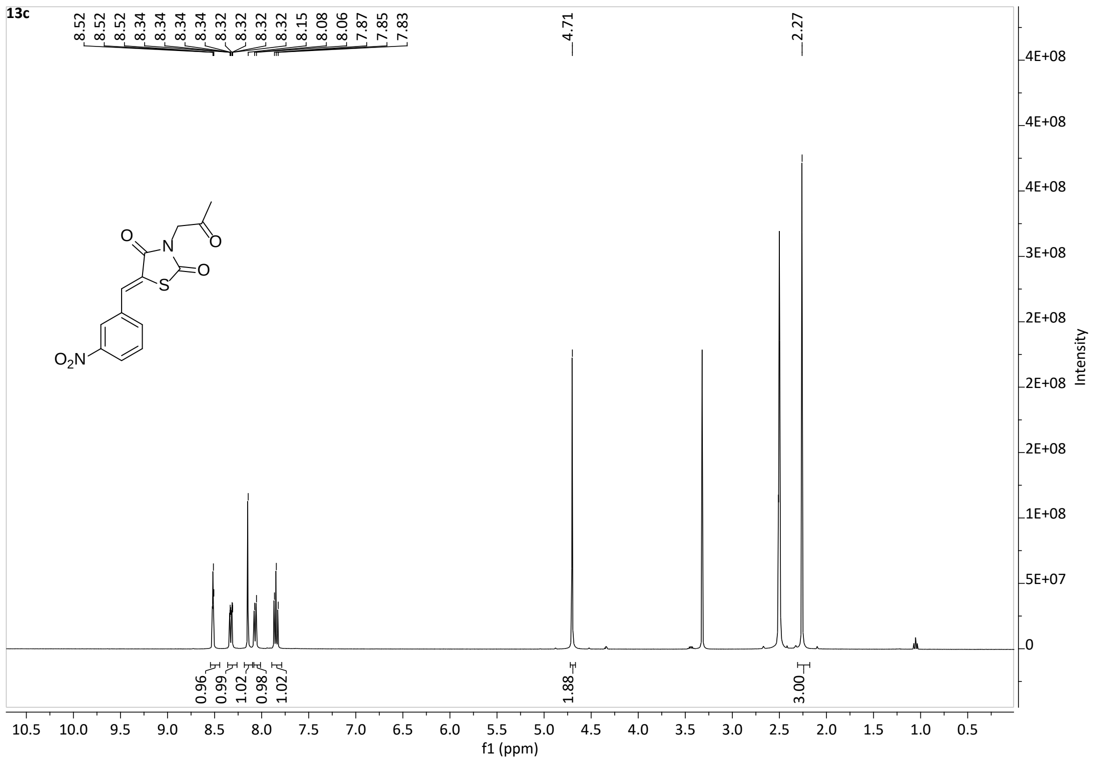

$^{1}$H-NMR spectrum of (*Z*)-5-(3-nitrobenzylidene)-3-(2-oxopropyl)thiazolidine-2,4-dione (**13c**)

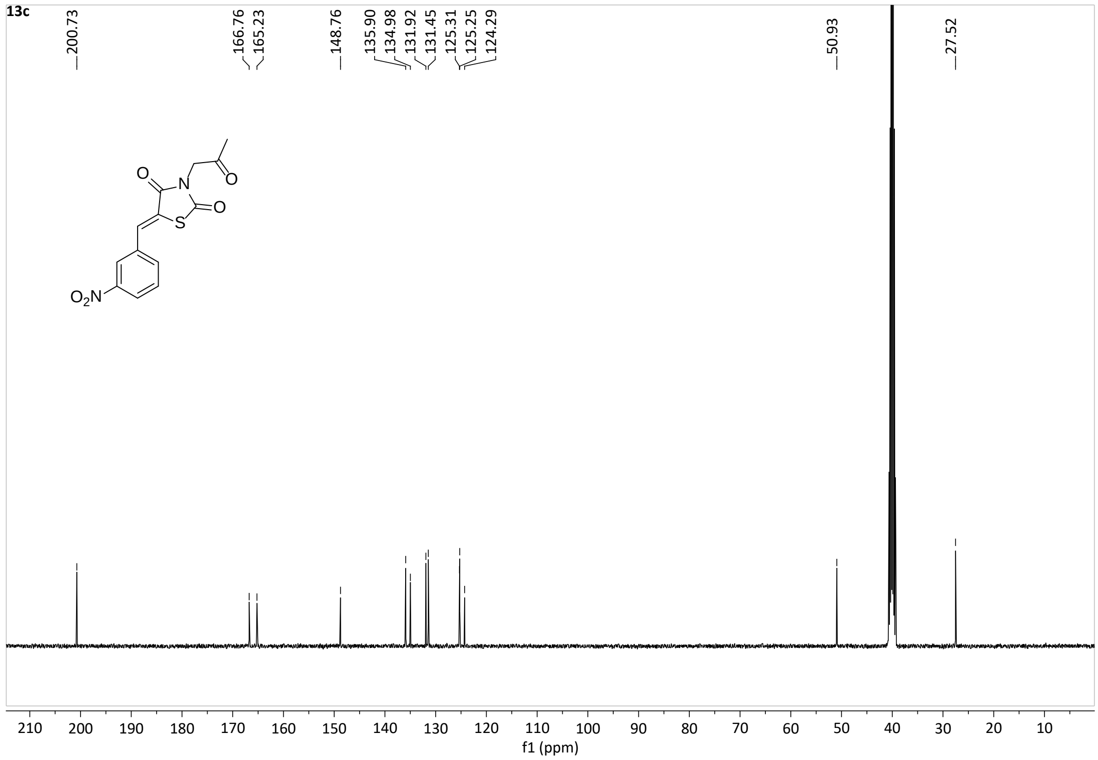

$^{13}$C-NMR spectrum of (*Z*)-5-(3-nitrobenzylidene)-3-(2-oxopropyl)thiazolidine-2,4-dione (**13c**)

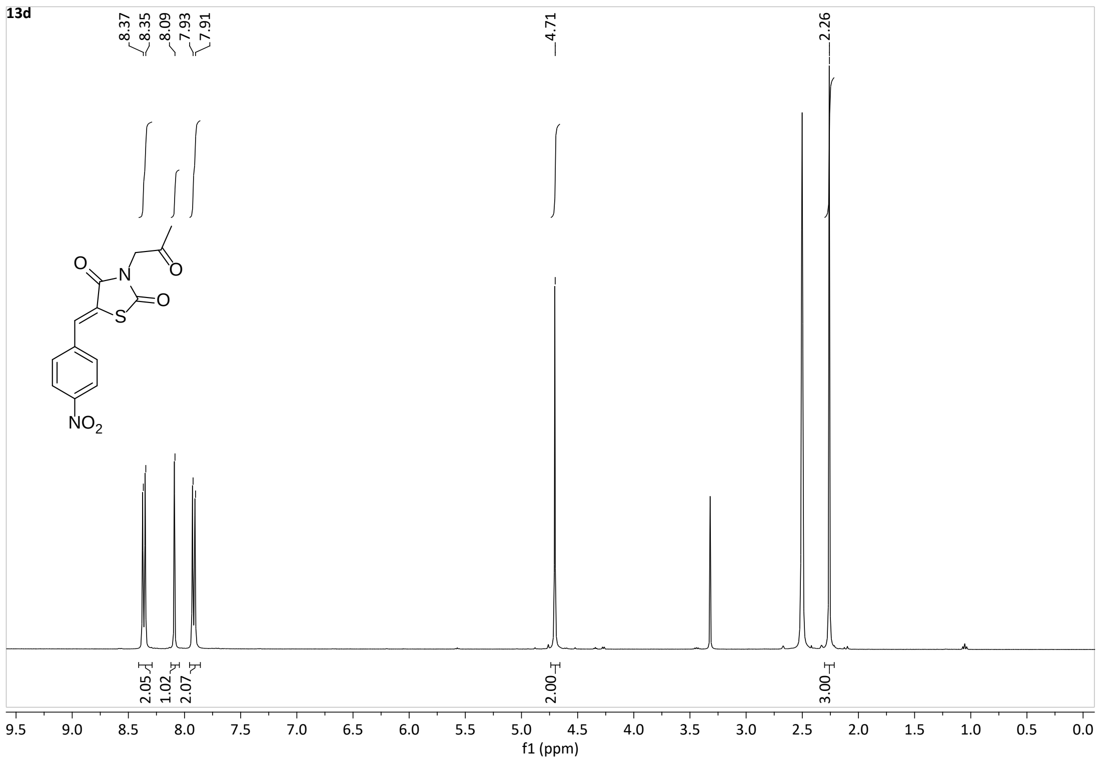


$^{1}$H-NMR spectrum of (*Z*)-5-(4-nitrobenzylidene)-3-(2-oxopropyl)thiazolidine-2,4-dione (**13d**)

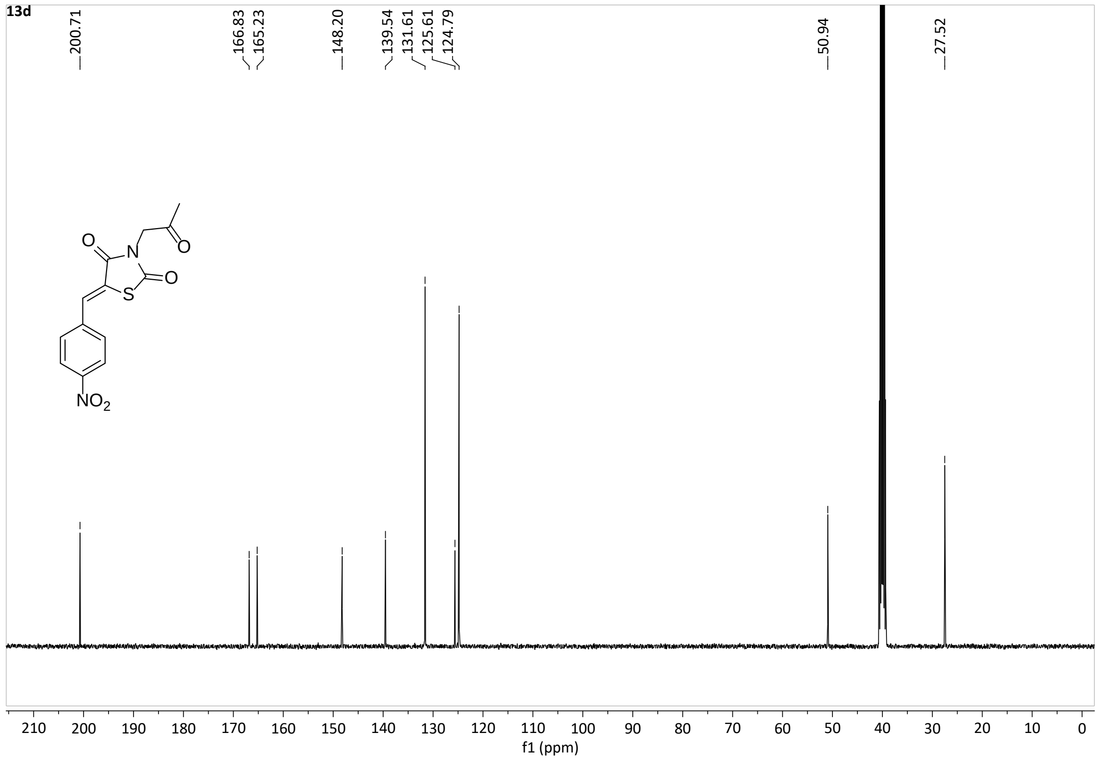


$^{13}$C-NMR spectrum of (*Z*)-5-(4-nitrobenzylidene)-3-(2-oxopropyl)thiazolidine-2,4-dione (**13d**)

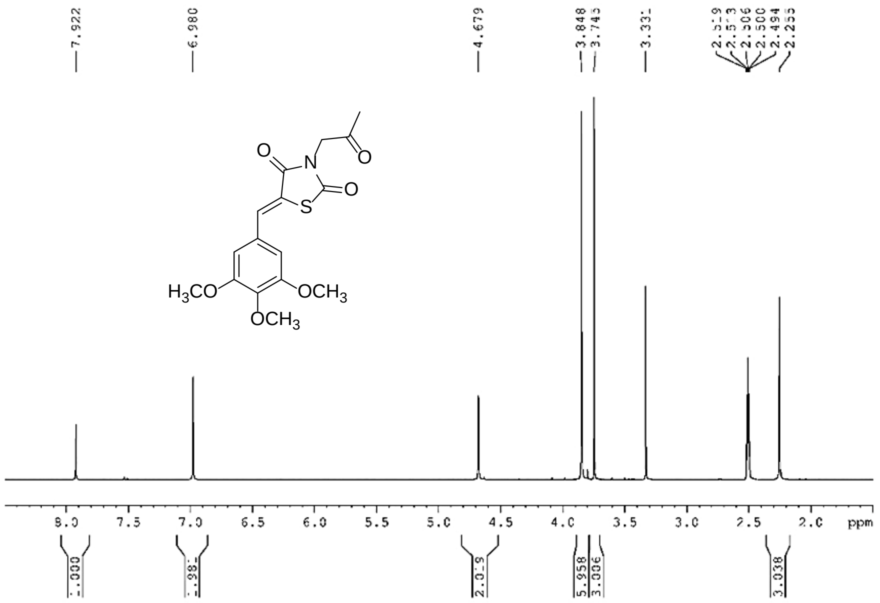


$^{1}$H-NMR spectrum of (*Z*)-5-(3,4,5-trimethoxybenzylidene)-3-(2-oxopropyl) thiazolidine-2,4-dione (**13e**)

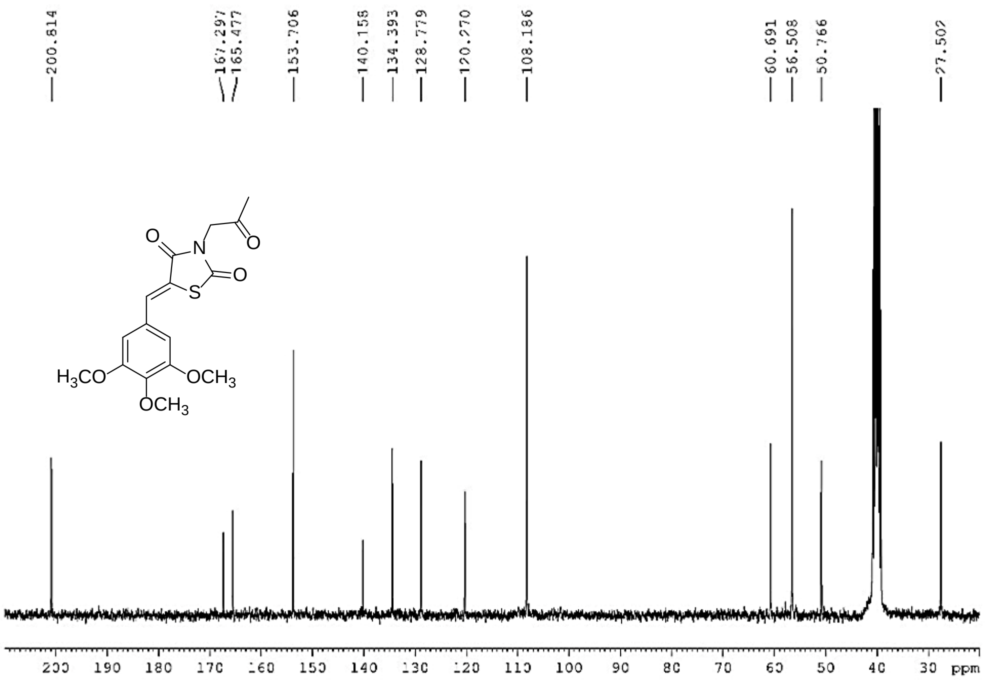


$^{13}$C-NMR spectrum of (*Z*)-5-(3,4,5-trimethoxybenzylidene)-3-(2-oxopropyl) thiazolidine-2,4-dione (**13e**)

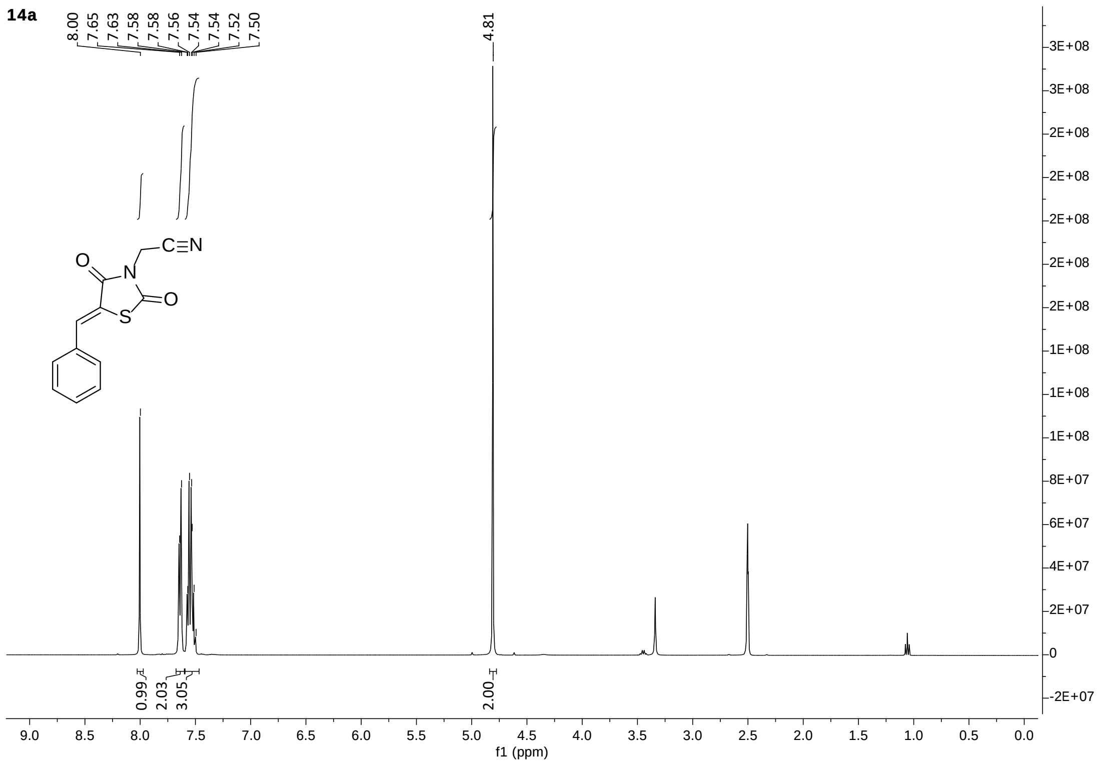


$^{1}$H-NMR spectrum of (*Z*)-2-(5-Benzylidenethiazolidine-2,4-dion-3-yl)acetonitrile (**14a**)

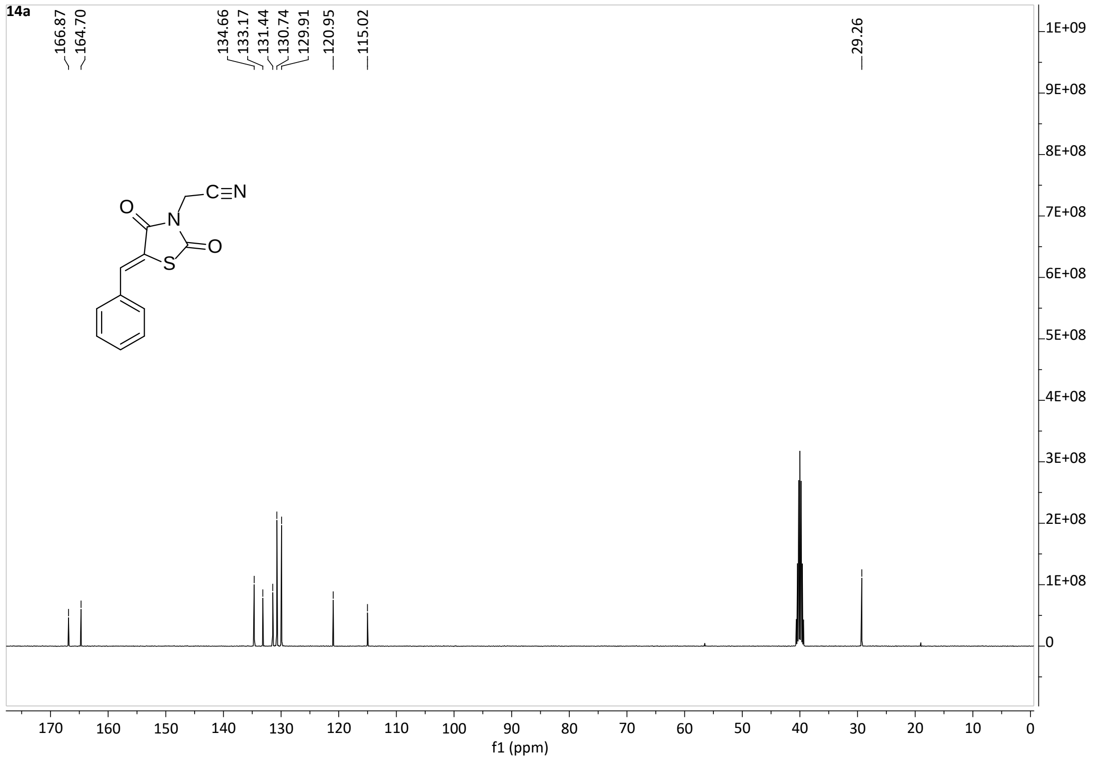


$^{13}$C-NMR spectrum of (*Z*)-2-(5-Benzylidenethiazolidine-2,4-dion-3-yl)acetonitrile (**14a**)

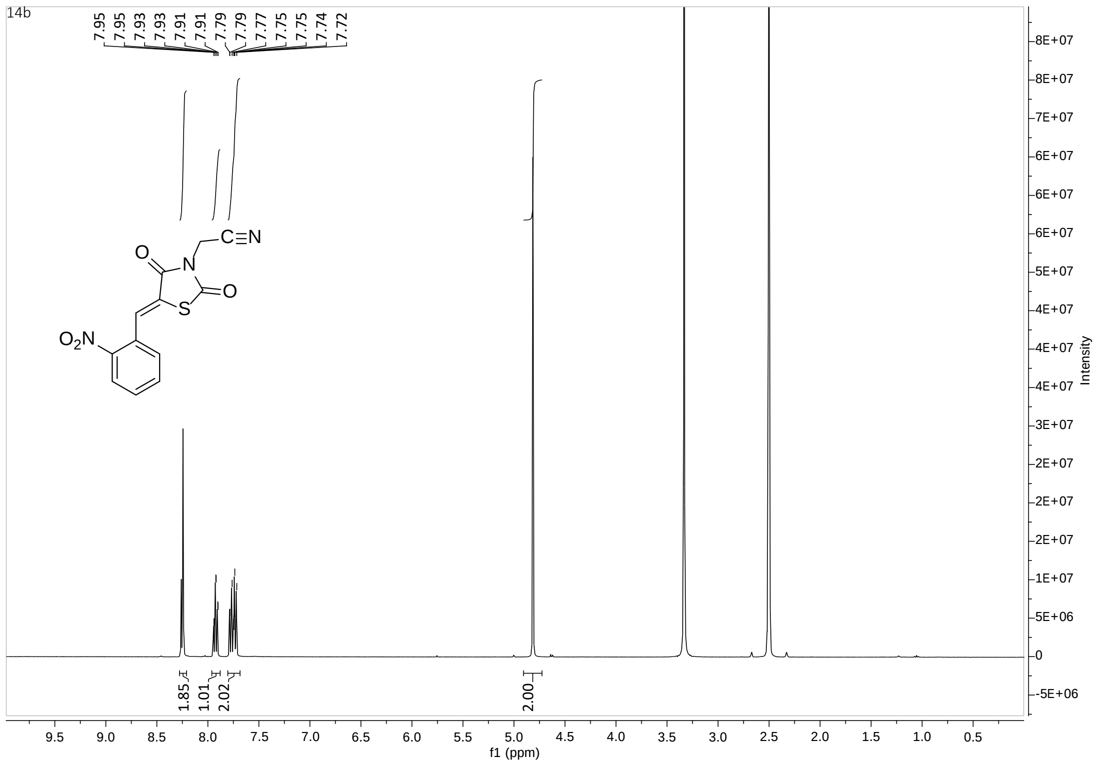


$^{1}$H-NMR spectrum of (*Z*)-2-(5-(2-nitrobenzylidene)thiazolidine-2,4-dion-3-yl) acetonitrile (**14b**)

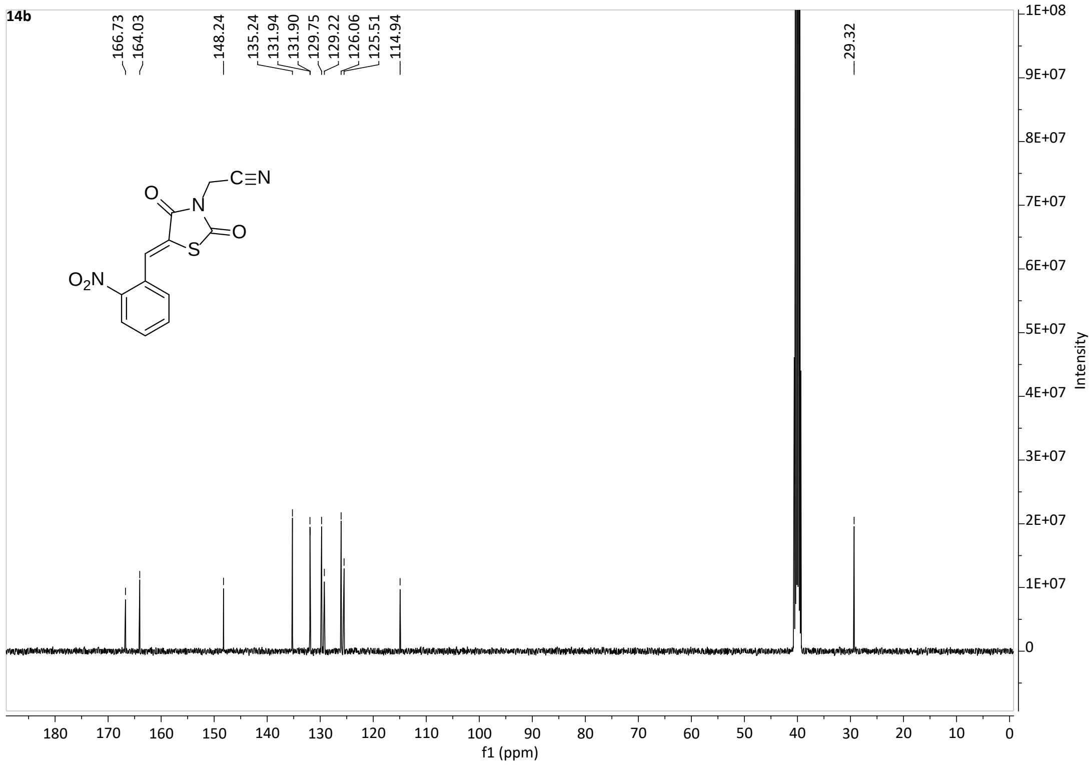


$^{13}$C-NMR spectrum of (*Z*)-2-(5-(2-nitrobenzylidene)thiazolidine-2,4-dion-3-yl) acetonitrile (**14b**)

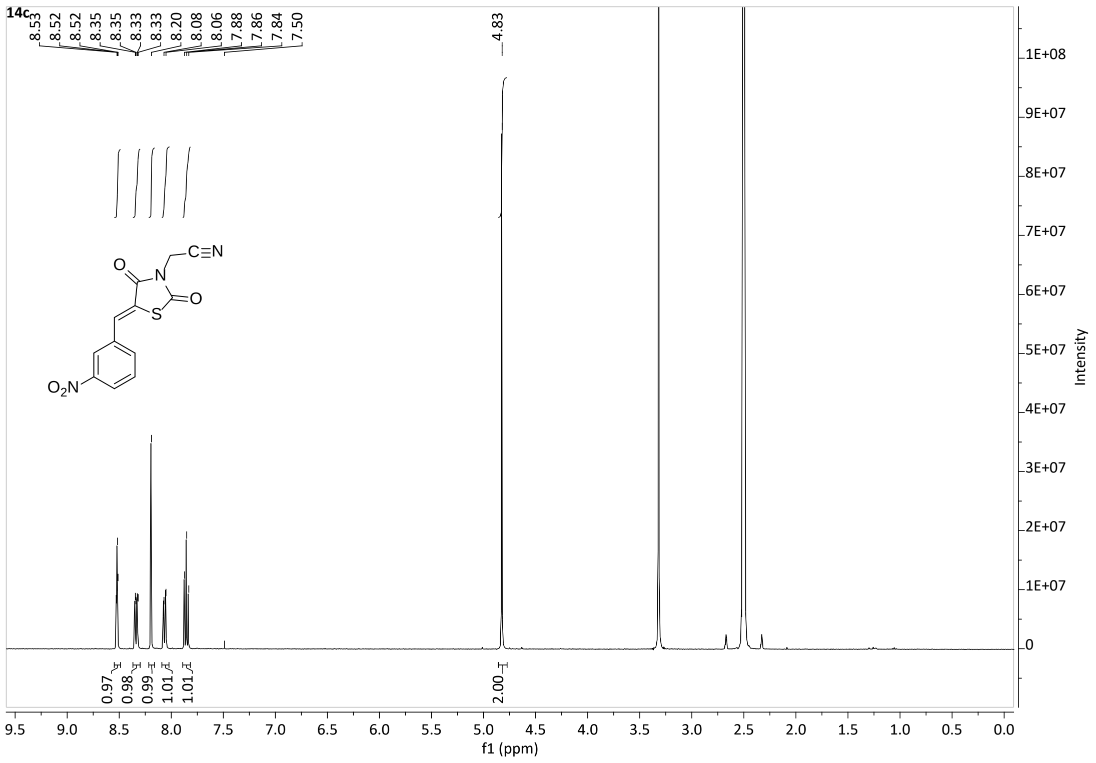


$^{1}$H-NMR spectrum of (*Z*)-2-(5-(3-nitrobenzylidene)thiazolidine-2,4-dion-3-yl) acetonitrile (**14c**)

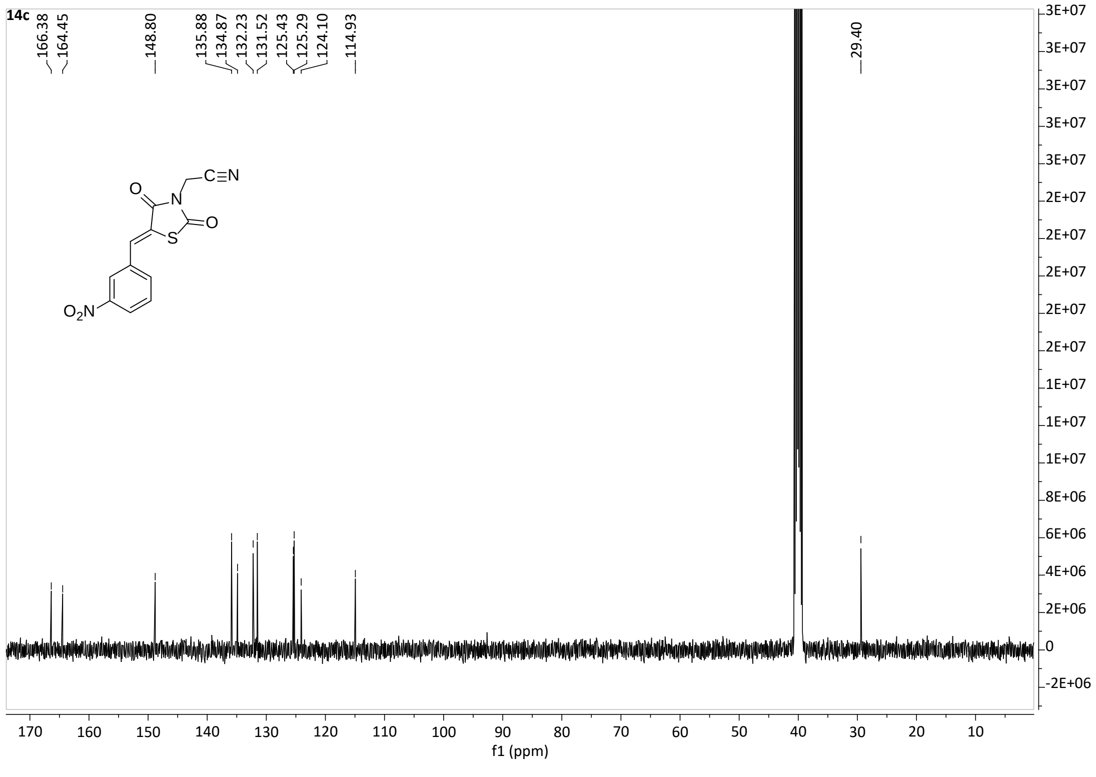


$^{13}$C-NMR spectrum of (*Z*)-2-(5-(3-nitrobenzylidene)thiazolidine-2,4-dion-3-yl) acetonitrile (**14c**)

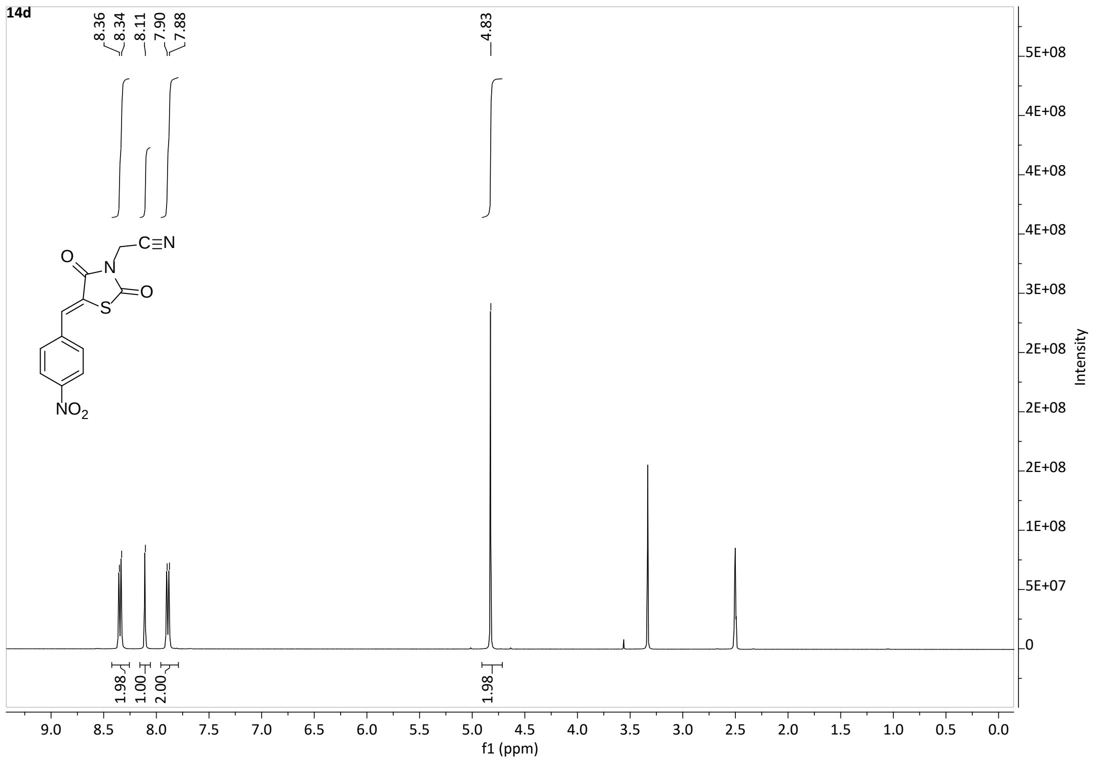


$^{1}$H-NMR spectrum of (*Z*)-2-(5-(4-nitrobenzylidene)thiazolidine-2,4-dion-3-yl) acetonitrile (**14d**)

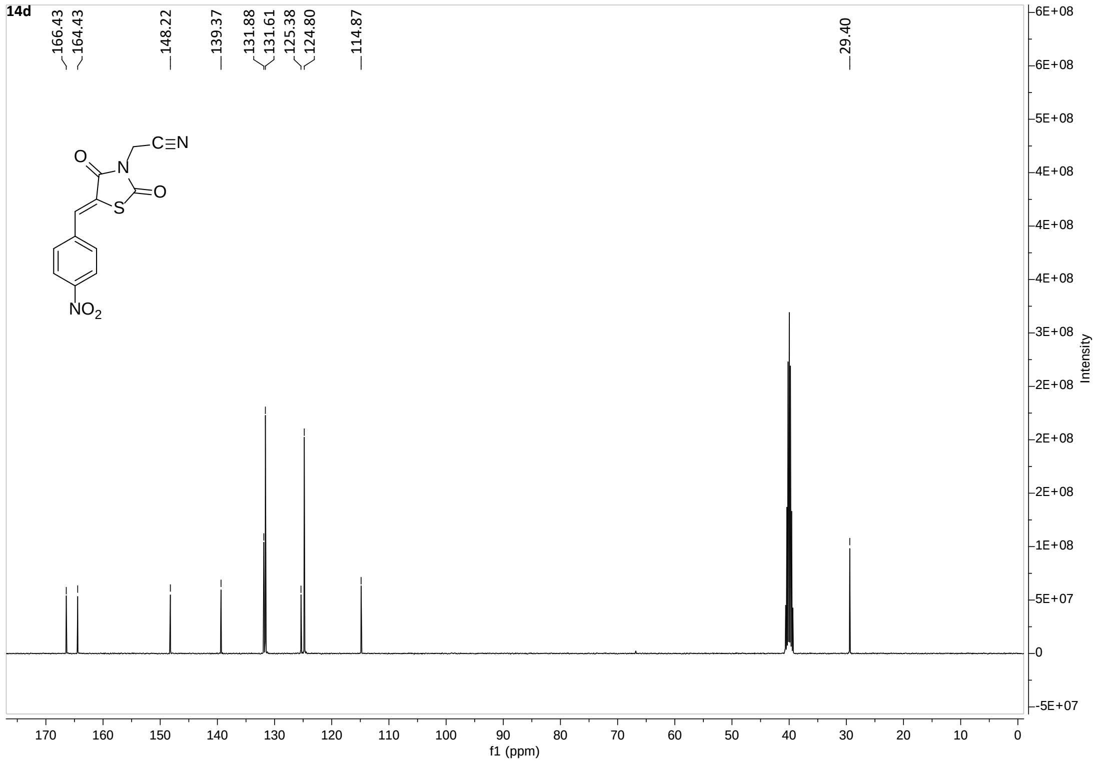


$^{13}$C-NMR spectrum of (*Z*)-2-(5-(4-nitrobenzylidene)thiazolidine-2,4-dion-3-yl) acetonitrile (**14d**)

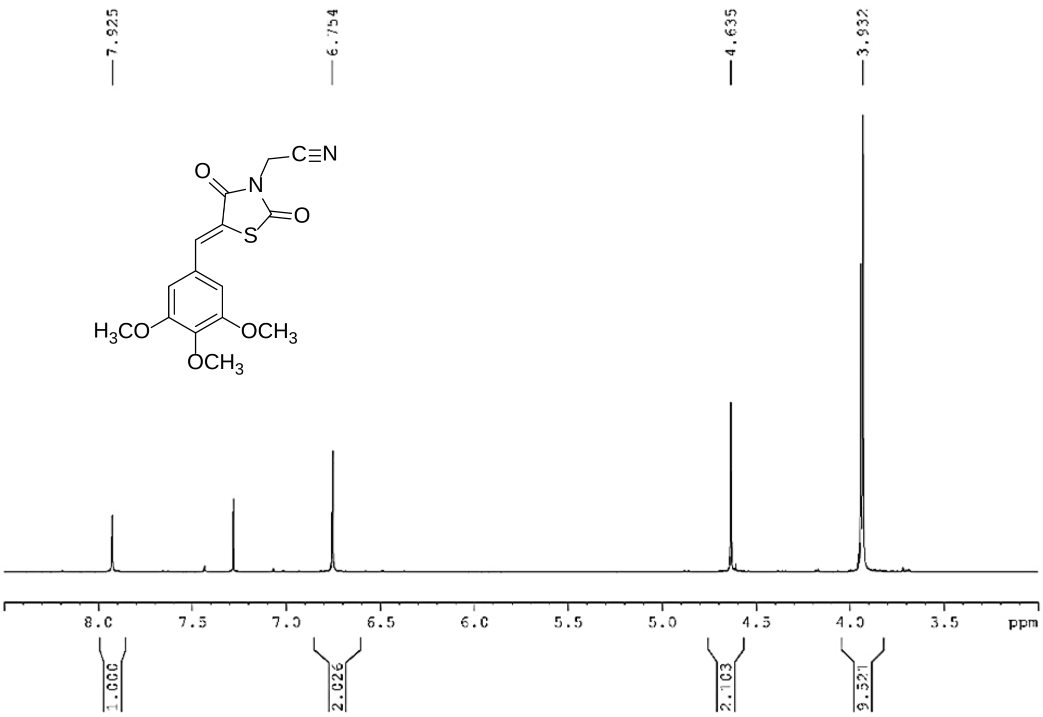


$^{1}$H-NMR spectrum of (*Z*)-2-(5-(3,4,5-trimethoxybenzylidene)thiazolidine-2,4-dion-3-

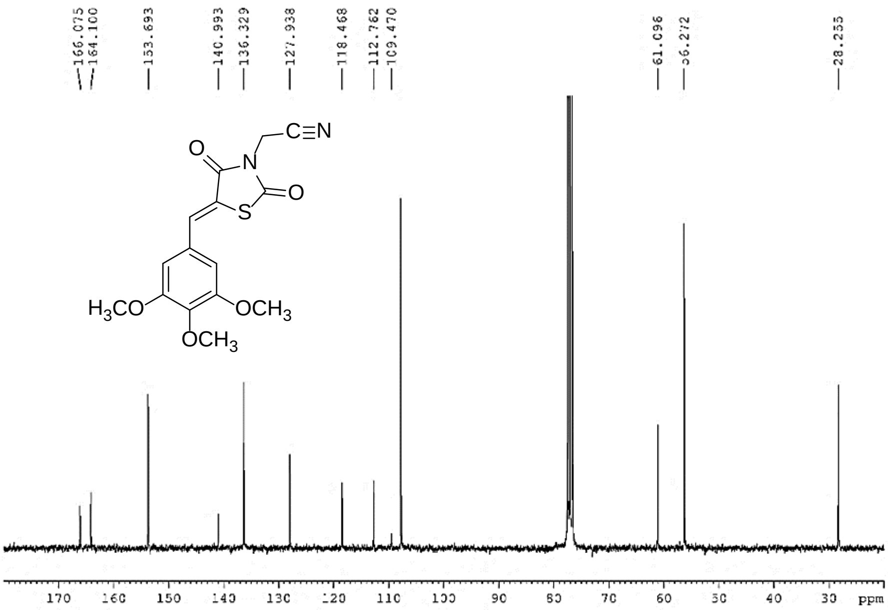


yl)acetonitrile (**14e**)

$^{13}$C-NMR spectrum of (*Z*)-2-(5-(3,4,5-trimethoxybenzylidene)thiazolidine-2,4-dion-3-yl)acetonitrile (**14e**)

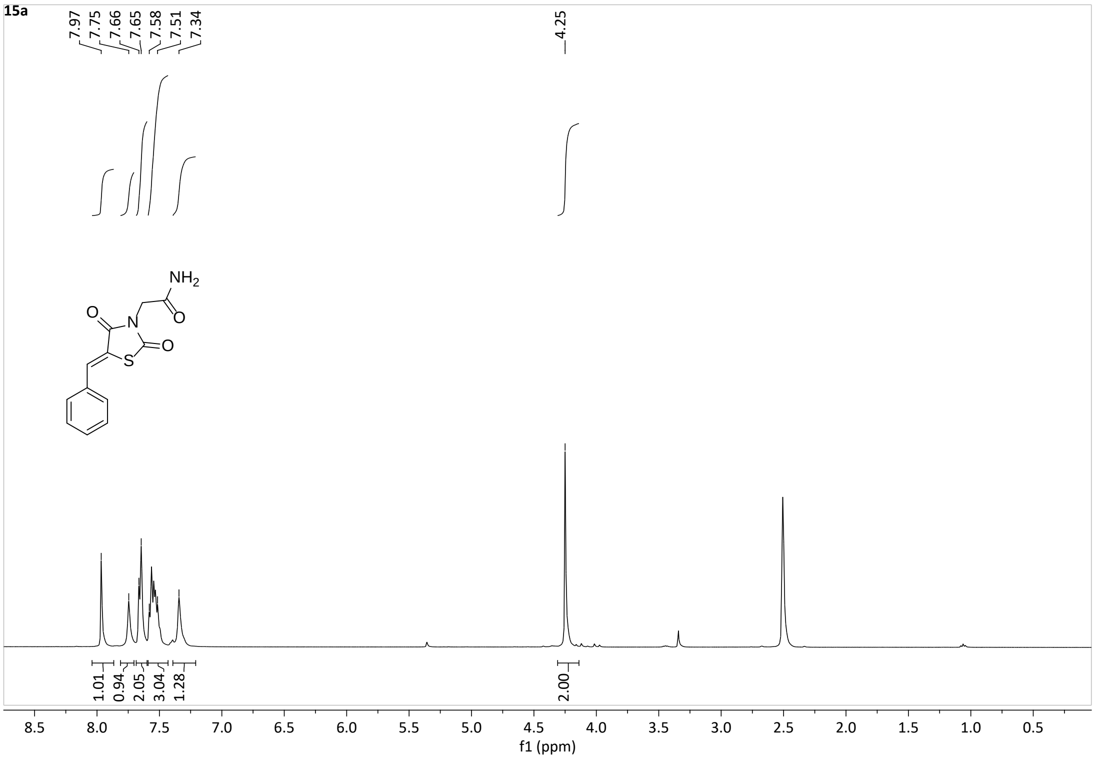


$^{1}$H-NMR spectrum of (*Z*)-2-(5-(benzylidene)thiazolidine-2,4-dion-3-yl) acetamide (**15a**)

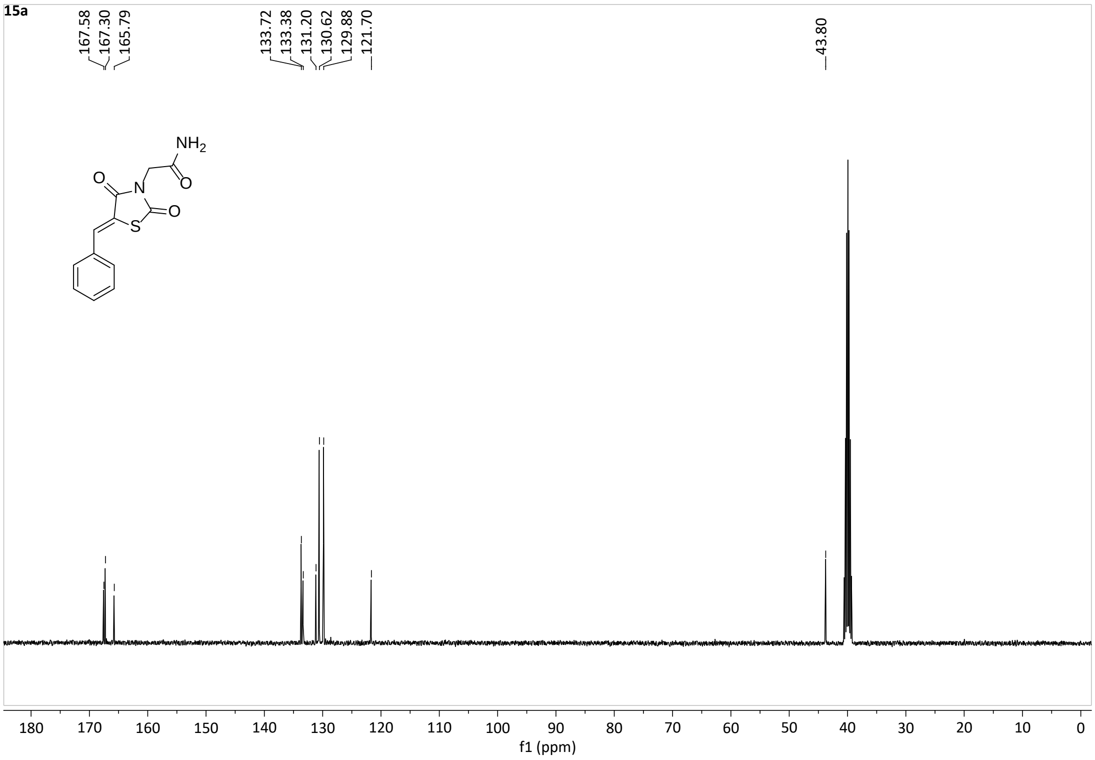


$^{13}$C-NMR spectrum of (*Z*)-2-(5-(benzylidene)thiazolidine-2,4-dion-3-yl) acetamide (**15a**)

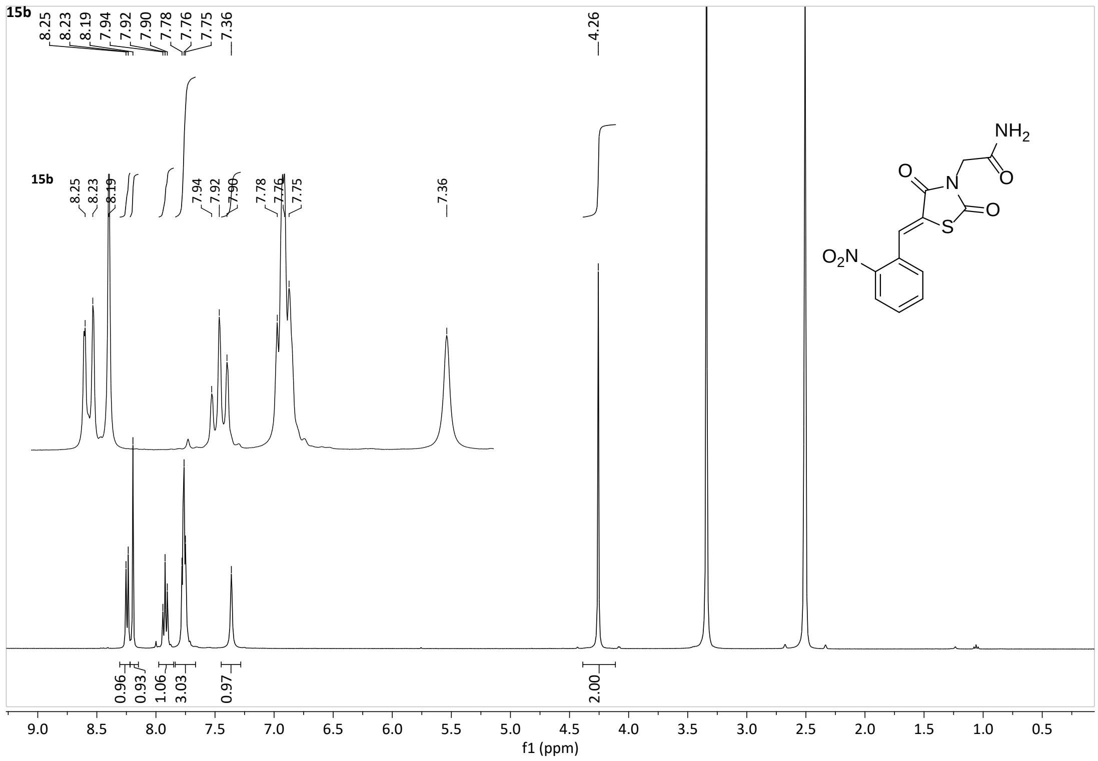


$^{1}$H-NMR spectrum of (*Z*)-2-(5-(2-nitrobenzylidene)thiazolidine-2,4-dion-3-yl) acetamide (**15b**)

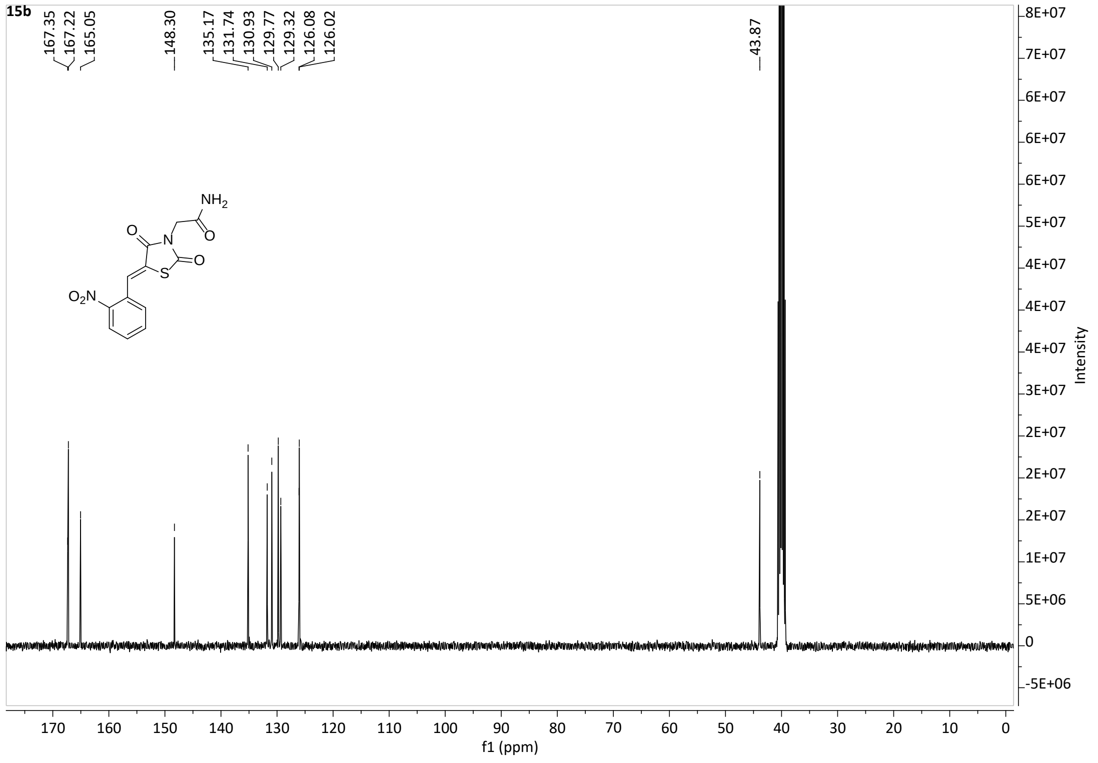

$^{13}$C-NMR spectrum of (*Z*)-2-(5-(2-nitrobenzylidene)thiazolidine-2,4-dion-3-yl) acetamide (**15b**)

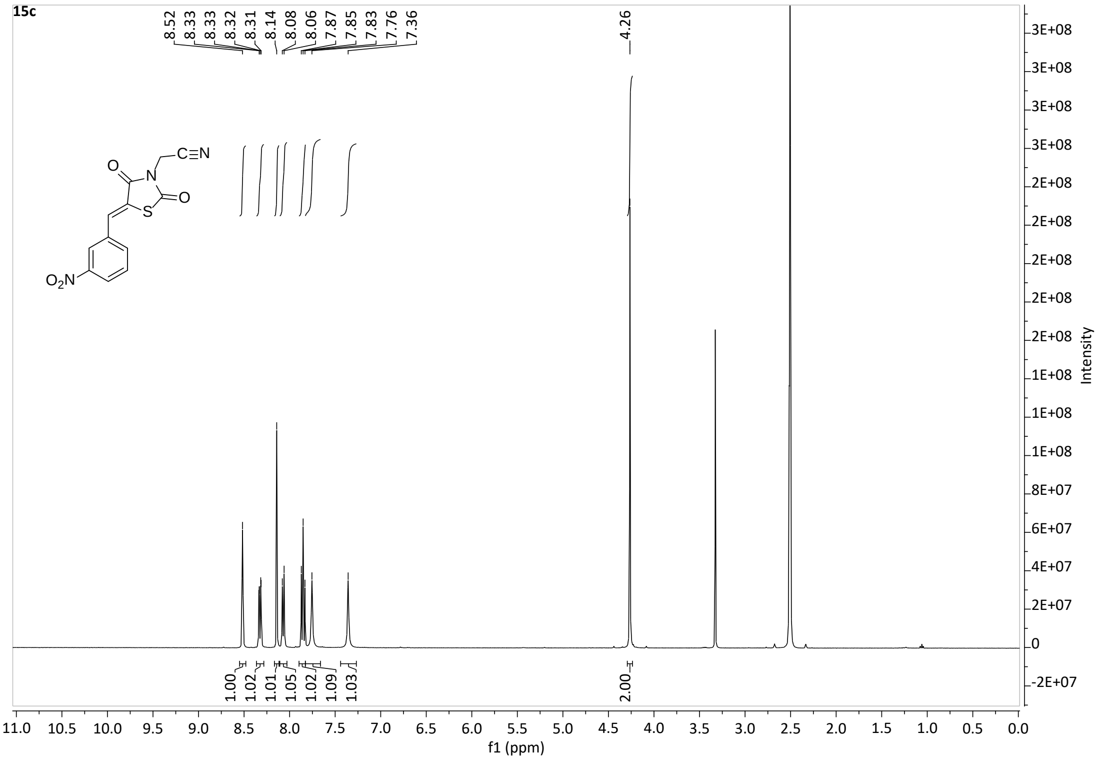


$^{1}$H-NMR spectrum of (*Z*)-2-(5-(3-nitrobenzylidene)thiazolidine-2,4-dion-3-yl) acetamide (**15c**)

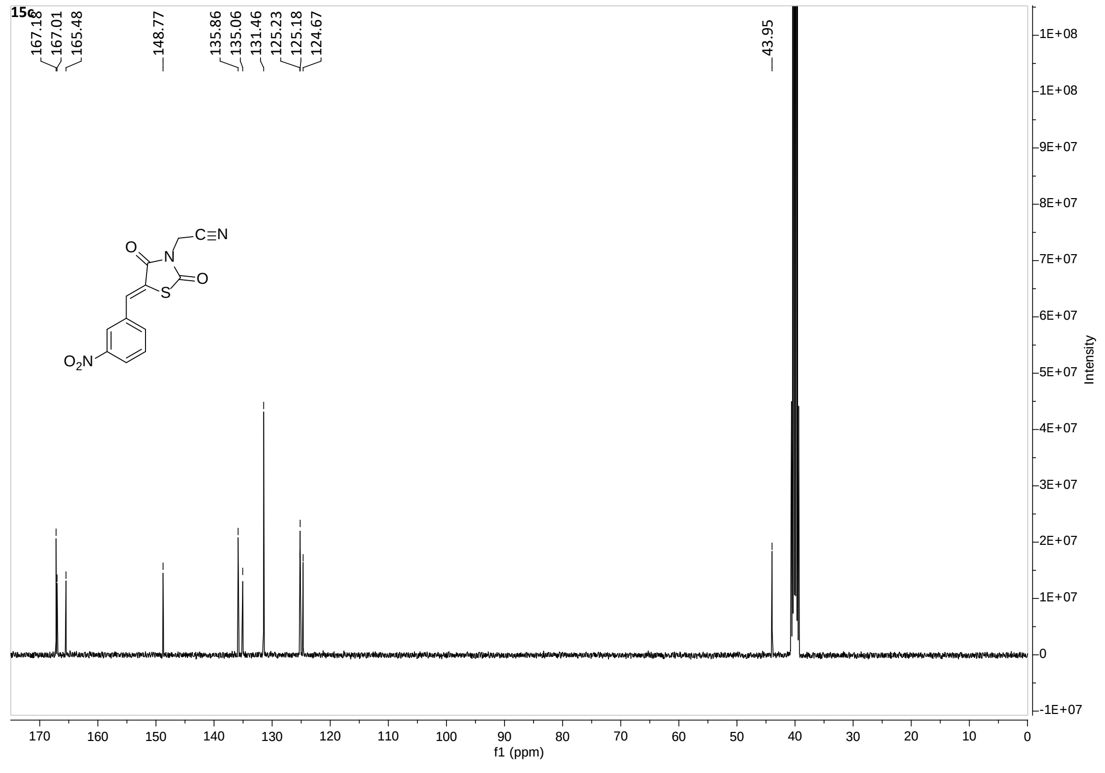

$^{13}C$-NMR spectrum of (*Z*)-2-(5-(3-nitrobenzylidene)thiazolidine-2,4-dion-3-yl) acetamide (**15c**)

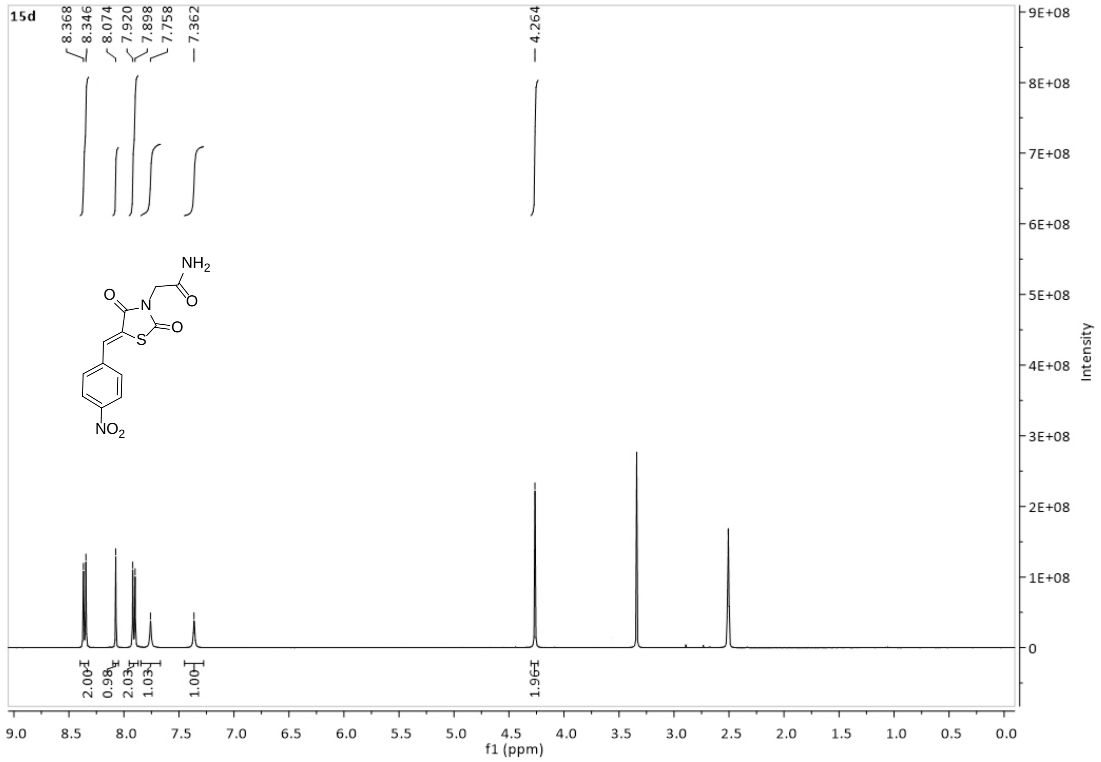


$^{1}H$-NMR spectrum of (*Z*)-2-(5-(4-nitrobenzylidene)thiazolidine-2,4-dion-3-yl) acetamide (**15d**)

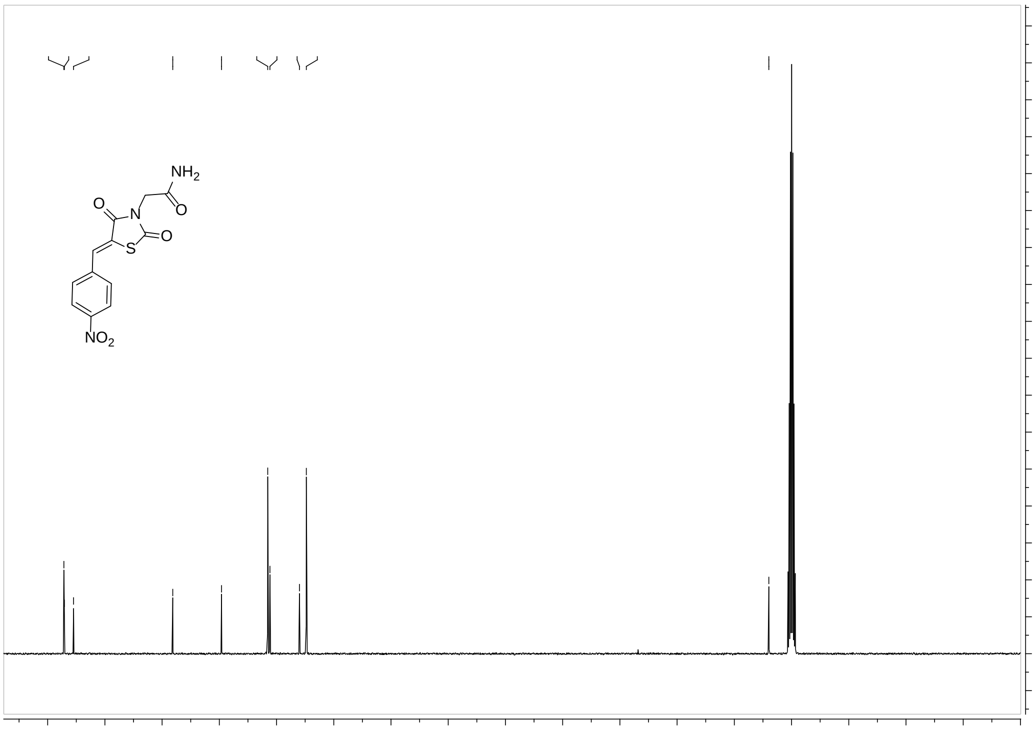


$^{13}$C-NMR spectrum of (*Z*)-2-(5-(4-nitrobenzylidene)thiazolidine-2,4-dion-3-yl) acetamide (**15d**)

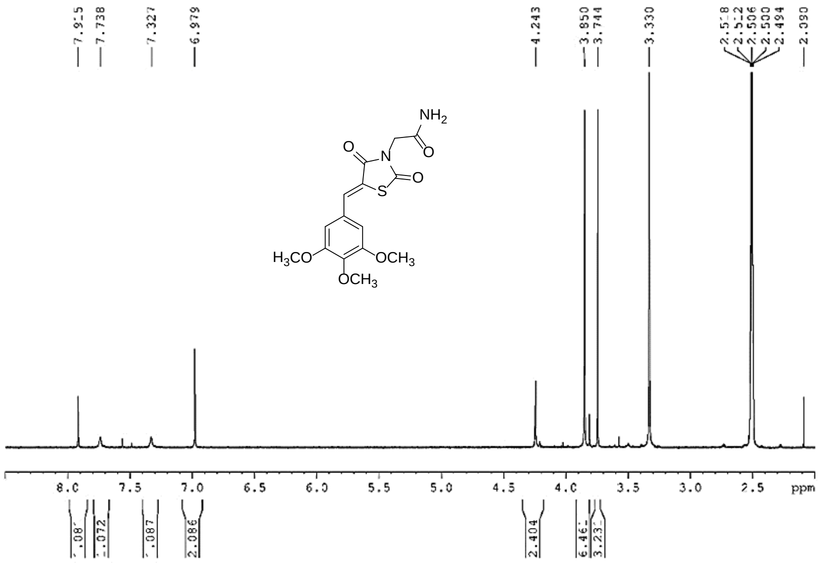


$^{1}$H-NMR spectrum of (*Z*)-2-(5-(3,4,5-trimethoxybenzylidene)thiazolidine-2,4-dion-3-yl)acetamide (**15e**)

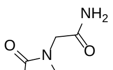

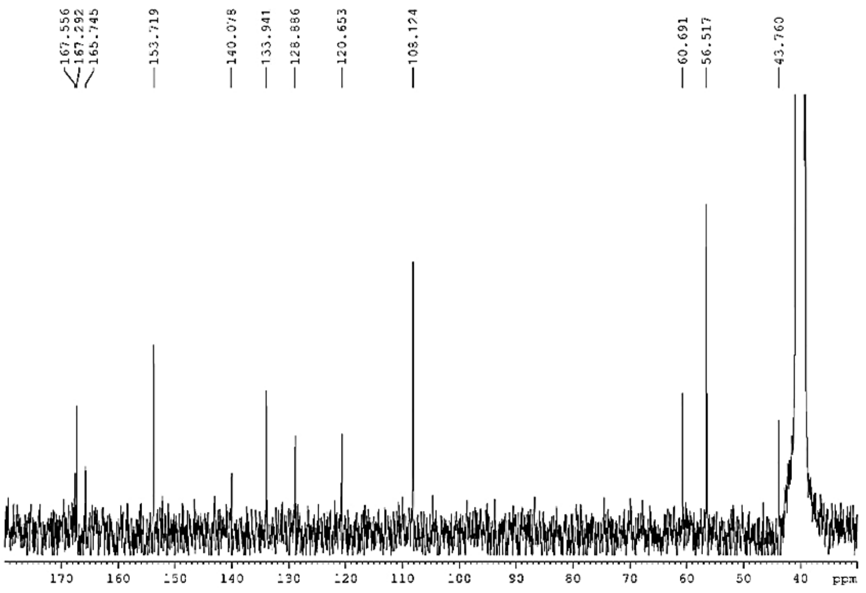


$^{13}$C-NMR spectrum of (*Z*)-2-(5-(3,4,5-trimethoxybenzylidene)thiazolidine-2,4-dion-3-yl)acetamide (**15e**)

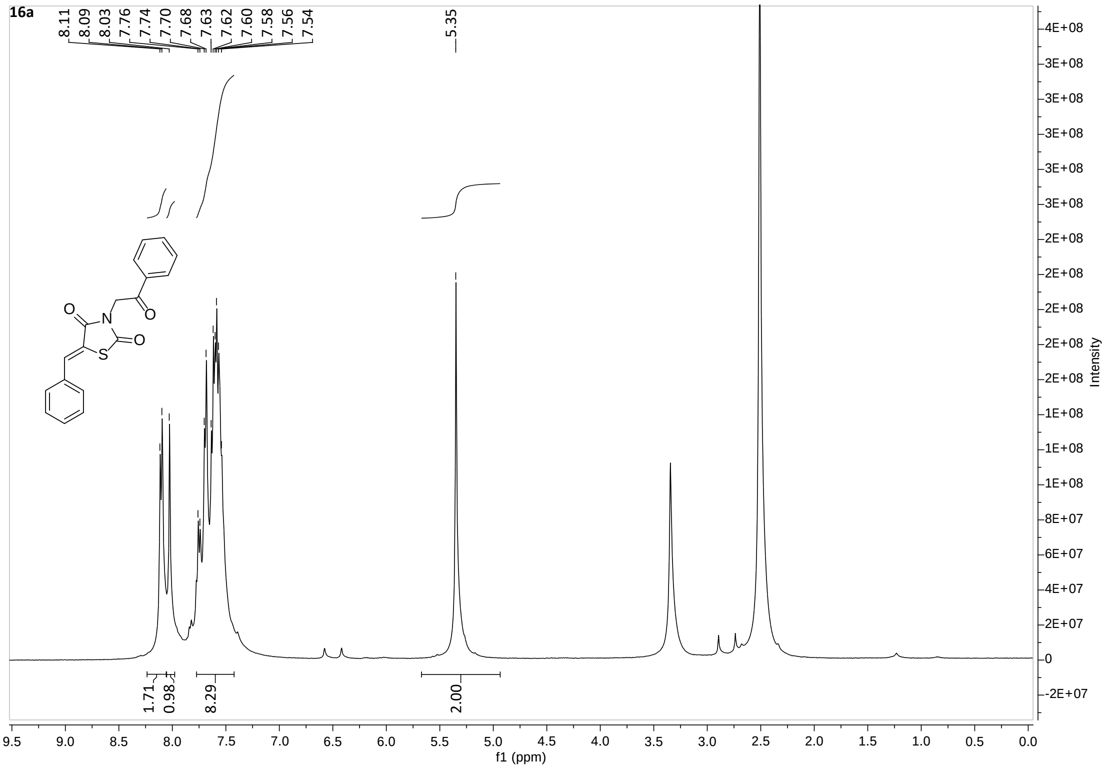


$^{1}$H-NMR spectrum of (*Z*)-5-Benzylidene-3-(2-oxo-2-phenylethyl)thiazolidine-2,4-dione (**16a**)

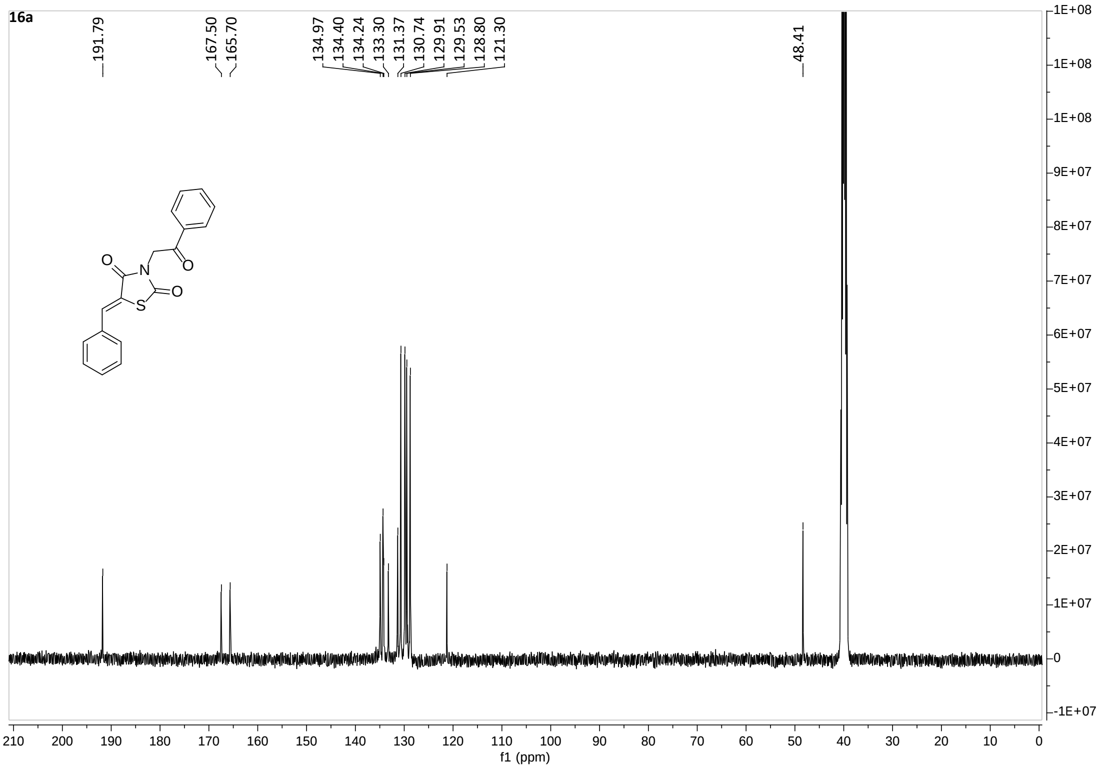


$^{13}$C-NMR spectrum of (*Z*)-5-Benzylidene-3-(2-oxo-2-phenylethyl)thiazolidine-2,4-dione (**16a**)

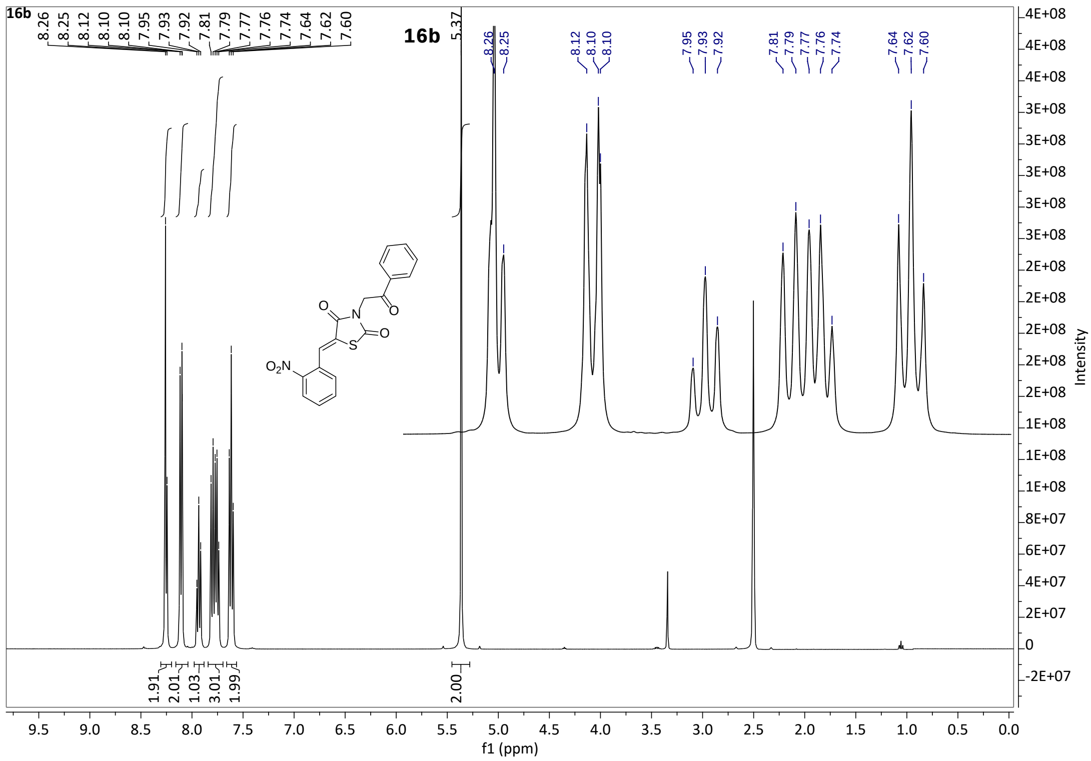

$^{1}$H-NMR spectrum of (*Z*)-5-(2-nitrobenzylidene)-3-(2-oxo-2-phenylethyl) thiazolidine-2,4-dione (**16b**)

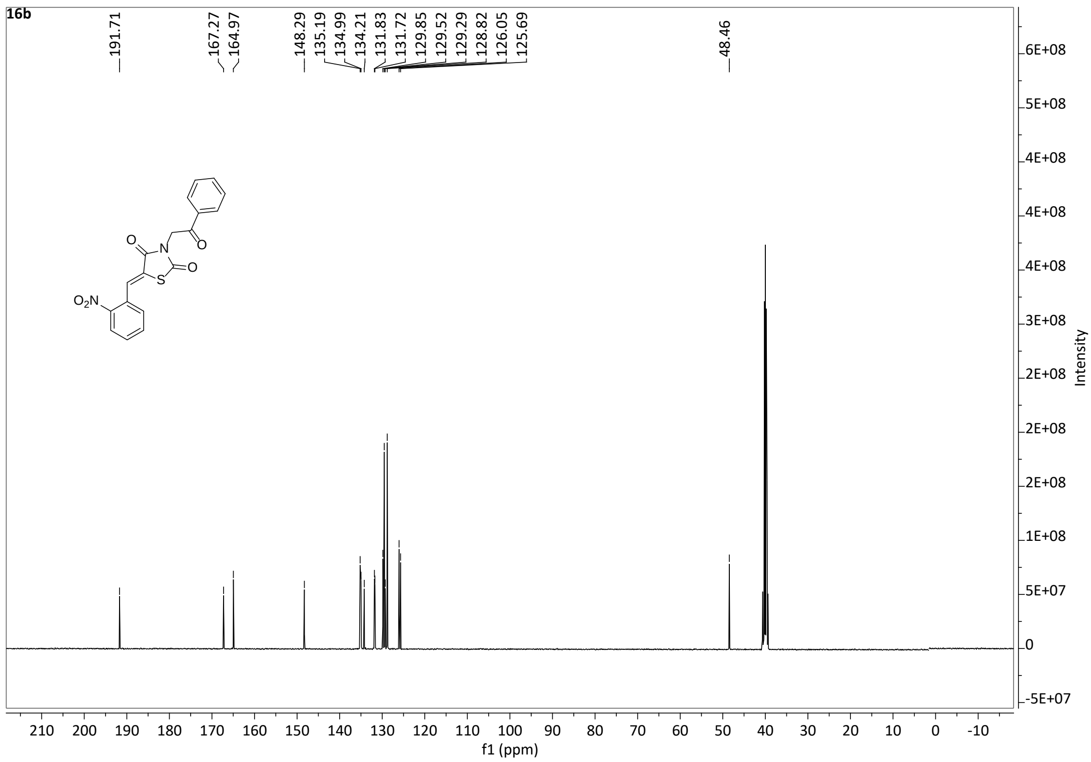

$^{13}$C-NMR spectrum of (*Z*)-5-(2-nitrobenzylidene)-3-(2-oxo-2-phenylethyl) thiazolidine-2,4-dione (**16b**)

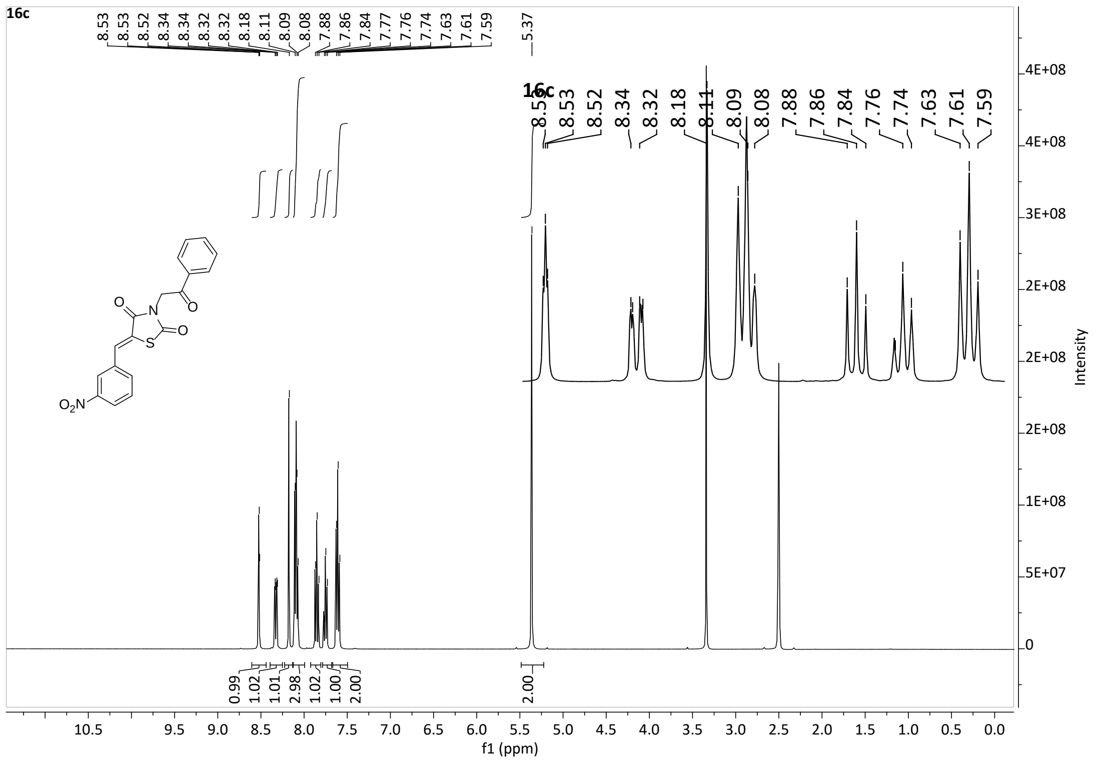

$^{1}$H-NMR spectrum of (*Z*)-5-(3-nitrobenzylidene)-3-(2-oxo-2-phenylethyl) thiazolidine-2,4-dione (**16c**)

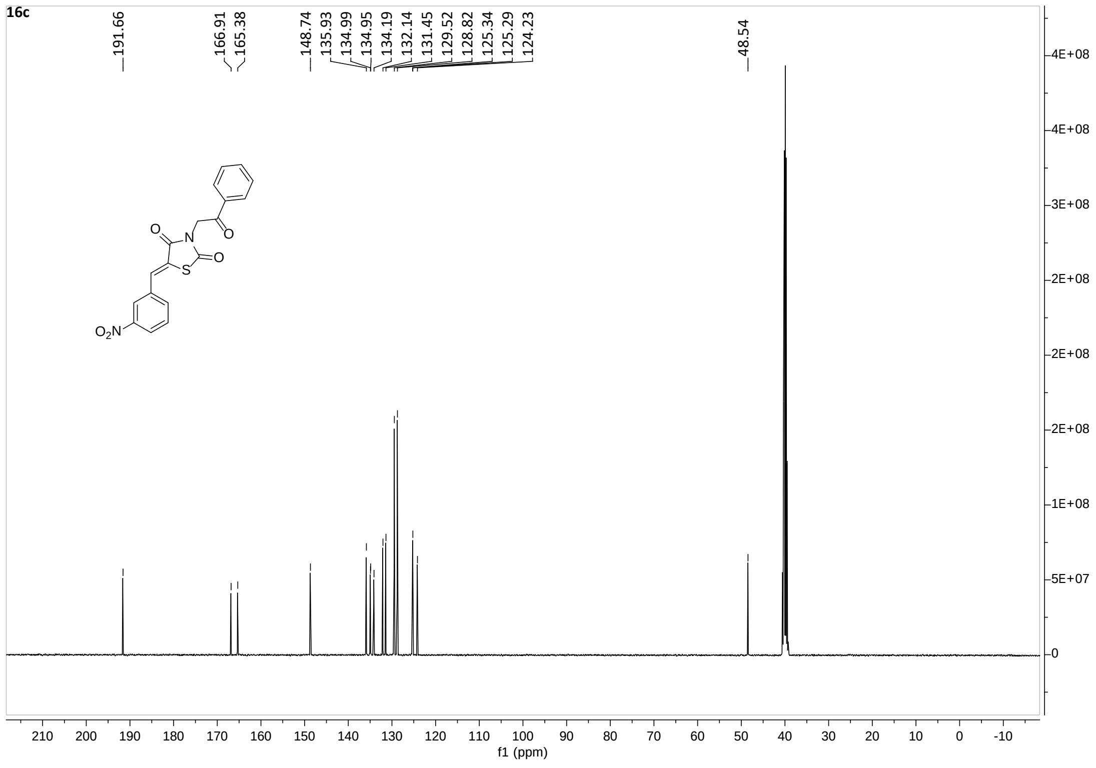

$^{13}$C-NMR spectrum of (*Z*)-5-(3-nitrobenzylidene)-3-(2-oxo-2-phenylethyl) thiazolidine-2,4-dione (**16c**)

$^{1}$H-NMR spectrum of (*Z*)-5-(4-nitrobenzylidene)-3-(2-oxo-2-phenylethyl) thiazolidine-2,4-dione (**16d**)

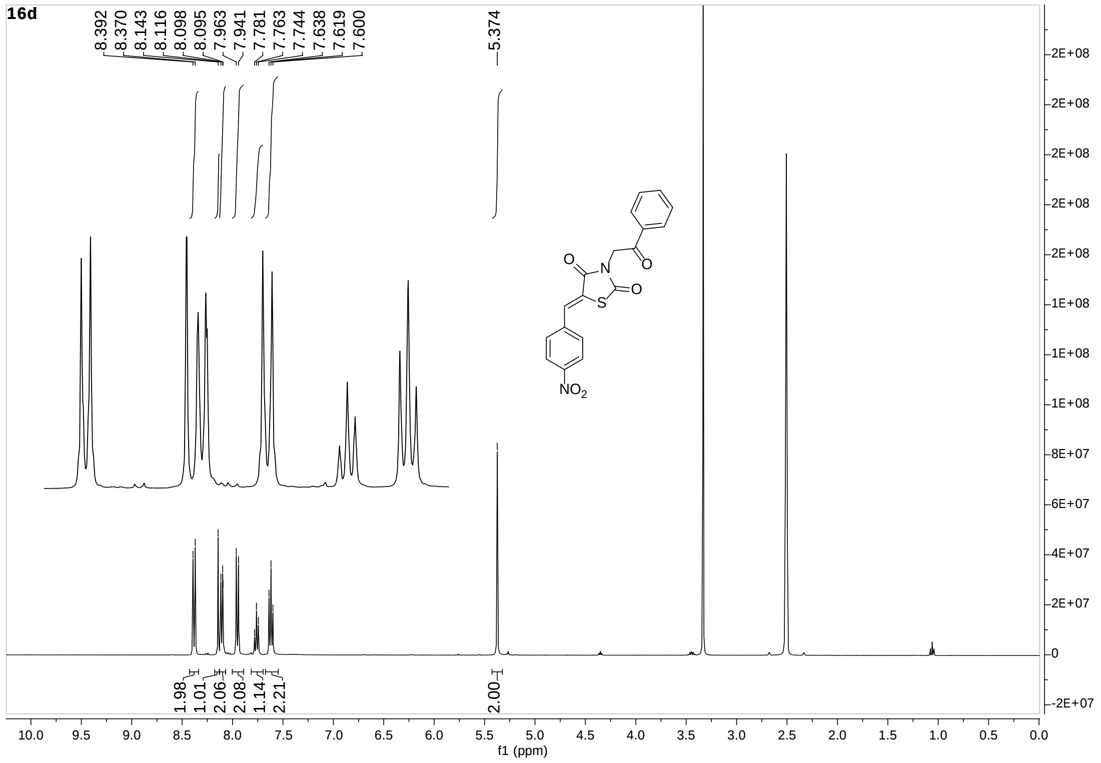


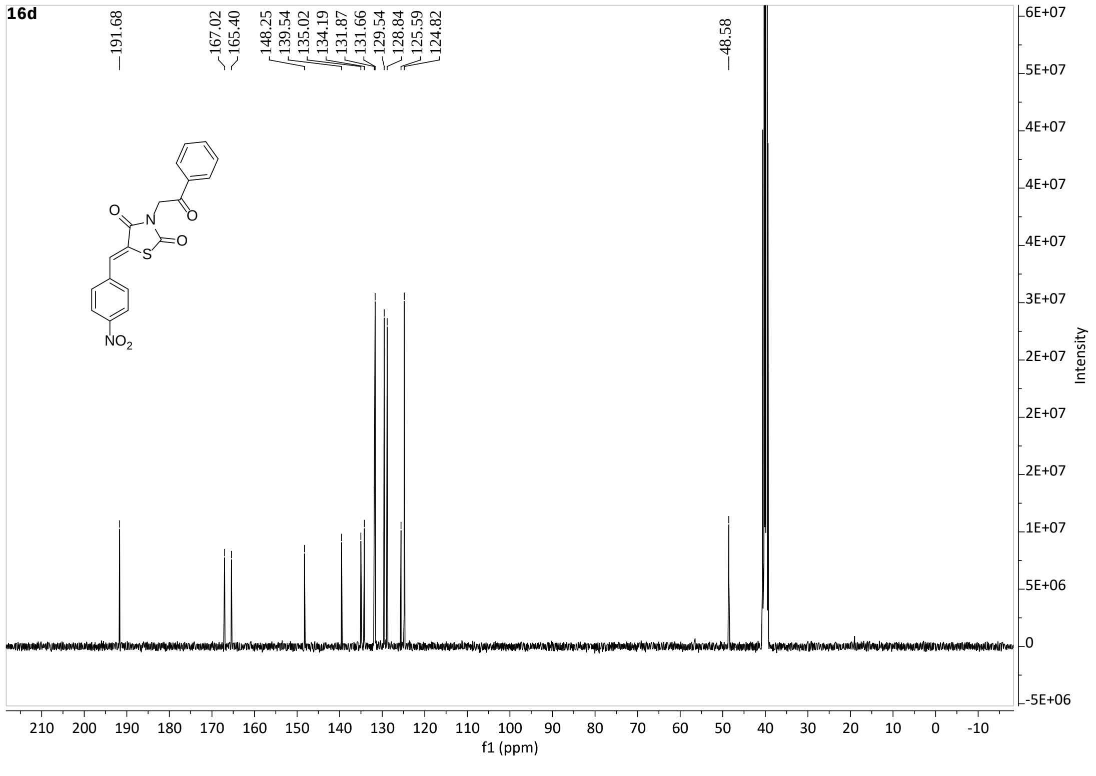


$^{13}$C-NMR spectrum of (*Z*)-5-(4-nitrobenzylidene)-3-(2-oxo-2-phenylethyl) thiazolidine-2,4-dione (**16d**)

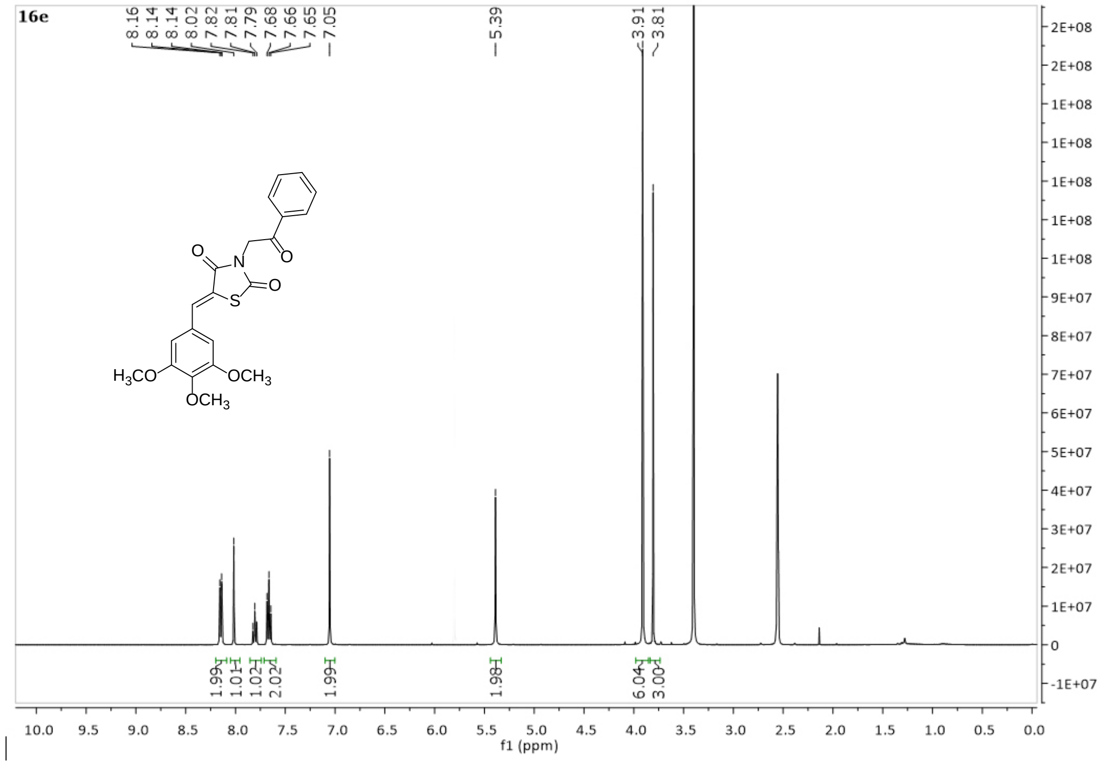


$^{1}$H-NMR spectrum of (Z)-3-(2-Oxo-2-phenylethyl)-5-(3,4,5-trimethoxybenzylidene) thiazolidine-2,4-dione (**16e**)

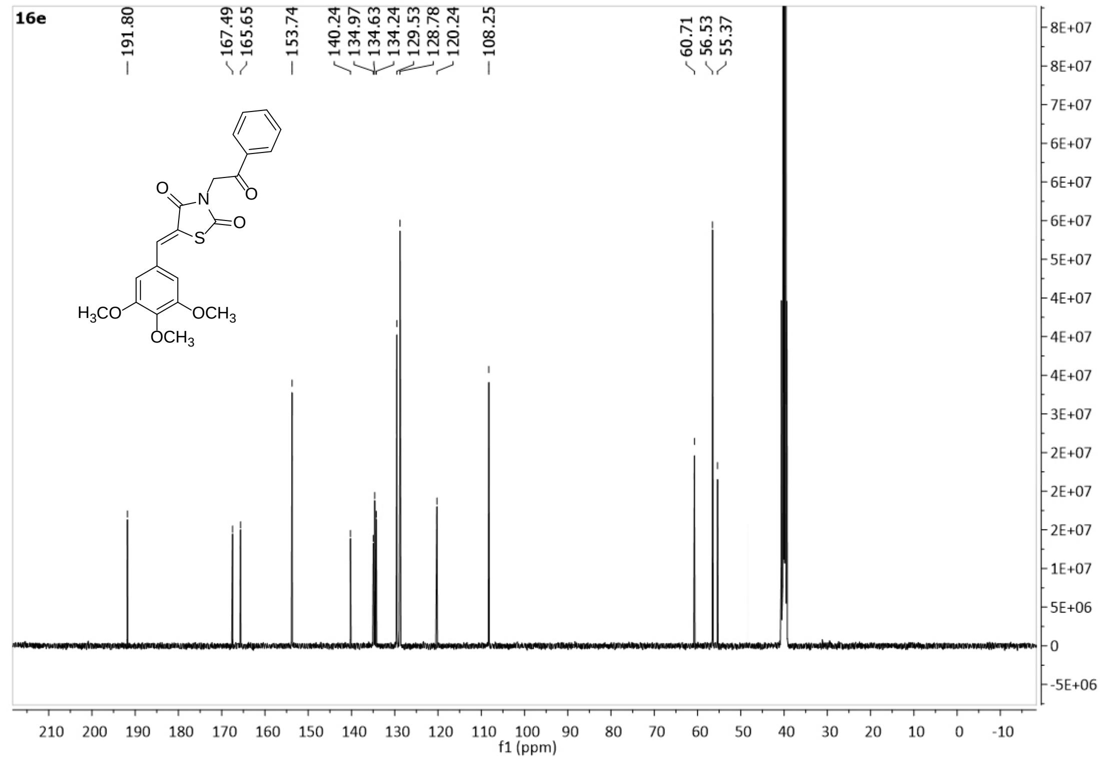


$^{13}$C-NMR spectrum of (Z)-3-(2-Oxo-2-phenylethyl)-5-(3,4,5-trimethoxybenzylidene) thiazolidine-2,4-dione (**16e**)

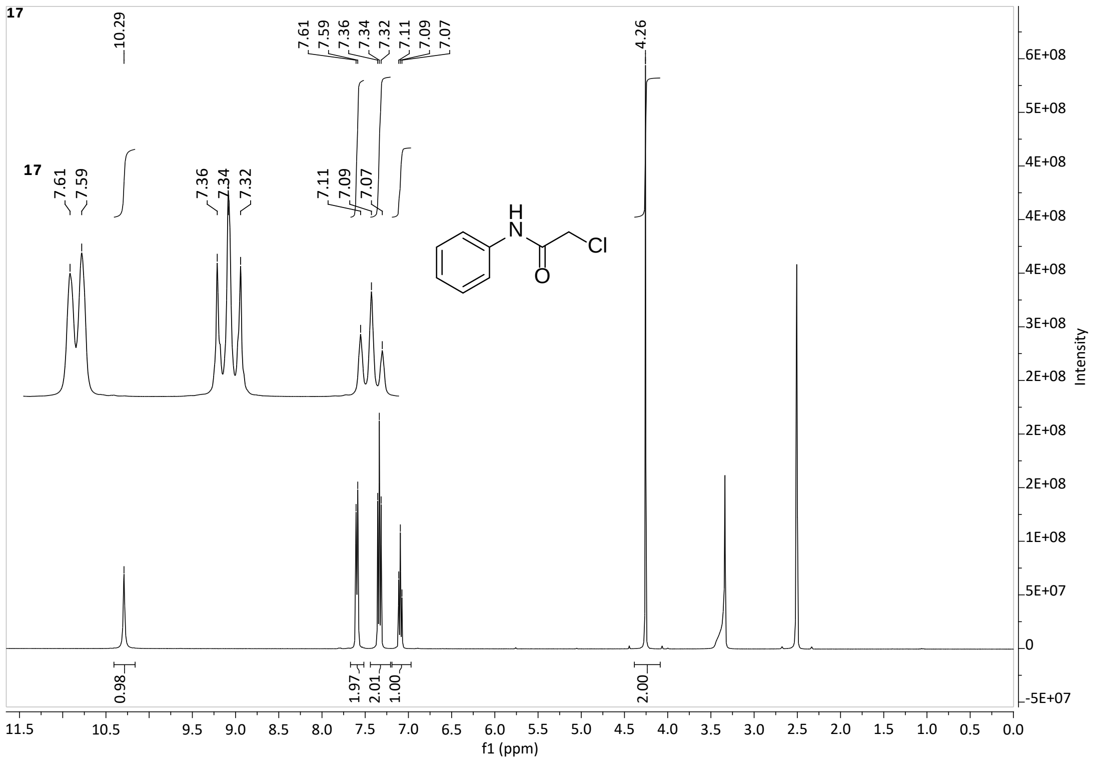


$^{1}$H-NMR spectrum of 2-chloro-*N*-phenylacetamide (**17**)

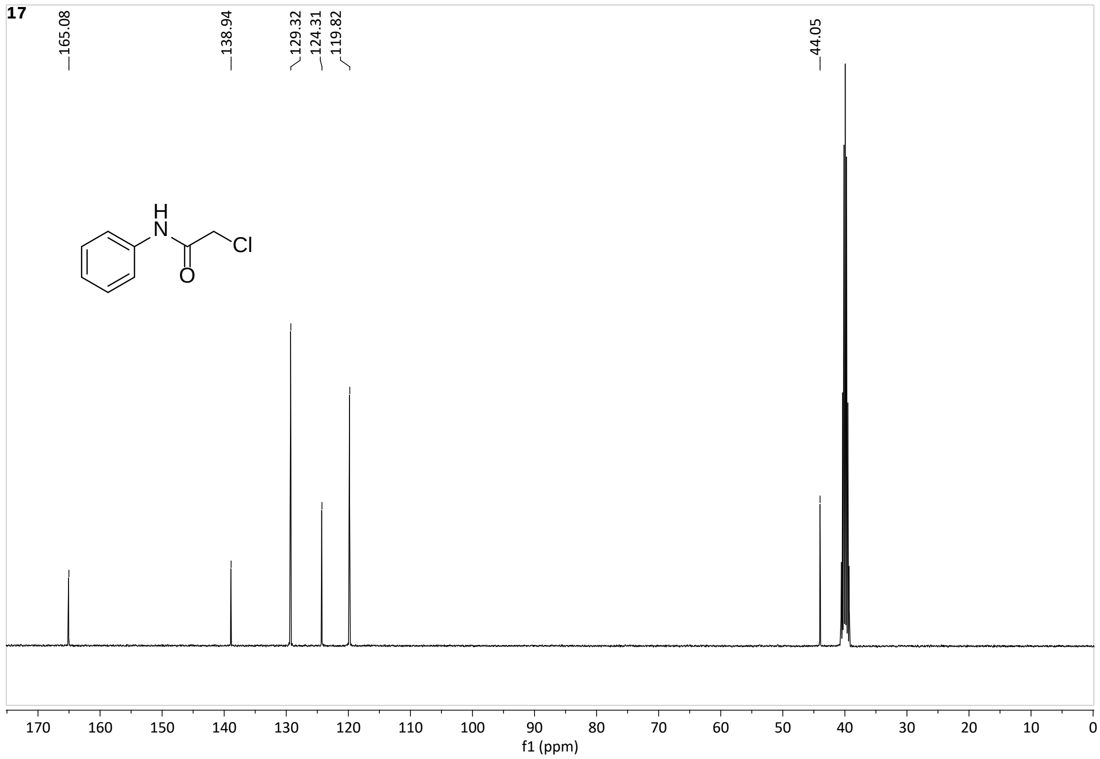


$^{13}$C-NMR spectrum of 2-chloro-*N*-phenylacetamide (**17**)

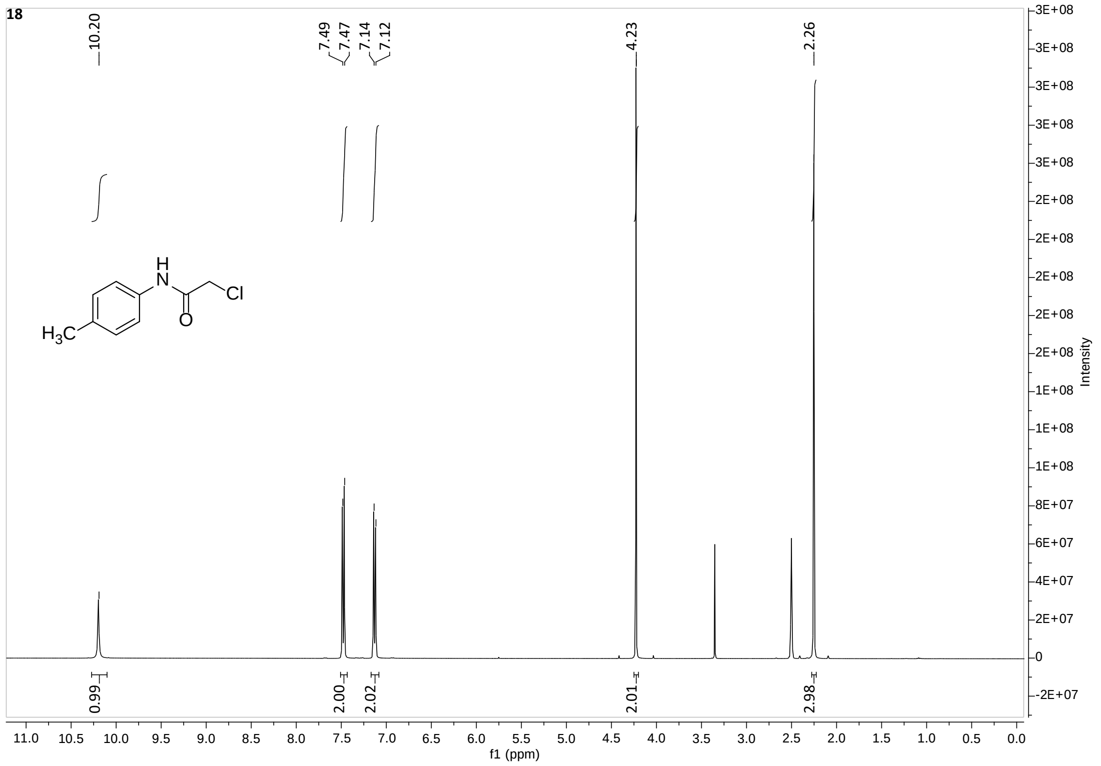


$^{1}$H-NMR spectrum of 2-chloro-*N*-(4-methylphenyl)acetamide (**18**)

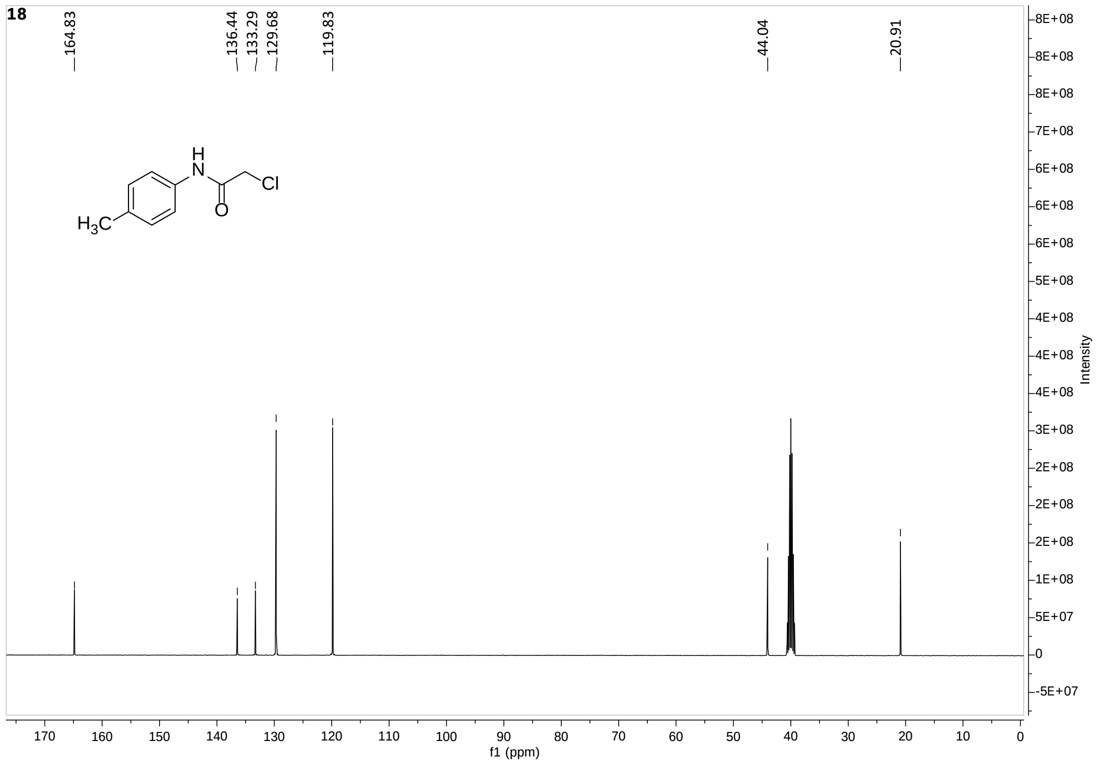


$^{13}$C-NMR spectrum of 2-chloro-*N*-(4-methylphenyl)acetamide (**18**)

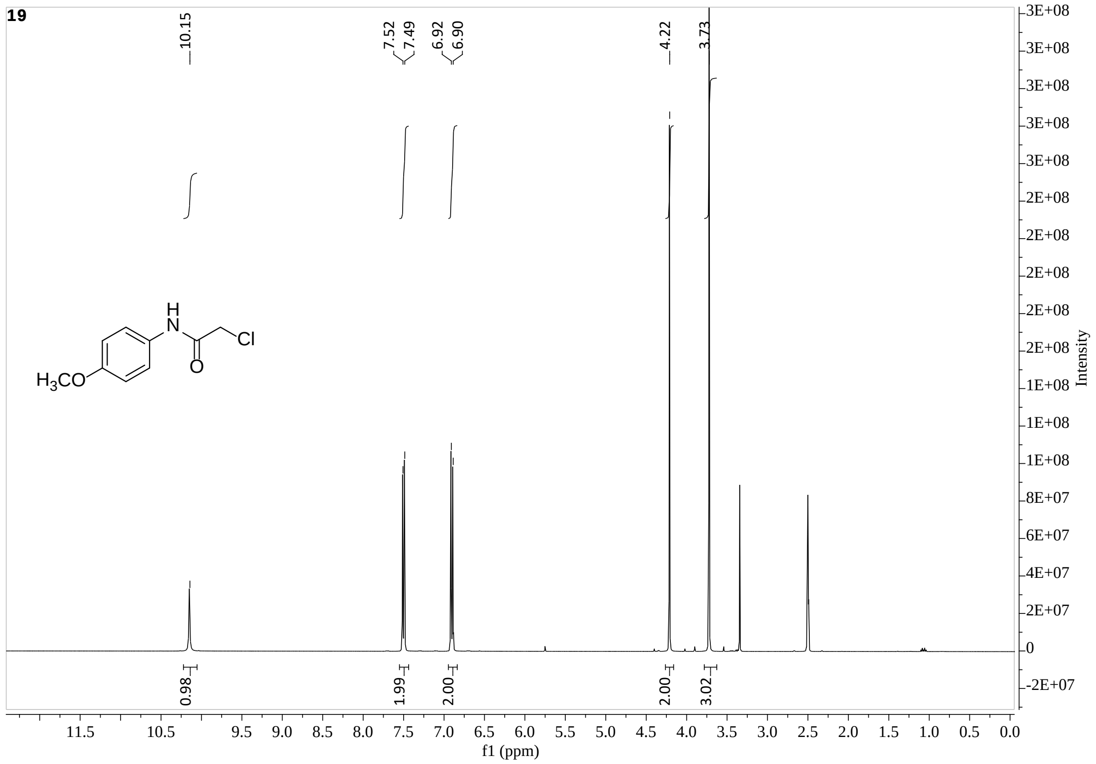


$^{1}$H-NMR spectrum of 2-chloro-*N*-(4-methoxylphenyl)acetamide (**19**)

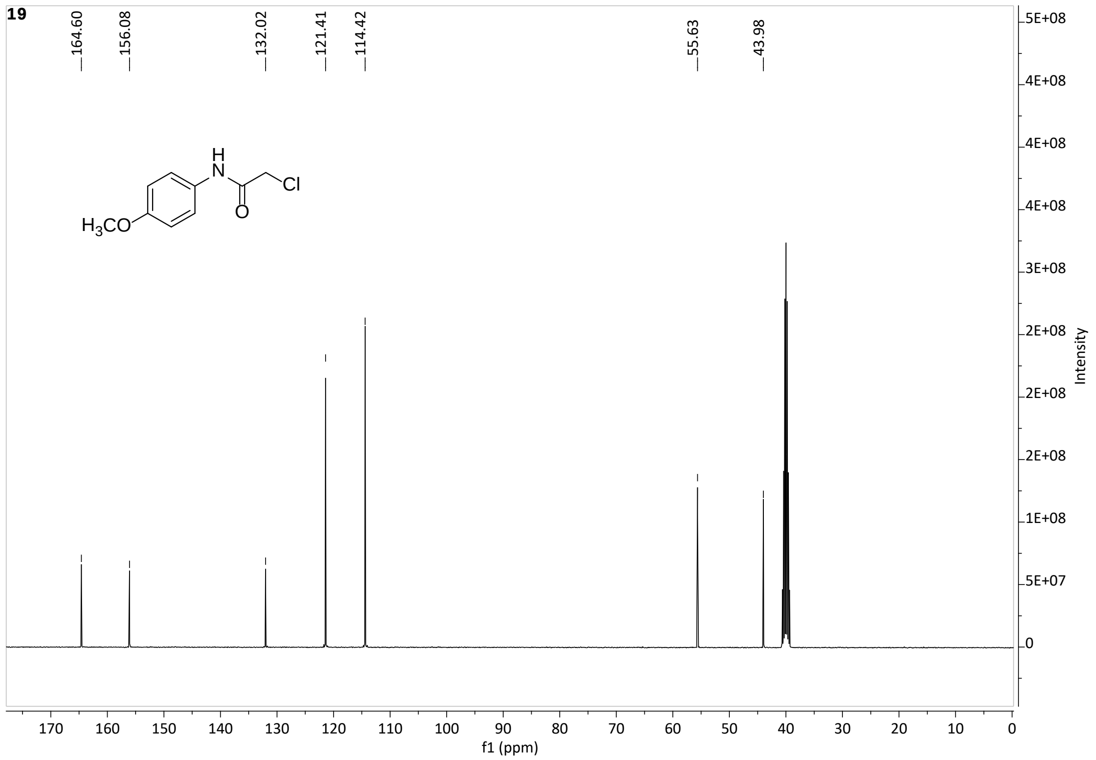


$^{13}$C-NMR spectrum of 2-chloro-*N*-(4-methoxylphenyl)acetamide (**19**)

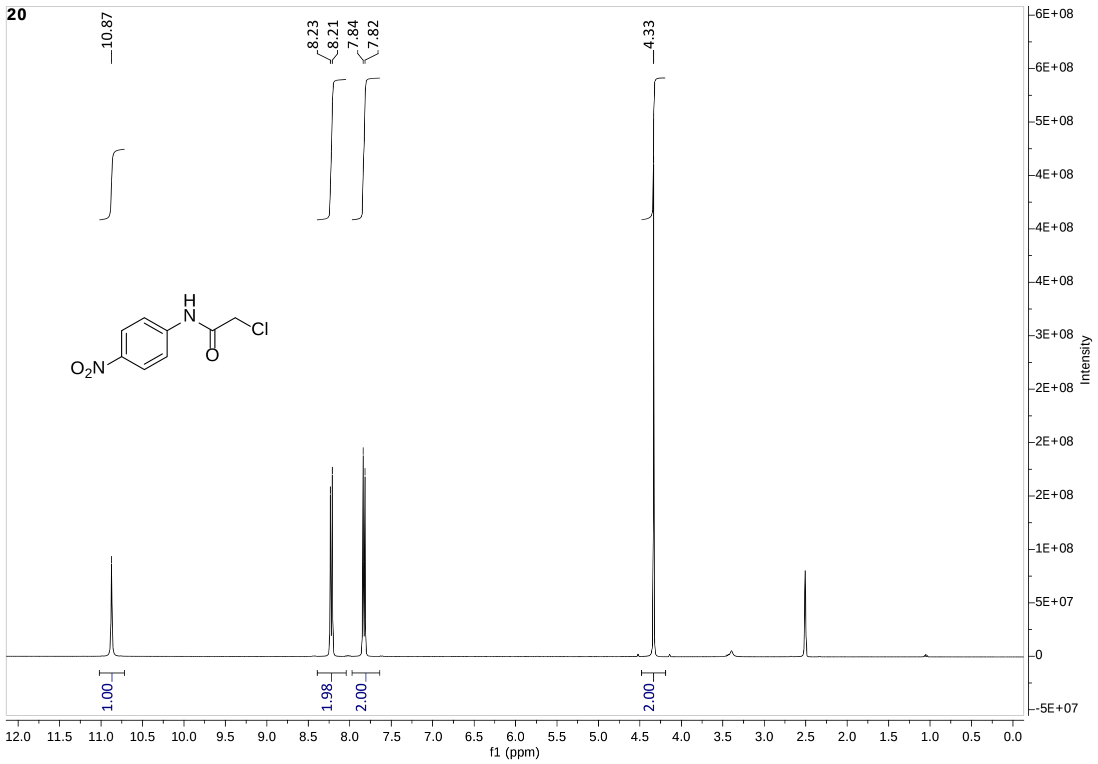


$^{1}$H-NMR spectrum of 2-chloro-*N*-(4-nitrolphenyl)acetamide (**20**)

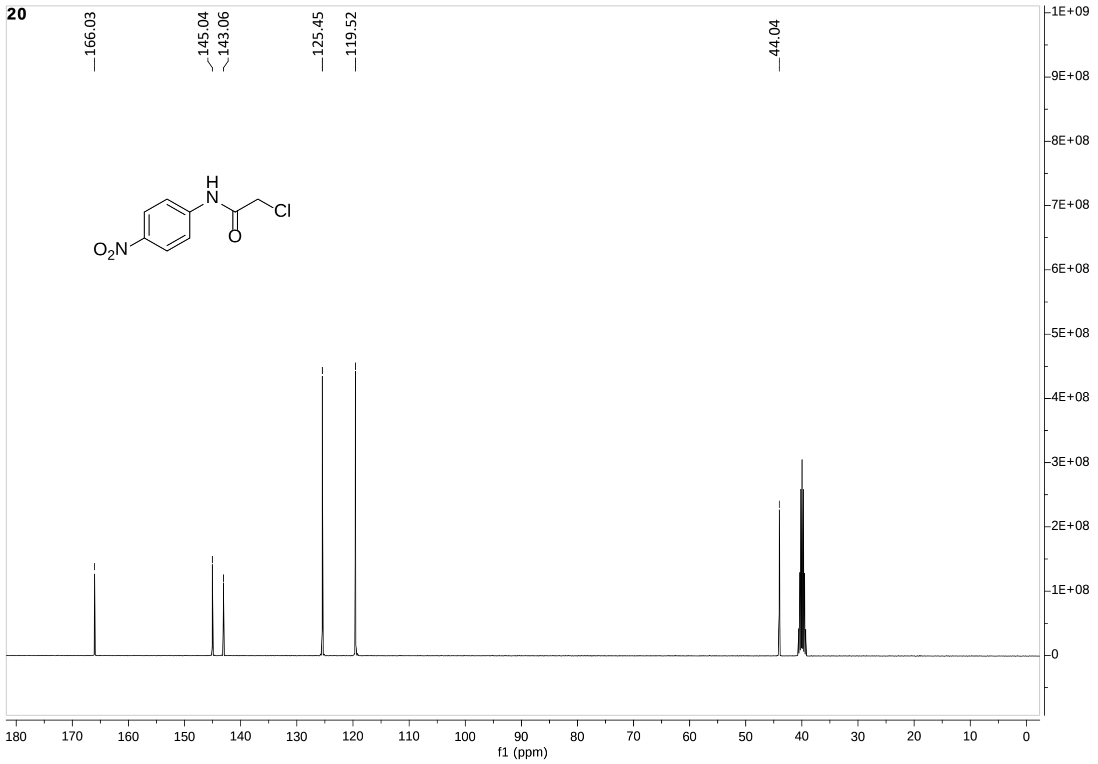


$^{13}$C-NMR spectrum of 2-chloro-*N*-(4-nitrolphenyl)acetamide (**20**)

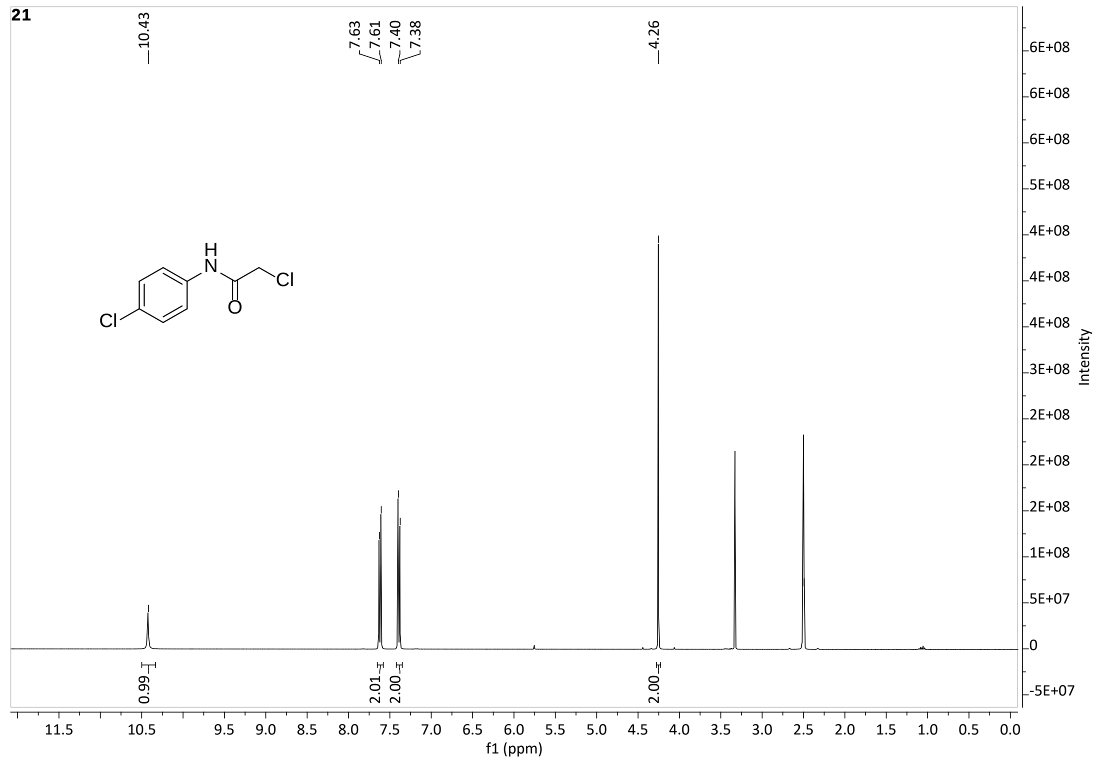


$^{1}$H-NMR spectrum of 2-chloro-*N*-(4-chlorophenyl)acetamide (**21**)

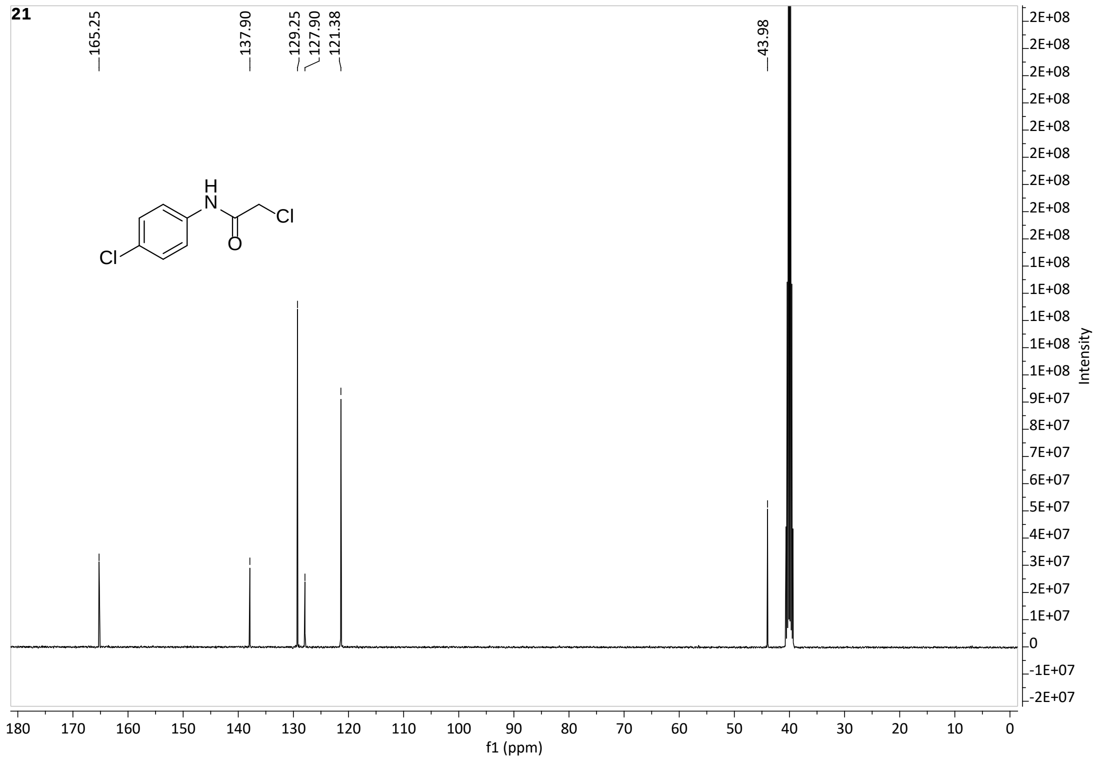


$^{13}$C-NMR spectrum of 2-chloro-*N*-(4-chlorophenyl)acetamide (**21**)

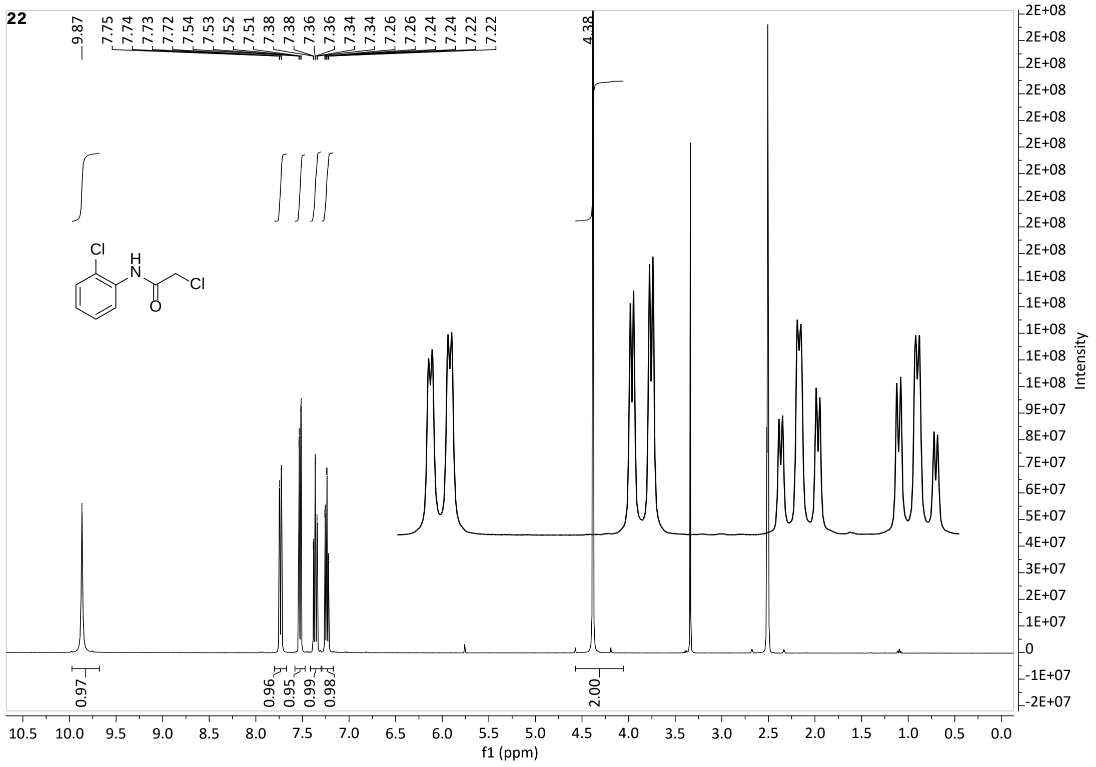


$^{1}$H-NMR spectrum of 2-chloro-*N*-(2-chlorophenyl)acetamide (**22**)

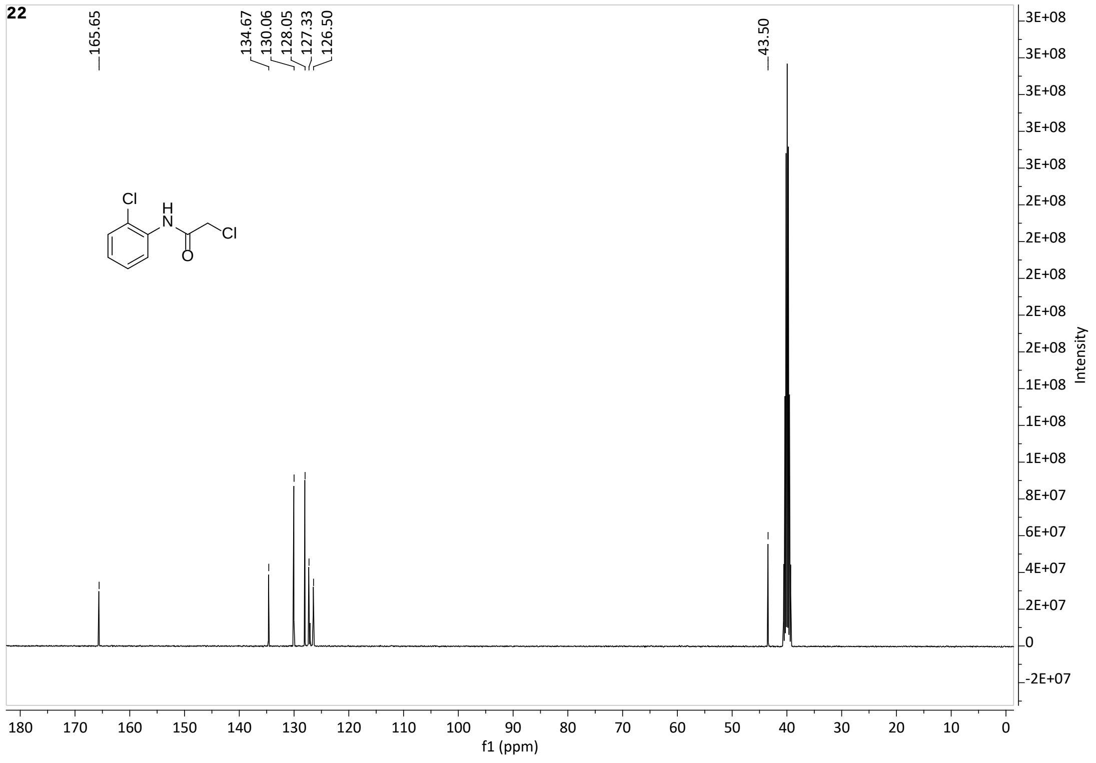


$^{13}$C-NMR spectrum of 2-chloro-*N*-(2-chlorophenyl)acetamide (**22**)

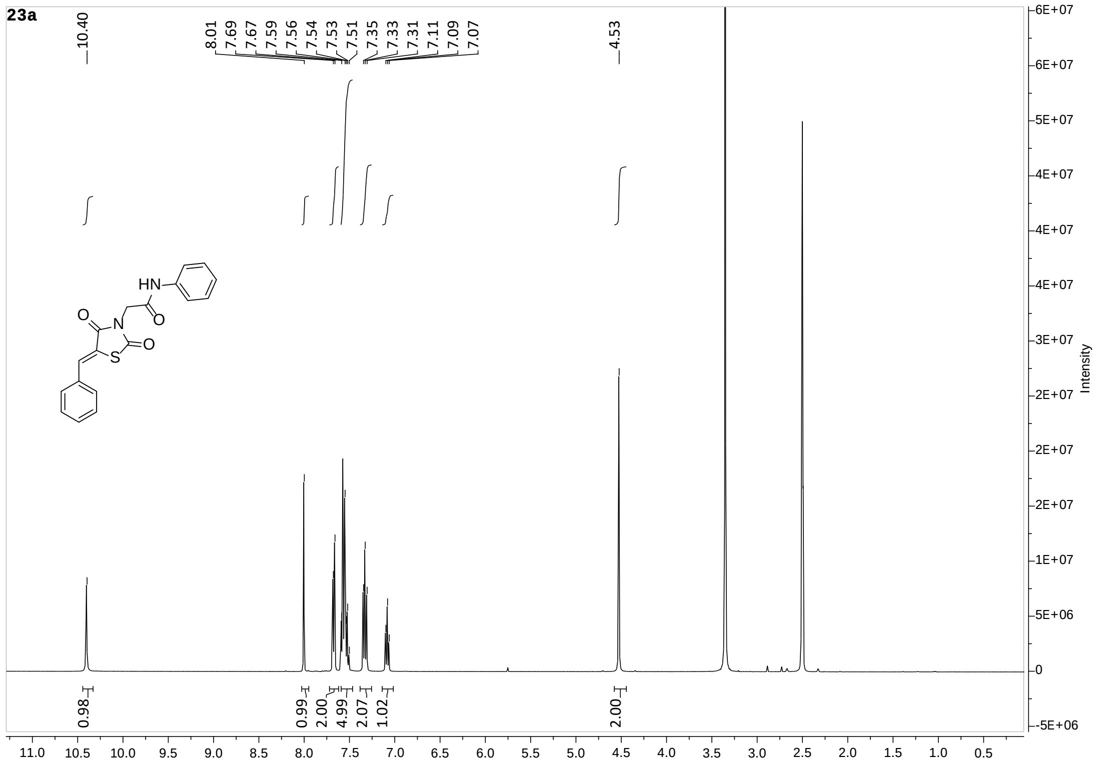


$^{1}$H-NMR spectrum of (*Z*)-2-(5-benzylidenethiazolidine-2,4-dion-3-yl)-*N*-phenyl acetamide (**23a**)

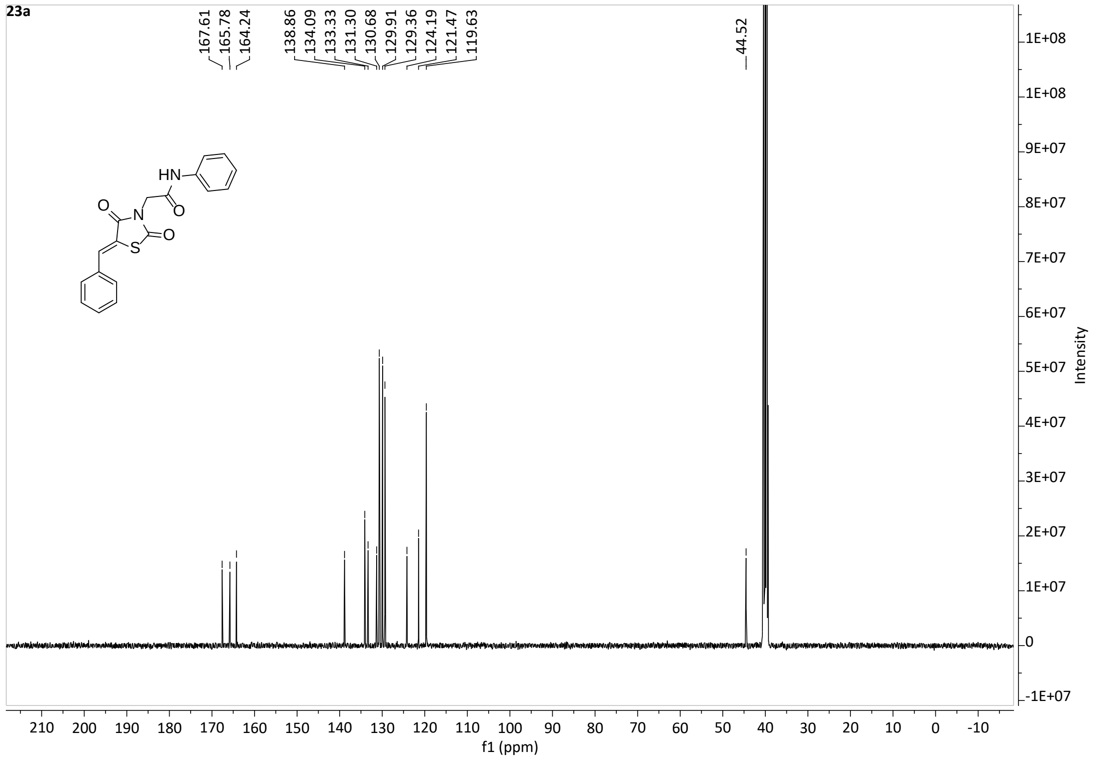


$^{13}$C-NMR spectrum of (*Z*)-2-(5-benzylidenethiazolidine-2,4-dion-3-yl)-*N*-phenyl acetamide (**23a**)

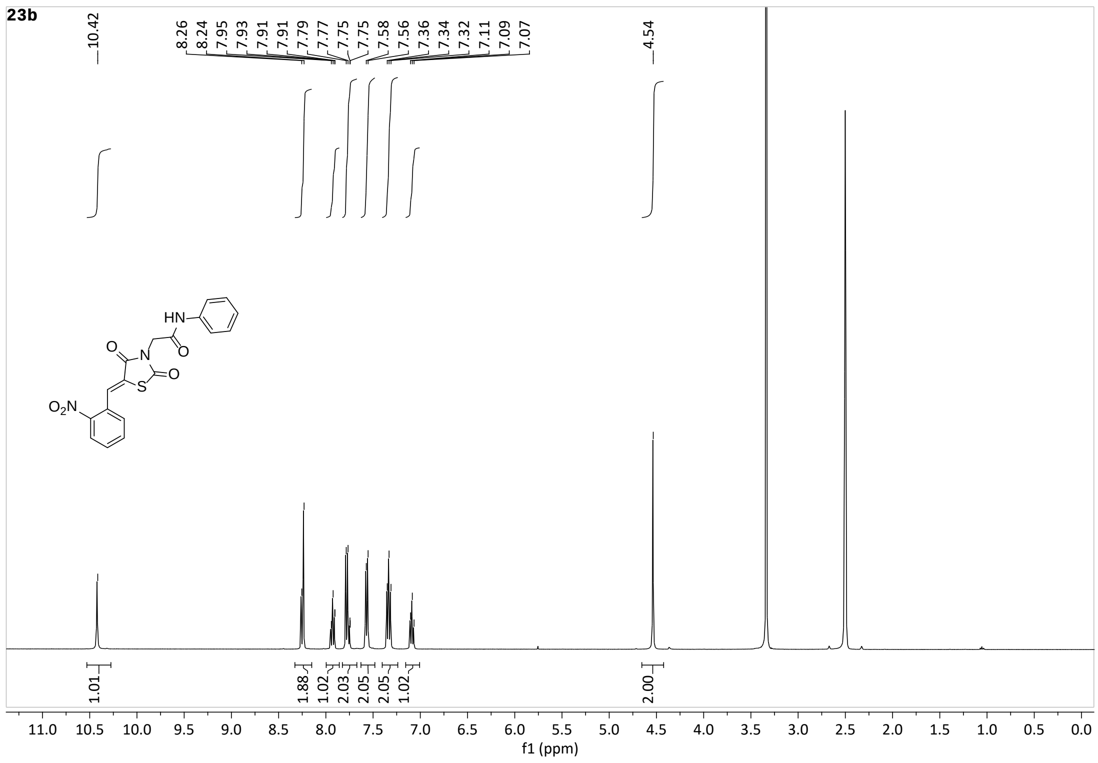


$^{1}$H-NMR spectrum of (*Z*)-2-(5-(2-nitrobenzylidene)thiazolidine-2,4-dion-3-yl)-*N*-phenylacetamide (**23b**)

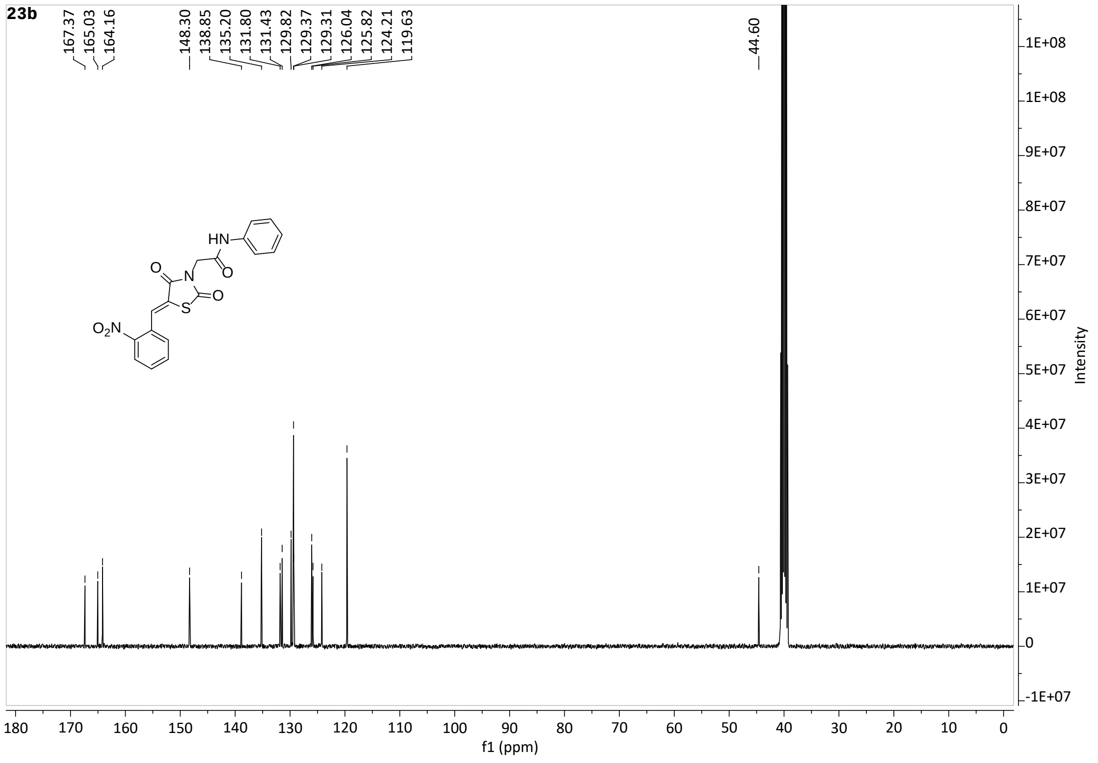


$^{13}$C-NMR spectrum of (*Z*)-2-(5-(2-nitrobenzylidene)thiazolidine-2,4-dion-3-yl)-*N*-phenylacetamide (**23b**)

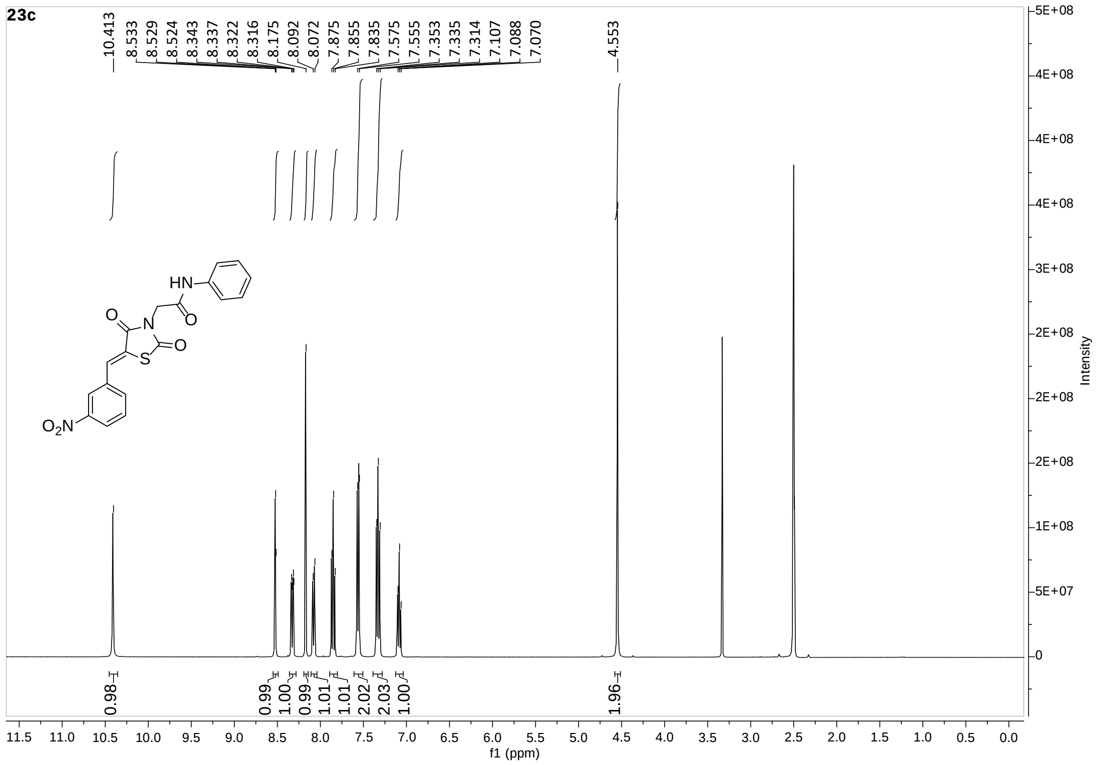


$^{1}$H-NMR spectrum of (*Z*)-2-(5-(3-nitrobenzylidene)thiazolidine-2,4-dion-3-yl)-*N*-phenylacetamide (**23c**)

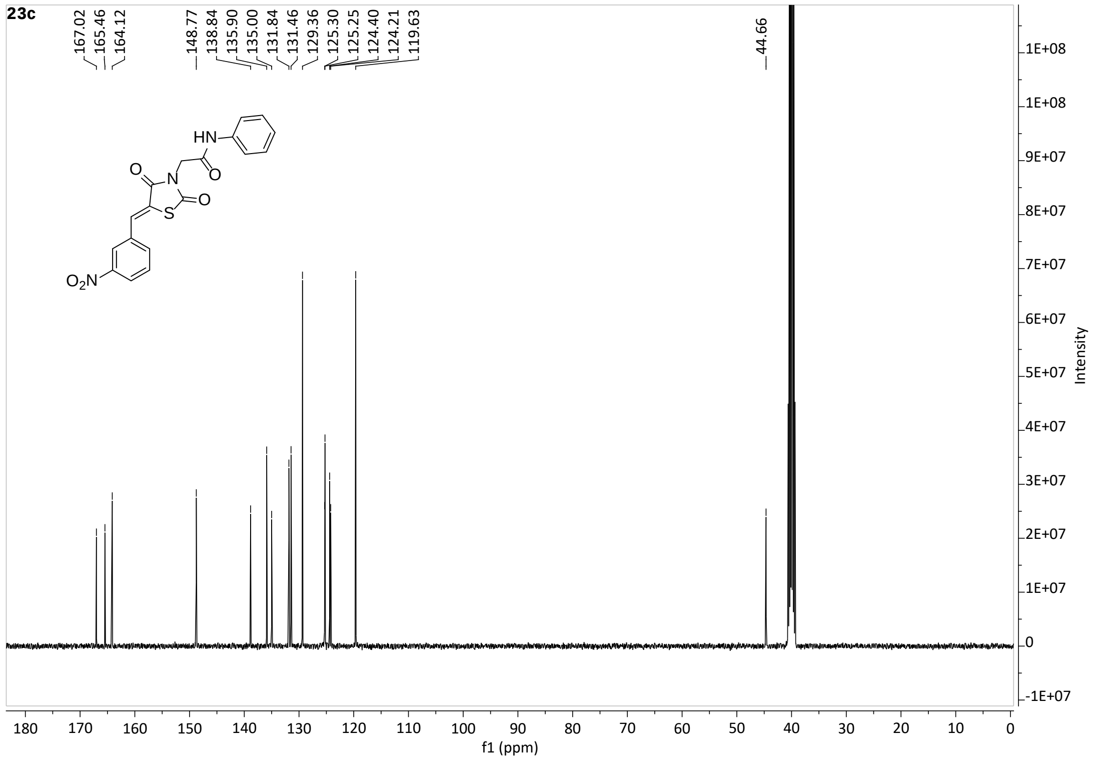


$^{13}$C-NMR spectrum of (*Z*)-2-(5-(3-nitrobenzylidene)thiazolidine-2,4-dion-3-yl)-*N*-phenylacetamide (**23c**)

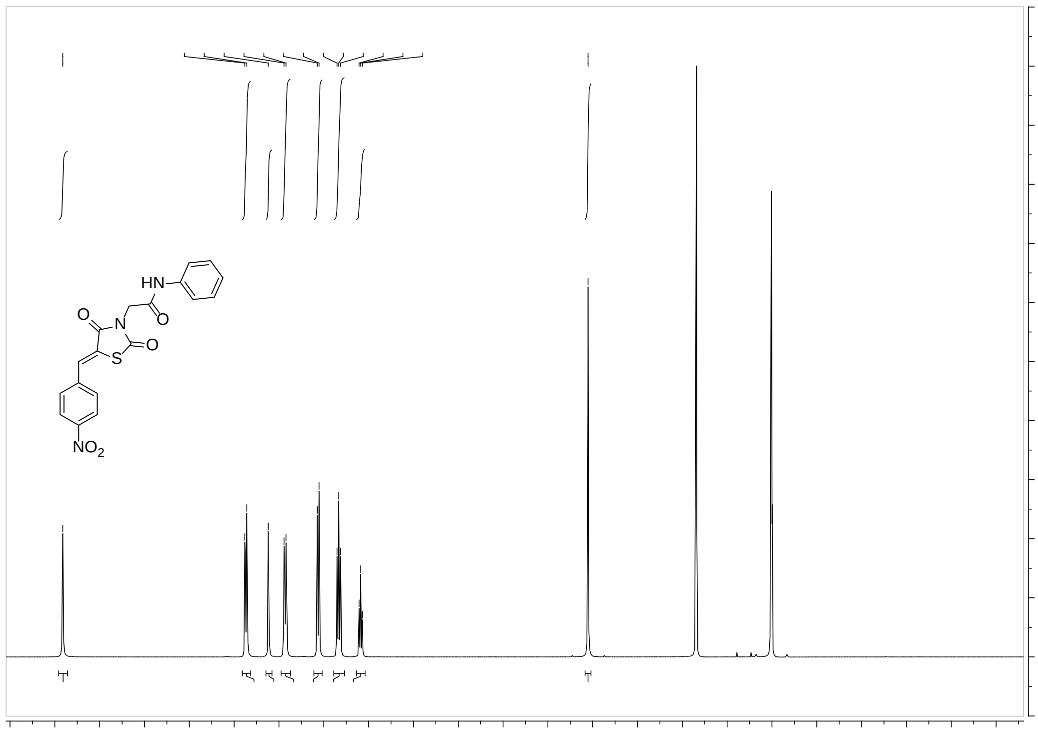


$^{1}$H-NMR spectrum of (*Z*)-2-(5-(4-nitrobenzylidene)thiazolidine-2,4-dion-3-yl)-*N*-phenylacetamide (**23d**)

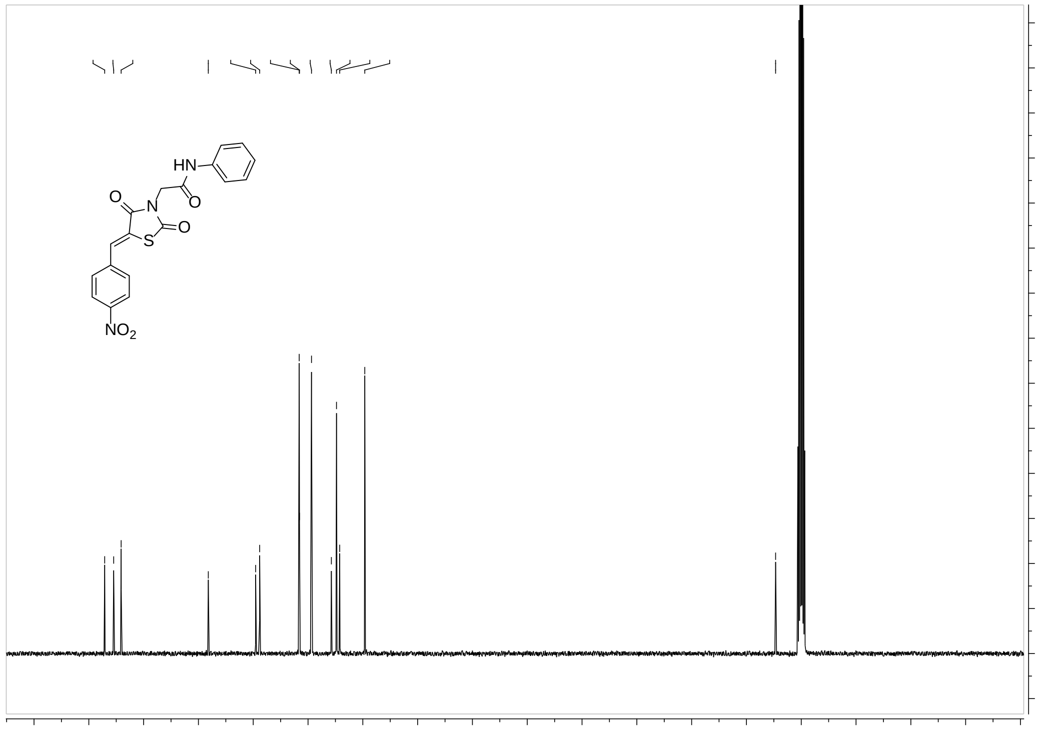


$^{13}$C-NMR spectrum of (*Z*)-2-(5-(4-nitrobenzylidene)thiazolidine-2,4-dion-3-yl)-*N*-phenylacetamide (**23d**)

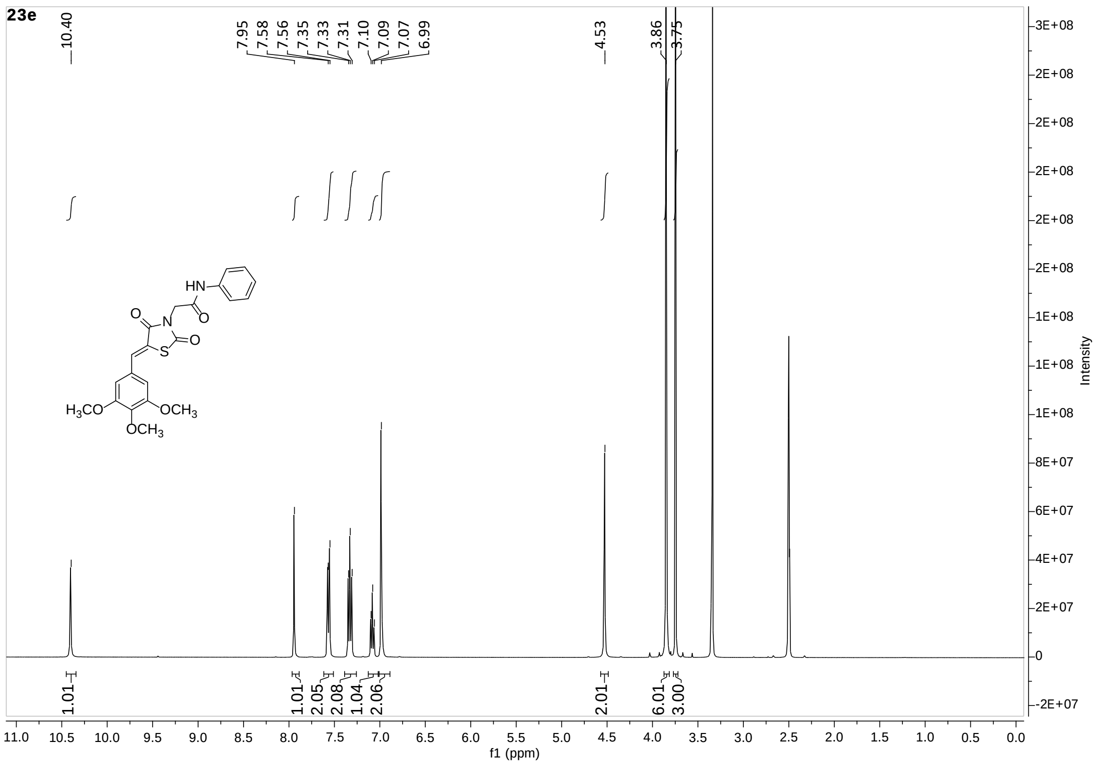


$^{1}$H-NMR spectrum of *(Z)*-*N*-phenyl-2-(5-(3,4,5-trimethoxybenzylidene)thiazolidine-2,4-dion-3-yl)acetamide (**23e**)

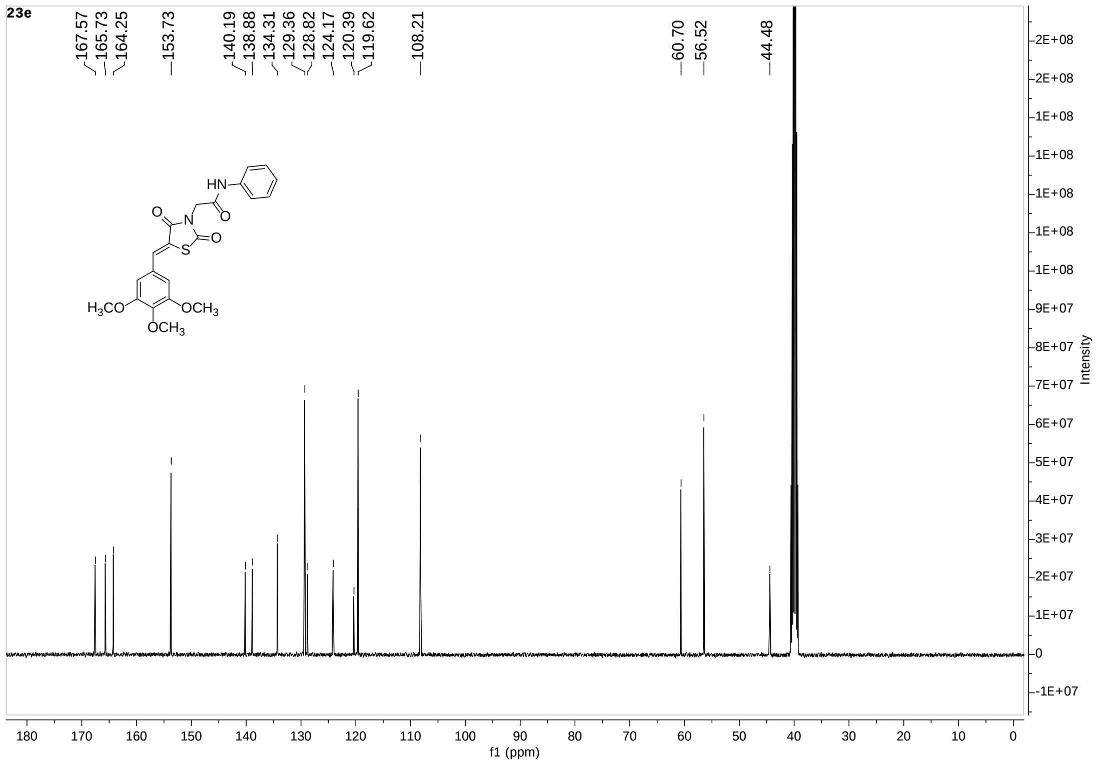


$^{13}$C-NMR spectrum of *(Z)*-*N*-phenyl-2-(5-(3,4,5-trimethoxybenzylidene)thiazolidine-2,4-dion-3-yl)acetamide (**23e**)

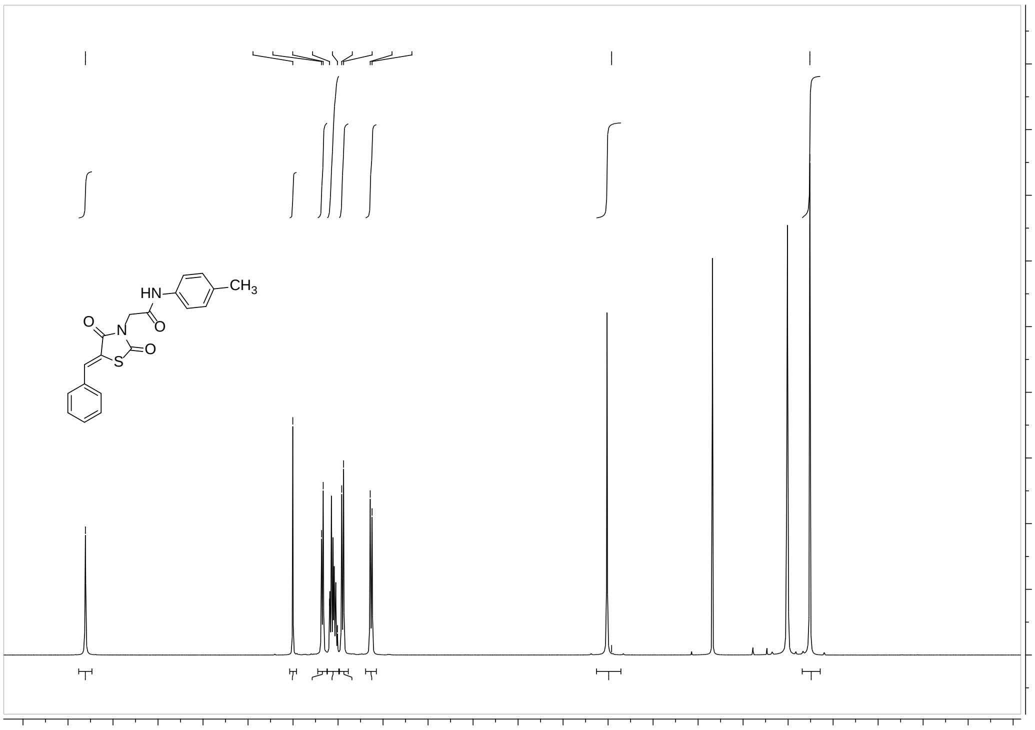


$^{1}$H-NMR spectrum of (*Z*)-2-(5-benzylidenethiazolidine-2,4-dion-3-yl)-N-(4-methylphenyl)acetamide (**24a**)

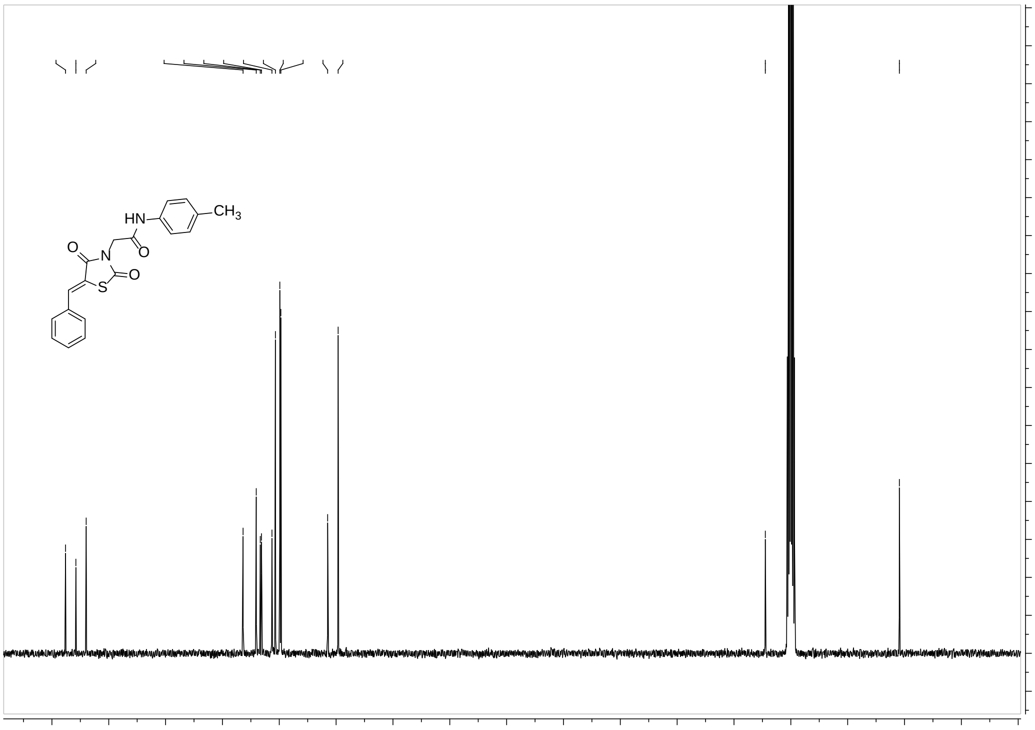


$^{13}$C-NMR spectrum of (*Z*)-2-(5-benzylidenethiazolidine-2,4-dion-3-yl)-N-(4-methylphenyl)acetamide (**24a**)

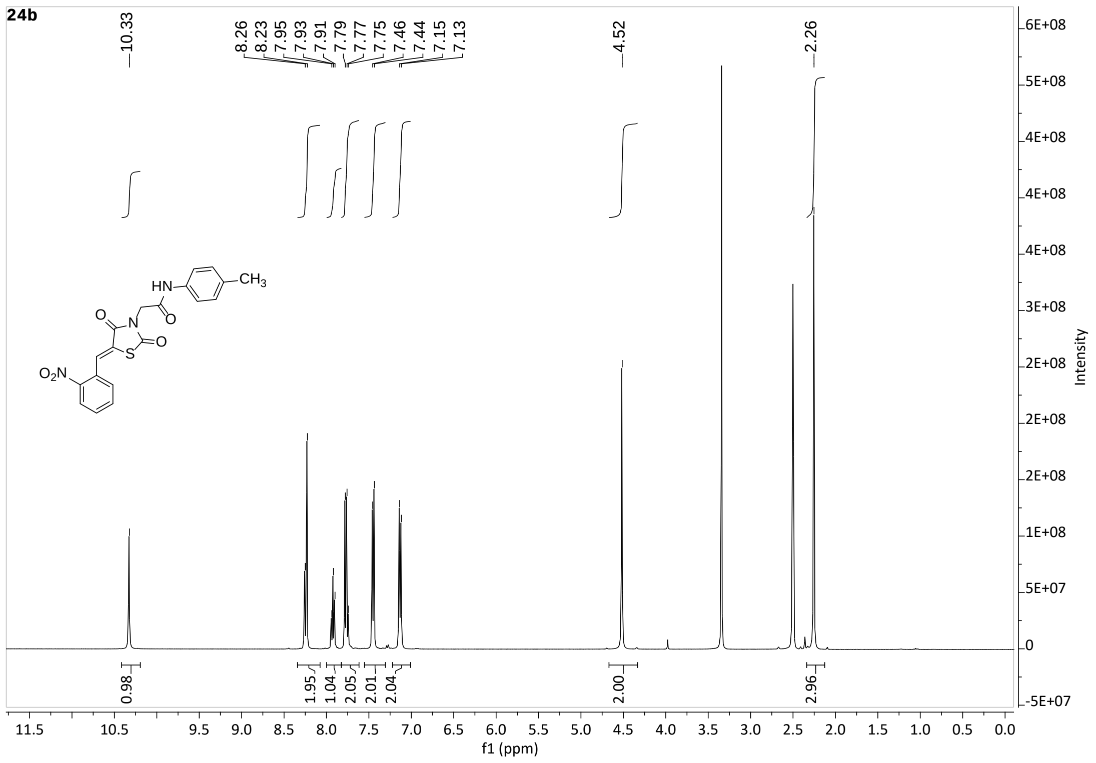


$^{1}$H-NMR spectrum of (*Z*)-*N*-(4-methylphenyl)2-(5-(2-nitrobenzylidene)thiazolidine-2,4-dion-3-yl)acetamide (**24b**)

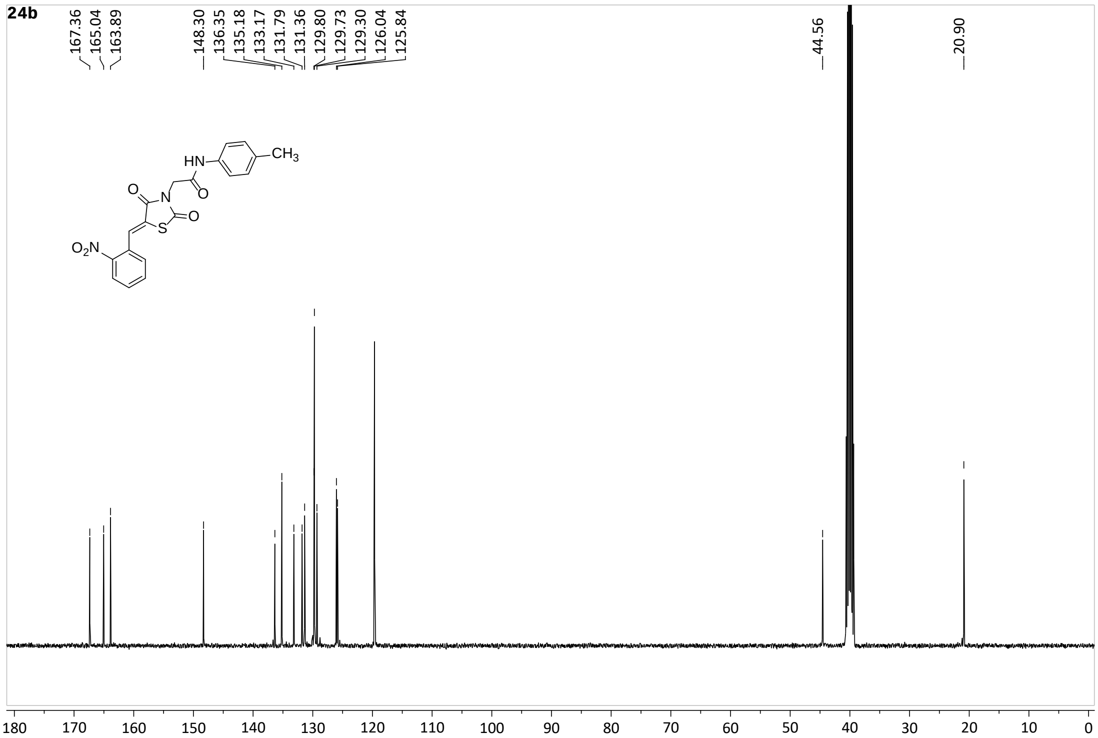


$^{13}$C-NMR spectrum of (*Z*)-*N*-(4-methylphenyl)2-(5-(2-nitrobenzylidene)thiazolidine-2,4-dion-3-yl)acetamide (**24b**)

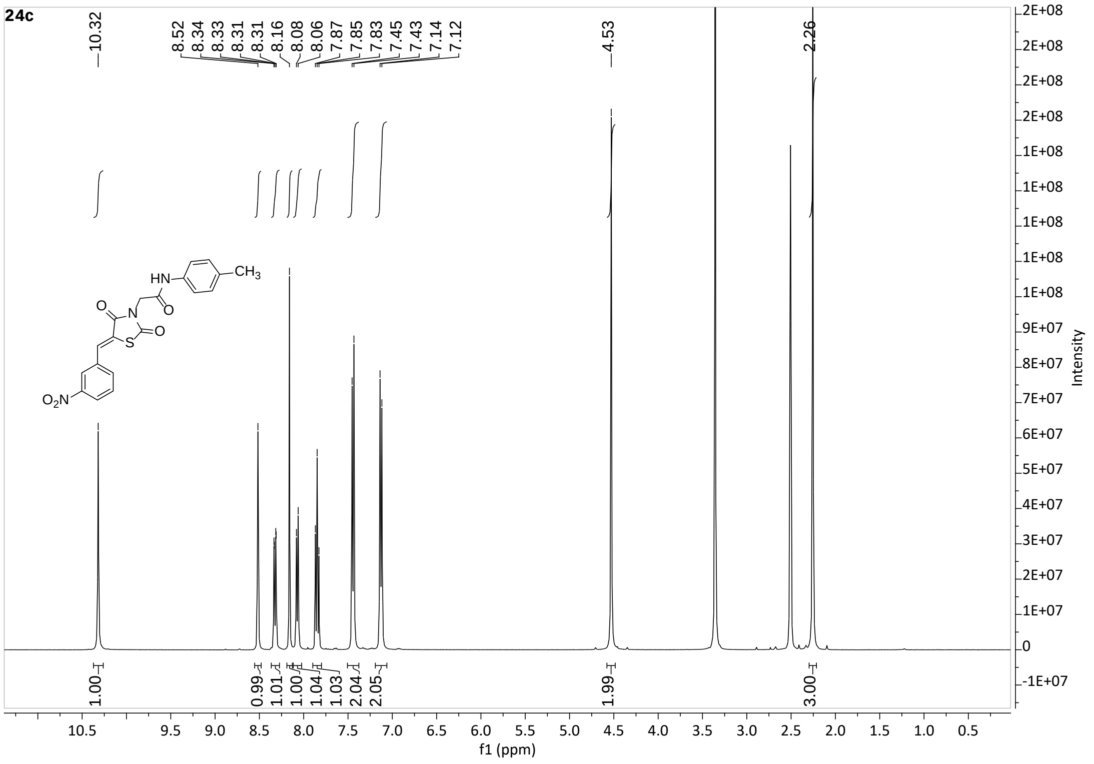


$^{1}$H-NMR spectrum of (*Z*)-*N*-(4-methylphenyl)2-(5-(3-nitrobenzylidene)thiazolidine-2,4-dion-3-yl)acetamide (**24c**)

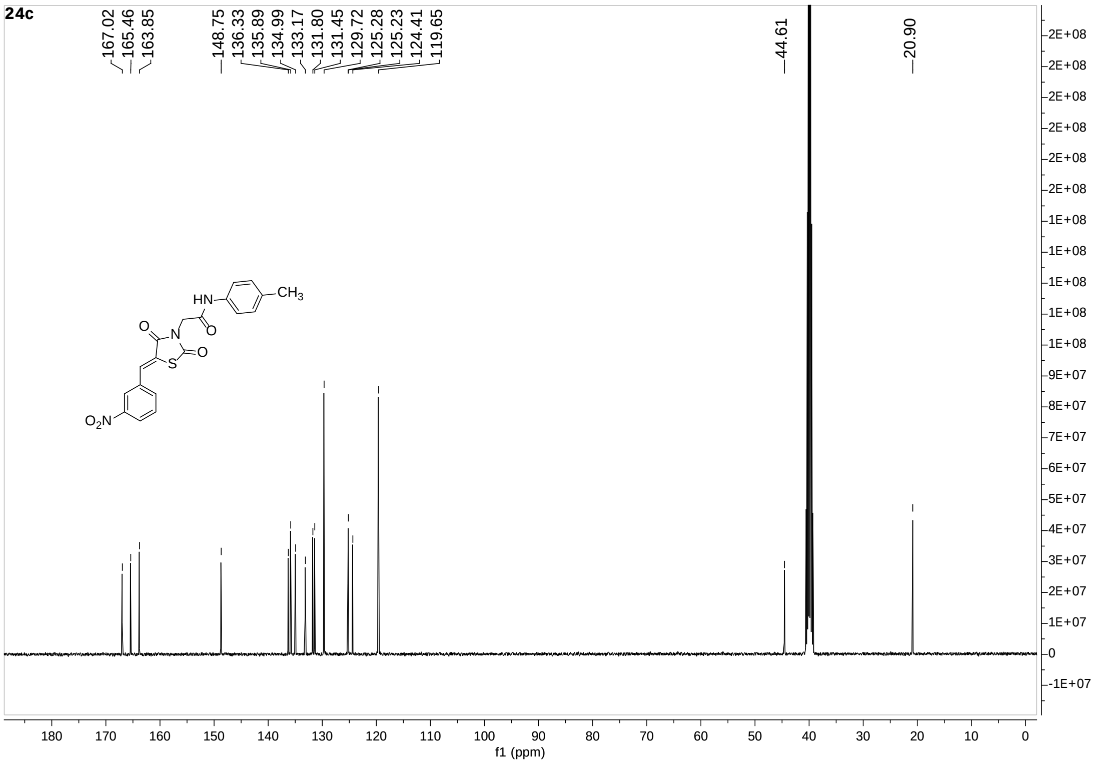


$^{13}$C-NMR spectrum of (*Z*)-*N*-(4-methylphenyl)2-(5-(3-nitrobenzylidene)thiazolidine-2,4-dion-3-yl)acetamide (**24c**)

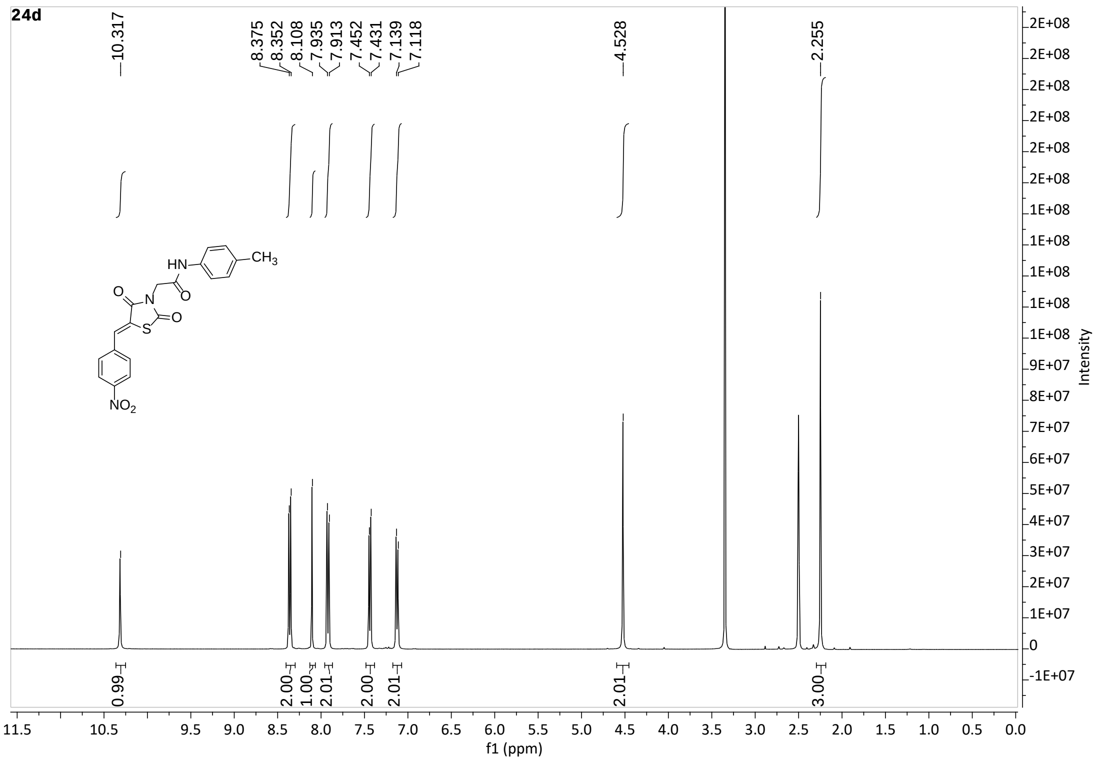


$^{1}$H-NMR spectrum of (*Z*)-*N*-(4-methylphenyl)2-(5-(3-nitrobenzylidene)thiazolidine-2,4-dion-3-yl)acetamide (**24d**)

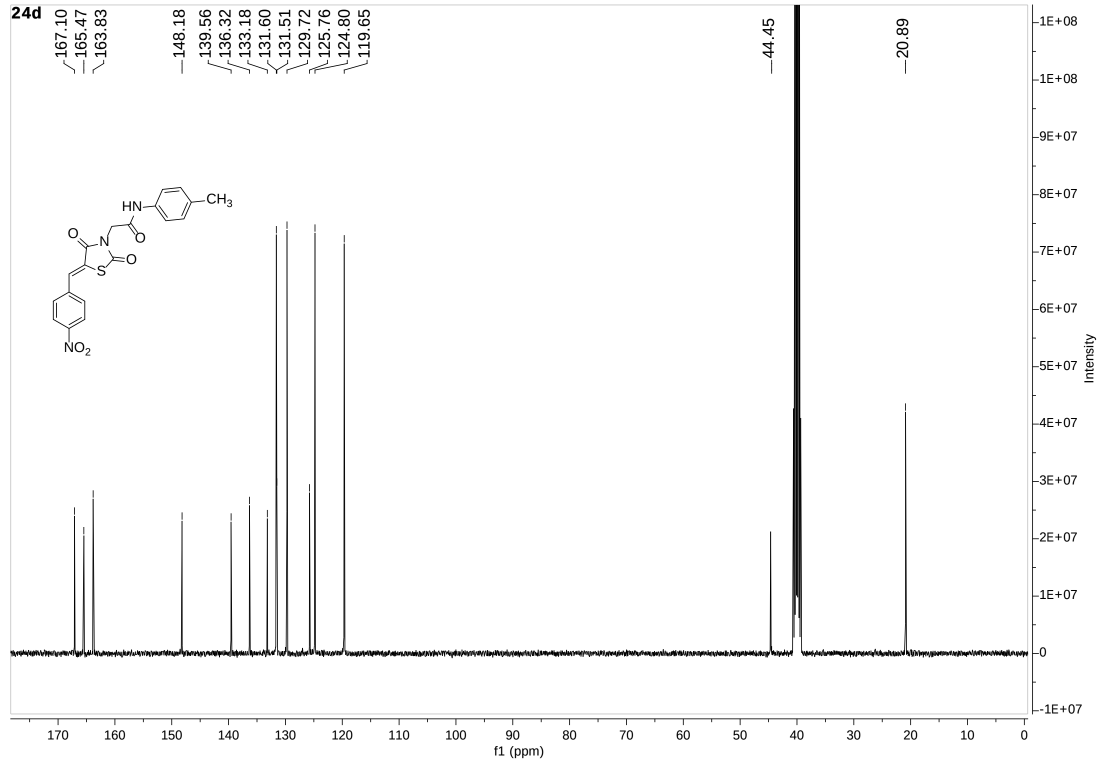


$^{13}$C-NMR spectrum of (*Z*)-*N*-(4-methylphenyl)2-(5-(3-nitrobenzylidene)thiazolidine-2,4-dion-3-yl)acetamide (**24d**)

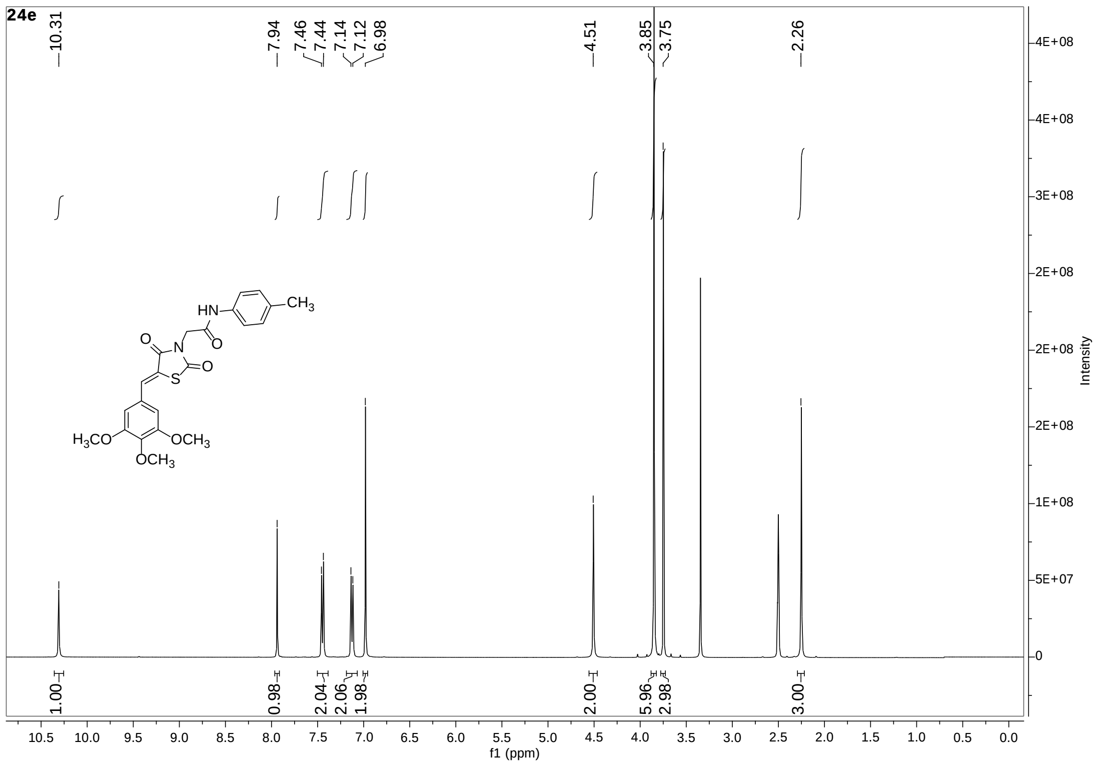


$^{1}$H-NMR spectrum of (*Z*)-*N*-(4-methylphenyl)-2-(5-(3,4,5-trimethoxybenzylidene) thiazolidine-2,4-dion-3-yl)acetamide (**24e**)

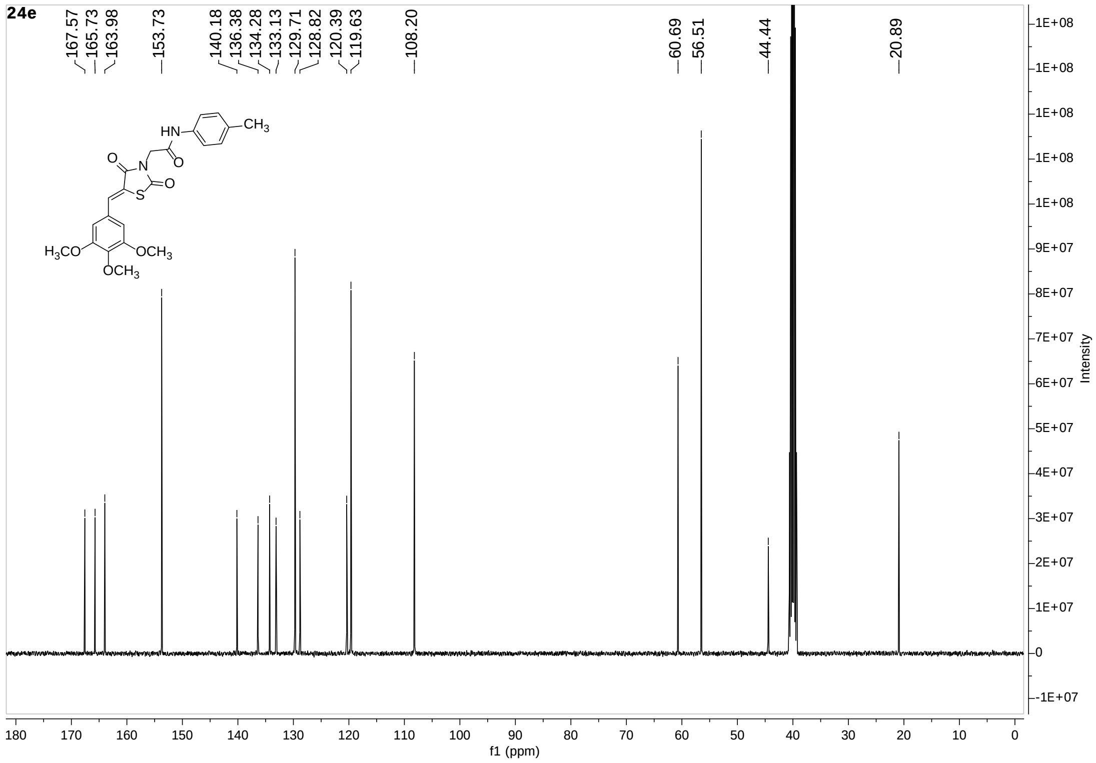


$^{13}$C-NMR spectrum of (*Z*)-*N*-(4-methylphenyl)-2-(5-(3,4,5-trimethoxybenzylidene) thiazolidine-2,4-dion-3-yl)acetamide (**24e**)

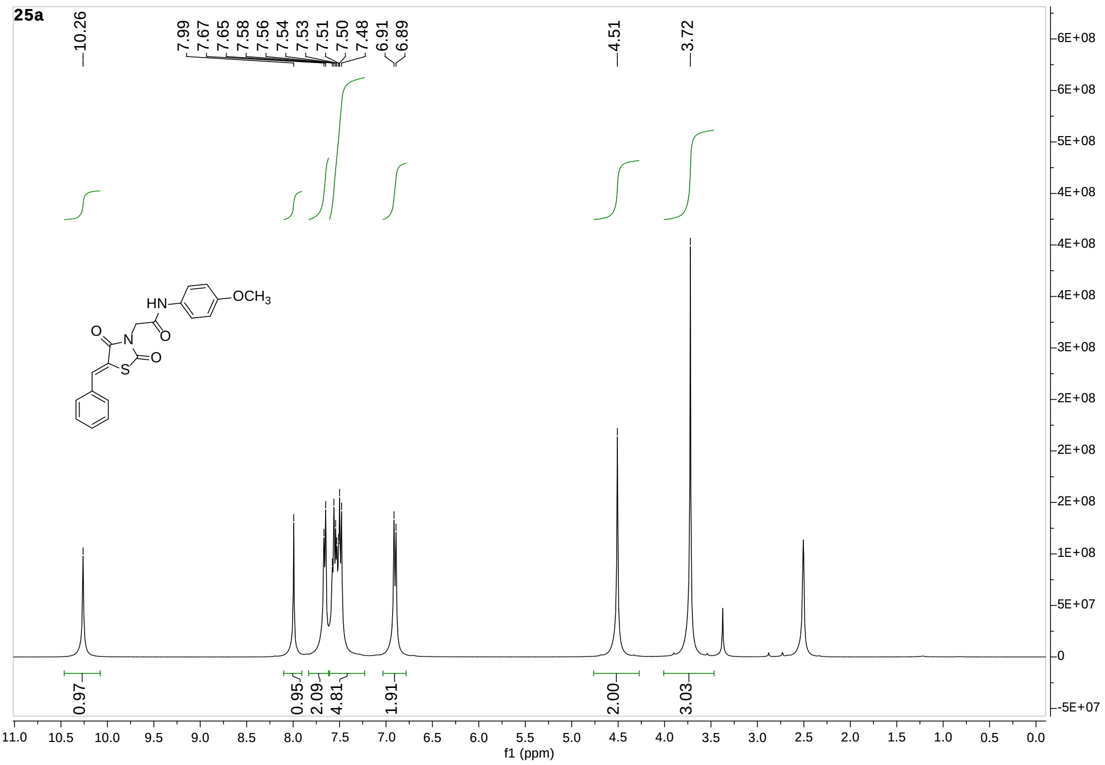


$^{1}$H-NMR spectrum of (*Z*)-2-(5-benzylidenethiazolidine-2,4-dion-3-yl)-N-(4-methoxyphenyl)acetamide (25a)

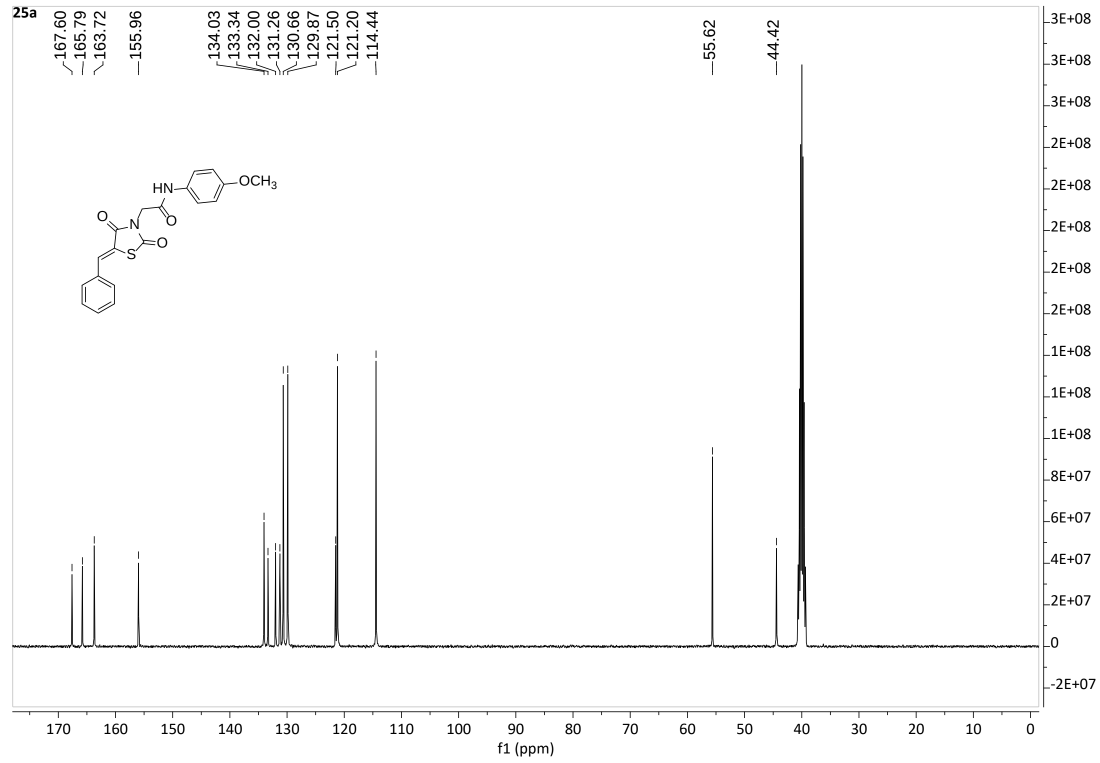


$^{13}$C-NMR spectrum of (*Z*)-2-(5-benzylidenethiazolidine-2,4-dion-3-yl)-N-(4-methoxyphenyl)acetamide (25a)

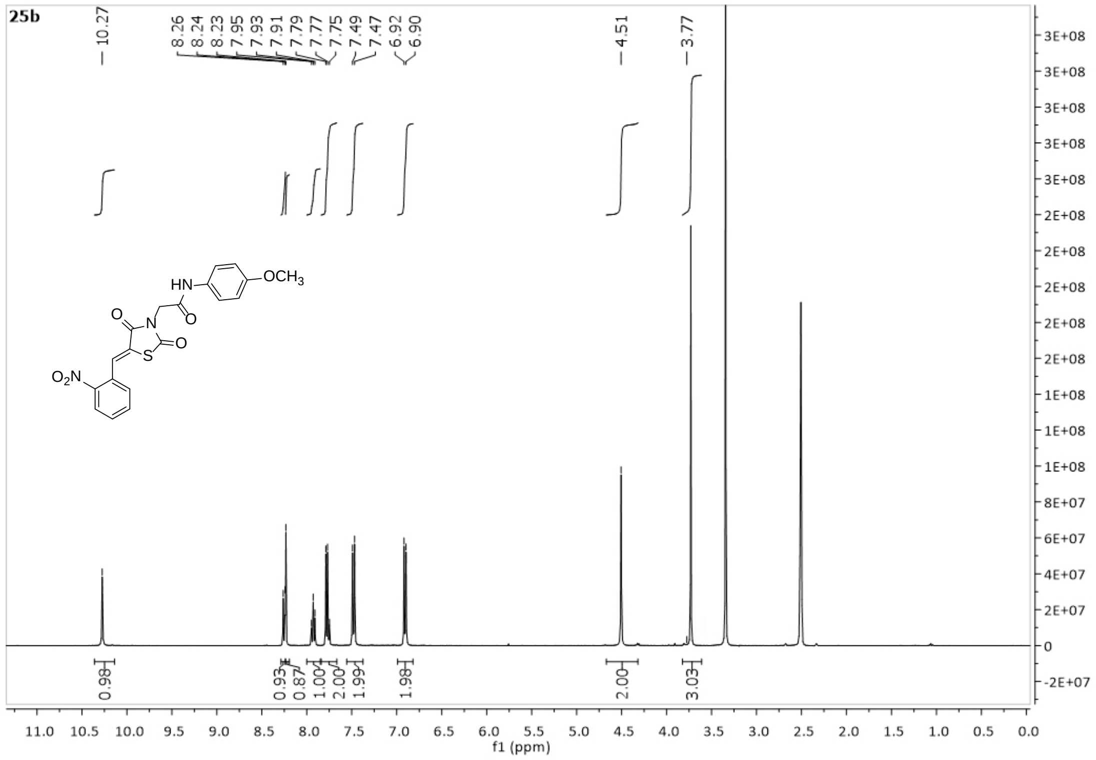


$^{1}$H-NMR spectrum of (*Z*)-2-(5-(2-nitrobenzylidene)thiazolidine-2,4-dion-3-yl)-N-(4-methoxyphenyl)acetamide (**25b**)

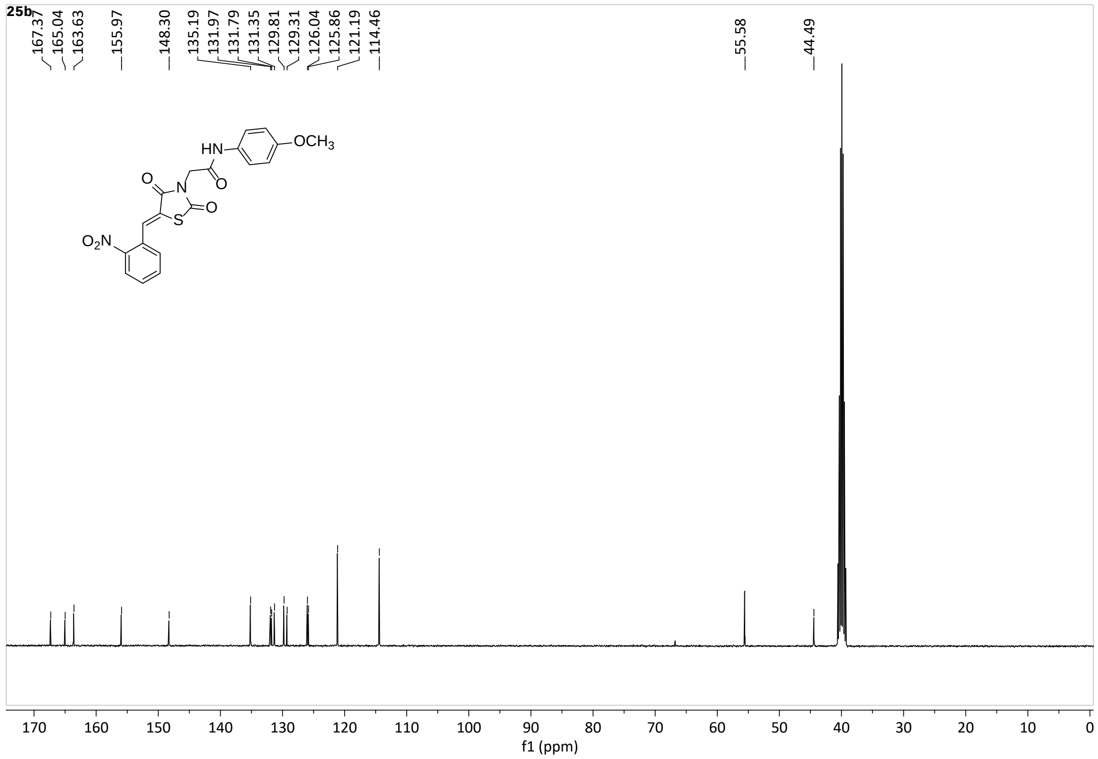


$^{13}$C-NMR spectrum of (*Z*)-2-(5-(2-nitrobenzylidene)thiazolidine-2,4-dion-3-yl)-N-(4-methoxyphenyl)acetamide (**25b**)

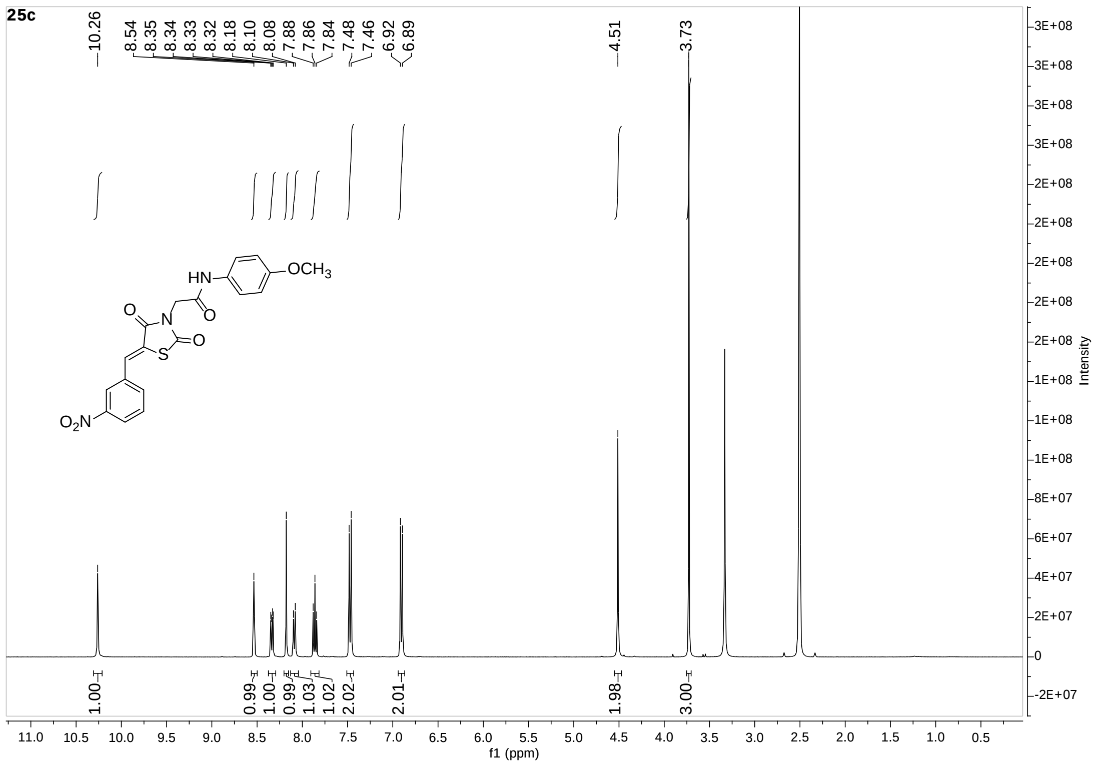


$^{1}$H-NMR spectrum of (*Z*)-2-(5-(3-nitrobenzylidene)thiazolidine-2,4-dion-3-yl)-N-(4-methoxyphenyl)acetamide (**25c**)

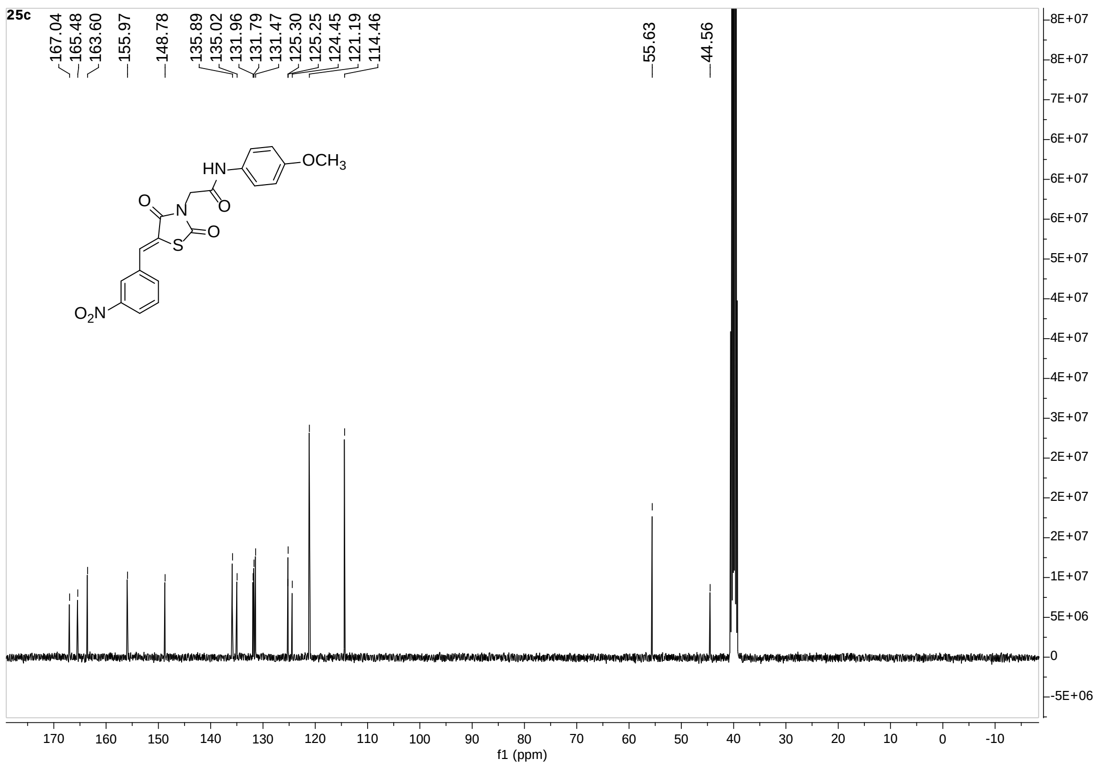


$^{13}$C-NMR spectrum of (*Z*)-2-(5-(3-nitrobenzylidene)thiazolidine-2,4-dion-3-yl)-N-(4-methoxyphenyl)acetamide (**25c**)

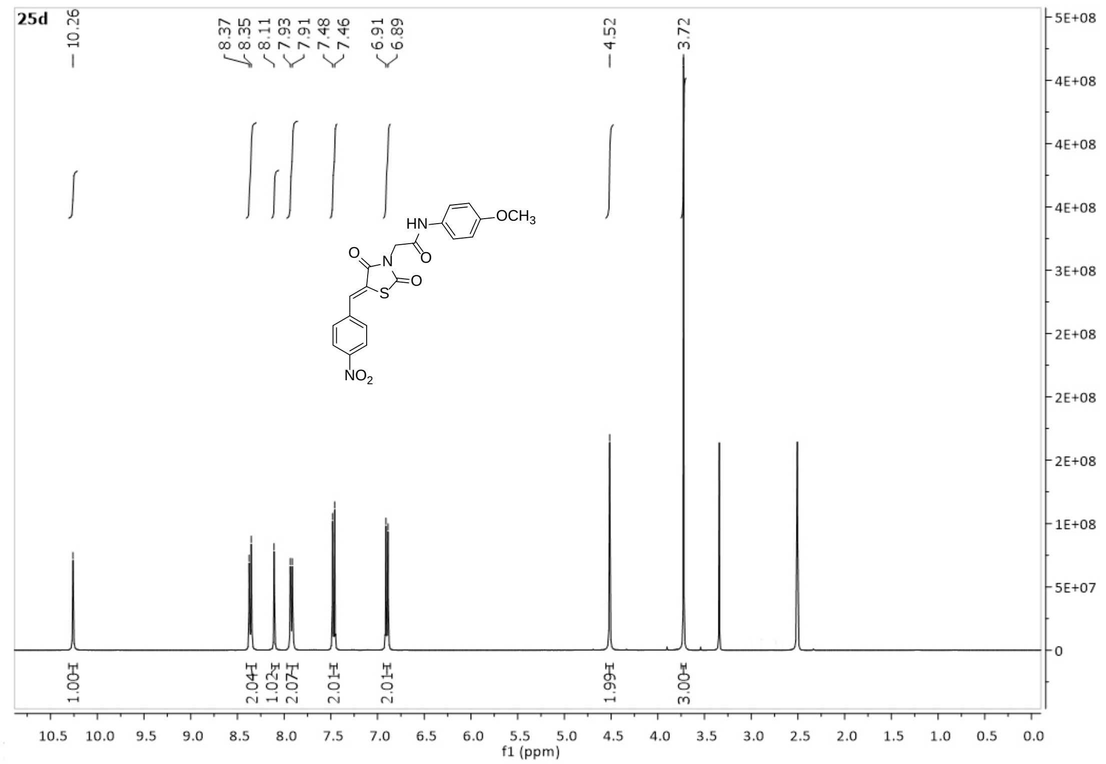


$^{1}$H-NMR spectrum of (*Z*)-*N*-(4-methoxyphenyl)-2-(5-(4-nitrobenzylidene)thiazolidine-2,4-dion-3-yl)acetamide (**25d**)

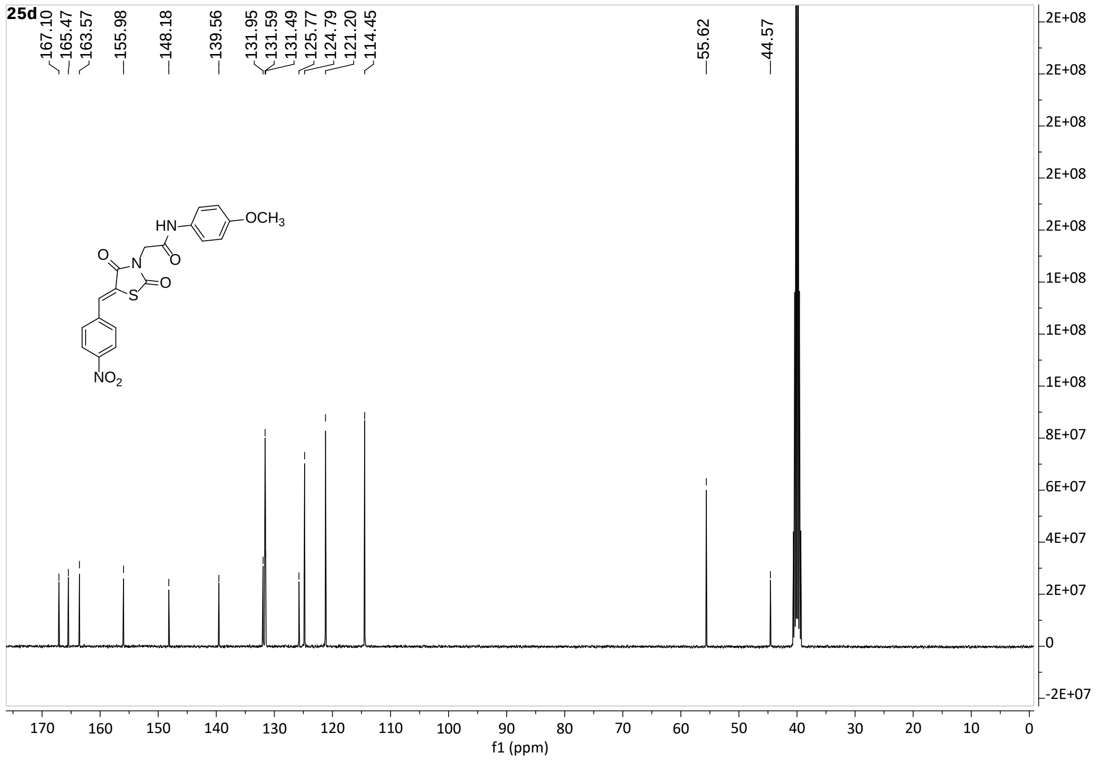


$^{13}$C-NMR spectrum of (*Z*)-*N*-(4-methoxyphenyl)-2-(5-(4-nitrobenzylidene) thiazolidine -2,4-dion-3-yl)acetamide (**25d**)

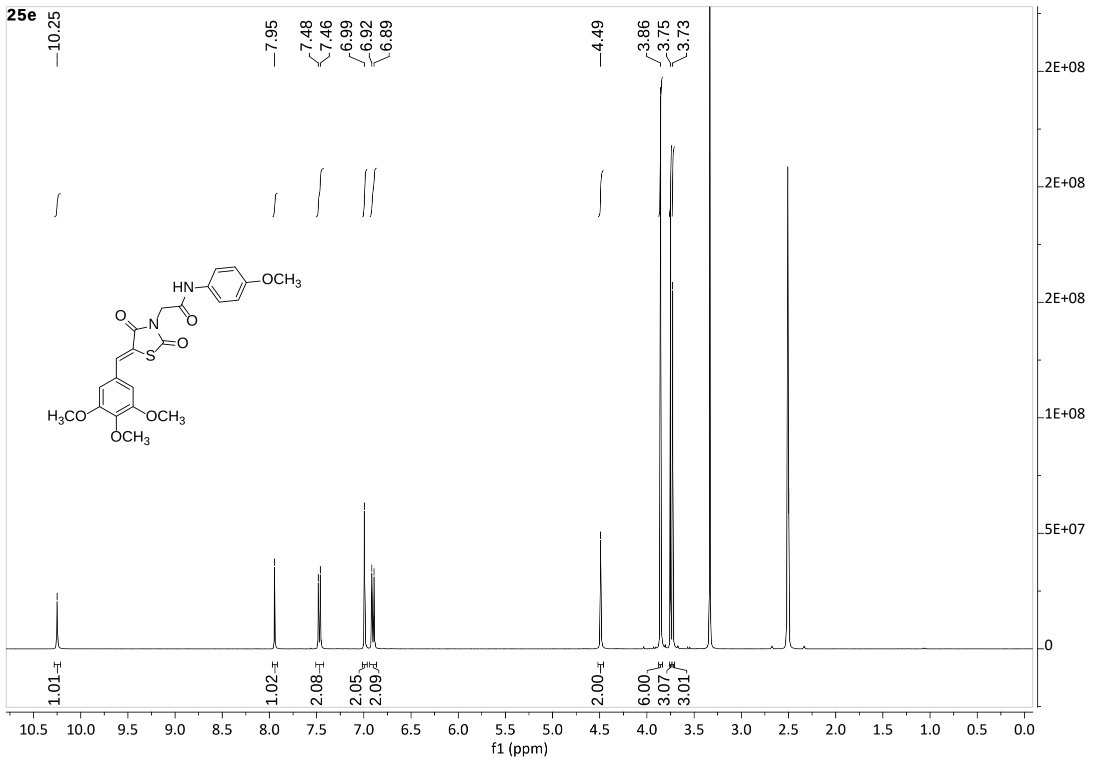


$^{1}$H-NMR spectrum of (*Z*)-*N*-(4-methoxyphenyl)-2-(5-(3,4,5-trimethoxybenzylidene) thiazolidine-2,4-dion-3-yl)acetamide (**25e**)

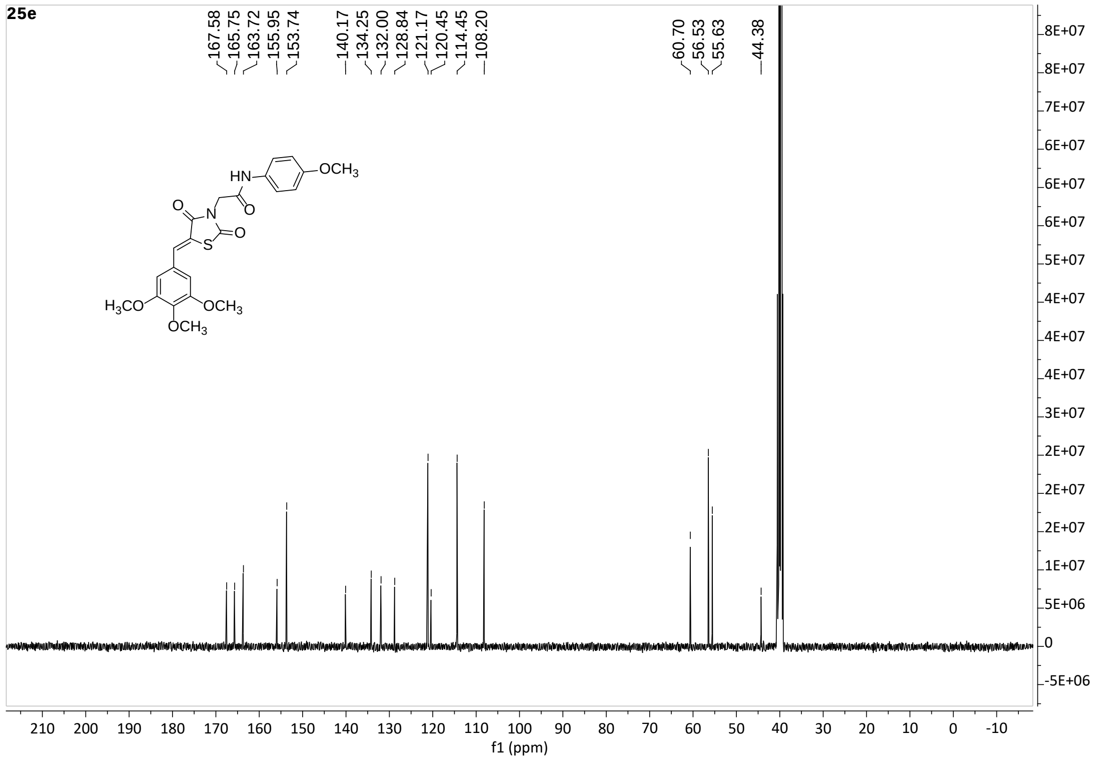


$^{13}$C-NMR spectrum of (*Z*)-*N*-(4-methoxyphenyl)-2-(5-(3,4,5-trimethoxybenzylidene) thiazolidine-2,4-dion-3-yl)acetamide (**25e**)

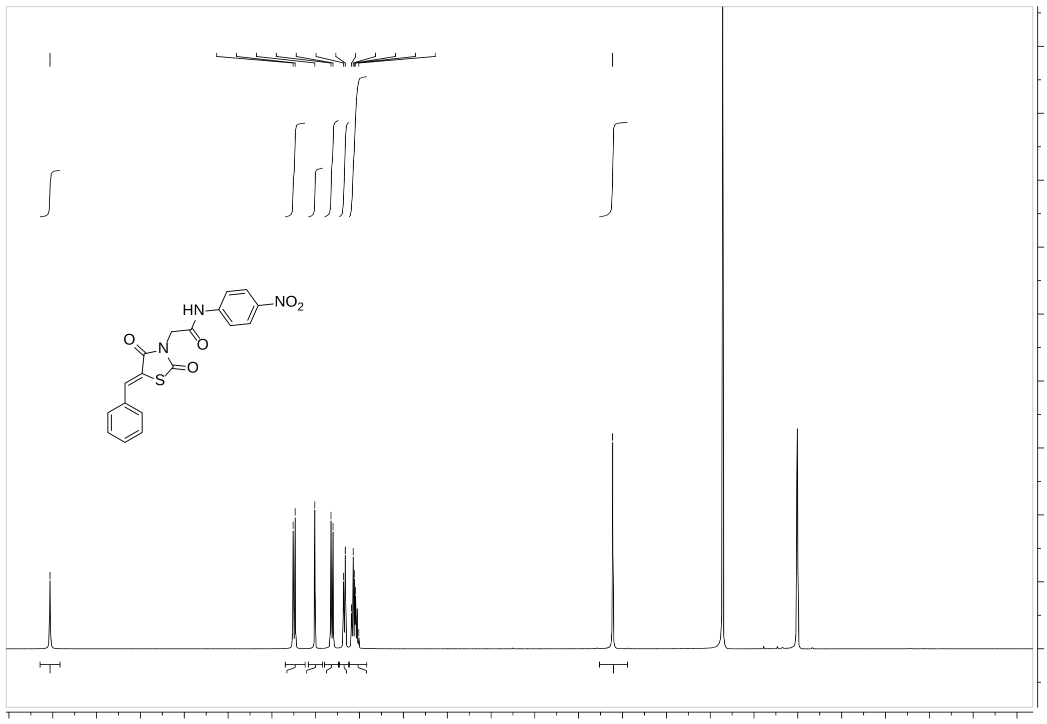


$^{1}$H-NMR spectrum of (*Z*)-2-(5-benzylidenethiazolidine-2,4-dion-3-yl)-*N*-(4-nitrophenyl)acetamide (**26a**)

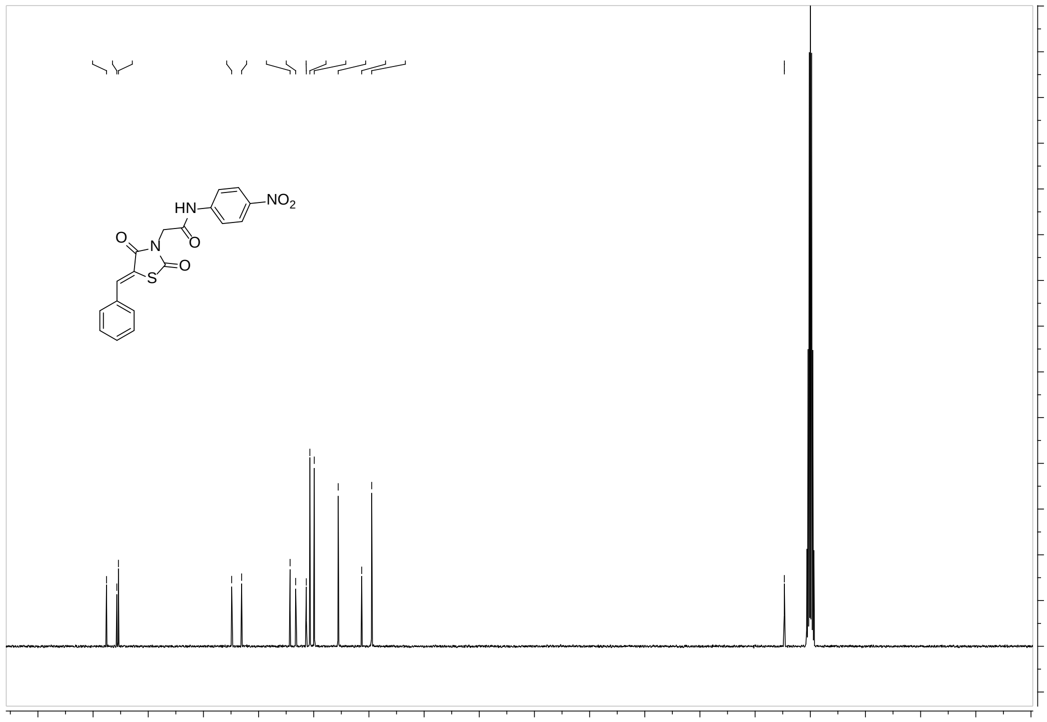


$^{13}$C-NMR spectrum of (*Z*)-2-(5-benzylidenethiazolidine-2,4-dion-3-yl)-*N*-(4-nitrophenyl)acetamide (**26a**)

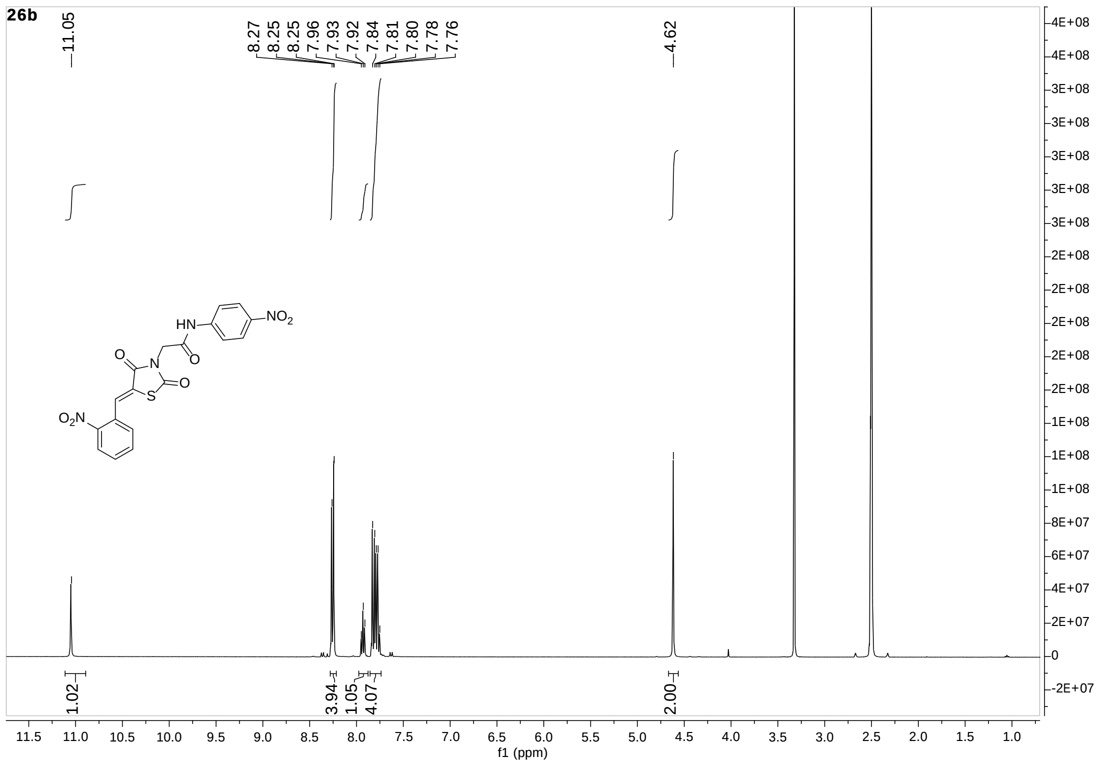

$^{1}$H-NMR spectrum of (*Z*)-2-(5-(2-nitrobenzylidene)thiazolidine-2,4-dion-3-yl)-*N*-(4-nitroyphenyl)acetamide (**26b**)

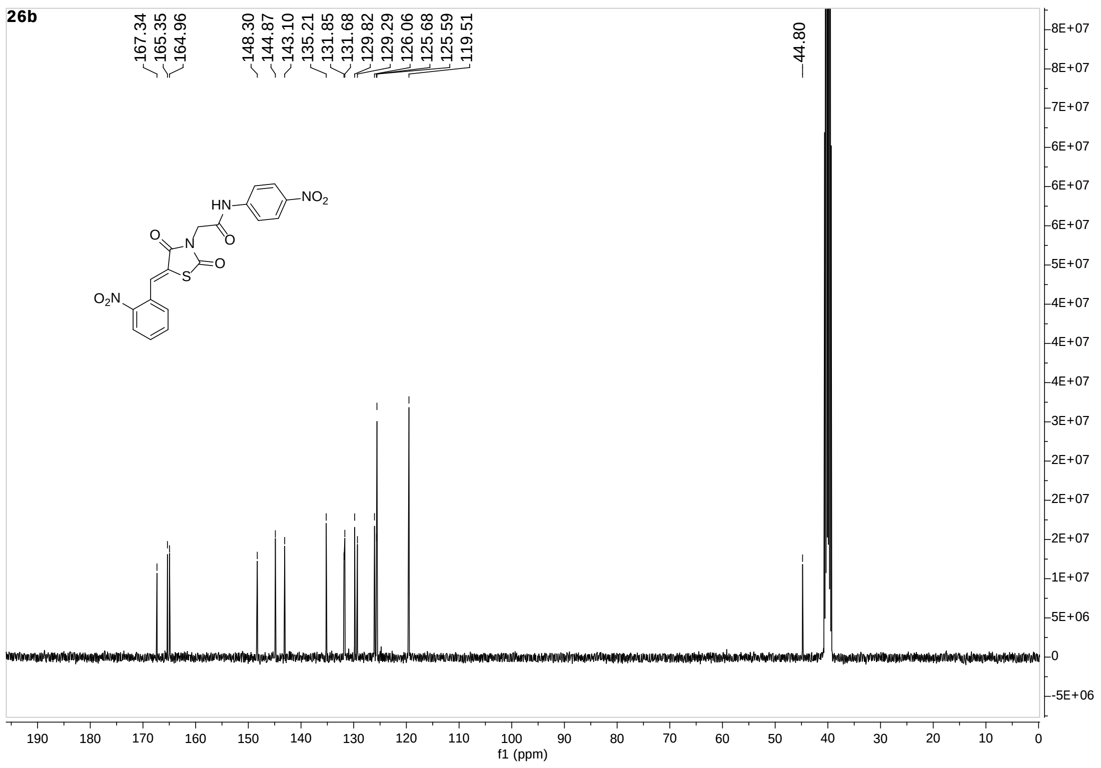

$^{13}$C-NMR spectrum of (*Z*)-2-(5-(2-nitrobenzylidene)thiazolidine-2,4-dion-3-yl)-*N*-(4-nitroyphenyl)acetamide (**26b**)

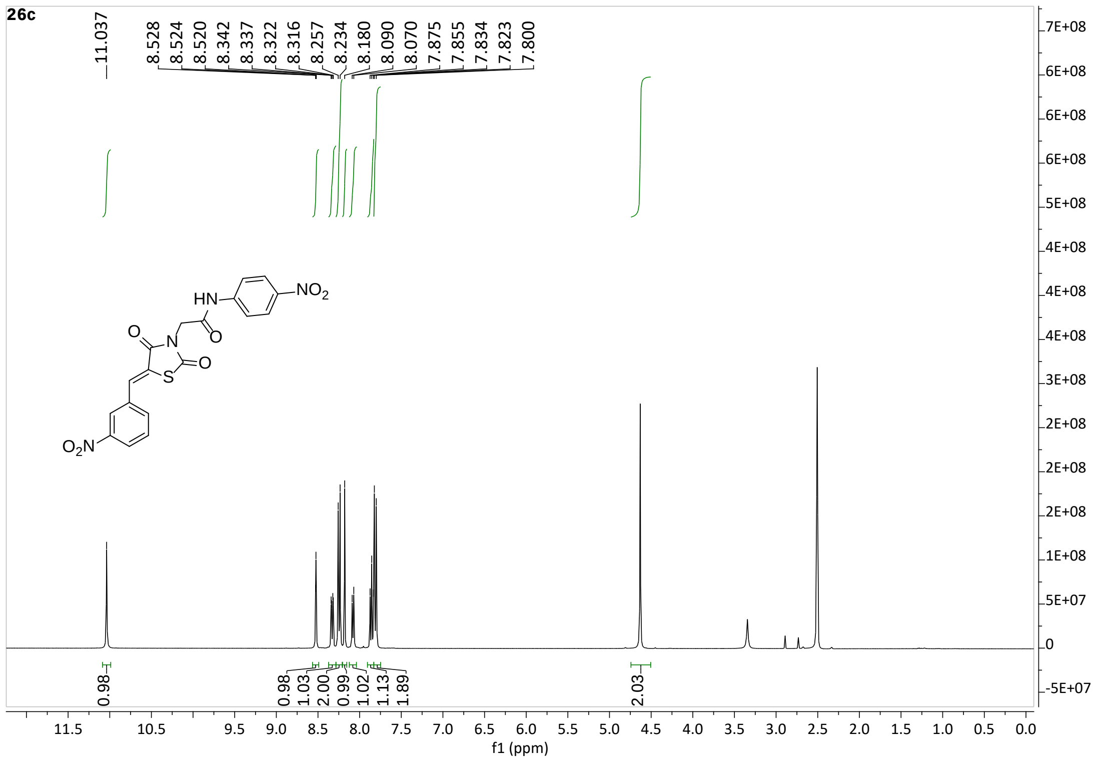

$^{1}$H-NMR spectrum of (*Z*)-2-(5-(3-nitrobenzylidene)thiazolidine-2,4-dion-3-yl)-*N*-(4-nitroyphenyl)acetamide (**26c**)

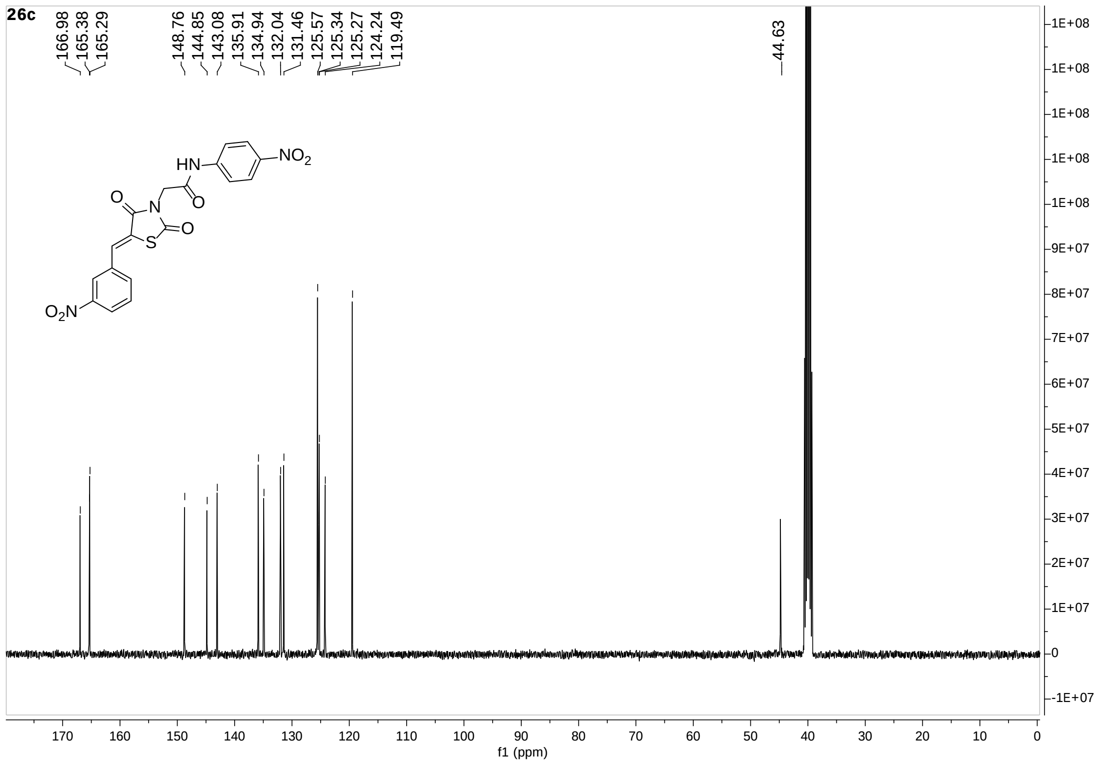

$^{13}$C-NMR spectrum of (*Z*)-2-(5-(3-nitrobenzylidene)thiazolidine-2,4-dion-3-yl)-*N*-(4-nitroyphenyl)acetamide (**26c**)

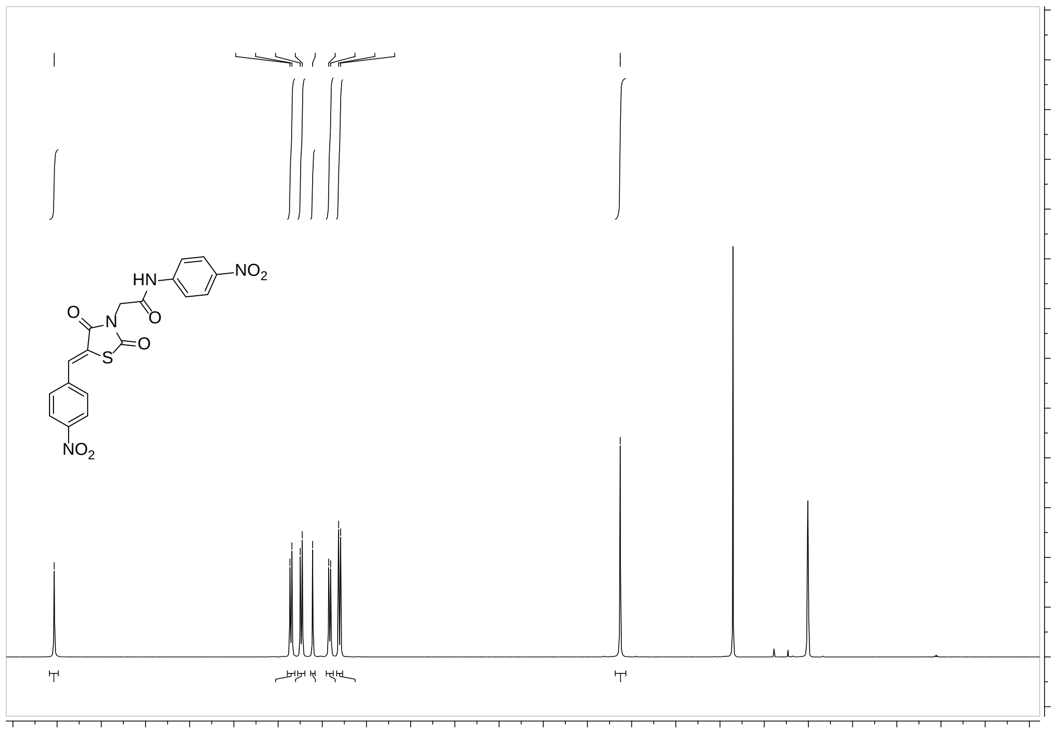


$^{1}$H-NMR spectrum of (*Z*)-2-(5-(4-nitrobenzylidene)thiazolidine-2,4-dion-3-yl)-*N*-(4-nitroyphenyl)acetamide (**26d**)

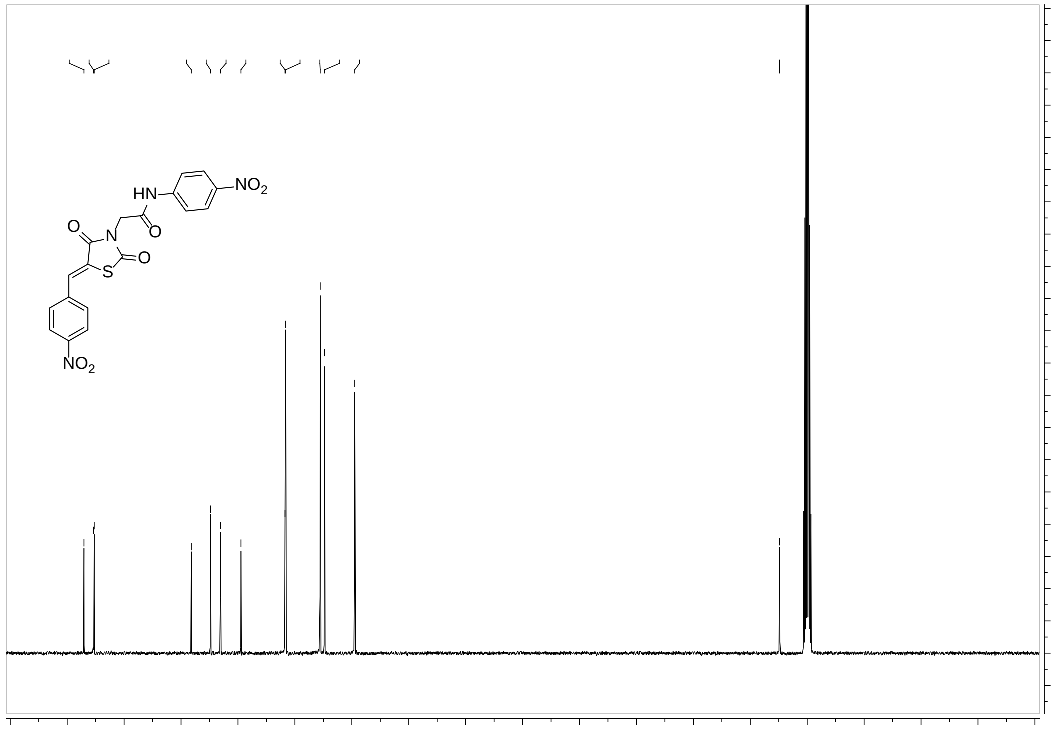


$^{13}$C-NMR spectrum of $^{1}$H-NMR spectrum of (*Z*)-2-(5-(4-nitrobenzylidene)thiazolidine -2,4-dion-3-yl)-*N*-(4-nitroyphenyl)acetamide (**26d**)

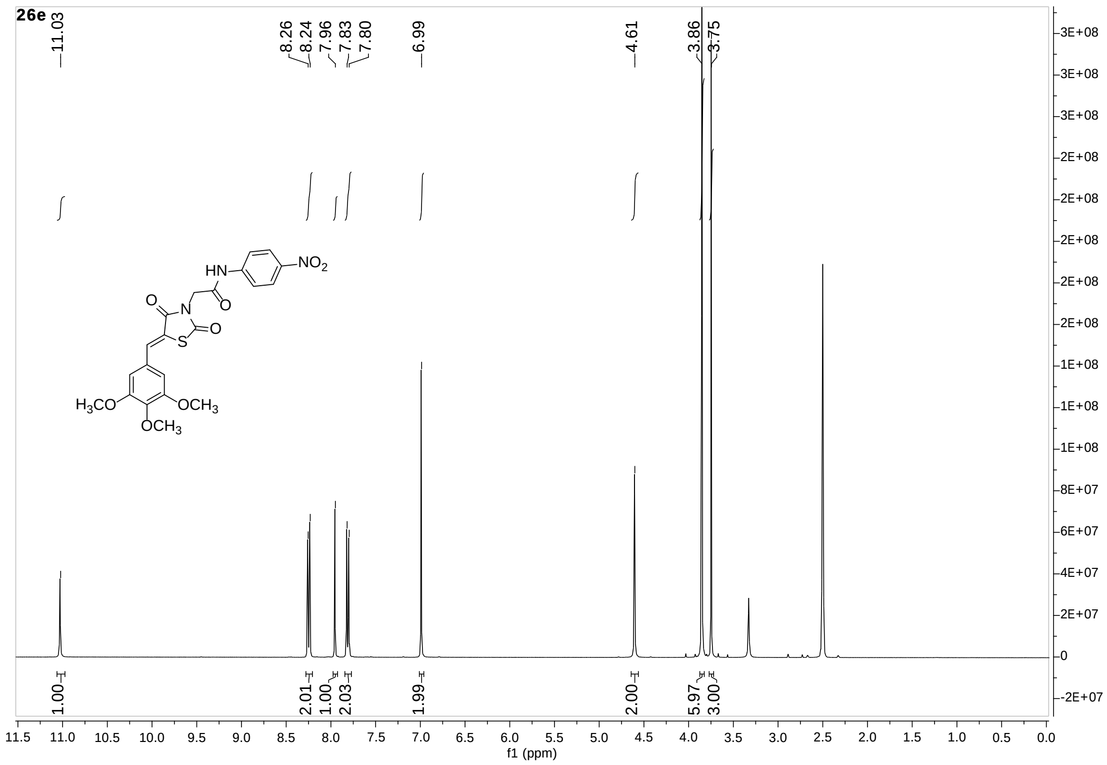


$^{1}$H-NMR spectrum of (*Z*)-*N*-(4-nitrophenyl)-2-(5-(3,4,5-trimethoxybenzylidene) thiazolidine-2,4-dion-3-yl)acetamide (**26e**)

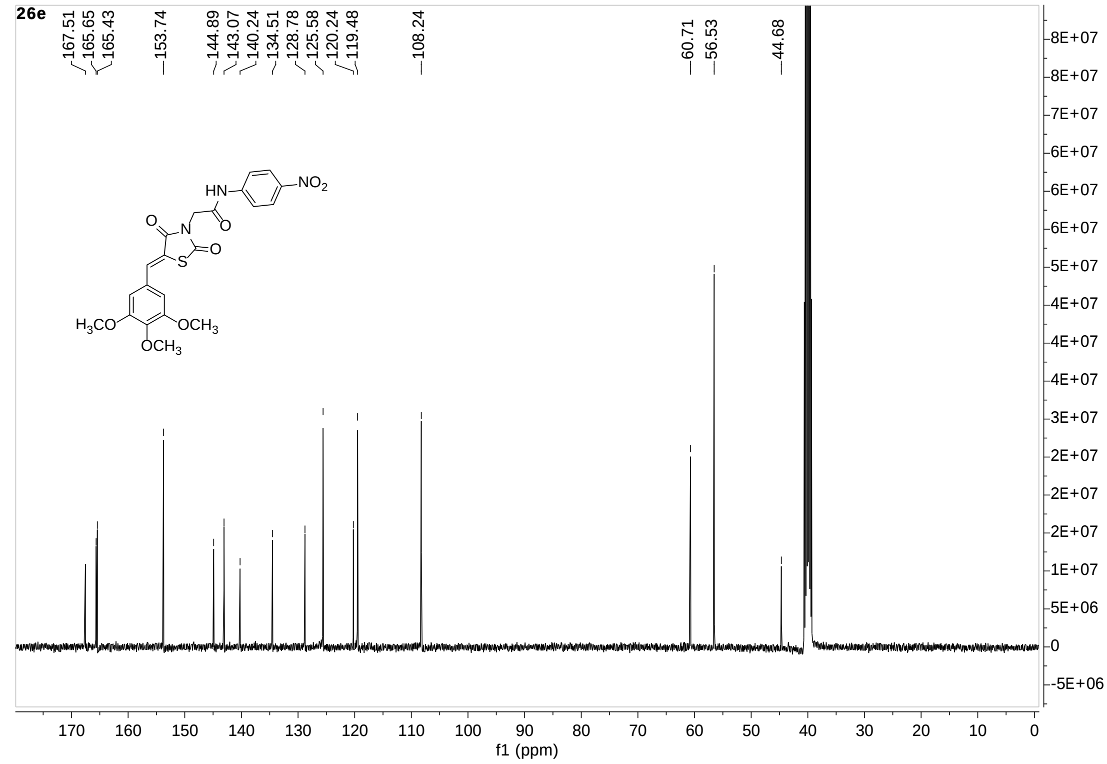


$^{13}$C-NMR spectrum of (*Z*)-*N*-(4-nitrophenyl)-2-(5-(3,4,5-trimethoxybenzylidene) thiazolidine-2,4-dion-3-yl)acetamide (**26e**)

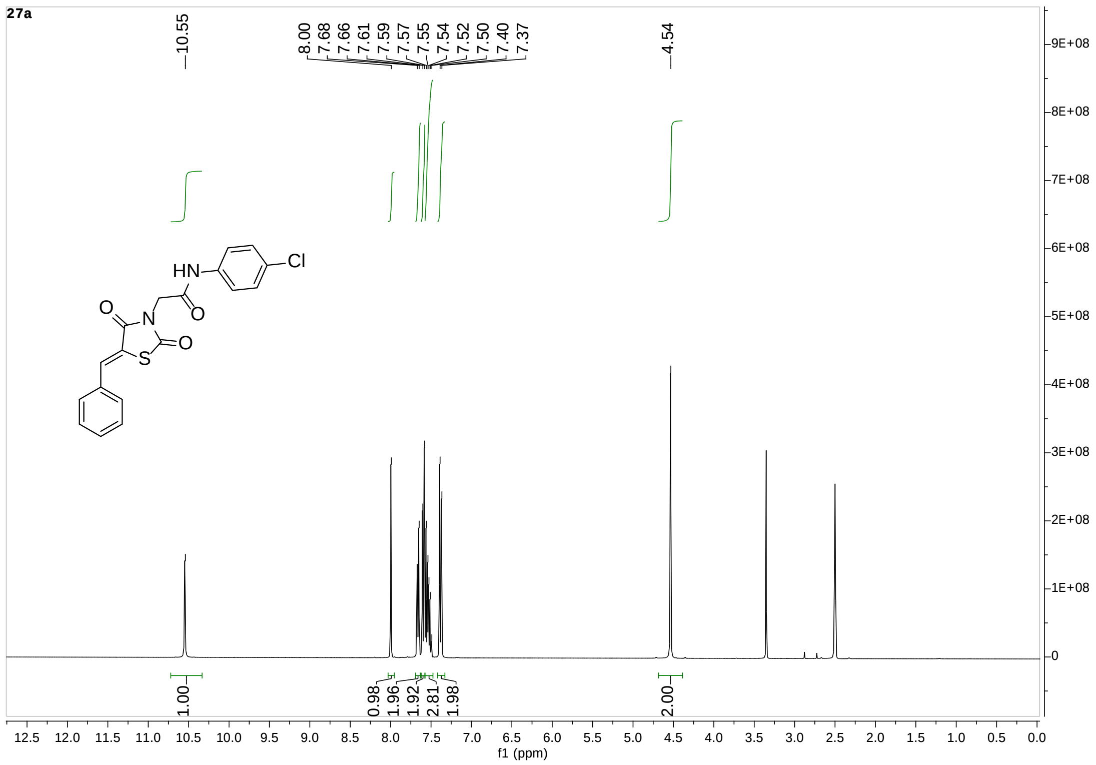


$^{1}$H-NMR spectrum of (*Z*)-2-(5-benzylidenethiazolidine-2,4-dion-3-yl)-*N*-(4-chlorophenyl)acetamide (**27a**)

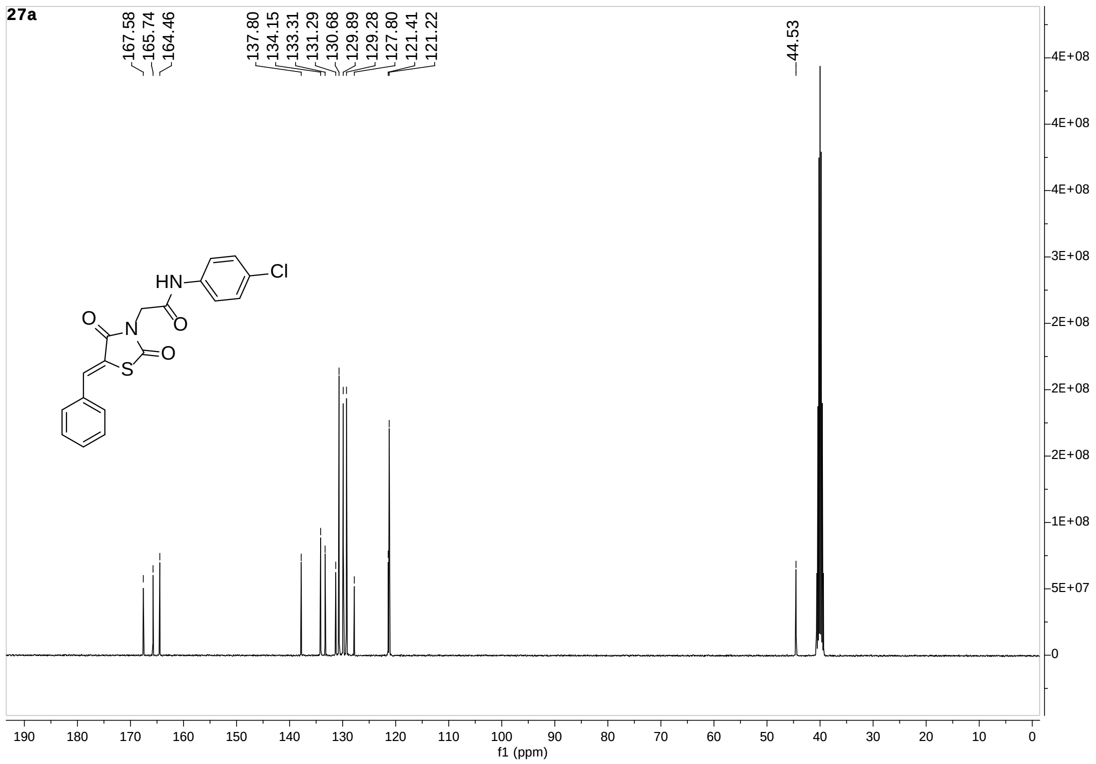


$^{13}$C-NMR spectrum of (*Z*)-2-(5-benzylidenethiazolidine-2,4-dion-3-yl)-*N*-(4-chlorophenyl)acetamide (**27a**)

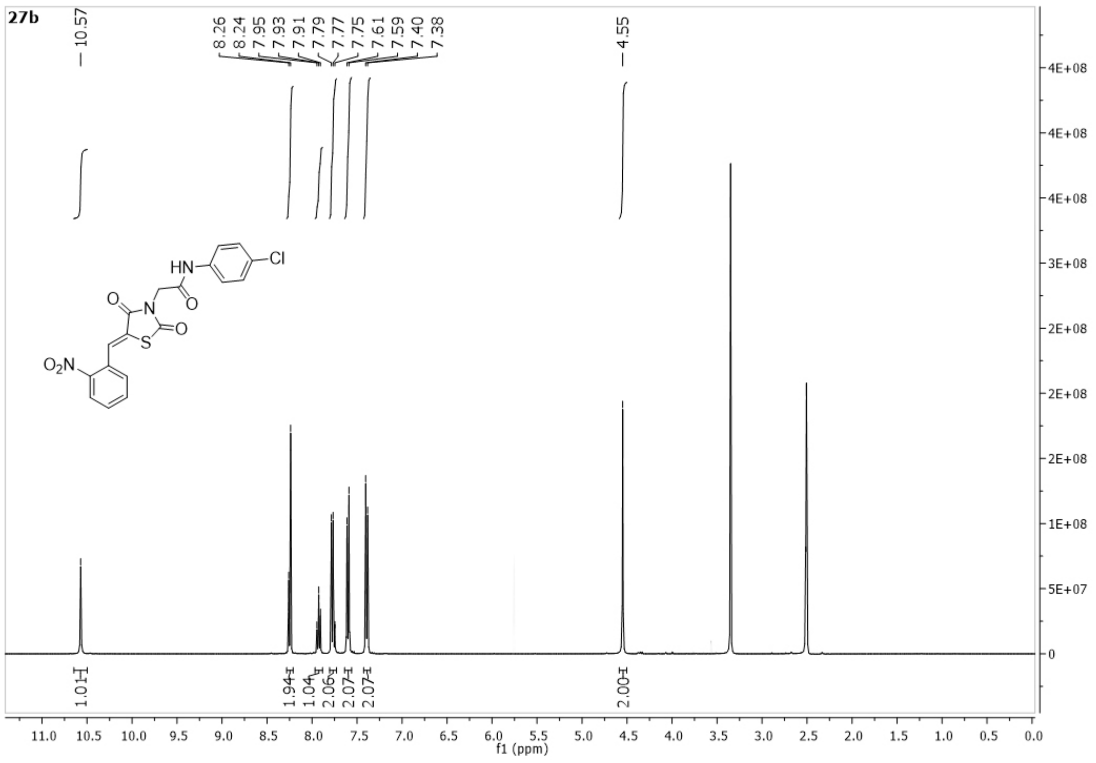


$^{1}$H-NMR spectrum of (*Z*)-*N*-(4-chlorophenyl)-2-(5-(2-nitrobenzylidene)thiazolidine-2,4-dion-3-yl)acetamide (**27b**)

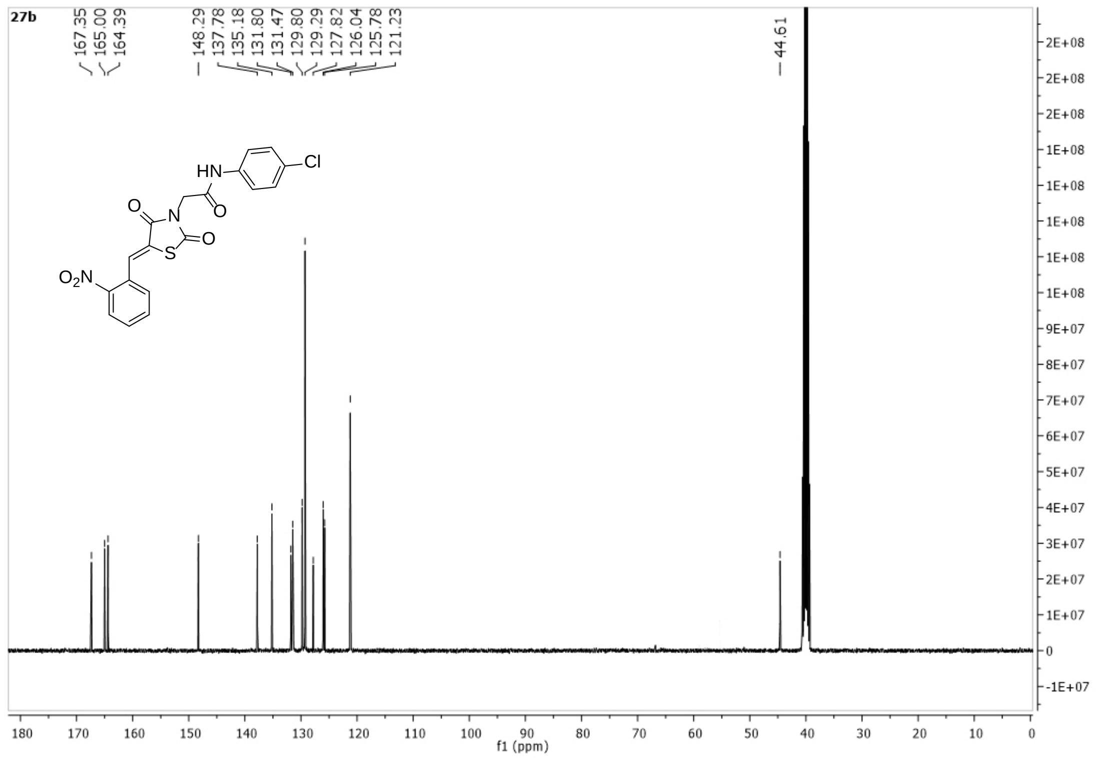


$^{13}$C-NMR spectrum of (*Z*)-*N*-(4-chlorophenyl)-2-(5-(2-nitrobenzylidene)thiazolidine-2,4-dion-3-yl)acetamide (**27b**)

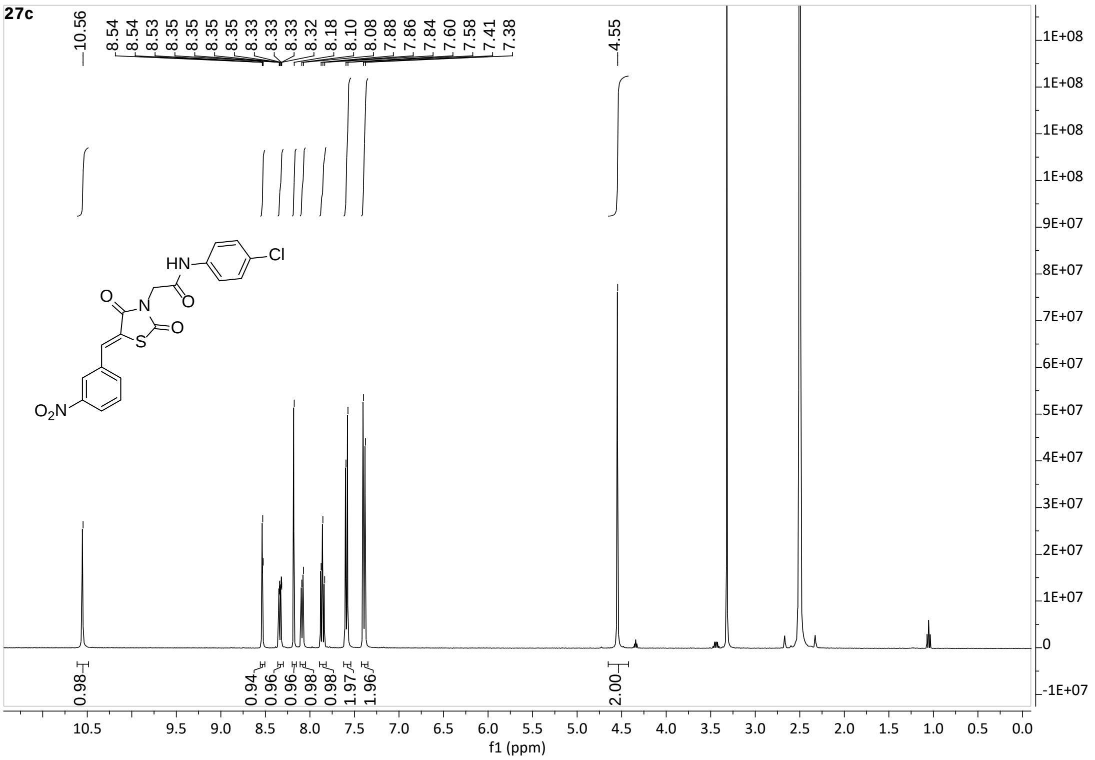


$^{1}$H-NMR spectrum of (*Z*)-*N*-(4-chlorophenyl)-2-(5-(3-nitrobenzylidene)thiazolidine-2,4-dion-3-yl)acetamide (**27c**)

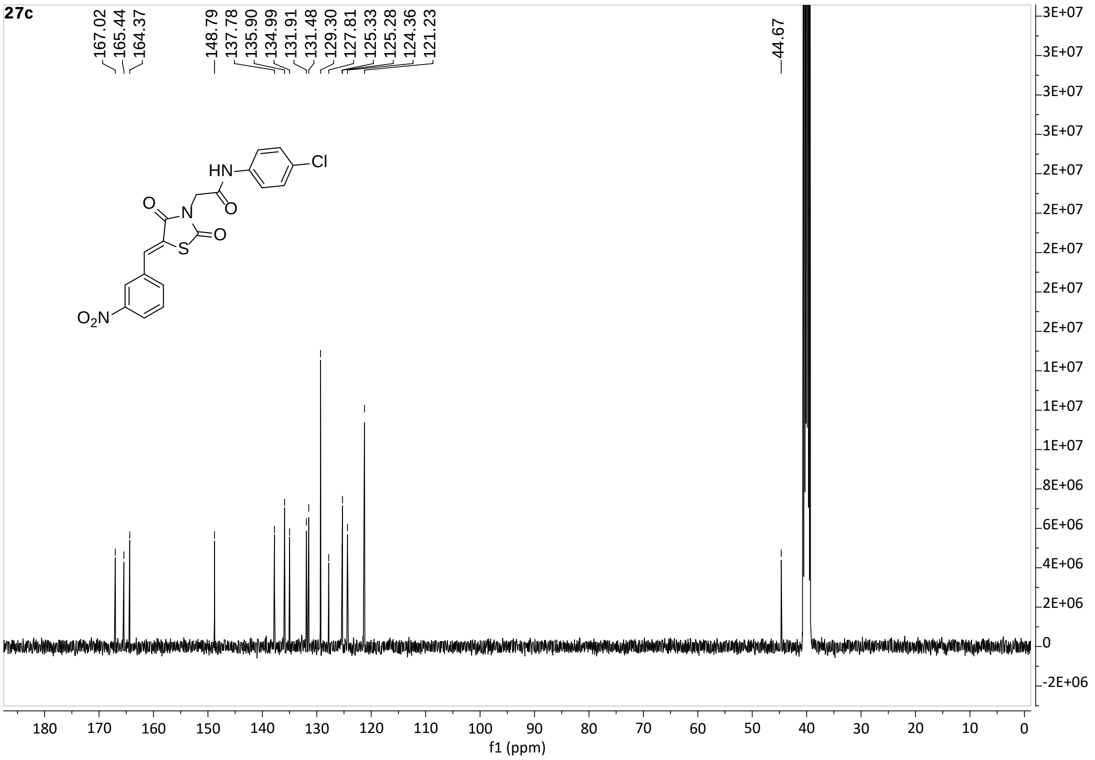


$^{13}$C-NMR spectrum of (*Z*)-*N*-(4-chlorophenyl)-2-(5-(3-nitrobenzylidene)thiazolidine-2,4-dion-3-yl)acetamide (**27c**)

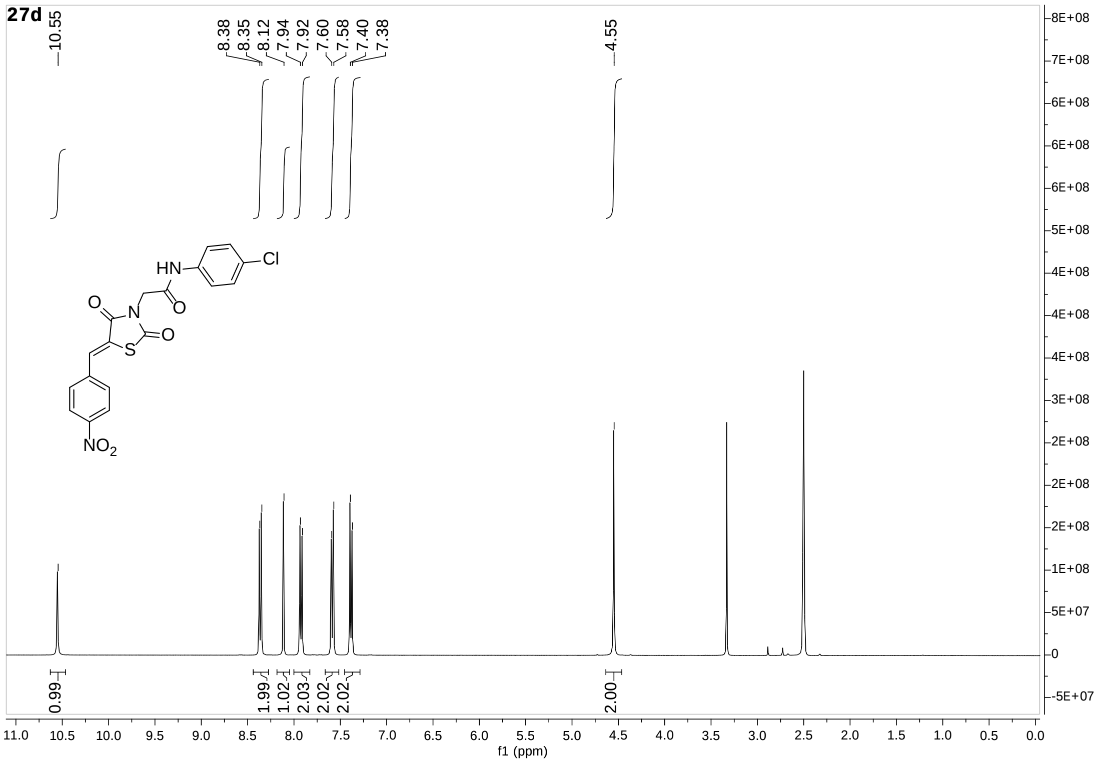


$^{1}$H-NMR spectrum of (*Z*)-*N*-(4-chlorophenyl)-2-(5-(4-nitrobenzylidene)thiazolidine-2,4-dion-3-yl)acetamide (**27d**)

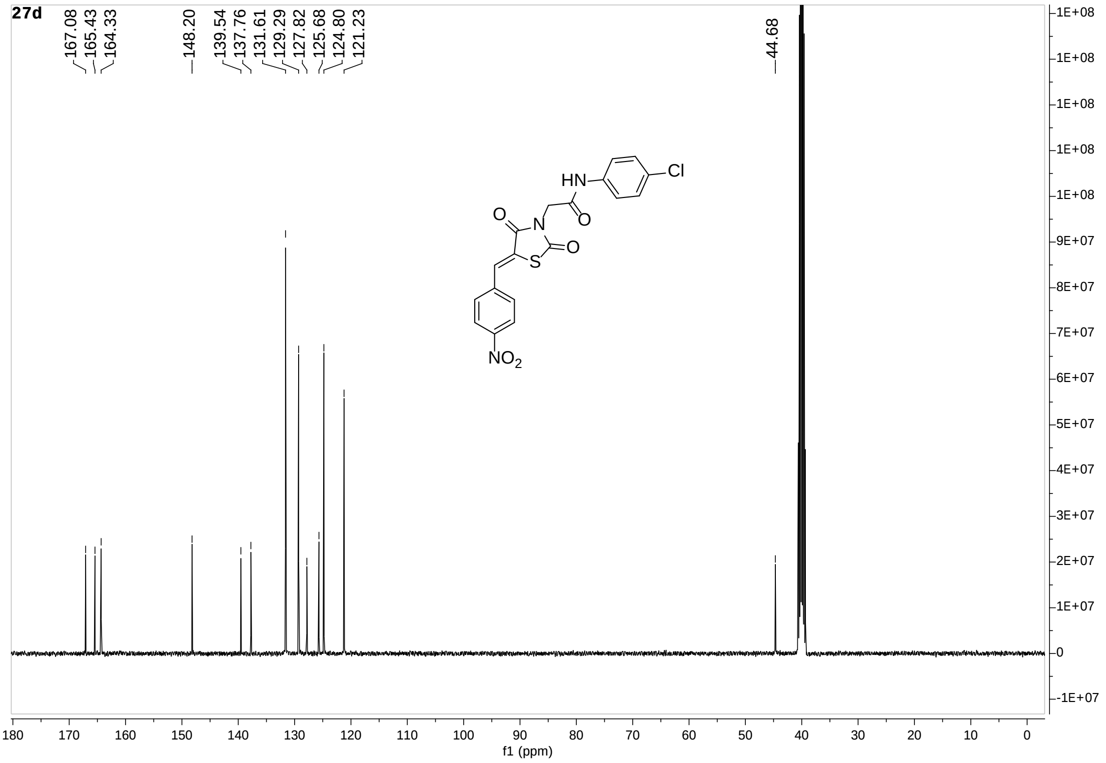


$^{13}$C-NMR spectrum of (*Z*)-*N*-(4-chlorophenyl)-2-(5-(4-nitrobenzylidene)thiazolidine-2,4-dion-3-yl)acetamide (**27d**)

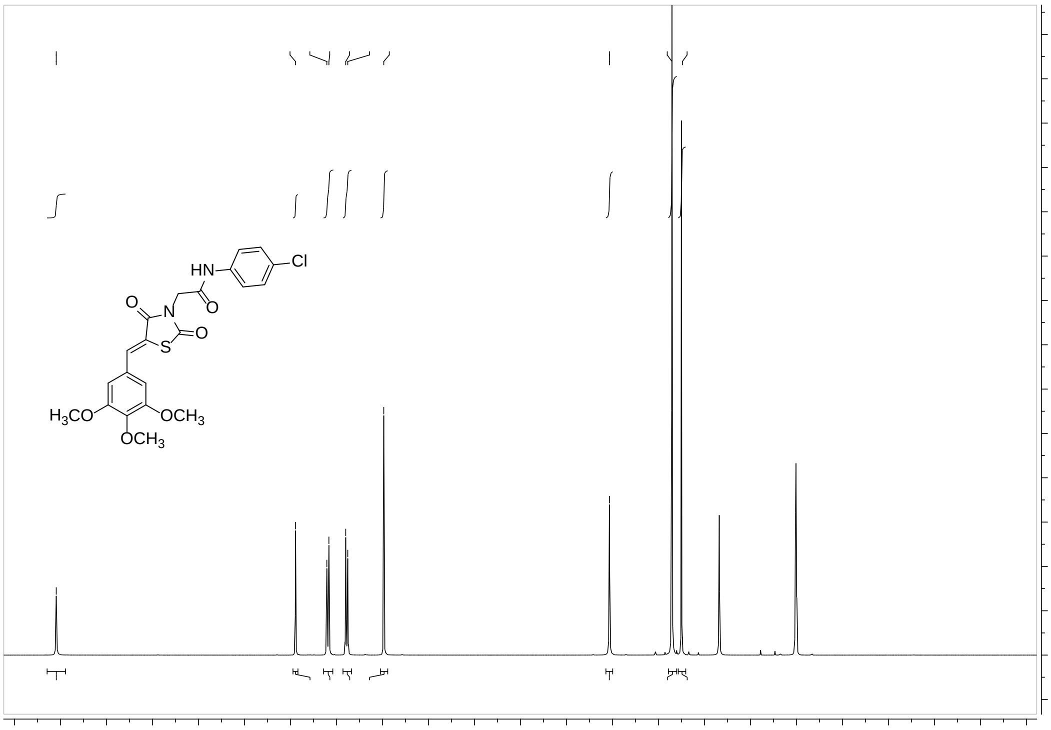


$^{1}$H-NMR spectrum of (*Z*)-*N*-(4-chlorophenyl)-2-(5-(3,4,5-trimethoxybenzylidene) thiazolidine-2,4-dion-3-yl)acetamide (**27e**)

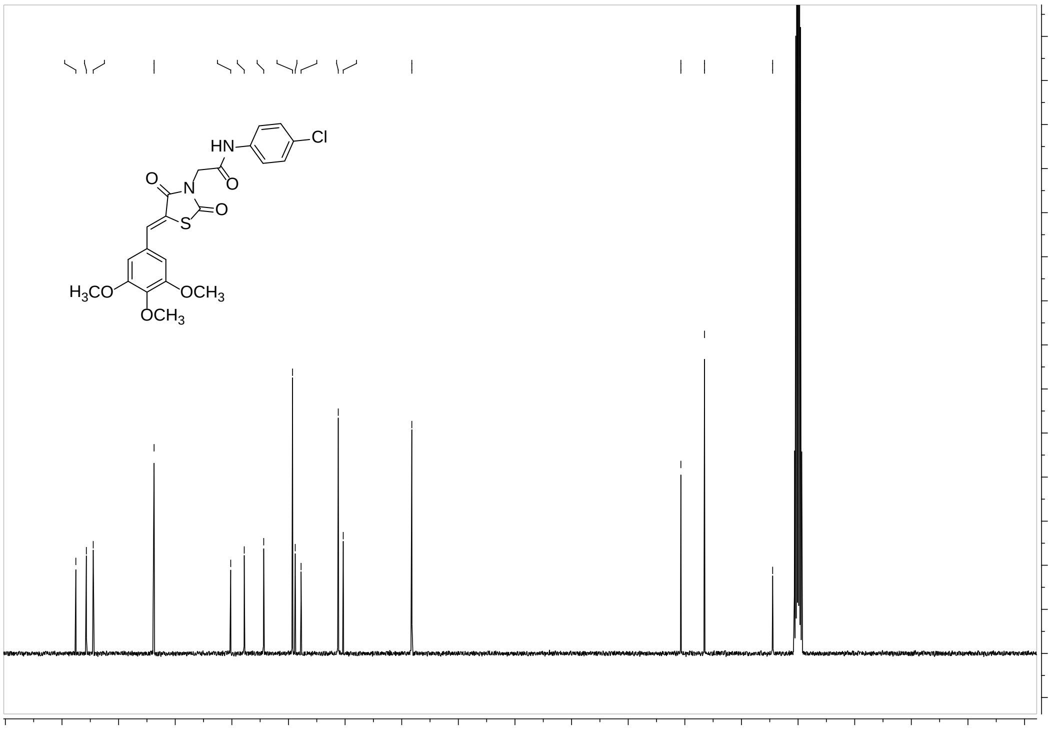


$^{13}$C-NMR spectrum of (*Z*)-*N*-(4-chlorophenyl)-2-(5-(3,4,5-trimethoxybenzylidene) thiazolidine-2,4-dion-3-yl)acetamide (**27e**)

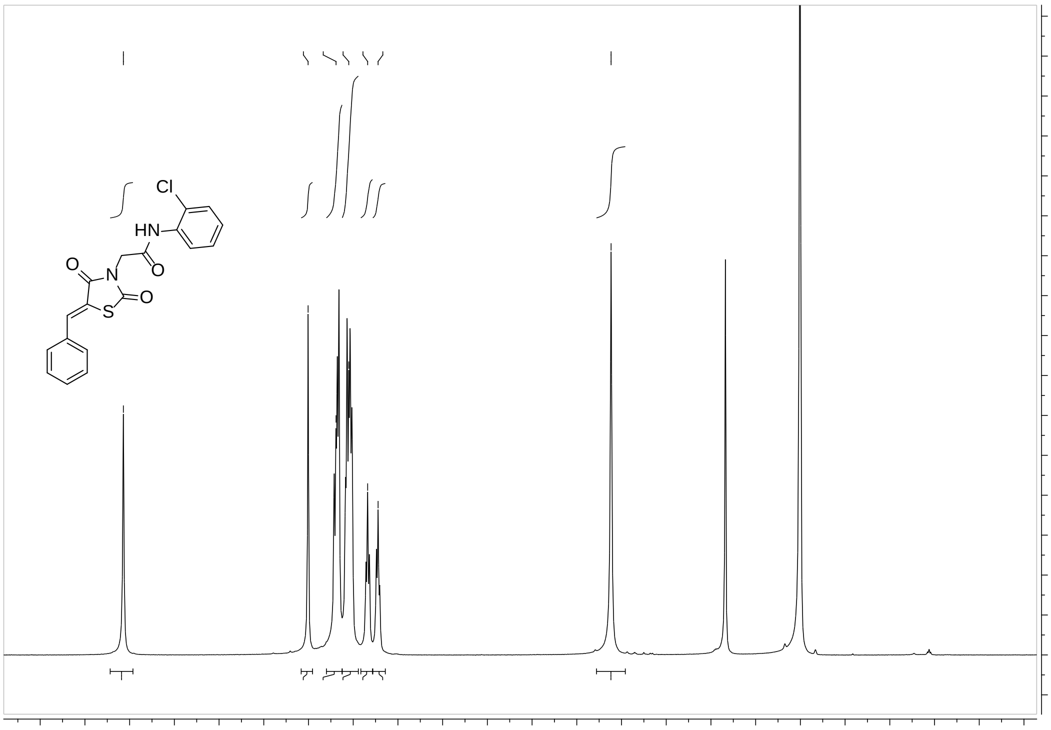


$^{1}$H-NMR spectrum of (*Z*)-2-(5-benzylidenethiazolidine-2,4-dion-3-yl)-N-(2-chlorophenyl)acetamide (**28a**)

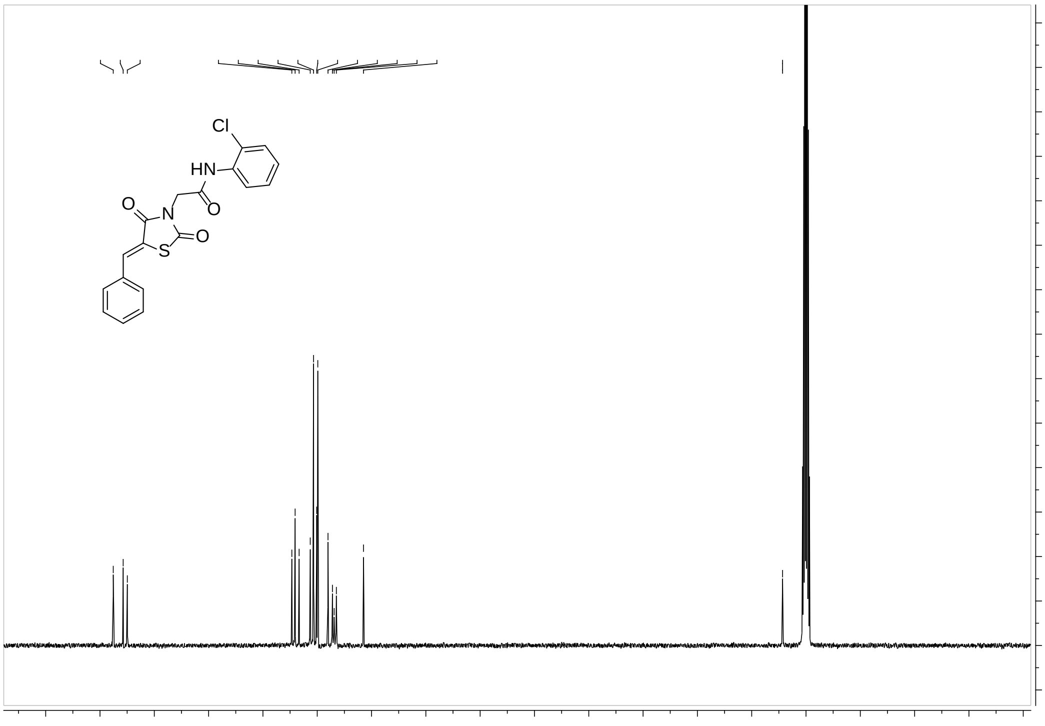


$^{13}$C-NMR spectrum of (*Z*)-2-(5-benzylidenethiazolidine-2,4-dion-3-yl)-N-(2-chlorophenyl)acetamide (**28a**)

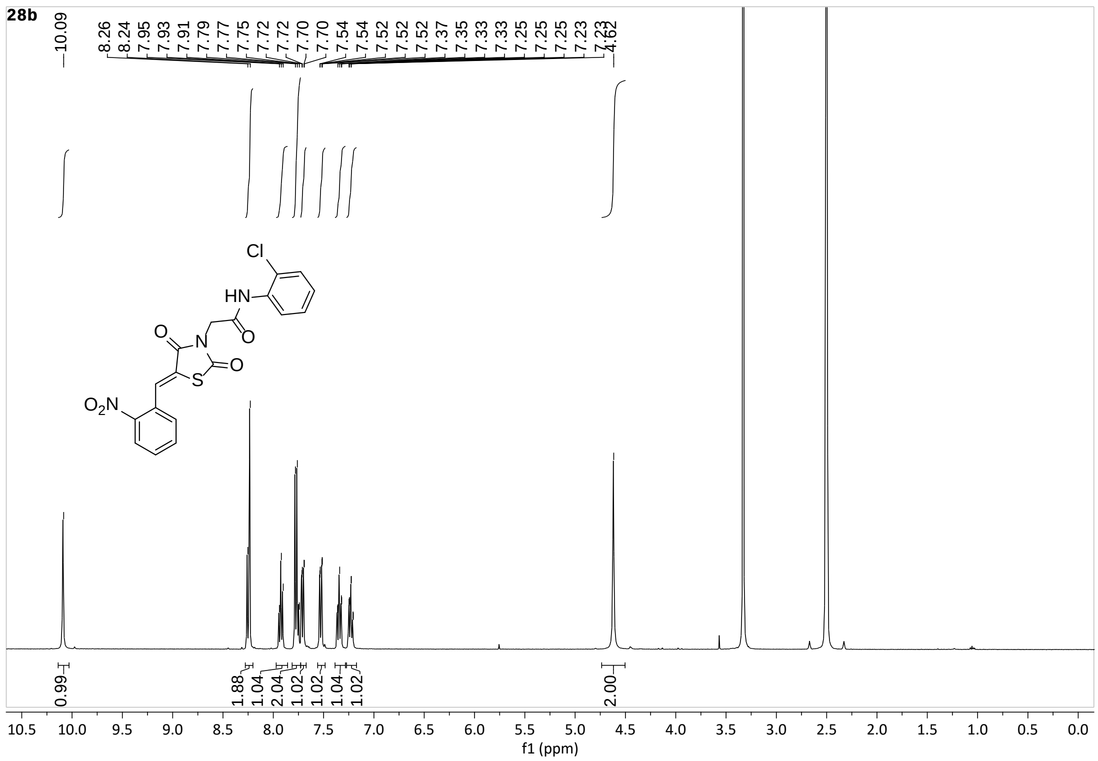

$^{1}$H-NMR spectrum of (*Z*)-*N*-(2-chlorophenyl)-2-(5-(2-nitrobenzylidene)thiazolidine-2,4-dion-3-yl)acetamide (**28b**)

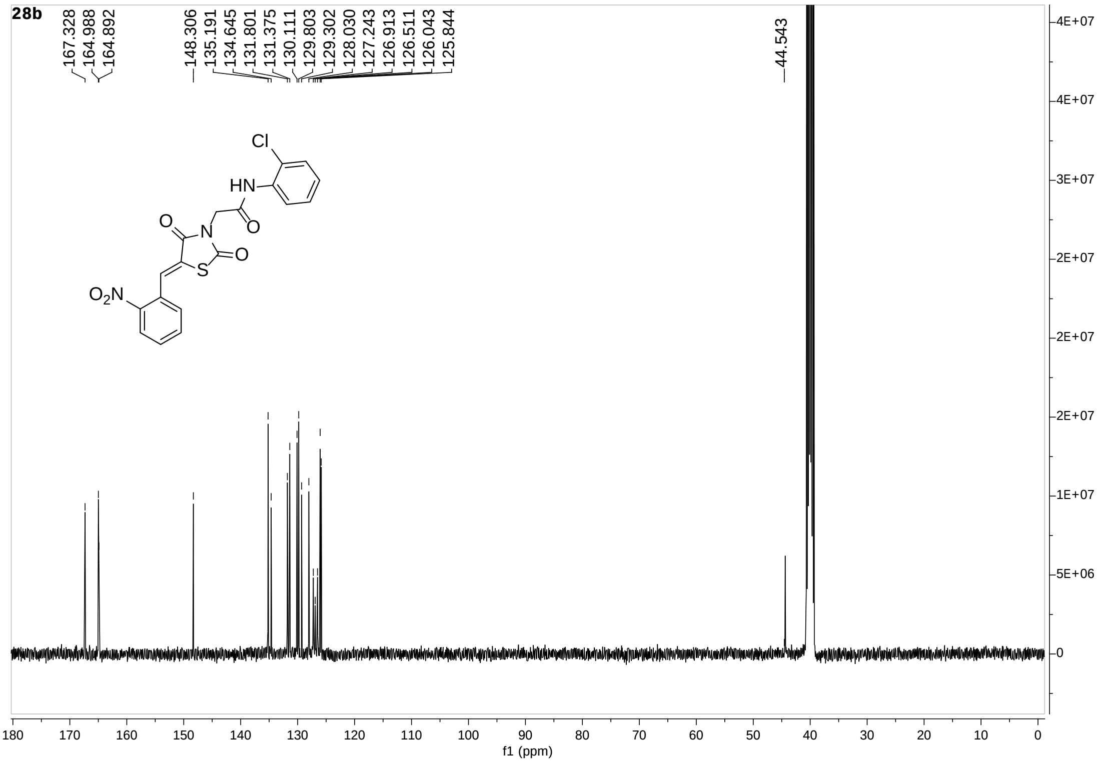

$^{13}$C-NMR spectrum of (*Z*)-*N*-(2-chlorophenyl)-2-(5-(2-nitrobenzylidene)thiazolidine-2,4-dion-3-yl)acetamide (**28b**)

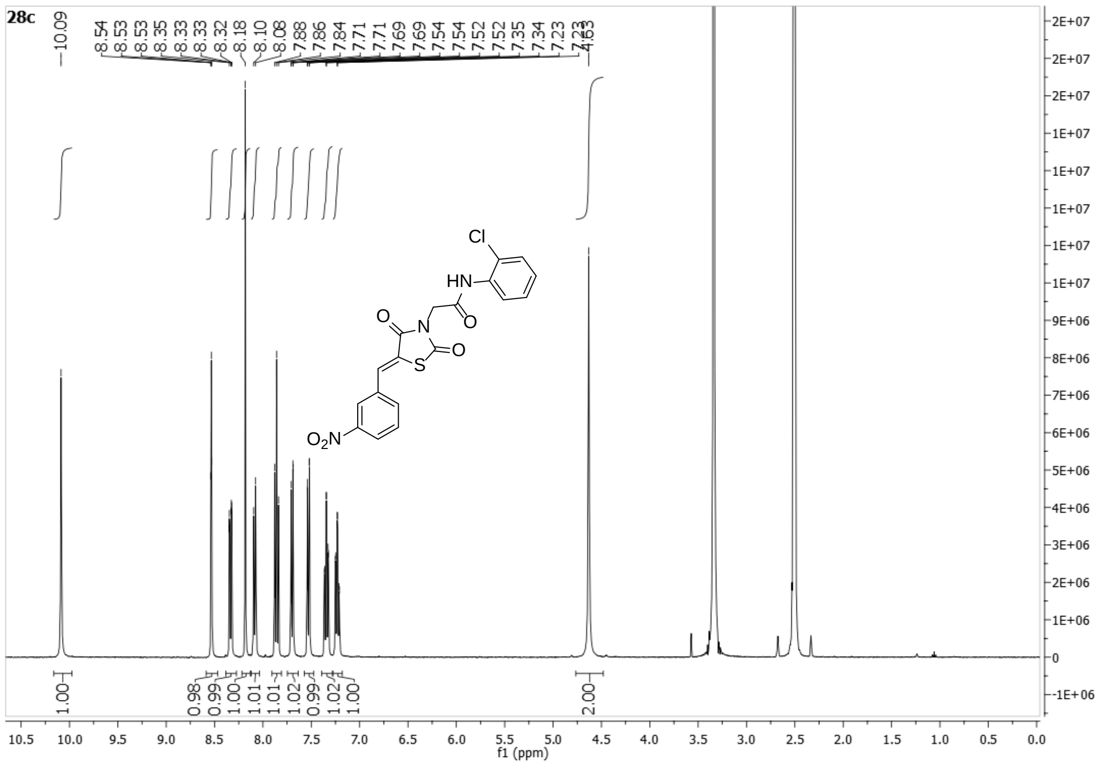


$^{1}$H-NMR spectrum of (*Z*)-*N*-(2-chlorophenyl)-2-(5-(3-nitrobenzylidene)thiazolidine-2,4-dion-3-yl)acetamide (**28c**)

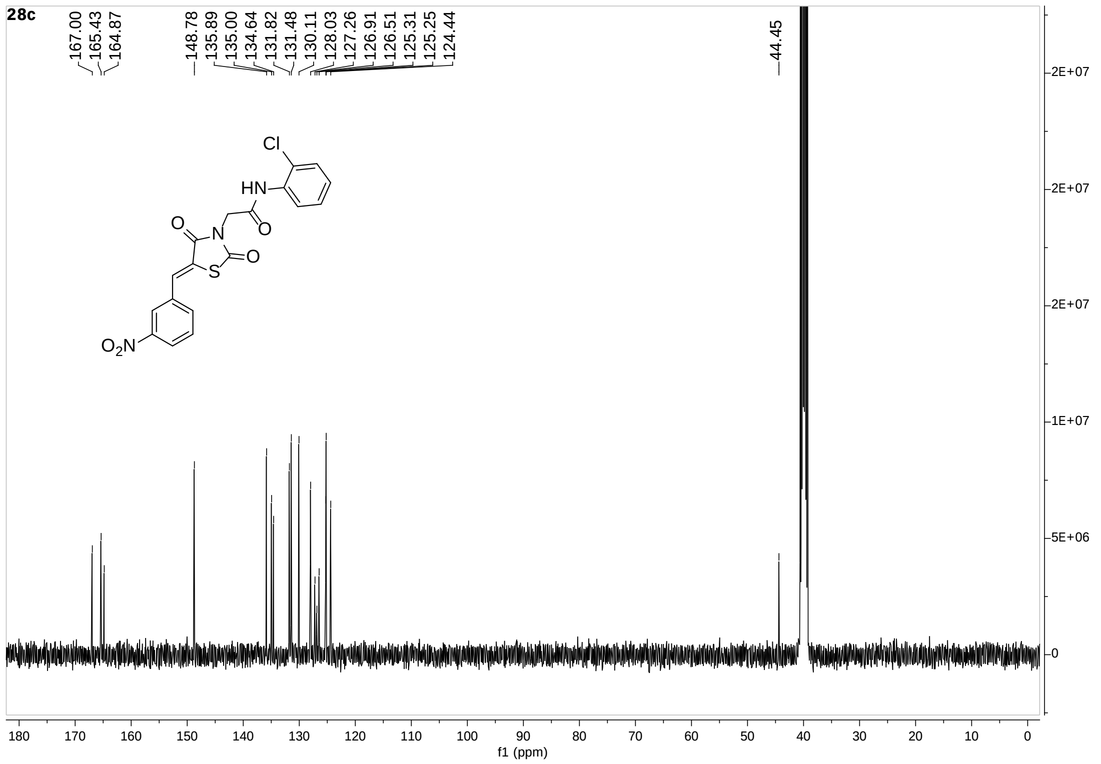


$^{13}$C-NMR spectrum of (*Z*)-*N*-(2-chlorophenyl)-2-(5-(3-nitrobenzylidene)thiazolidine-2,4-dion-3-yl)acetamide (**28c**)

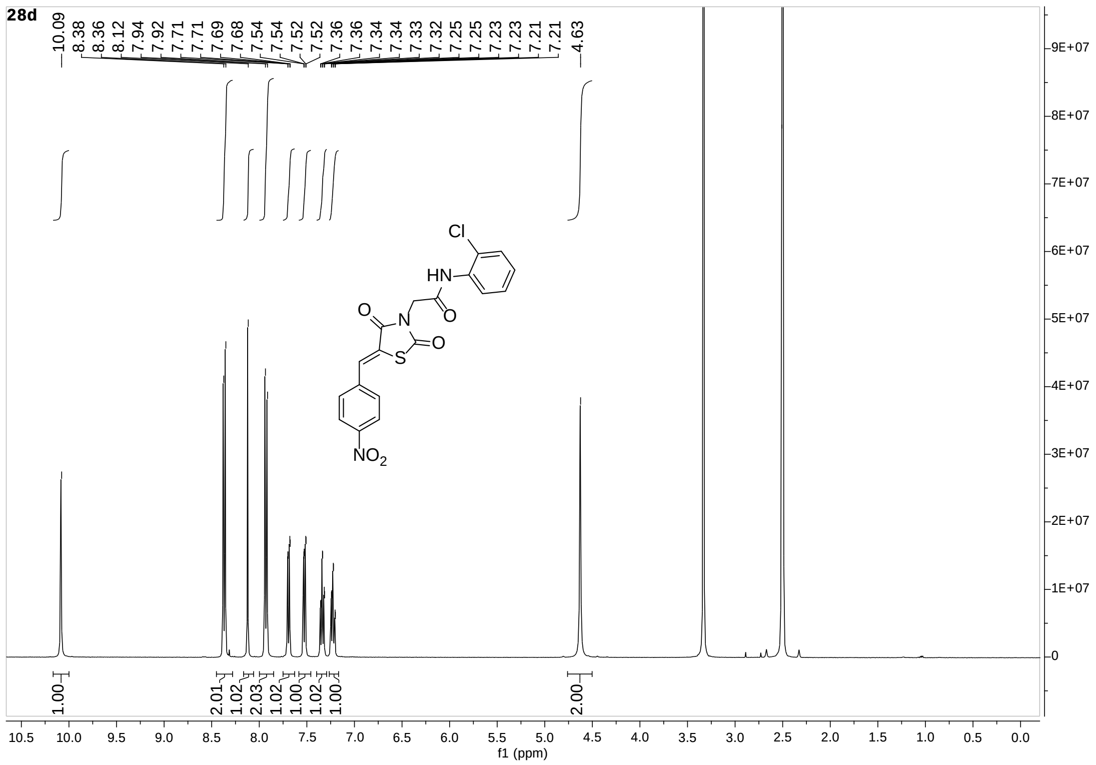

$^{1}$H-NMR spectrum of (*Z*)-*N*-(2-chlorophenyl)-2-(5-(4-nitrobenzylidene)thiazolidine-2,4-dion-3-yl)acetamide (**28d**)

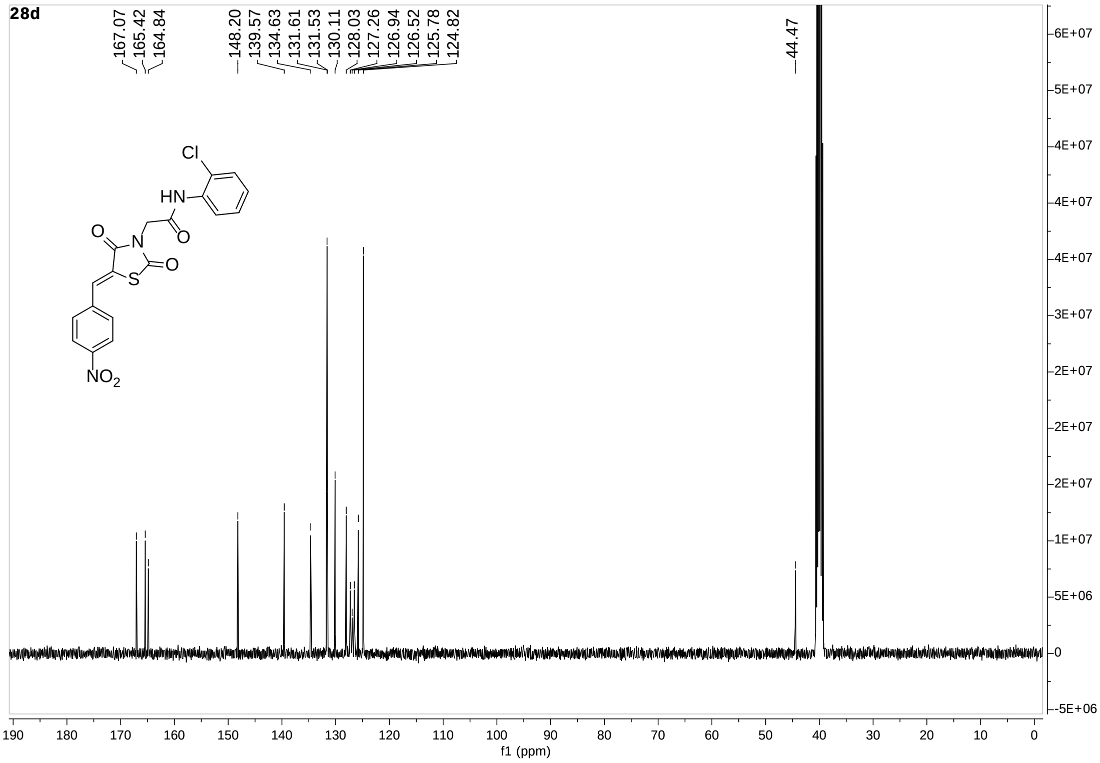

$^{13}$C-NMR spectrum of (*Z*)-*N*-(2-chlorophenyl)-2-(5-(4-nitrobenzylidene)thiazolidine-2,4-dion-3-yl)acetamide (**28d**)

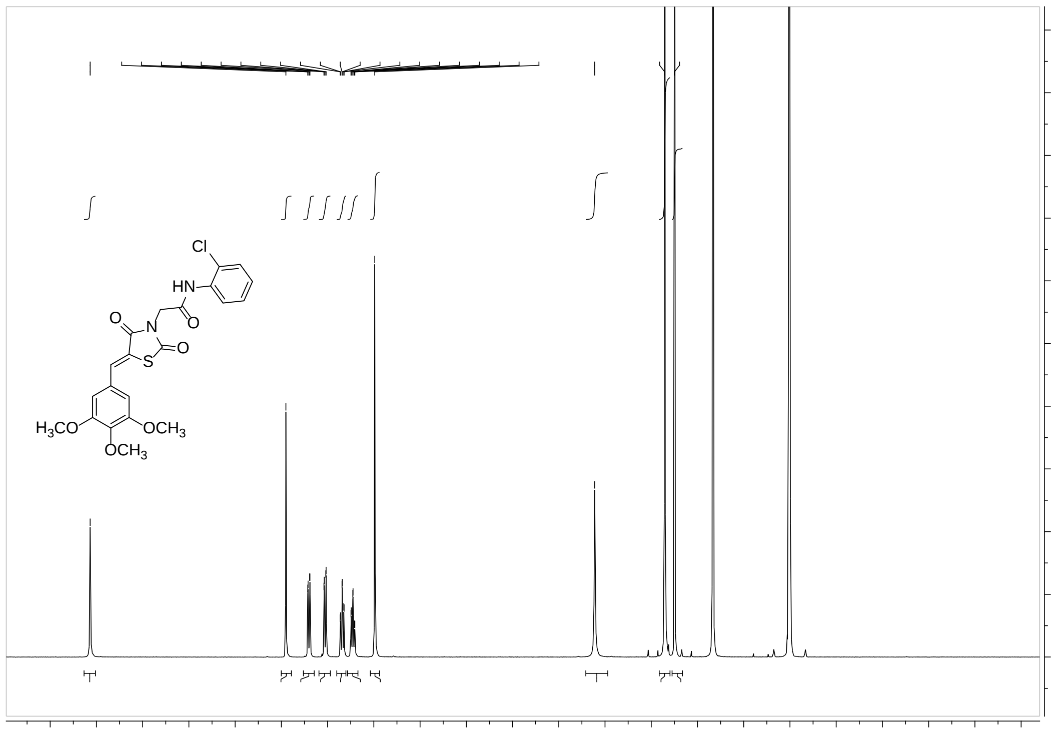


$^{1}$H-NMR spectrum of (*Z*)-*N*-(2-chlorophenyl)-2-(5-(3,4,5-trimethoxybenzylidene) thiazolidine-2,4-dion-3-yl)acetamide (**28e**)

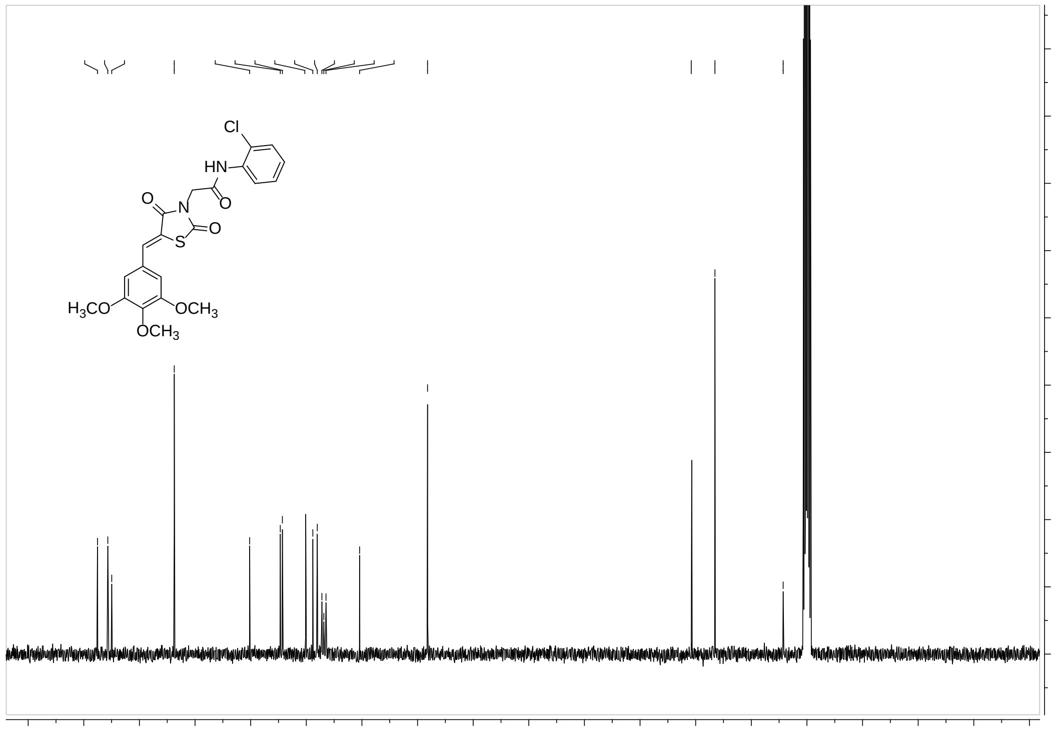


$^{13}$C-NMR spectrum of (*Z*)-*N*-(2-chlorophenyl)-2-(5-(3,4,5-trimethoxybenzylidene) thiazolidine-2,4-dion-3-yl)acetamide (**28e**)

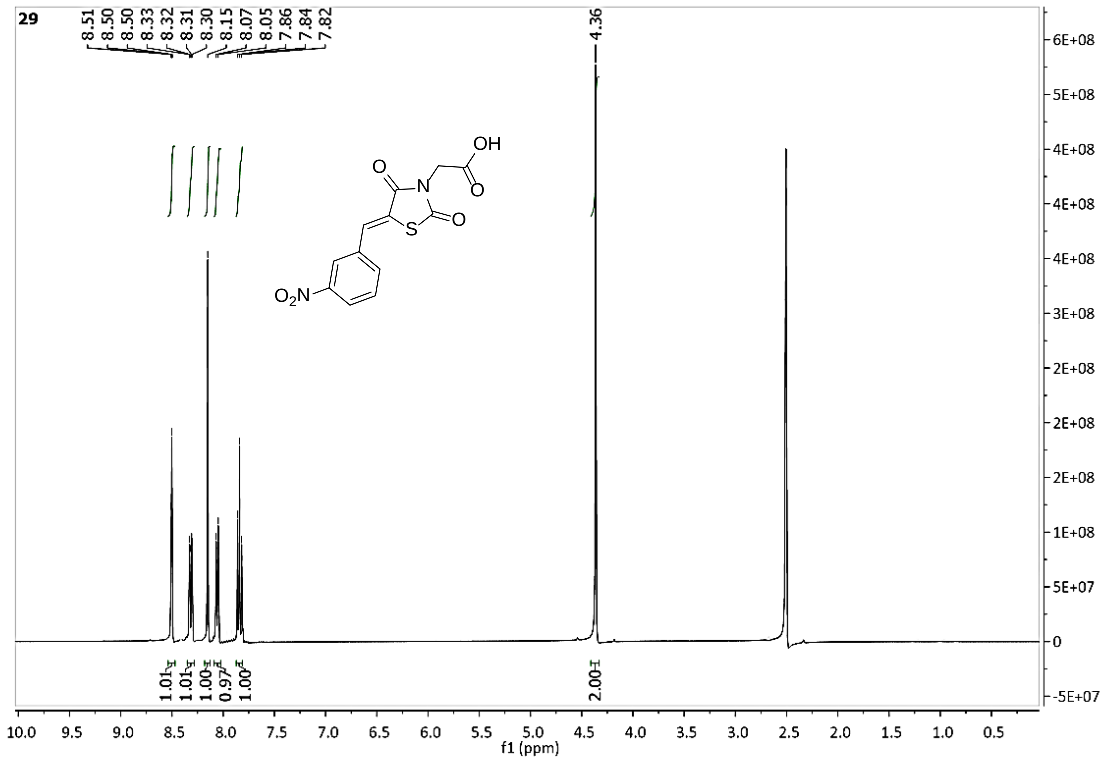


$^{1}$H-NMR spectrum of (*Z*)-2-(5-(3-Nitrobenzylidene)thiazolidin-2,4-dion-3-yl)acetic acid (**29**)

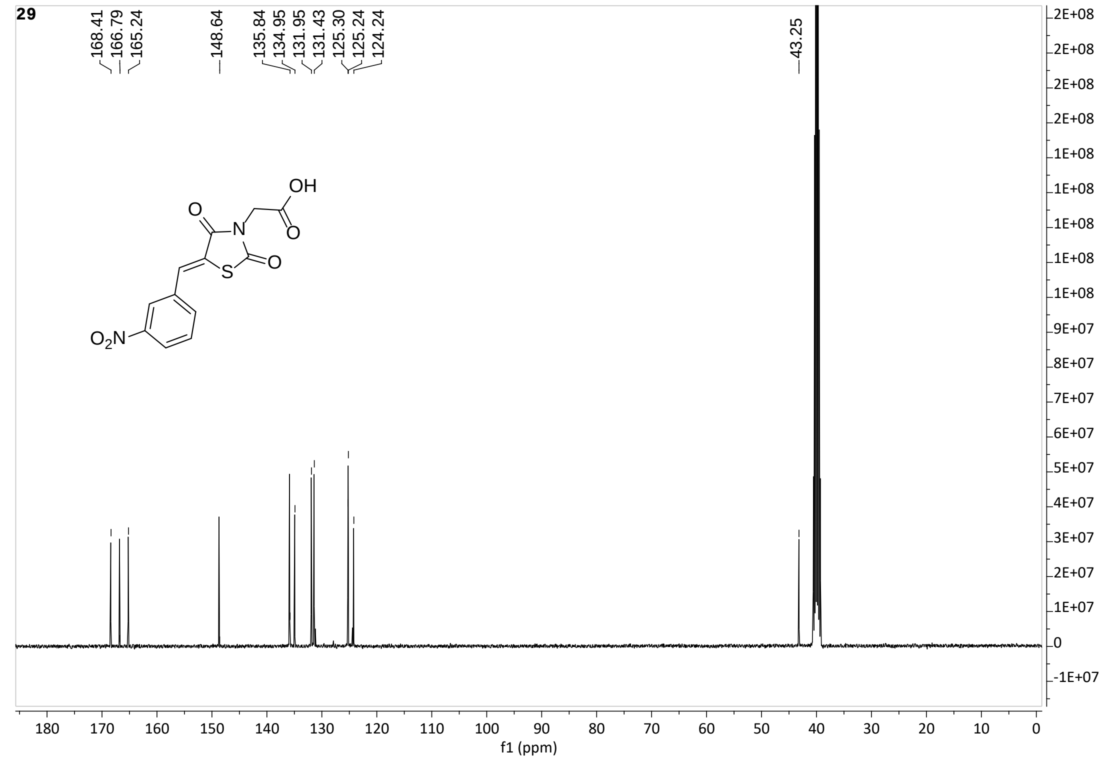


$^{13}$C-NMR spectrum of (*Z*)-2-(5-(3-Nitrobenzylidene)thiazolidin-2,4-dion-3-yl)acetic acid (**29**)
